# Epidemiological trends of women’s cancers from 1990 to 2019 at the global, regional, and national levels: a population-based study

**DOI:** 10.1186/s40364-021-00310-y

**Published:** 2021-07-07

**Authors:** Ming Yi, Tianye Li, Mengke Niu, Suxia Luo, Qian Chu, Kongming Wu

**Affiliations:** 1 Department of Oncology, Tongji Hospital of Tongji Medical College, Huazhong University of Science and Technology, 430030 Wuhan, China; 2 Department of Obstetrics and Gynecology, Tongji Hospital, Tongji Medical College, Huazhong University of Science and Technology, 430030 Wuhan, Hubei China; 3 Department of Medical Oncology, The Affiliated Cancer Hospital of Zhengzhou University & Henan Cancer Hospital, 450008 Zhengzhou, China

**Keywords:** Breast cancer, Cervical cancer, Ovarian cancer, Uterine cancer, Gynecological cancer, Cancer epidemiology

## Abstract

**Background:**

Every year around the world, more than 2 million women are diagnosed with breast cancer and genital tract cancers. However, there are rare studies comprehensively describing the global and regional trends of incidence and mortality of women’s cancers.

**Methods:**

To study the burden and trend of women’s cancers, we conducted this cross-sectional study based on the epidemiologic data of Global Burden of Disease 2019. In this study, female patients with breast cancer, cervical cancer, ovarian cancer, and uterine cancer worldwide from 1990 to 2019 were involved. The incidence, death, and disability-adjusted life-year (DALY) were used to measure the outcomes of women’s cancers. The estimated annual percentage change (EAPC) was calculated to assess the changing trend of cancer burden.

**Results:**

Among the four women’s cancers, the burden of female breast cancer was highest. During the past 30 years, the incidence, death, and DALY of female breast cancer kept increasing worldwide. In most regions especially developing countries, cervical cancer was the second most common women’s cancer. At the same time, ovarian cancer and uterine cancer occurred less frequently. Generally, the age-standardized incidence rates (ASIRs) of breast cancer, ovarian cancer, and uterine cancer were positively correlated to sociodemographic index (SDI) value. In contrast, the ASIR of cervical cancer was negatively correlated to SDI value.

**Conclusions:**

Our study indicates that the incidence and mortality of women’s cancers have geographical variations and change along with SDI value. The results might be helpful to policy-makers to allocate healthy resources to control women’s cancers.

**Supplementary Information:**

The online version contains supplementary material available at 10.1186/s40364-021-00310-y.

## Background

According to GLOBOCAN 2020 Project data, breast cancer, cervical cancer, ovarian cancer, and uterine cancer are among the top 10 common cancers in females worldwide [1]. It is estimated that female breast cancer has been the most common cancer, surpassing lung cancer globally [1]. There are significant geographical variations in the incidence rate of female breast cancer [1]. The incidence rate of female breast cancer in some developed regions (Australia, New Zealand, North America) is nearly three times higher than that in developing regions (South-central Asia, Middle Africa, East Africa) [1]. Also, among females, breast cancer is the leading cause of cancer deaths in most countries [1]. However, in some sub-Saharan Africa countries, breast cancer is preceded by cervical cancer, which causes the most cancer deaths [1]. Although cervical cancer could be prevented by HPV vaccination and screening pre-cancerous lesions, cervical cancer’s incidence and mortality are still high in these counties due to lacking essential health interventions [2]. Relative to cervical cancer, ovarian cancer and uterine cancer occur less frequently globally [3–5]. Generally, the incidences and deaths of four types of women’s cancers have continued to grow during the past decades.

Global Burden of Disease 2019 (GBD 2019) study provides the epidemiological data of 369 diseases and injuries in 204 countries or territories from 1990 to 2019, which updates based on GBD 2017 [6–8]. The incidences, deaths, disability-adjusted life-year (DALY) of diseases could be used to measure disease and economic burdens [9–14]. The epidemiological patterns of women’s cancers vary in different regions and change over time. A systematic analysis comprehensively reporting the variation trends of women’s cancers helps policy-makers measure the burden of women’s cancer, build health service infrastructures, and allocate public health resources. In this cross-sectional study, we reported the incidences, deaths, and disability-adjusted life-years (DALYs) of female breast cancer, cervical cancer, ovarian cancer, and uterine cancer from 1990 to 2019 in 204 countries. Besides, we analyzed the correlation between incidence rate or mortality rate and sociodemographic index (SDI).

## Methods

### Study Population and Data Collection

All data were obtained from GBD 2019 study, including incidences, deaths, DALY, age-standardized rate of incidence (ASIR), age-standardized rate of death (ASDR), and age-standardized rate of DALY from 1990 to 2019. SDI value is a parameter to evaluate a country’s development status, which is scored from 0 to 1 by calculating the geometric mean of fertility rate, length of education, and per capita income [15]. All countries or territories are classified into five groups according to SDI value: low SDI, low-middle SDI, middle SDI, high-middle SDI, and high SDI. SDI values of all countries from 1990 to 2019 were obtained from Institute for Health Metrics and Evaluation.

Because the study was based on publicly available dataset, this study was exempted by the ethics committee of Tongji Hospital, Tongji Medical College, Huazhong University of Science and Technology. This study followed the guideline of cross-sectional study described in the Guidelines for Accurate and Transparent Health Estimates Reporting (GATHER) [16].

### Statistical Analysis

Firstly, we analyzed the burden of women’s cancers at the global, geographic regional, and national levels. Additionally, to offset the differences in demographic and population number, we used the age-standardized rates of incidence, death, and DALY to explore incidence and mortality levels in different regions and countries. All rates were presented as numbers of per 100,000 persons.

Moreover, we assessed the variation trends of incidence and mortality by calculating estimated annual percentage changes (EAPCs) of age-standardized rates. In the calculating formula y = βx + α, x refers to calendar year, and y refers to log10 (age-standardized rate). EAPC was calculating as following: EAPC = 100* (10^β -1) [15, 17–19]. When EAPC value and its 95 % confidence interval (CI) are greater than zero, the age-standardized rate is upward. Conversely, the age-standardized rate is downward [20].

Besides, to investigate the potential effect of SDI on cancer burden, we analyzed the Spearman correlation between SDI value and age-standardized rates of incidence, death, and DALY [21]. All statistical analyses were conducted using R software (version 4.0.0, R Project for Statistical Computing). A two-tailed *P* value below 0.05 was regarded as statistical significance. Data visualization was performed using R software with package ggplot2.

## Results

### Female breast cancer

**Fig. 1:**
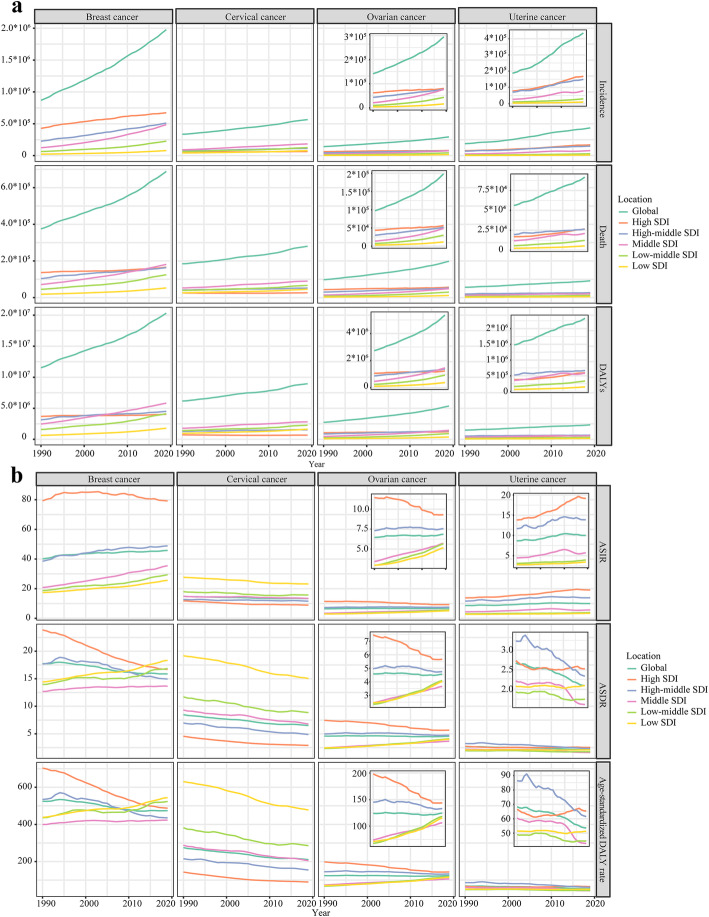
The changing trend of the burden of women’s cancers from 1990 to 2019. **a** The changing trends of incidence, death, and DALYs. **b** The changing trends of ASIR, ASDR, and age-standardized DALY rate. Note: DALY, disability adjusted life year; ASIR, Age-standardized incidence rate; ASDR, Age-standardized death rate

During the past 30 years worldwide, the incidences of female breast cancer increased from 867.62 × 10^3^ (95 %CI 840.4 × 10^3^~894.76 × 10^3^) to 1977.21 × 10^3^ (95 %CI 1807.61 × 10^3^~2145.21 × 10^3^) cases (Table S1). At the same time, the deaths of female breast cancer increased from 375.02 × 10^3^ (95 %CI 358.98 × 10^3^~390.82 × 10^3^) to 688.56 × 10^3^ (95 %CI 635.32 × 10^3^~739.57 × 10^3^) cases (Table S2), and the DALYs of female breast cancer increased from 11526.68 × 10^3^ (11021.13 × 10^3^~12107.83 × 10^3^) to 20310.19 × 10^3^ (95 %CI 18744.8 × 10^3^~21866.65 × 10^3^) years (Table S3). Globally, from 1990 to 2019, the incidences, deaths, and DALYs of female breast cancer were gradually increased **(**Fig. 1a**)**. For age-standardized rates, the ASIR of female breast cancer rose from 40.12 (95 %CI 38.78 ~ 41.33) in 1990 to 45.86 (95 %CI 41.91 ~ 49.76) in 2019, with EAPC_ASIR_: 0.36 (95 %CI 0.31 ~ 0.42) **(**Fig. 1b**)**. The ASDR of female breast cancer decreased from 17.76 (95 %CI 16.93 ~ 18.51) in 1990 to 15.88 (95 % CI 14.66 ~ 17.07) in 2019, with EAPC_ASDR_: -0.51 (95 %CI -0.57~-0.46). The age-standardized DALY rate of female breast cancer decreased from 524.87 (95 %CI 501.78 ~ 551.15) in 1990 to 473.83 (95 %CI 437.3 ~ 510.51) in 2019, with EAPC_age−standardized DALY rate_: -0.51 (95 %CI -0.57~-0.45).

The burden of female breast cancer varied in different regions. In 2019, the incidence number of female breast cancer was highest in high SDI region (673.15 × 10^3^, 95 %CI 601.27 × 10^3^~747.67 × 10^3^). However, the ASIR of female breast cancer was stable in high SDI region (79.3 with 95 %CI 77 ~ 80.87 in 1990; 79.22 with 95 %CI 70.83 ~ 87.7 in 2019; EAPC_ASIR_: -0.12, 95 %CI -0.22~-0.01). In contrast, the ASIR of female breast cancer was rapidly increased in middle SDI region (20.81 with 95 %CI 19.25 ~ 22.45 in 1990; 35.52 with 95 %CI 31.47 ~ 39.81 in 2019; EAPC_ASIR_: 1.87, 95 %CI 1.84 ~ 1.9). Relative to ASIR, at the same time, the ASDR and age-standardized DALY rate were significantly declined in high SDI region: EAPC_ASDR_: -1.36, 95 %CI -1.41~-1.31; EAPC_age−standardized DALY rate_: -1.41, 95 %CI -1.47~-1.36.

**Fig. 2:**
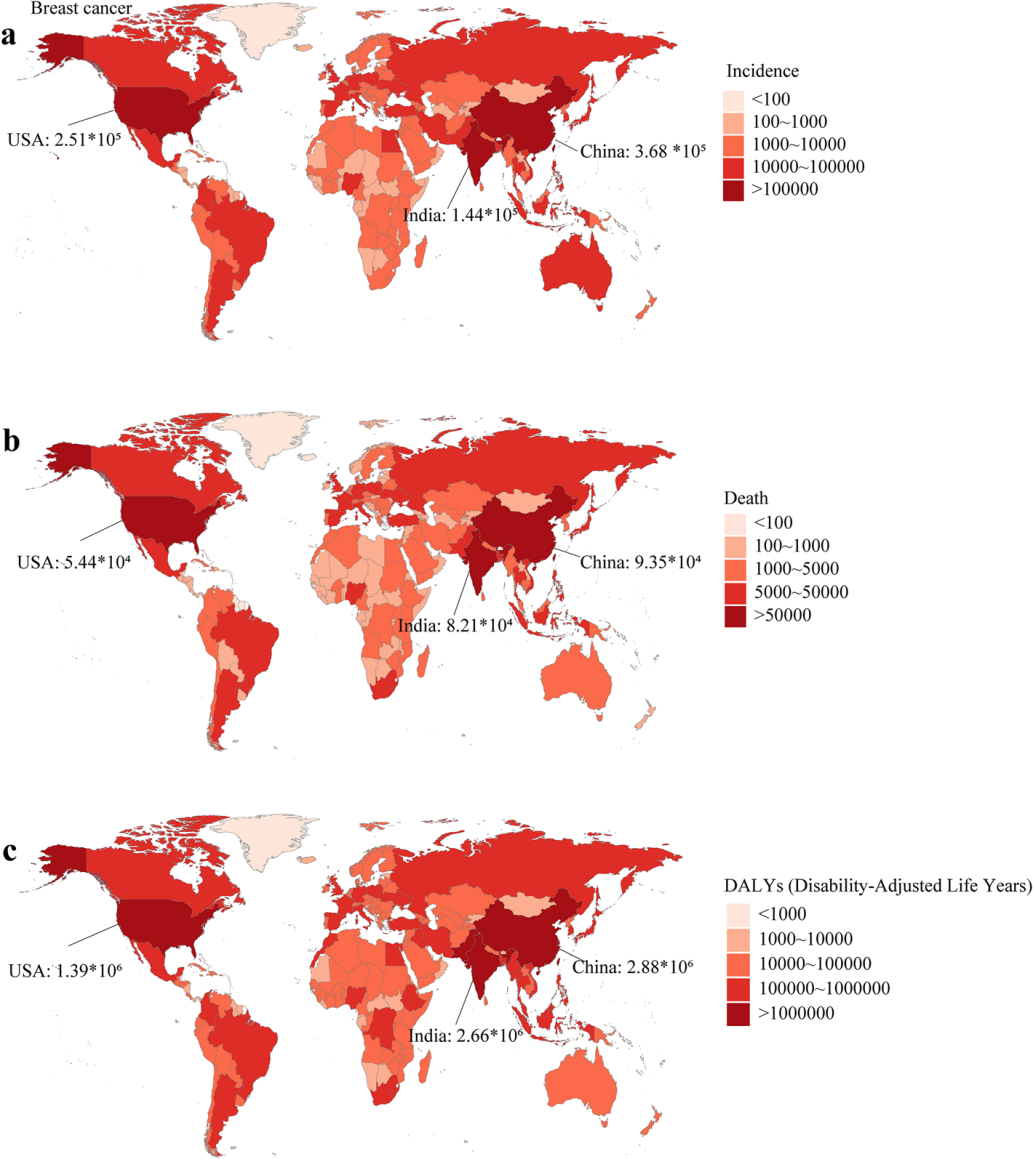
The burden of breast cancer in 204 countries or territories in 2019. **a** The incidence of breast cancer in 204 countries or territories; **b** The death of breast cancer in 204 countries or territories; **c** The DALYs of breast cancer in 204 countries or territories. Note: DALY, disability adjusted life year

In 1990, the USA, China, Germany were the top three countries with the most incidence cases. In 2019, China, the USA, and India were the countries with the most incidence cases (368.37 × 10^3^, 251.53 × 10^3^, 144.09 × 10^3^; respectively) (Fig. 2a). In 2019, China, India, and the USA were the countries with the most deaths (93.50 × 10^3^, 82.10 × 10^3^, 54.40 × 10^3^; respectively) (Fig. 2b) and most DALYs (2877.24 × 10^3^, 2659.24 × 10^3^, 1387.67 × 10^3^; respectively) (Fig. 2c). Monaco was the country with the highest ASIR in 2019 (149.60, 95 %CI 108.67 ~ 193.81). The ASIR was relatively high in the USA in 2019 (94.21, 95 %CI 77.35 ~ 115.12) (Figure S1a). Solomon Islands was the country with the highest ASDR and age-standardized DALY rate in 2019 (75.04, 95 %CI 59.00 ~ 94.48; 2635.74, 2026.64 ~ 3377.14; respectively). Pakistan had a relatively high ASDR and age-standardized DALY rate in 2019 (51.94, 95 %CI 39.03 ~ 69.76; 1570.06, 1177.2 ~ 2135.47; respectively) (Figure S1b and Figure S2c).

### Cervical cancer

Globally, the incidence cases of cervical cancer increased from 335.64 × 10^3^ (95 %CI 300.35 × 10^3^~393.89 × 10^3^) in 1990 to 565.54 × 10^3^ (95 %CI 481.52 × 10^3^~636.43 × 10^3^) (Table S4). In the same period, the deaths of cervical cancer increased from 184.53 × 10^3^ (95 %CI 164.84 × 10^3^~218.94 × 10^3^) to 280.48 × 10^3^ (95 %CI 238.86 × 10^3^~313.93 × 10^3^) (Table S5), and the DALYs of cervical cancer increased from 6176.25 × 10^3^ (95 %CI 5437.67 × 10^3^~7316.93 × 10^3^) to 8955.01 × 10^3^ (95 %CI 7547.73 × 10^3^~9978.46 × 10^3^) (Table S6). The incidences, deaths, and DALYs of cervical cancer kept steadily increased from 1990 to 2019 in the world (Fig. 1a). However, the global incidence rate and mortality rate gradually decreased in the past three decades: EAPC_ASIR_: -0.38, 95 %CI -0.41~-0.34; EAPC_ASDR_: -0.93, 95 %CI -0.98~-0.88; EAPC_age−standardized DALY rate_: -0.95, 95 %CI -1.00~-0.89 (Fig. 1b).

Middle SDI region had the highest cervical cancer burden in 2019: 183.34 × 10^3^ (95 %CI 144.49 × 10^3^~208.86 × 10^3^) incidence cases, 90.1 × 10^3^ (95 %CI 71.33 × 10^3^~103.2 × 10^3^) deaths, and 2817.25 × 10^3^ (2223.19 × 10^3^~3217.72 × 10^3^) DALYs. Low SDI region had the highest incidence rate and mortality rate in 2019: ASIR: 23.21, 95 %CI 18.31 ~ 28.76; ASDR: 15.05, 95 %CI 11.92 ~ 18.46; age-standardized DALY rate: 477.53, 95 %CI 374.33 ~ 591.38. High SDI region had the lowest incidence rate and mortality rate in 2019: ASIR: 8.91, 95 %CI 7.74 ~ 9.99; ASDR: 2.9, 95 %CI 2.6 ~ 3.1; age-standardized DALY rate: 89.72, 95 %CI 81.88 ~ 95.85. Besides, high SDI region had the most rapid decline in incidence rate and mortality rate: EAPC_ASIR_: -0.95, 95 %CI -1.06~-0.85; EAPC_ASDR_: -1.57, 95 %CI -1.68~-1.46; EAPC_age−standardized DALY rate_: -1.62, 95 %CI -1.75~-1.49.

**Fig. 3:**
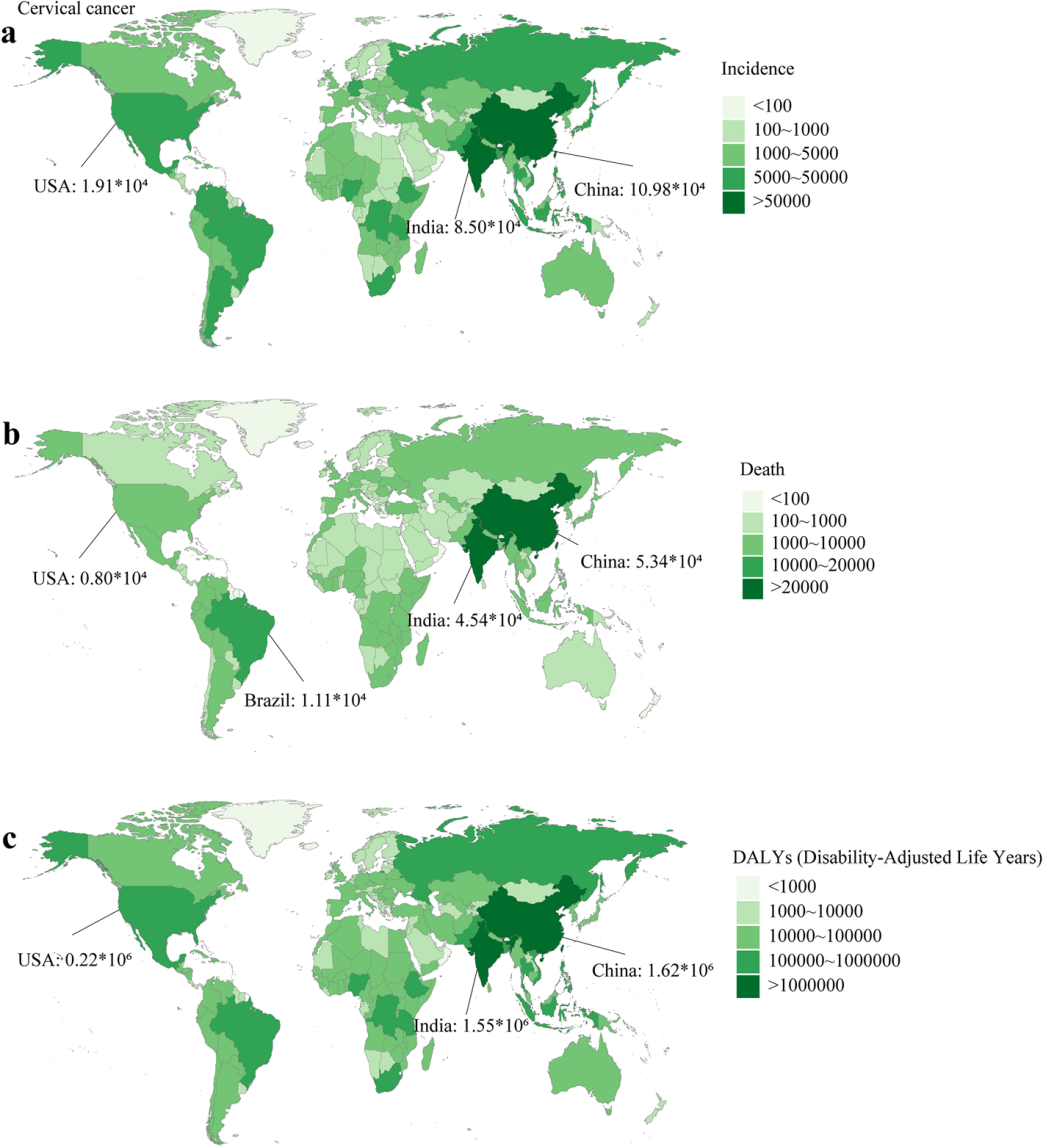
The burden of cervical cancer in 204 countries or territories in 2019. **a** The incidence of breast cancer in 204 countries or territories; **b** The death of cervical cancer in 204 countries or territories; **c** The DALYs of cervical cancer in 204 countries or territories. Note: DALY, disability adjusted life year

China, India, Brazil were top three countries with the most incidence cases in 2019 (109.76 × 10^3^, 84.98 × 10^3^, 22.65 × 10^3^, respectively) (Fig. 3a), followed by the USA (19.11 × 10^3^, ranking fourth). Similarly, China, India, Brazil were top three countries with the most deaths and DALYs in 2019 (deaths: 53.44 × 10^3^, 45.45 × 10^3^, 11.07 × 10^3^, respectively; DALYs: 1622.24 × 10^3^, 1554.49 × 10^3^, 348.42 × 10^3^, respectively), followed by the USA (deaths: 7.99 × 10^3^, ranking fifth; DALYs: 224.78 × 10^3^, ranking sixth) (Fig. 3b and c). Kiribati had the highest age-standardized rates of incidence, death, and DALY in 2019 (ASIR: 108.8, 95 %CI 78.79 ~ 140.73; ASDR: 69.52, 95 %CI 51.09 ~ 88.92; age-standardized DALY rate: 2143.06, 95 %CI 1534.43 ~ 2781.26). In general, the incidence rate and mortality rate were low in China (ASIR: 11.01, ASDR: 5.13, age-standardized DALY rate: 157.5), India (ASIR: 13.1, ASDR: 7.38, age-standardized DALY rate: 239.49), and the USA (ASIR: 8.67, ASDR: 3.05, age-standardized DALY rate: 98.77) (Figure S2).

### Ovarian cancer

Relative to incidence cases of female breast cancer and cervical cancer, the incidence cases of ovarian cancer were much lower worldwide. In 2019, the incidence of ovarian cancer was 294.42 × 10^3^ (95 %CI 260.65 × 10^3^~329.73 × 10^3^) worldwide (Table S7). The deaths of ovarian cancer were 198.41 × 10^3^ (95 %CI 175.36 × 10^3^~217.66 × 10^3^), and the DALYs of ovarian cancer were 5359.74 × 10^3^ (4692.95 × 10^3^~5954.99 × 10^3^) in 2019 globally (Table S8 and Table S9). From 1990 to 2019, the incidence and mortality rates of ovarian cancer remained stable at the global level (EAPC_ASIR_: 0.11, EAPC_ASDR_: -0.11, EAPC_age−standardized DALY rate_: -0.08).

Although high SDI region had the highest ovarian cancer burden and mortality from 1990 to 2019, the age-standardized rates of incidence, death, and DALY in high SDI region were promptly decreased: EAPC_ASIR_: -0.87, 95 %CI -0.96~-0.78; EAPC_ASDR_: -1.1, 95 %CI -1.17~-1.02; EAPC_age−standardized DALY rate_: -1.27, 95 %CI -1.34~-1.2. In contrast, despite having a relatively low ovarian cancer burden and mortality, low-middle SDI region and low SDI region had the most rapid increase in age-standardized rates of incidence, death, and DALY (Table S8 and Table S9).

**Fig. 4:**
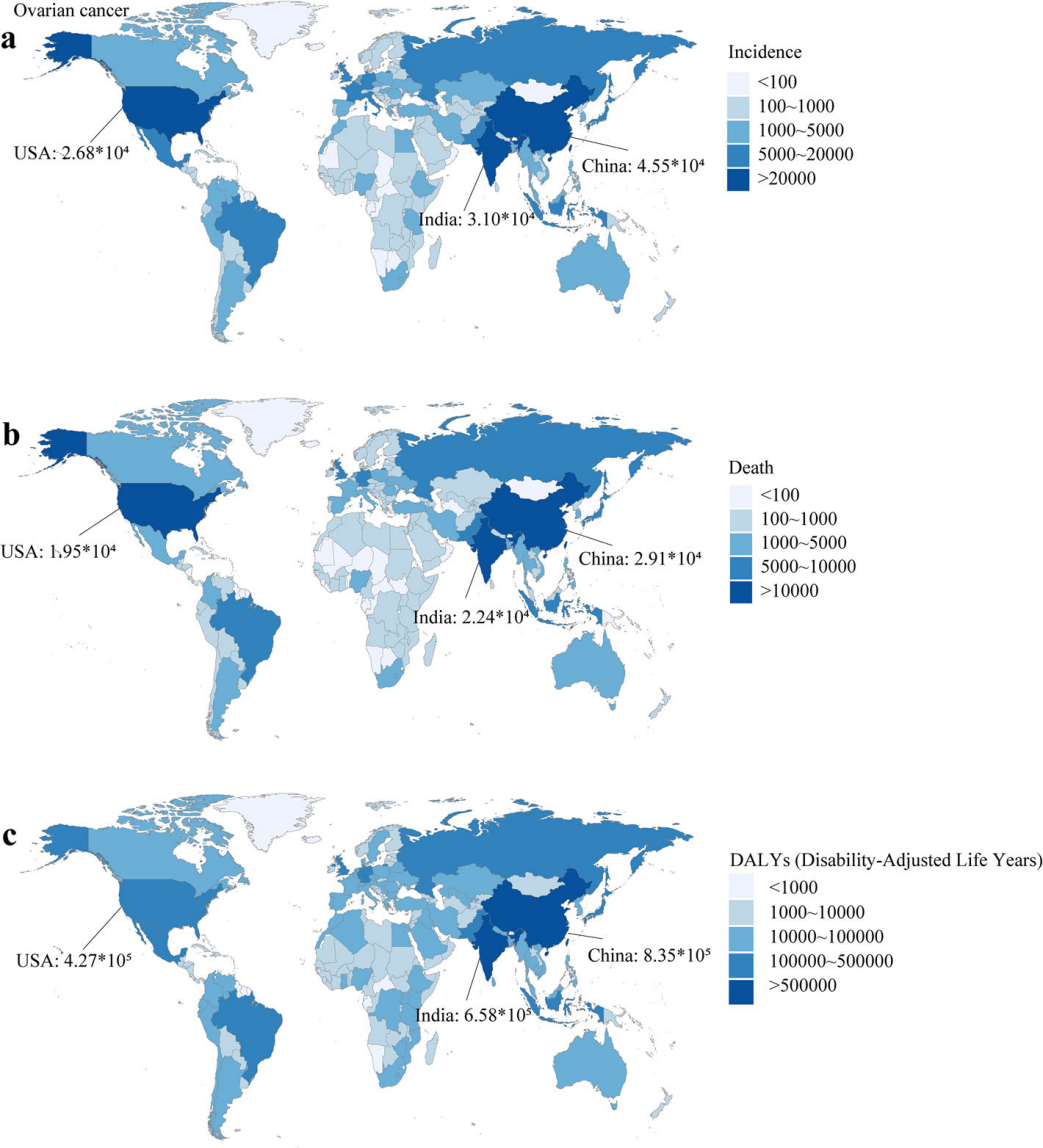
The burden of ovarian cancer in 204 countries or territories in 2019. **a** The incidence of ovarian cancer in 204 countries or territories; **b** The death of ovarian cancer in 204 countries or territories; **c** The DALYs of ovarian cancer in 204 countries or territories. Note: DALY, disability adjusted life year

China, India, and the USA were the top three countries with the most ovarian cancer incidence cases (China: 45.48 × 10^3^, India: 31.02 × 10^3^, the USA: 26.79 × 10^3^) (Fig. 4a), deaths (China: 29.09 × 10^3^, India: 22.35 × 10^3^, the USA: 19.5 × 10^3^) (Fig. 4b), and DALYs (China: 835.06 × 10^3^, India: 657.74 × 10^3^, the USA: 426.5 × 10^3^) in 2019 (Fig. 4c). Monaco had the highest ASIR (22.75, 95 %CI 16.73 ~ 28.89) and ASDR (13.67, 95 %CI 9.78 ~ 17.24), and Pakistan had the highest age-standardized DALY rate (348.37, 95 %CI 182.72 ~ 562.44) in 2019. Among China, India, and the USA, the USA had a relatively high age-standardized rates of incidence, death, and DALY in 2019 (Figure S3).

### Uterine cancer

Around the world, the incidence cases of uterine cancer elevated from 187.19 × 10^3^ (174.63 × 10^3^~196.03 × 10^3^) in 1990 to 435.04 × 10^3^ (397.02 × 10^3^~479.73 × 10^3^) in 2019 (Table S10). In the same period, the global deaths increased from 56.13 × 10^3^ (95 %CI 51.1 × 10^3^~60.2 × 10^3^) to 91.64 × 10^3^ (95 %CI 82.39 × 10^3^~101.5 × 10^3^), and the global DALYs increased from 1483.29 × 10^3^ (95 %CI 1317.51 × 10^3^~1612.75 × 10^3^) to 2329.07 × 10^3^ (95 %CI 2092.95 × 10^3^~2560.89 × 10^3^) (Table S11 and Table S12). At the global level, the incidence rate of uterine cancer was increased, but the mortality rate was decreased during the past three decades (EAPC_ASIR_: 0.69, 95 %CI 0.57 ~ 0.81; EAPC_ASDR_: -0.85, 95 %CI -0.93~-0.76; EAPC_age−standardized DALY rate_: -0.84, 95 %CI -0.93~-0.75).

High SDI region had the most uterine incidence cases (168.02 × 10^3^, 95 %CI 148.61 × 10^3^~188.55 × 10^3^) in 2019 and the most rapidly increased ASIR (EAPC_ASIR_: 1.37, 95 %CI 1.28 ~ 1.46) from 1990 to 2019. Also, high SDI region had the most deaths (26.63 × 10^3^, 95 %CI 24 × 10^3^~28.14 × 10^3^) and the highest ASDR (2.52, 95 %CI 2.32 ~ 2.64) and age-standardized DALY rate (65.31, 95 %CI 60.99 ~ 69.65) in 2019.

**Fig. 5:**
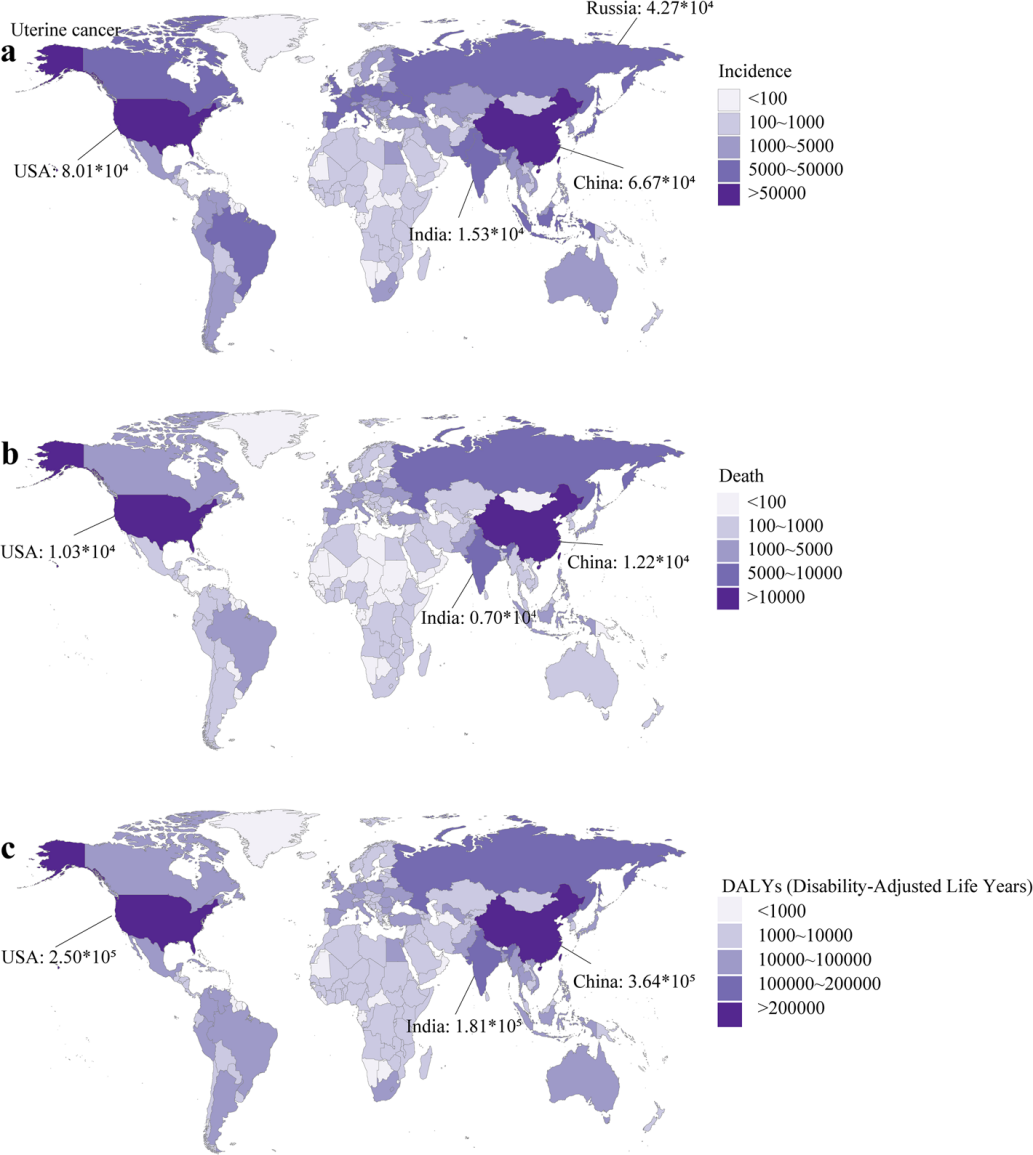
The burden of uterine cancer in 204 countries or territories in 2019. **a** The incidence of uterine cancer in 204 countries or territories; **b** The death of uterine cancer in 204 countries or territories; **c** The DALYs of uterine cancer in 204 countries or territories. Note: DALY, disability adjusted life year

The USA, China, and Russia were the top three countries with the most incidence cases (80.07 × 10^3^, 66.74 × 10^3^, 42.71 × 10^3^, respectively) in 2019, followed by India (15.26 × 10^3^, ranking sixth) (Fig. 5a). China, the USA, India had the most deaths (12.22 × 10^3^, 10.26 × 10^3^, 7.04 × 10^3^, respectively) and DALYs (364.28 × 10^3^, 249.56 × 10^3^, 180.91 × 10^3^) in 2019 (Fig. 5b and Fig. 5c). Northern Mariana Islands had the highest ASIR (32.77, 95 %CI 21.3 ~ 42.36), Grenada had the highest ASDR (11.3, 95 %CI 9.79 ~ 12.99), and American Samoa had the highest age-standardized DALY rate (285.41, 95 %CI 185.19 ~ 370.95) in 2019. Compared to China and India, the USA had a higher age-standardized rates of incidence, death, and DALY in 2019 (Figure S4). The age-standardized rates of women’s cancers in all countries or territories were summarized on Table S13.

### The incidence, death, and DALY of women’s cancers and demographic

In 2019, there were rare women’s cancer patients aged less than 25 worldwide (Figure S5a). For breast cancer, the global incidence case and death number reached the peak at the age of 50 ~ 70. Besides, the death number of breast cancer reached the second peak at the age older than 80, and the DALY of breast cancer reached the peak at the age of 50 ~ 55. The ASIR and ASDR of breast cancer increased with age, reaching the peak at the age older than 80 (Figure S5b). The age-standardized DALY rate elevated with age as well, but it reached the plateau after the age of 55.

The global incidence of cervical cancer was in peak at the age of 40 ~ 55 (Figure S5a). The global death of cervical cancer reached a peak at the age of 50 ~ 60. The global DALY of cervical cancer peaked at the age of 50 ~ 55. The global ASIR of cervical cancer reached the plateau after the age of 40, and the global ASDR of cervical cancer increased with age. The global age-standardized DALY rate peaked at the age of 55 ~ 60 (Figure S5b).

The global incidence of ovarian cancer reached a plateau after the age of 50 (Figure S5a). The global death of cervical cancer climbed the first peak at the age of 65 ~ 70 and reached the second peak after 80. The global DALY of ovarian cancer was in peak at the age of 50 ~ 65. The global ASIR and ASDR of ovarian cancer increased with age. The global age-standardized DALY rate of ovarian cancer peaked at the age of 65 ~ 75 (Figure S5b).

The global incidence of uterine cancer was in peak at the age of 55 ~ 65 (Figure S5a). The global death of uterine cancer reached the first peak at the age of 65 ~ 70 and reached the second peak after the age of 80. The global DALY of uterine cancer peaked at the age of 60 ~ 65. The global ASIR and age-standardized DALY rate of uterine cancer were in peak at the age of 70 ~ 75 (Figure S5b). The global ASDR of uterine cancer increased with age.

Generally, the demographic characteristics of women’s cancer patients were similar in regions with different SDI values except for the ratio of patients aged older than 80 (Figure S6, S7, S8, S9, S10, S11, S12, S13, S14, S15). In high SDI region, there was a much higher ratio of patients aged older than 80, especially compared to the data of low SDI region.

### The correlations between SDI and rates of incidence and mortality

**Fig. 6:**
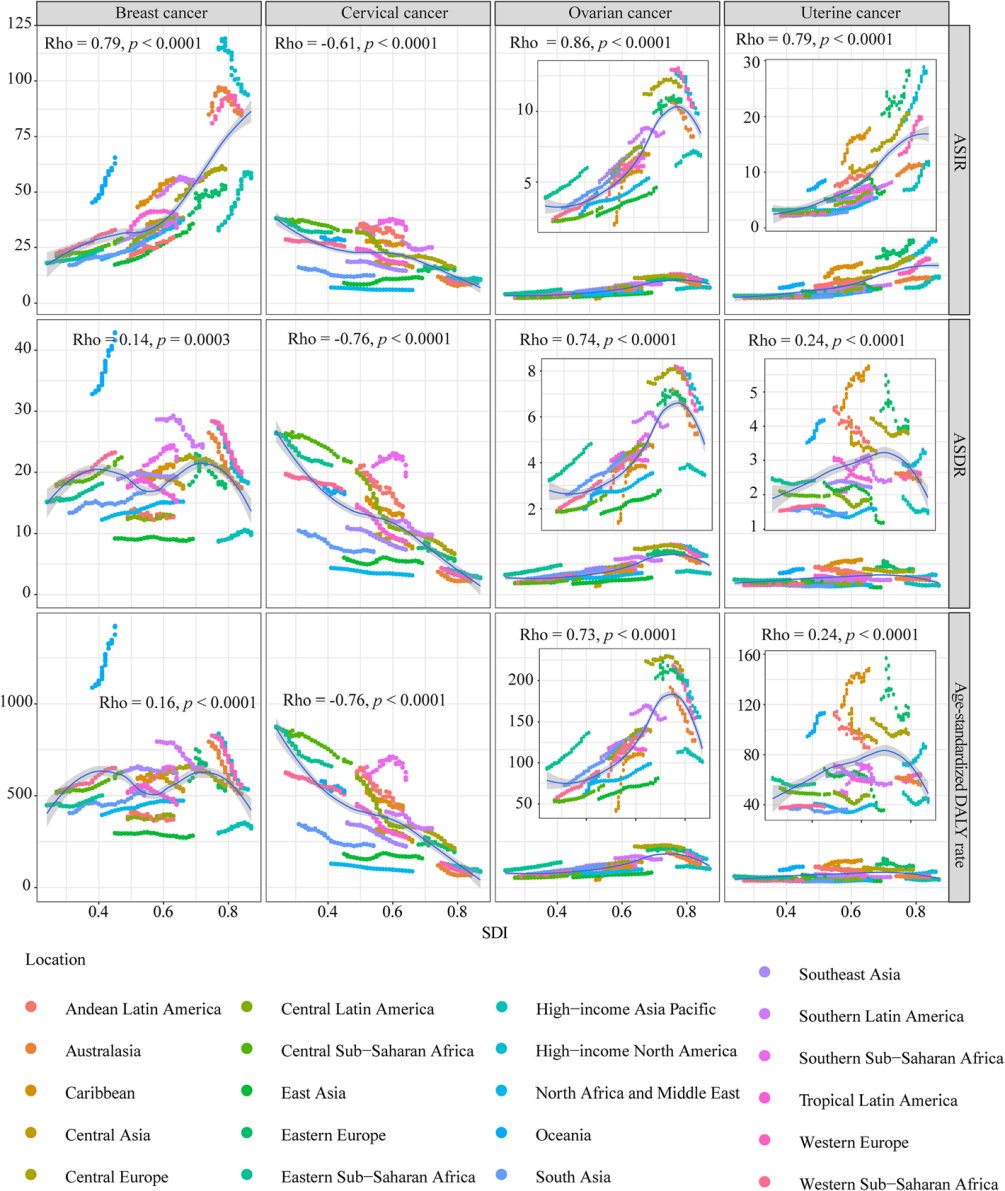
The correlation analyses of age-standardized rates and SDI from 1990 to 2019. The correlations between ASIR/ASDR/age-standardized DALY rate and SDI were analyzed based the data of 21 regions. Note: ASIR, Age-standardized incidence rate; ASDR, Age-standardized death rate; DALY, disability adjusted life year

The ASIRs of breast cancer were significantly positively correlated with SDI value (ρ: 0.79, *P* < 0.0001) **(**Fig. 6**)**. The ASDR and age-standardized DALY rate of breast cancer exhibited non-linear correlations with SDI value. When SDI value was greater than 0.7, the ASDR and age-standardized DALY rate of breast cancer sharply decreased with the increase of SDI. In contrast, for cervical cancer, the age-standardized rates of incidence, death, and DALY were negatively correlative with SDI value (ρ: -0.61, *P* < 0.0001; ρ: -0.76, *P* < 0.0001; ρ: -0.76, *P* < 0.0001; respectively).

For ovarian cancer, generally, the age-standardized rates of incidence, death, and DALY were positively correlative with SDI value (ρ: 0.86, *P* < 0.0001; ρ: 0.74, *P* < 0.0001; ρ: 0.73, *P* < 0.0001; respectively). However, when SDI was more than 0.7, the ASIR, ASDR and age-standardized DALY rate rapidly declined with SDI increase. For uterine cancer, the ASIR was significantly positively correlated with SDI value (ρ: 0.79, *P* < 0.0001). The correlation between mortality rate and SDI in uterine cancer was similar to that in ovarian cancer.

## Discussion

In the present study, we reported the incidences and mortalities of female breast cancer, cervical cancer, ovarian cancer, and uterine cancer. Around the world, the total incidence cases and death cases of the four types of cancers increased sharply from 1990 to 2019. Excluding the increased population and changed demographic, the ASIRs and ASDRs of the four types of cancers exhibited different epidemiological patterns.

Globally, the ASIR of breast cancer gradually increased, while the ASDR of breast cancer steadily decreased in the past 30 years. The two contradictory trends are partly attributed to the widely-adopted mammographic screening and improved treatment strategy [22]. A pervious study based on GBD 2017 reported a similar conclusion: steadily growing ASIR and declining ASDR [15]. From 2017 to 2019, China, the USA, and India were still the top 3 countries with the most breast cancer incidence and death. Moreover, Li et al. found that high body mass index, alcohol use, and high fasting plasma glucose were risk factors of breast cancer-caused mortality [15]. In parallel, a registry-based cohort study in Norway showed that alcohol use and tobacco smoking were associated with higher risk of breast cancer for women with benign breast diseases [23]. Up to now, it has been identified multiple modifiable risk factors of breast cancer including alcohol, obesity, younger age of menarche, older age of menopause, fewer childbearing, fewer breastfeeding, lower physical activity, and unhealthy lifestyles such as high fat consumption and low intakes of fruits, vegetables, and grains [24]. The alterations in reproductive patterns (such as low fertility rate), lifestyles, obesity rate, and age of menarche and menopause contribute to the increased incidence rate of breast cancer globally [25–28]. Besides, the breast cancer incidence rate varies in different ethnicities. It was reported that the incidence rate of white women was higher than that of ethnic minorities [29].

In the study, for cervical cancer, the age-standardized rates of incidence, death, and DALY were negatively correlated with SDI. Relatively, the incidence rate and death rate were significantly higher in these low-source countries. It has been confirmed that the carcinogenic HPV infection is the primary cause of cervical cancer [30]. In the past decades, due to the implementation of HPV vaccination and HPV-based screening, the incidence rate of cervical cancer kept declining [31]. Also, based on the microscopic inspection of cervical scraping smear, the treatment of pre-cancerous lesions is the secondary prevention of cervical cancer [31]. However, in some developing areas, such as Southern Sub-Saharan Africa, it is hard for most women to access effective interventions [32]. Therefore, it is urgent for these areas to implement essential prevention and population-based screening programs, including introducing vaccination, screening cervical cancer and precursor lesions. It is estimated that the implementation of WHO cervical cancer estimation strategy could significantly decrease cervical cancer mortality by 99 % in the following century [33].

Our results showed that the incidence rate of ovarian cancer varied geographically, positively related to SDI value. Fewer breastfeeding, infertility or nulliparity, hormone treatment, and obesity are risk factors of ovarian cancer [34–38]. Contrarily, oral contraceptive is a strong protective factor for ovarian cancer [39, 40]. Previous studies showed that oral contraceptives could inhibit carcinogenesis by interfering with estradiol production and decreasing estradiol exposure in the menstrual cycle [39]. The high ovarian cancer incidence rate in regions such as Western Europe and North America, might be related to the high prevalence of these risk factors. In some developed countries, including the USA, the declined incidence rate paralleled the decreased mortality rate in the past 30 years. The developments in ovarian cancer treatment, including targeted therapy, neoadjuvant chemotherapy, intraperitoneal chemotherapy, aggressive surgery, contribute to the declined mortality rate [41].

The incidence rate of uterine cancer was mushrooming globally, especially in high SDI region. According to GBD 2019, in the USA, the ASIR of uterine cancer increased from 19.63 to 1990 to 28.80 in 2019. Obesity was a vital risk factor contributing to the increased uterine cancer incidence [42]. Overweight or obese women are more likely to develop uterine cancer than normal-weight women [43]. Besides, decreased physical activity and a higher prevalence of diabetes are also the risk factors of uterine cancer [44]. Public health programs to help women maintain normal weight and increase physical activity could decrease uterine cancer risk.

Although population screening might be an effective measure to control cancer-caused mortality, the effectiveness of general population screening for women’s cancers needs further validation in the real world. A recent randomized controlled trial in UK showed that annual transvaginal ultrasound screening increased the incidence rate of Stage I/II ovarian and tubal cancer but decreased the incidence rate of Stage III/IV ovarian and tubal cancer, relative to non-screening [45]. Notably, there was no significant reduction in disease-specific death in annual transvaginal ultrasound screening group, indicating the general population screening for ovarian cancer might be unnecessary at present stage [45].

Cancer is an aging-related disease. In the four types of women’s cancers, we found the ASIR was positively correlated with age. Due to the demographic alterations in the future including the declining parity and the aging of baby boom generation, the mean age of some countries will further grow. It is estimated that in the USA, the incidence cases of ovarian cancer will increase by 37 %, from 20,921 cases in 2010 to 28,591 cases in 2030 [46]. This rise in ovarian cancer cases is mainly attributed to alterations in age-distribution and population growth [46]. Given the aging trend, the burden of women’s cancers might continue to increase in the following decades.

## Conclusions

Breast cancer was the most common women’s cancer worldwide, which was followed by cervical cancer in the low SDI region. Relatively, ovarian cancer and uterine cancer were more common in high SDI region. Women’s cancers cause numerous mortalities worldwide and place heavy economic burdens on women and their families. In the background of global aging, international efforts are needed to reduce women’s cancer incidence and mortality and improve women’s health.

## Supplementary Information

**Additional file 1: Figure S1:** The age standardized rates of breast cancer in 204 countries or territories in 2019. (a) The ASIR of breast cancer in 204 countries or territories; (b) The ASDR of breast cancer in 204 countries or territories; (c) The age-standardized DALY rate of breast cancer in 204 countries or territories. Note: ASIR, Age-standardized incidence rate; ASDR, Age-standardized death rate; DALY, disability adjusted life year.

**Additional file 2: Figure S2:** The age standardized rates of cervical cancer in 204 countries or territories in 2019. (a) The ASIR of cervical cancer in 204 countries or territories; (b) The ASDR of cervical cancer in 204 countries or territories; (c) The age-standardized DALY rate of cervical cancer in 204 countries or territories. Note: ASIR, Age-standardized incidence rate; ASDR, Age-standardized death rate; DALY, disability adjusted life year.

**Additional file 3: Figure S3:** The age standardized rates of ovarian cancer in 204 countries or territories in 2019. (a) The ASIR of ovarian cancer in 204 countries or territories; (b) The ASDR of ovarian cancer in 204 countries or territories; (c) The age-standardized DALY rate of ovarian cancer in 204 countries or territories. Note: ASIR, Age-standardized incidence rate; ASDR, Age-standardized death rate; DALY, disability adjusted life year.

**Additional file 4: Figure S4:** The age standardized rates of uterine cancer in 204 countries or territories in 2019. (a) The ASIR of uterine cancer in 204 countries or territories; (b) The ASDR of uterine cancer in 204 countries or territories; (c) The age-standardized DALY rate of uterine cancer in 204 countries or territories. Note: ASIR, Age-standardized incidence rate; ASDR, Age-standardized death rate; DALY, disability adjusted life year.

**Additional file 5: Figure S5:** The incidence, death, DALY, and corresponding age-standardized rates of cancers in different age groups in 2019. (a) The incidence, death, and DALY of cancers in different age groups. (b) The ASIR, ASDR, and age-standardized DALY rate of cancers in different age groups. Note: ASIR, Age-standardized incidence rate; ASDR, Age-standardized death rate; DALY, disability adjusted life year.

**Additional file 6: Figure S6:** The incidence, death, and DALY of cancers in different age groups in high SDI region in 2019. Note: DALY, disability adjusted life year.

**Additional file 7: Figure S7:** The incidence, death, and DALY of cancers in different age groups in high-middle SDI region in 2019. Note: DALY, disability adjusted life year.

**Additional file 8: Figure S8:** The incidence, death, and DALY of cancers in different age groups in middle SDI region in 2019. Note: DALY, disability adjusted life year.

**Additional file 9: Figure S9:** The incidence, death, and DALY of cancers in different age groups in low-middle SDI region in 2019. Note: DALY, disability adjusted life year.

**Additional file 10: Figure S10:** The incidence, death, and DALY of cancers in different age groups in low SDI region in 2019. Note: DALY, disability adjusted life year.

**Additional file 11: Figure S11:** The ASIR, ASDR, and age-standardized DALY rate of cancers in different age groups in high SDI region in 2019. Note: ASIR, Age-standardized incidence rate; ASDR, Age-standardized death rate; DALY, disability adjusted life year.

**Additional file 12: Figure S12:** The ASIR, ASDR, and age-standardized DALY rate of cancers in different age groups in high-middle SDI region in 2019. Note: ASIR, Age-standardized incidence rate; ASDR, Age-standardized death rate; DALY, disability adjusted life year.

**Additional file 13: Figure S13:** The ASIR, ASDR, and age-standardized DALY rate of cancers in different age groups in middle SDI region in 2019. Note: ASIR, Age-standardized incidence rate; ASDR, Age-standardized death rate; DALY, disability adjusted life year.

**Additional file 14: Figure S14:** The ASIR, ASDR, and age-standardized DALY rate of cancers in different age groups in low-middle SDI region in 2019. Note: ASIR, Age-standardized incidence rate; ASDR, Age-standardized death rate; DALY, disability adjusted life year.

**Additional file 15: Figure S15:** The ASIR, ASDR, and age-standardized DALY rate of cancers in different age groups in low SDI region in 2019. Note: ASIR, Age-standardized incidence rate; ASDR, Age-standardized death rate; DALY, disability adjusted life year.

**Additional file 16: Table S1:** The incidence of female breast cancer and temporal trends.

**Additional file 17: Table S2:** The death of female breast cancer and temporal trends.

**Additional file 18: Table S3:** The Disability-Adjusted Life Year (DALY) of female breast cancer and temporal trends.

**Additional file 19: Table S4:** The incidence of female cervical cancer and temporal trends.

**Additional file 20: Table S5:** The death of female cervical cancer and temporal trends.

**Additional file 21: Table S6:** The Disability-Adjusted Life Year (DALY) of female cervical cancer and temporal trends.

**Additional file 22:Table S7:** The incidence of female ovarian cancer and temporal trends.

**Additional file 23: Table S8:** The death of female ovarian cancer and temporal trends.

**Additional file 24: Table S9:** The Disability-Adjusted Life Year (DALY) of female ovarian cancer and temporal trends.

**Additional file 25: Table S10:** The incidence of uterine cancer and temporal trends.

**Additional file 26: Table S11:** The death of uterine cancer and temporal trends.

**Additional file 27: Table S12:** The Disability-Adjusted Life Year (DALY) of uterine cancer and temporal trends.

**Additional file 28: Table S13:** The age-standardized rate of women's cancers in 204 countries or territories.

## Data Availability

All data in this study is available from http://ghdx.healthdata.org/gbd-results-tool.

## Abbreviations

GBD: Global burden disease
DALY: Disability adjusted life year
SDI: Socio-demographic Index
ASIR: Age-standardized incidence rate
ASDR: Age-standardized death rate
EAPC: Estimated annual percentage change

## References

[1] Sung H, Ferlay J, Siegel RL, Laversanne M, Soerjomataram I, Jemal A (2021). Global cancer statistics 2020: GLOBOCAN estimates of incidence and mortality worldwide for 36 cancers in 185 countries. CA Cancer J Clin.

[2] Ginsburg O, Bray F, Coleman MP, Vanderpuye V, Eniu A, Kotha SR (2017). The global burden of women’s cancers: a grand challenge in global health. Lancet.

[3] Cabasag CJ, Arnold M, Butler J, Inoue M, Trabert B, Webb PM (2020). The influence of birth cohort and calendar period on global trends in ovarian cancer incidence. Int J Cancer.

[4] Zheng L, Cui C, Shi O, Lu X, Li YK, Wang W (2020). Incidence and mortality of ovarian cancer at the global, regional, and national levels, 1990–2017. Gynecol Oncol.

[5] Fitzmaurice C, Abate D, Abbasi N, Abbastabar H, Abd-Allah F, Abdel-Rahman O (2019). Global, Regional, and National Cancer Incidence, Mortality, Years of Life Lost, Years Lived With Disability, and Disability-Adjusted Life-Years for 29 Cancer Groups, 1990 to 2017: A Systematic Analysis for the Global Burden of Disease Study. JAMA Oncol.

[6] Global burden (2020). of 87 risk factors in 204 countries and territories, 1990–2019: a systematic analysis for the Global Burden of Disease Study 2019. Lancet.

[7] Ning L, Hu C, Lu P, Que Y, Zhu X, Li D (2020). Trends in disease burden of chronic myeloid leukemia at the global, regional, and national levels: a population-based epidemiologic study. Exp Hematol Oncol.

[8] Keykhaei M, Masinaei M, Mohammadi E, Azadnajafabad S, Rezaei N, Saeedi Moghaddam S (2021). A global, regional, and national survey on burden and Quality of Care Index (QCI) of hematologic malignancies; global burden of disease systematic analysis 1990–2017. Exp Hematol Oncol.

[9] Yi M, Li A, Zhou L, Chu Q, Song Y, Wu K (2020). The global burden and attributable risk factor analysis of acute myeloid leukemia in 195 countries and territories from 1990 to 2017: estimates based on the global burden of disease study 2017. J Hematol Oncol.

[10] Deng Y, Li H, Wang M, Li N, Tian T, Wu Y (2020). Global Burden of Thyroid Cancer From 1990 to 2017. JAMA Netw Open.

[11] Bai X, Yi M, Dong B, Zheng X, Wu K (2020). The global, regional, and national burden of kidney cancer and attributable risk factor analysis from 1990 to 2017. Exp Hematol Oncol.

[12] Yi M, Zhou L, Li A, Luo S, Wu K (2020). Global burden and trend of acute lymphoblastic leukemia from 1990 to 2017. Aging.

[13] Lin L, Yan L, Liu Y, Yuan F, Li H, Ni J (2019). Incidence and death in 29 cancer groups in 2017 and trend analysis from 1990 to 2017 from the Global Burden of Disease Study. J Hematol Oncol.

[14] Liu W, Liu J, Song Y, Zeng X, Wang X, Mi L (2019). Burden of lymphoma in China, 2006–2016: an analysis of the Global Burden of Disease Study 2016. J Hematol Oncol.

[15] Li N, Deng Y, Zhou L, Tian T, Yang S, Wu Y (2019). Global burden of breast cancer and attributable risk factors in 195 countries and territories, from 1990 to 2017: results from the Global Burden of Disease Study 2017. J Hematol Oncol.

[16] Stevens GA, Alkema L, Black RE, Boerma JT, Collins GS, Ezzati M (2016). Guidelines for Accurate and Transparent Health Estimates Reporting: the GATHER statement. Lancet.

[17] Zhai Z, Zheng Y, Li N, Deng Y, Zhou L, Tian T (2020). Incidence and disease burden of prostate cancer from 1990 to 2017: Results from the Global Burden of Disease Study 2017. Cancer.

[18] Deng Y, Wang M, Zhou L, Zheng Y, Li N, Tian T (2020). Global burden of larynx cancer, 1990–2017: estimates from the global burden of disease 2017 study. Aging.

[19] Deng Y, Zhao P, Zhou L, Xiang D, Hu J, Liu Y (2020). Epidemiological trends of tracheal, bronchus, and lung cancer at the global, regional, and national levels: a population-based study. J Hematol Oncol.

[20] Zhou L, Deng Y, Li N, Zheng Y, Tian T, Zhai Z (2019). Global, regional, and national burden of Hodgkin lymphoma from 1990 to 2017: estimates from the 2017 Global Burden of Disease study. J Hematol Oncol.

[21] Yang S, Lin S, Li N, Deng Y, Wang M, Xiang D (2020). Burden, trends, and risk factors of esophageal cancer in China from 1990 to 2017: an up-to-date overview and comparison with those in Japan and South Korea. J Hematol Oncol.

[22] Jatoi I, Miller AB (2003). Why is breast-cancer mortality declining?. Lancet Oncol.

[23] Lilleborge M, Falk RS, Sørlie T, Ursin G, Hofvind S (2021). Can breast cancer be stopped? Modifiable risk factors of breast cancer among women with a prior benign or premalignant lesion. Int J Cancer.

[24] Britt KL, Cuzick J, Phillips KA (2020). Key steps for effective breast cancer prevention. Nat Rev Cancer.

[25] Khalis M, Charbotel B, Chajès V, Rinaldi S, Moskal A, Biessy C (2018). Menstrual and reproductive factors and risk of breast cancer: A case-control study in the Fez region, Morocco. PLoS One.

[26] Chlebowski RT, Aragaki AK, Anderson GL, Pan K, Neuhouser ML, Manson JE (2020). Dietary Modification and Breast Cancer Mortality: Long-Term Follow-Up of the Women’s Health Initiative Randomized Trial. J Clin Oncol.

[27] Picon-Ruiz M, Morata-Tarifa C, Valle-Goffin JJ, Friedman ER, Slingerland JM (2017). Obesity and adverse breast cancer risk and outcome: Mechanistic insights and strategies for intervention. CA Cancer J Clin.

[28] Menarche menopause. and breast cancer risk: individual participant meta-analysis, including 118 964 women with breast cancer from 117 epidemiological studies. Lancet Oncol. 2012;13:1141–51. 10.1016/S1470-2045(12)70425-4 PMC3488186 23084519

[29] Chlebowski RT, Chen Z, Anderson GL, Rohan T, Aragaki A, Lane D (2005). Ethnicity and breast cancer: factors influencing differences in incidence and outcome. J Natl Cancer Inst.

[30] Lei J, Ploner A, Elfström KM, Wang J, Roth A, Fang F (2020). HPV Vaccination and the Risk of Invasive Cervical Cancer. N Engl J Med.

[31] Arbyn M, Weiderpass E, Bruni L, de Sanjosé S, Saraiya M, Ferlay J (2020). Estimates of incidence and mortality of cervical cancer in 2018: a worldwide analysis. Lancet Glob Health.

[32] Parkin DM, Hämmerl L, Ferlay J, Kantelhardt EJ (2020). Cancer in Africa 2018: The role of infections. Int J Cancer.

[33] Canfell K, Kim JJ, Brisson M, Keane A, Simms KT, Caruana M (2020). Mortality impact of achieving WHO cervical cancer elimination targets: a comparative modelling analysis in 78 low-income and lower-middle-income countries. Lancet.

[34] Babic A, Sasamoto N, Rosner BA, Tworoger SS, Jordan SJ, Risch HA (2020). Association Between Breastfeeding and Ovarian Cancer Risk. JAMA Oncol.

[35] Jiang YT, Gong TT, Zhang JY, Li XQ, Gao S, Zhao YH (2020). Infertility and ovarian cancer risk: Evidence from nine prospective cohort studies. Int J Cancer.

[36] Olsen CM, Green AC, Whiteman DC, Sadeghi S, Kolahdooz F, Webb PM (2007). Obesity and the risk of epithelial ovarian cancer: a systematic review and meta-analysis. Eur J Cancer.

[37] Coburn SB, Bray F, Sherman ME, Trabert B (2017). International patterns and trends in ovarian cancer incidence, overall and by histologic subtype. Int J Cancer.

[38] Tanday S (2015). Hormone therapy increases ovarian cancer risk. Lancet Oncol.

[39] Michels KA, Pfeiffer RM, Brinton LA, Trabert B (2018). Modification of the Associations Between Duration of Oral Contraceptive Use and Ovarian, Endometrial, Breast, and Colorectal Cancers. JAMA Oncol.

[40] Moorman PG, Havrilesky LJ, Gierisch JM, Coeytaux RR, Lowery WJ, Peragallo Urrutia R (2013). Oral contraceptives and risk of ovarian cancer and breast cancer among high-risk women: a systematic review and meta-analysis. J Clin Oncol.

[41] Sopik V, Iqbal J, Rosen B, Narod SA (2015). Why have ovarian cancer mortality rates declined? Part II. Case-fatality. Gynecol Oncol.

[42] Steele CB, Thomas CC, Henley SJ, Massetti GM, Galuska DA, Agurs-Collins T (2017). Vital Signs: Trends in Incidence of Cancers Associated with Overweight and Obesity - United States, 2005–2014. MMWR Morb Mortal Wkly Rep.

[43] Henley SJ, Miller JW, Dowling NF, Benard VB, Richardson LC (2018). Uterine Cancer Incidence and Mortality - United States, 1999–2016. MMWR Morb Mortal Wkly Rep.

[44] Cote ML, Ruterbusch JJ, Olson SH, Lu K, Ali-Fehmi R (2015). The Growing Burden of Endometrial Cancer: A Major Racial Disparity Affecting Black Women. Cancer Epidemiol Biomarkers Prev.

[45] Menon U, Gentry-Maharaj A, Burnell M, Singh N, Ryan A, Karpinskyj C (2021). Ovarian cancer population screening and mortality after long-term follow-up in the UK Collaborative Trial of Ovarian Cancer Screening (UKCTOCS): a randomised controlled trial. Lancet.

[46] Sopik V, Iqbal J, Rosen B, Narod SA (2015). Why have ovarian cancer mortality rates declined? Part I. Incidence. Gynecol Oncol.

