# Supplementary figures and images for: Epidemiological trends of women’s cancers from 1990 to 2019 at the global, regional, and national levels: a population-based study
Source: Biomark Res. 2021 Jul 7;9:55. doi: 10.1186/s40364-021-00310-y (PMC8261911; doi:10.1186/s40364-021-00310-y)

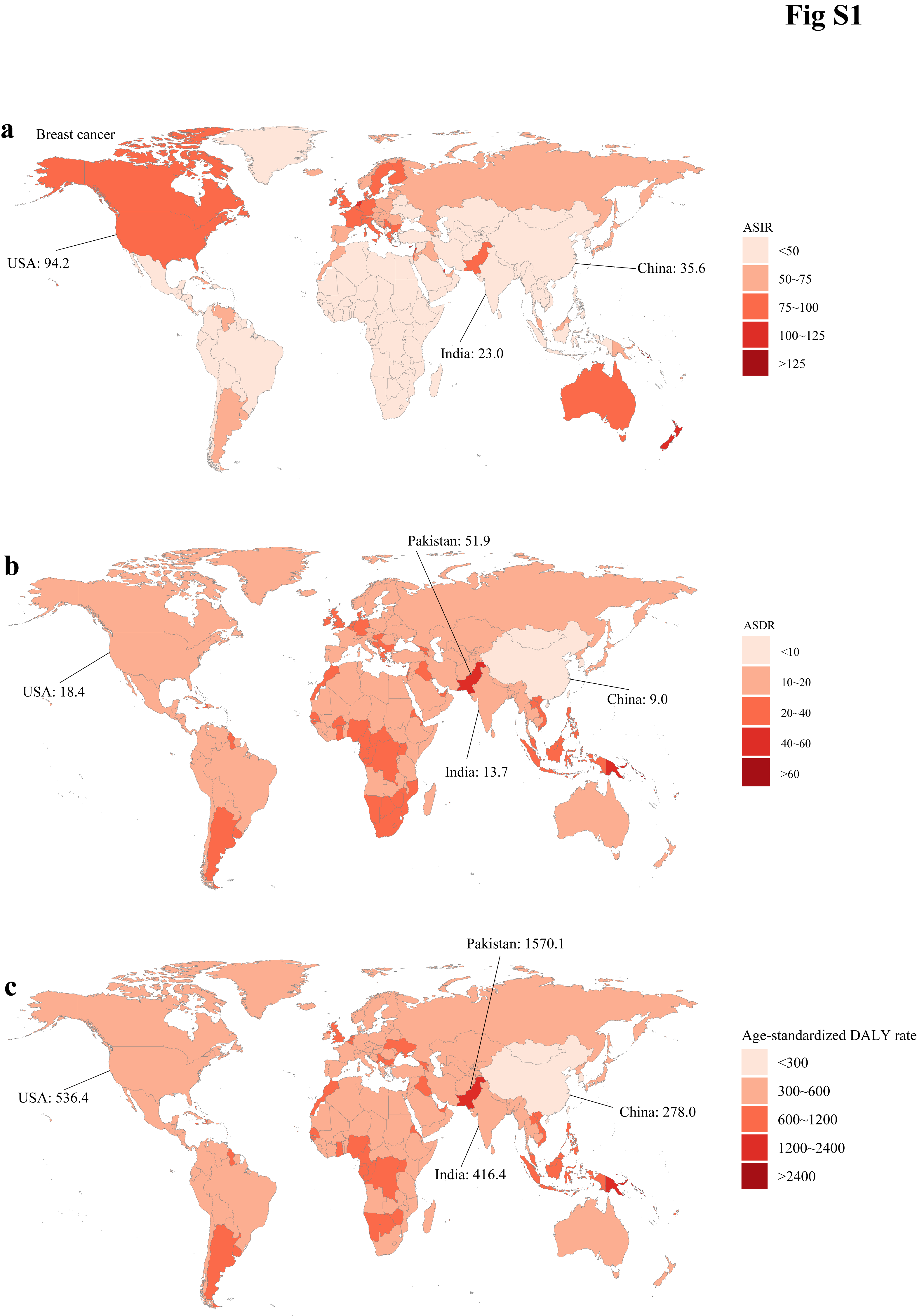

Supplement: Supplementary file 1 — Additional file 1: Figure S1: The age standardized rates of breast cancer in 204 countries or territories in 2019. (a) The ASIR of breast cancer in 204 countries or territories; (b) The ASDR of breast cancer in 204 countries or territories; (c) The age-standardized DALY rate of breast cancer in 204 countries or territories. Note: ASIR, Age-standardized incidence rate; ASDR, Age-standardized death rate; DALY, disability adjusted life year. [file 40364_2021_310_MOESM1_ESM.tif]

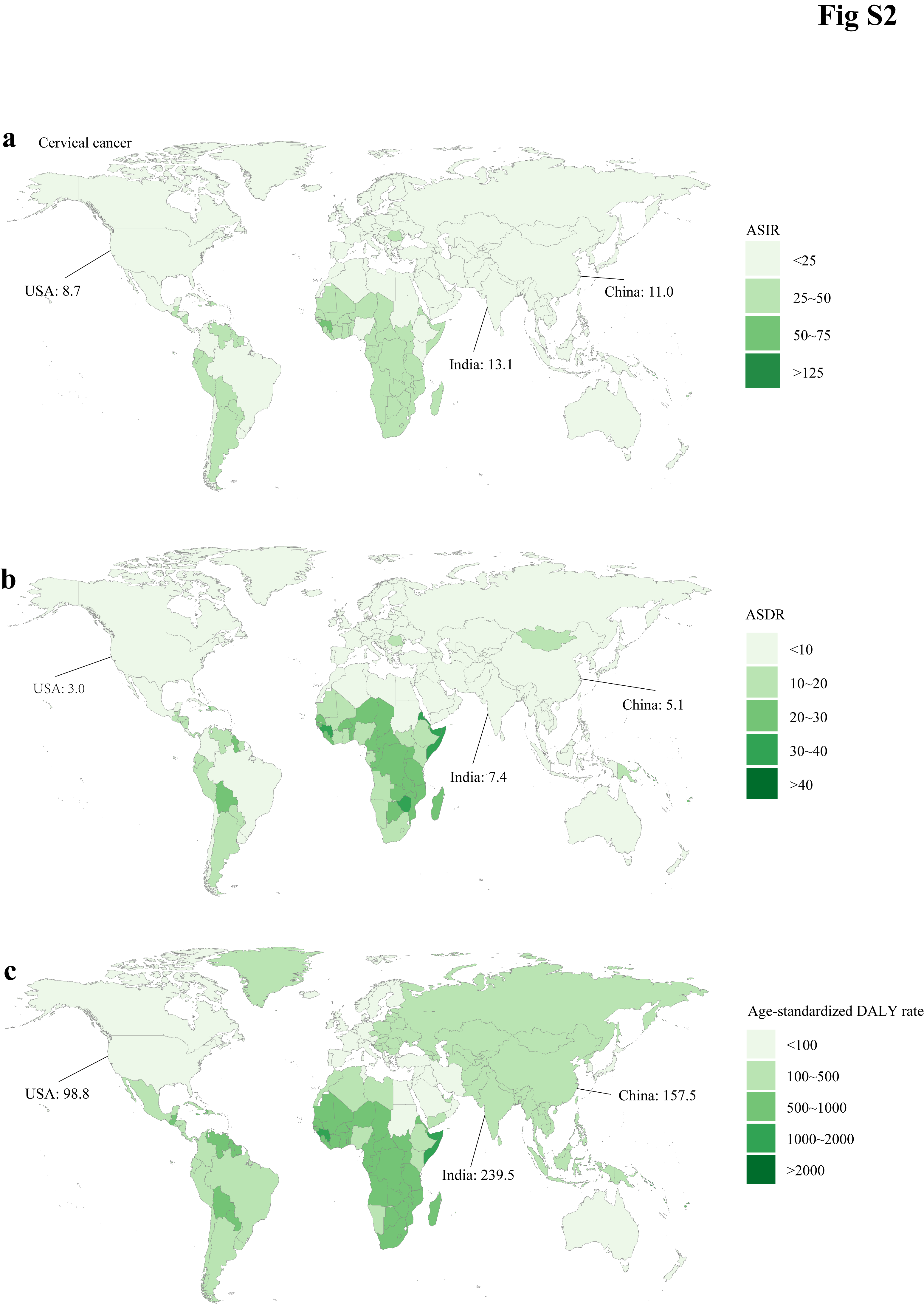

Supplement: Supplementary file 2 — Additional file 2: Figure S2: The age standardized rates of cervical cancer in 204 countries or territories in 2019. (a) The ASIR of cervical cancer in 204 countries or territories; (b) The ASDR of cervical cancer in 204 countries or territories; (c) The age-standardized DALY rate of cervical cancer in 204 countries or territories. Note: ASIR, Age-standardized incidence rate; ASDR, Age-standardized death rate; DALY, disability adjusted life year. [file 40364_2021_310_MOESM2_ESM.tif]

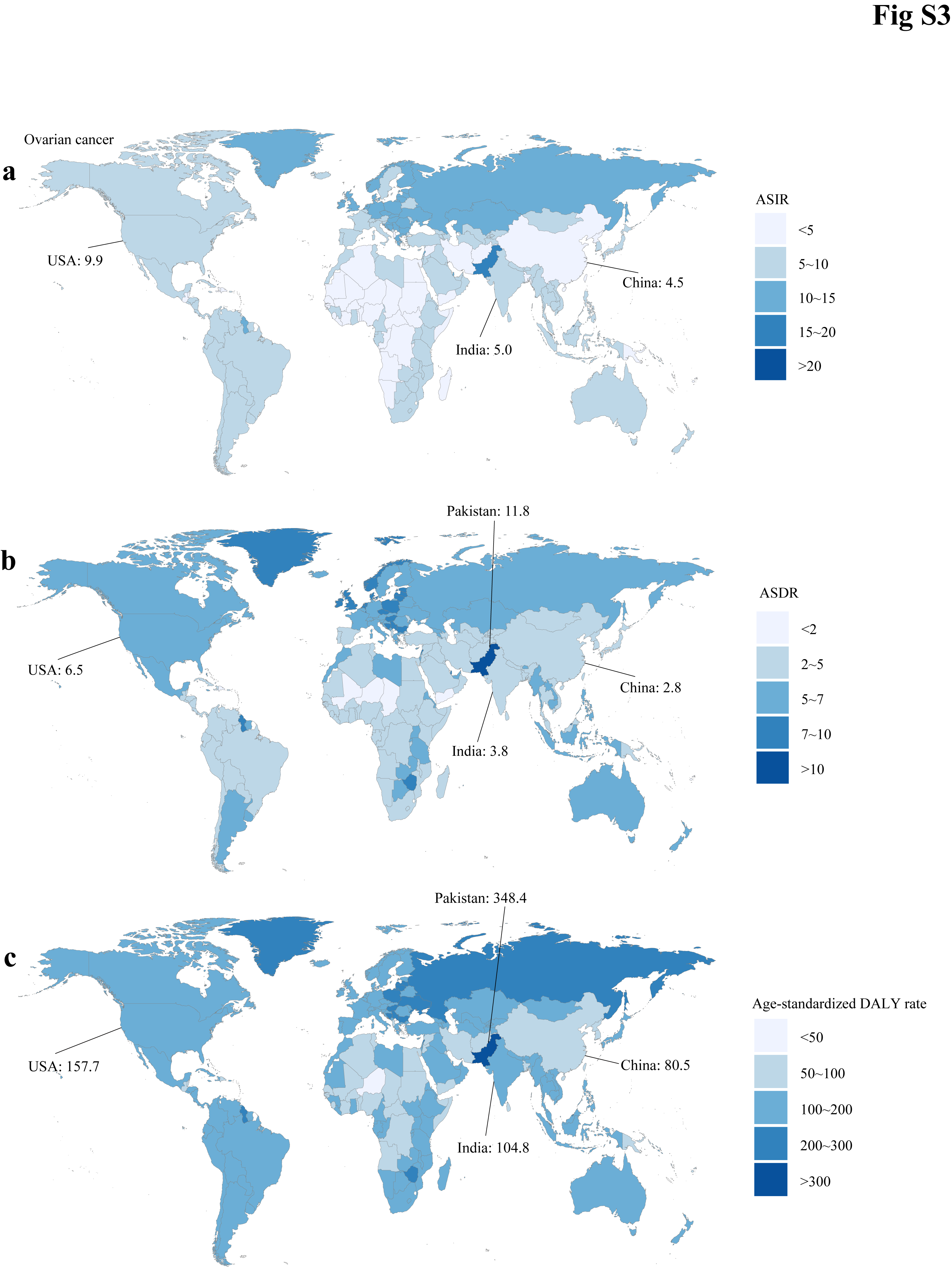

Supplement: Supplementary file 3 — Additional file 3: Figure S3: The age standardized rates of ovarian cancer in 204 countries or territories in 2019. (a) The ASIR of ovarian cancer in 204 countries or territories; (b) The ASDR of ovarian cancer in 204 countries or territories; (c) The age-standardized DALY rate of ovarian cancer in 204 countries or territories. Note: ASIR, Age-standardized incidence rate; ASDR, Age-standardized death rate; DALY, disability adjusted life year. [file 40364_2021_310_MOESM3_ESM.tif]

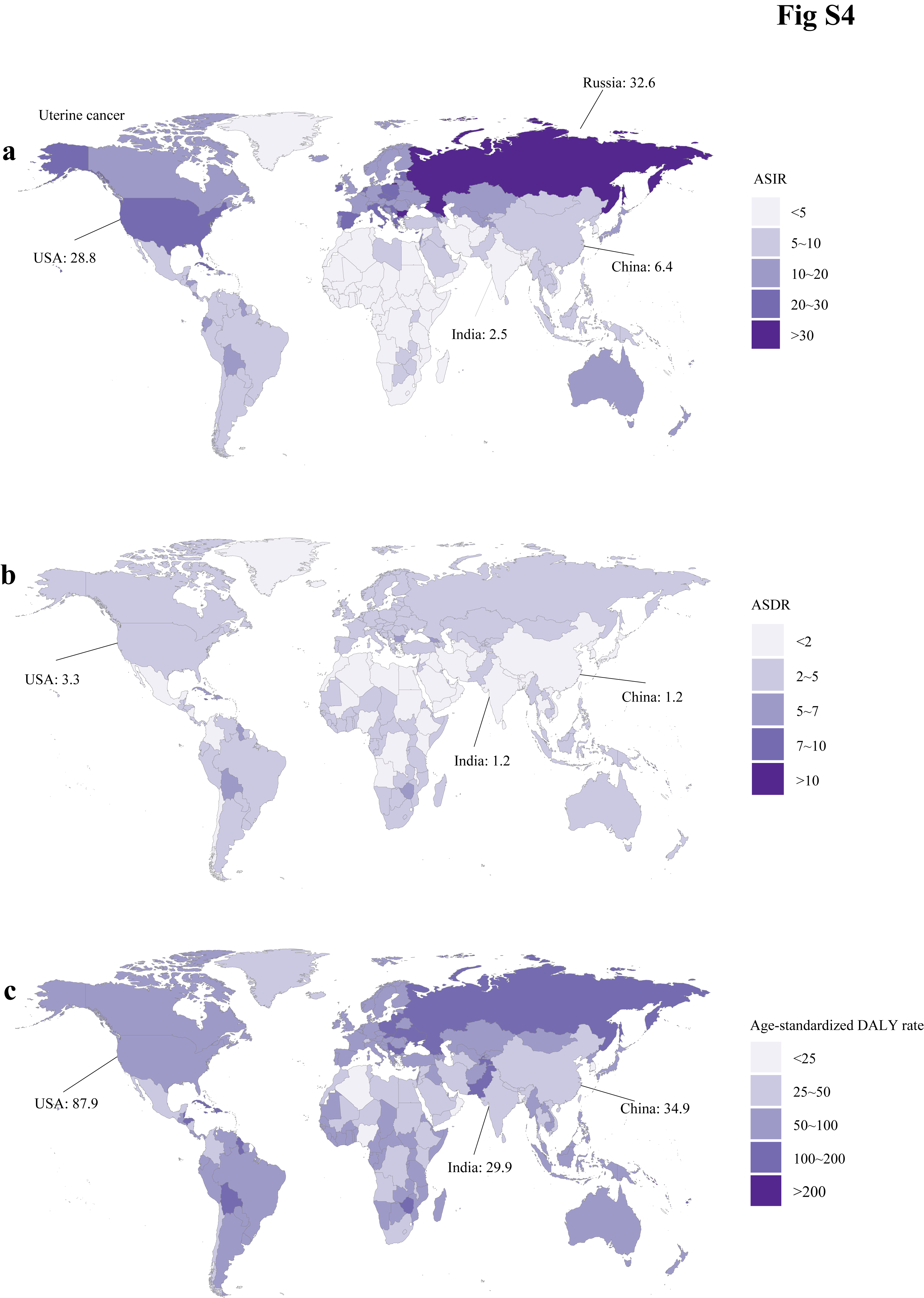

Supplement: Supplementary file 4 — Additional file 4: Figure S4: The age standardized rates of uterine cancer in 204 countries or territories in 2019. (a) The ASIR of uterine cancer in 204 countries or territories; (b) The ASDR of uterine cancer in 204 countries or territories; (c) The age-standardized DALY rate of uterine cancer in 204 countries or territories. Note: ASIR, Age-standardized incidence rate; ASDR, Age-standardized death rate; DALY, disability adjusted life year. [file 40364_2021_310_MOESM4_ESM.tif]

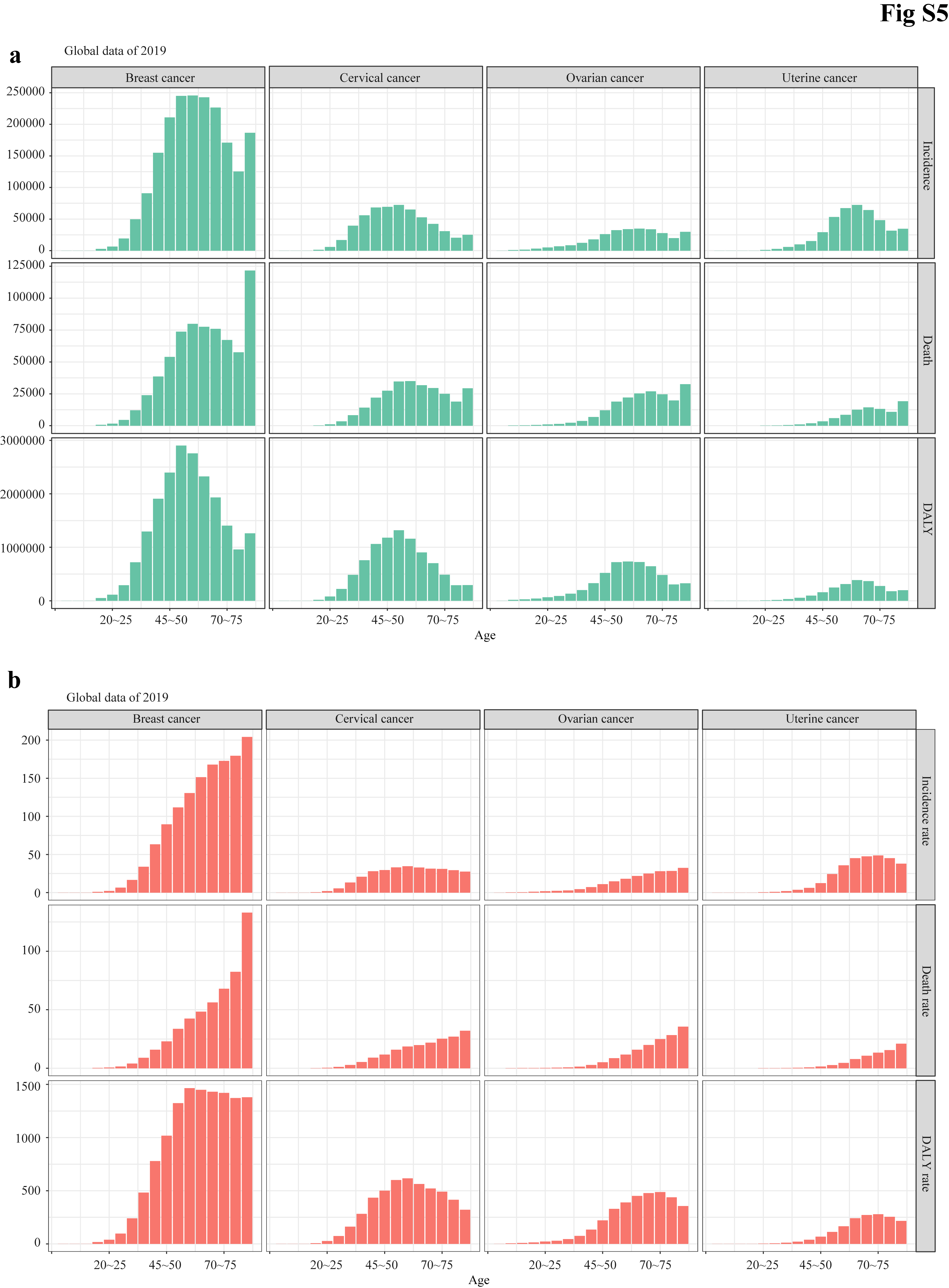

Supplement: Supplementary file 5 — Additional file 5: Figure S5: The incidence, death, DALY, and corresponding age-standardized rates of cancers in different age groups in 2019. (a) The incidence, death, and DALY of cancers in different age groups. (b) The ASIR, ASDR, and age-standardized DALY rate of cancers in different age groups. Note: ASIR, Age-standardized incidence rate; ASDR, Age-standardized death rate; DALY, disability adjusted life year. [file 40364_2021_310_MOESM5_ESM.tif]

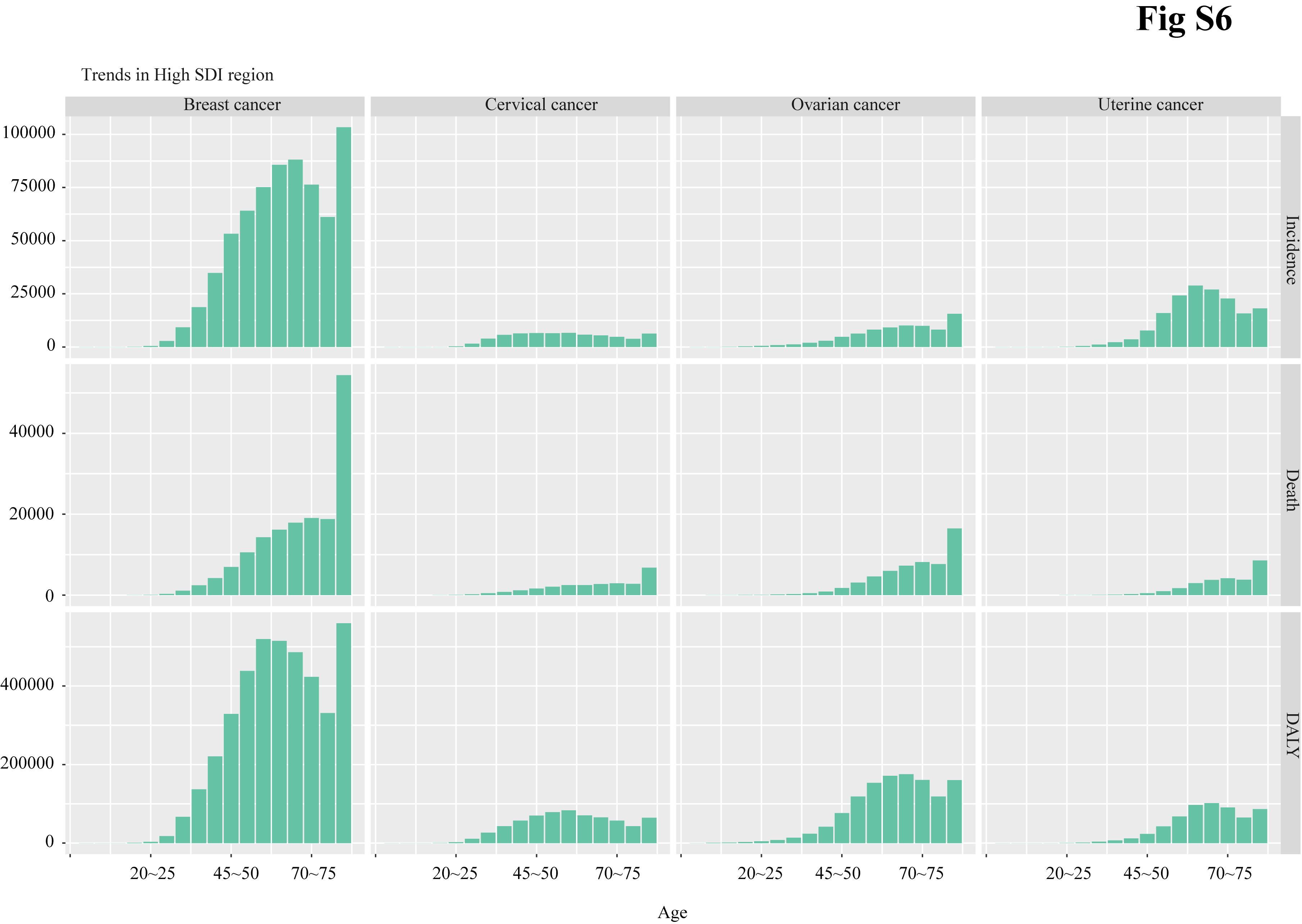

Supplement: Supplementary file 6 — Additional file 6: Figure S6: The incidence, death, and DALY of cancers in different age groups in high SDI region in 2019. Note: DALY, disability adjusted life year. [file 40364_2021_310_MOESM6_ESM.tif]

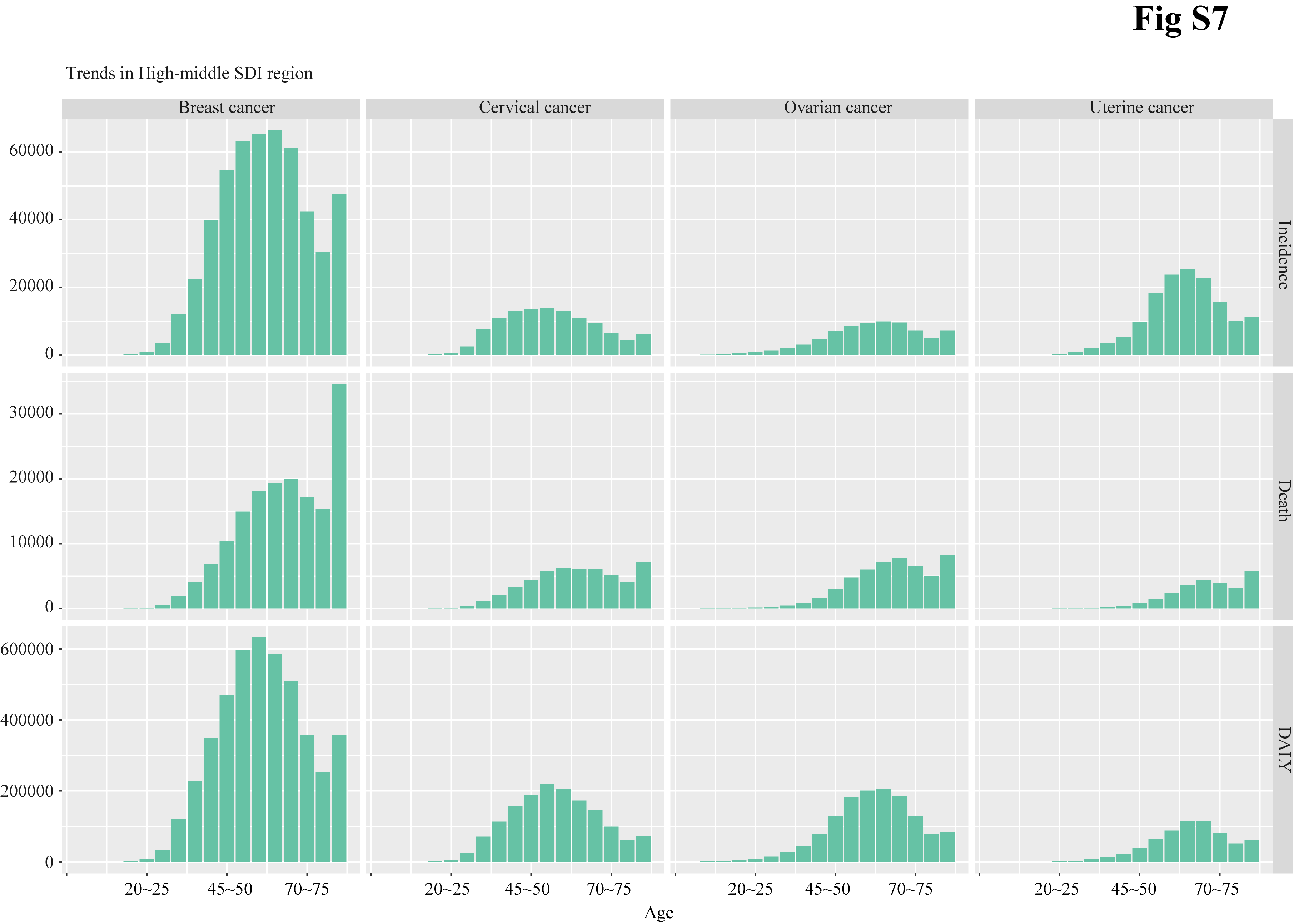

Supplement: Supplementary file 7 — Additional file 7: Figure S7: The incidence, death, and DALY of cancers in different age groups in high-middle SDI region in 2019. Note: DALY, disability adjusted life year. [file 40364_2021_310_MOESM7_ESM.tif]

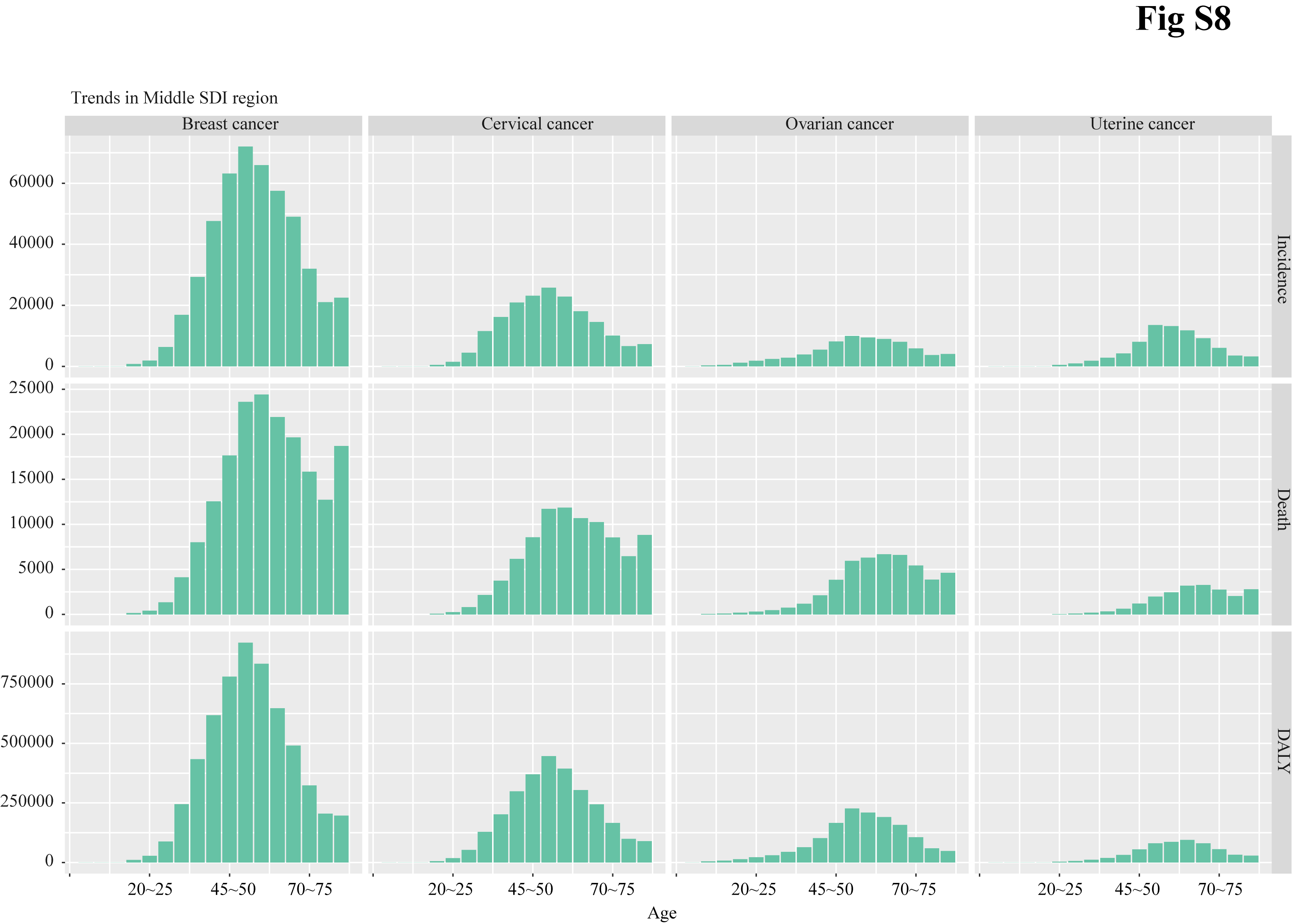

Supplement: Supplementary file 8 — Additional file 8: Figure S8: The incidence, death, and DALY of cancers in different age groups in middle SDI region in 2019. Note: DALY, disability adjusted life year. [file 40364_2021_310_MOESM8_ESM.tif]

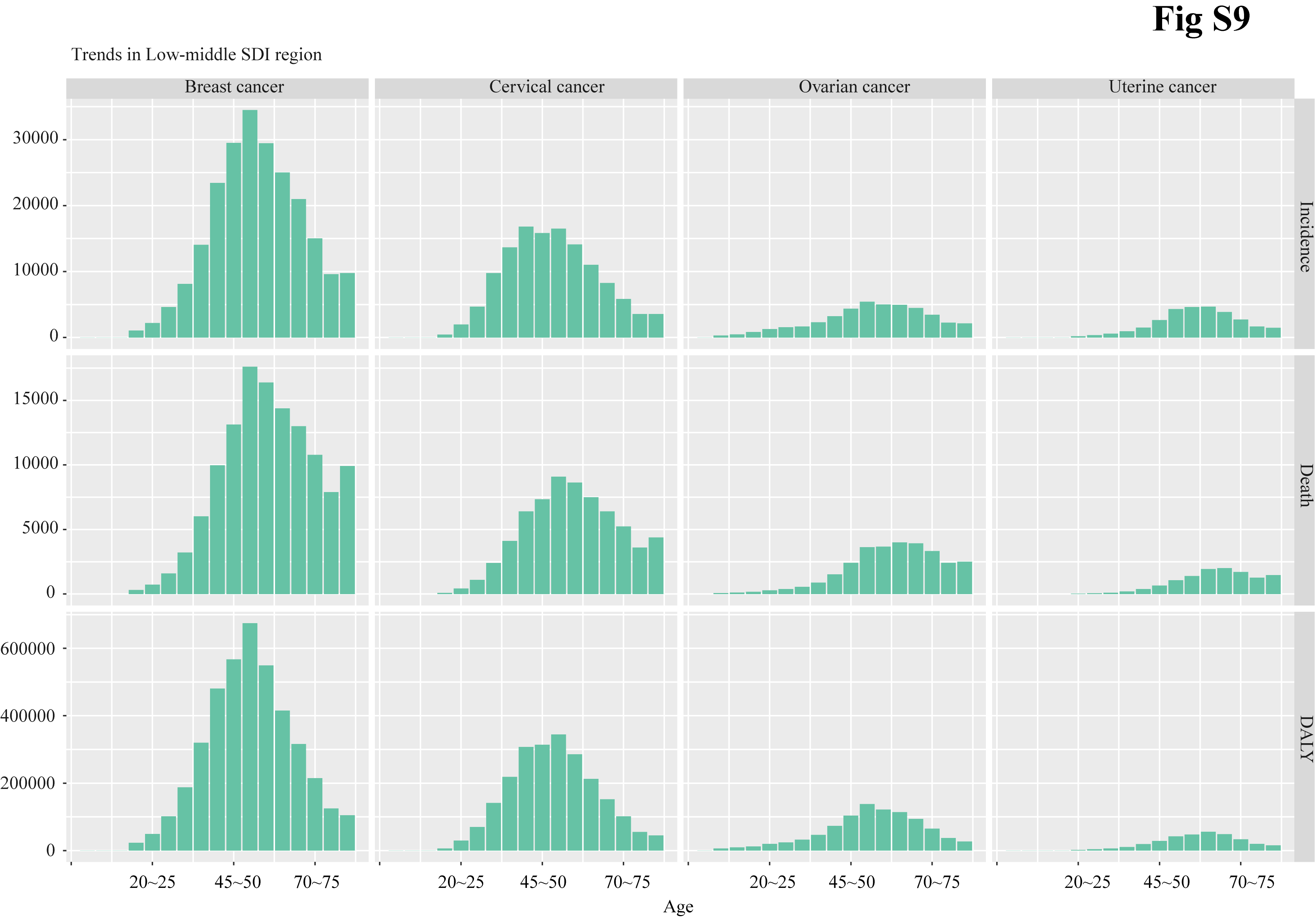

Supplement: Supplementary file 9 — Additional file 9: Figure S9: The incidence, death, and DALY of cancers in different age groups in low-middle SDI region in 2019. Note: DALY, disability adjusted life year. [file 40364_2021_310_MOESM9_ESM.tif]

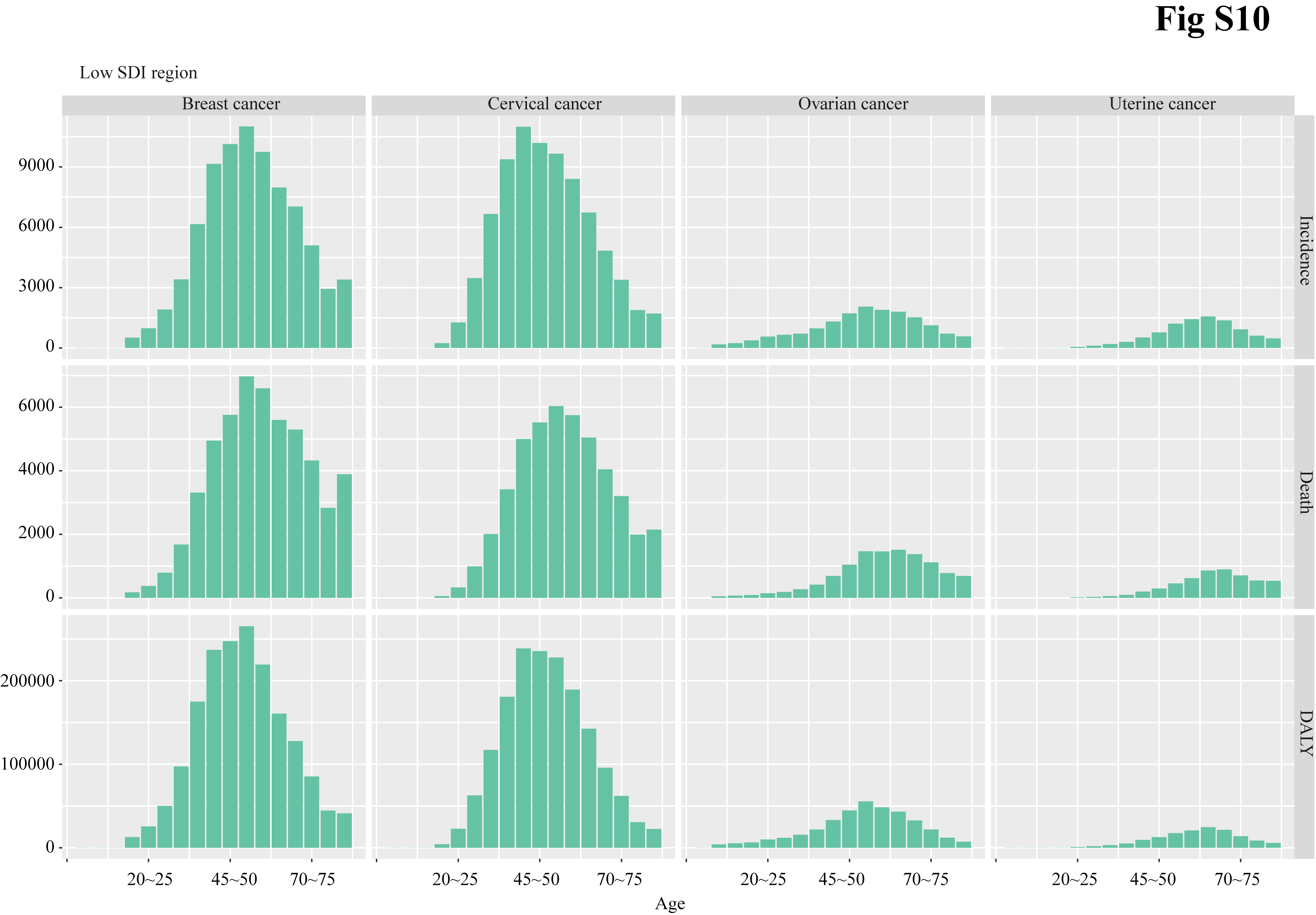

Supplement: Supplementary file 10 — Additional file 10: Figure S10: The incidence, death, and DALY of cancers in different age groups in low SDI region in 2019. Note: DALY, disability adjusted life year. [file 40364_2021_310_MOESM10_ESM.tif]

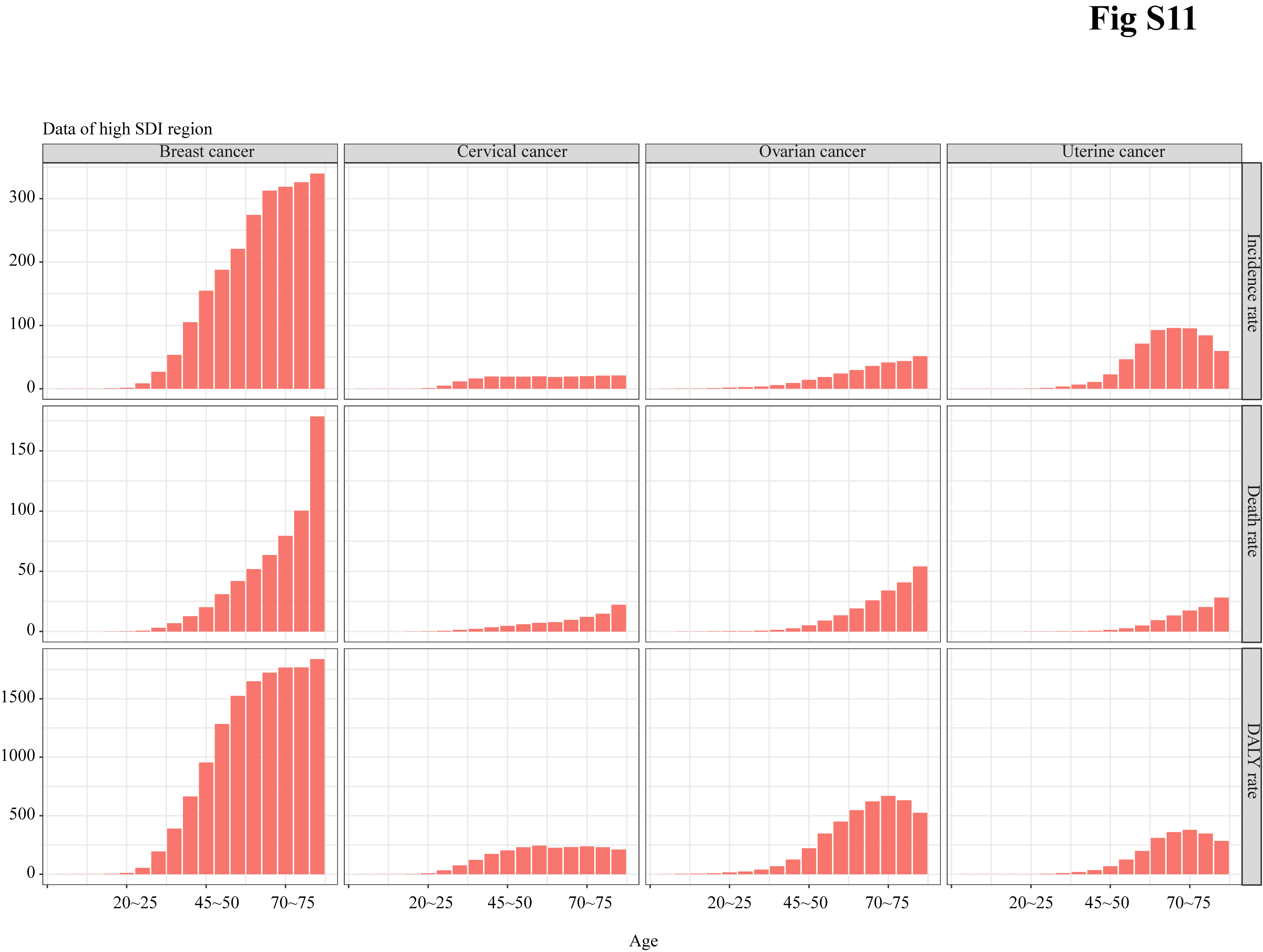

Supplement: Supplementary file 11 — Additional file 11: Figure S11: The ASIR, ASDR, and age-standardized DALY rate of cancers in different age groups in high SDI region in 2019. Note: ASIR, Age-standardized incidence rate; ASDR, Age-standardized death rate; DALY, disability adjusted life year. [file 40364_2021_310_MOESM11_ESM.tif]

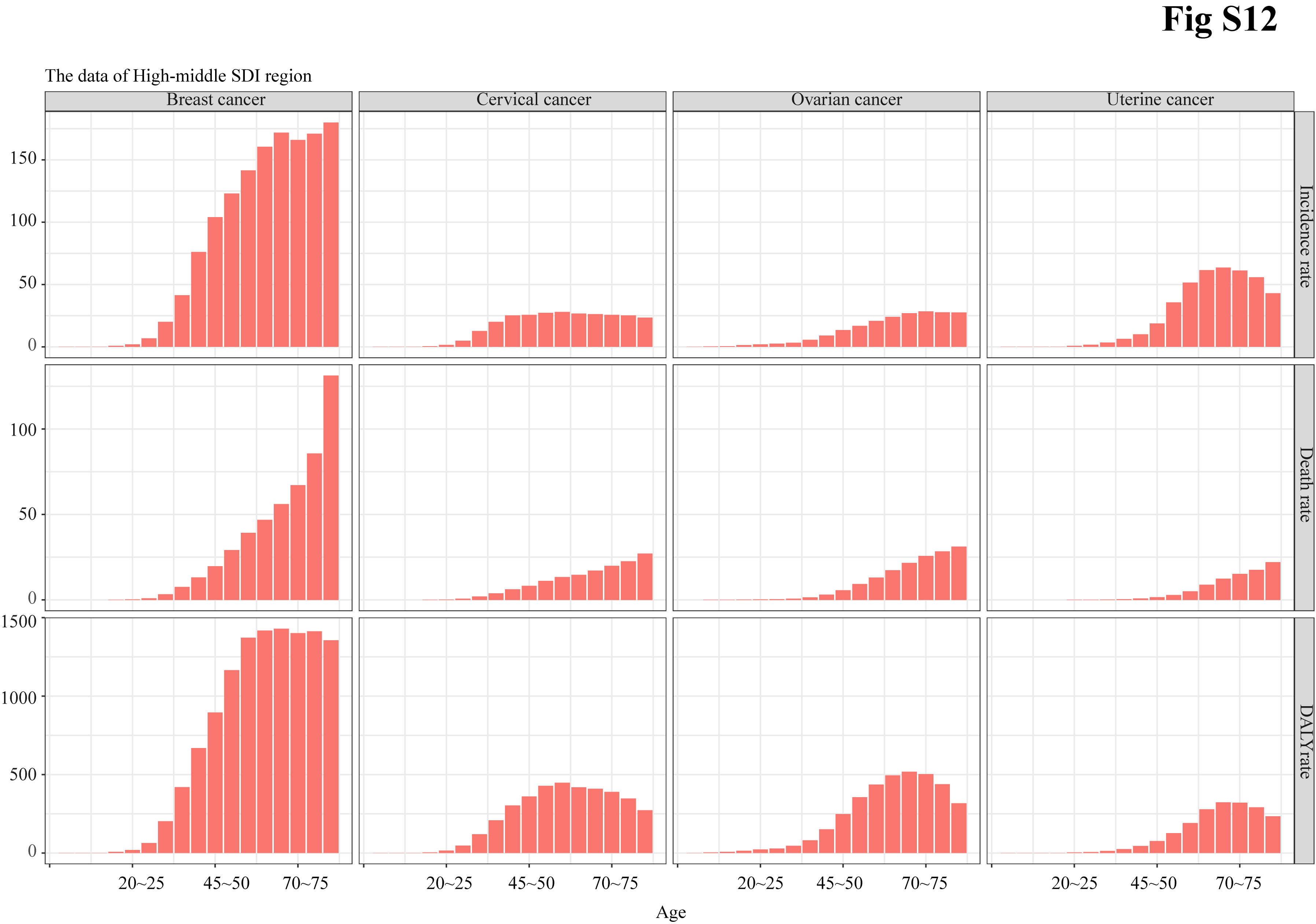

Supplement: Supplementary file 12 — Additional file 12: Figure S12: The ASIR, ASDR, and age-standardized DALY rate of cancers in different age groups in high-middle SDI region in 2019. Note: ASIR, Age-standardized incidence rate; ASDR, Age-standardized death rate; DALY, disability adjusted life year. [file 40364_2021_310_MOESM12_ESM.tif]

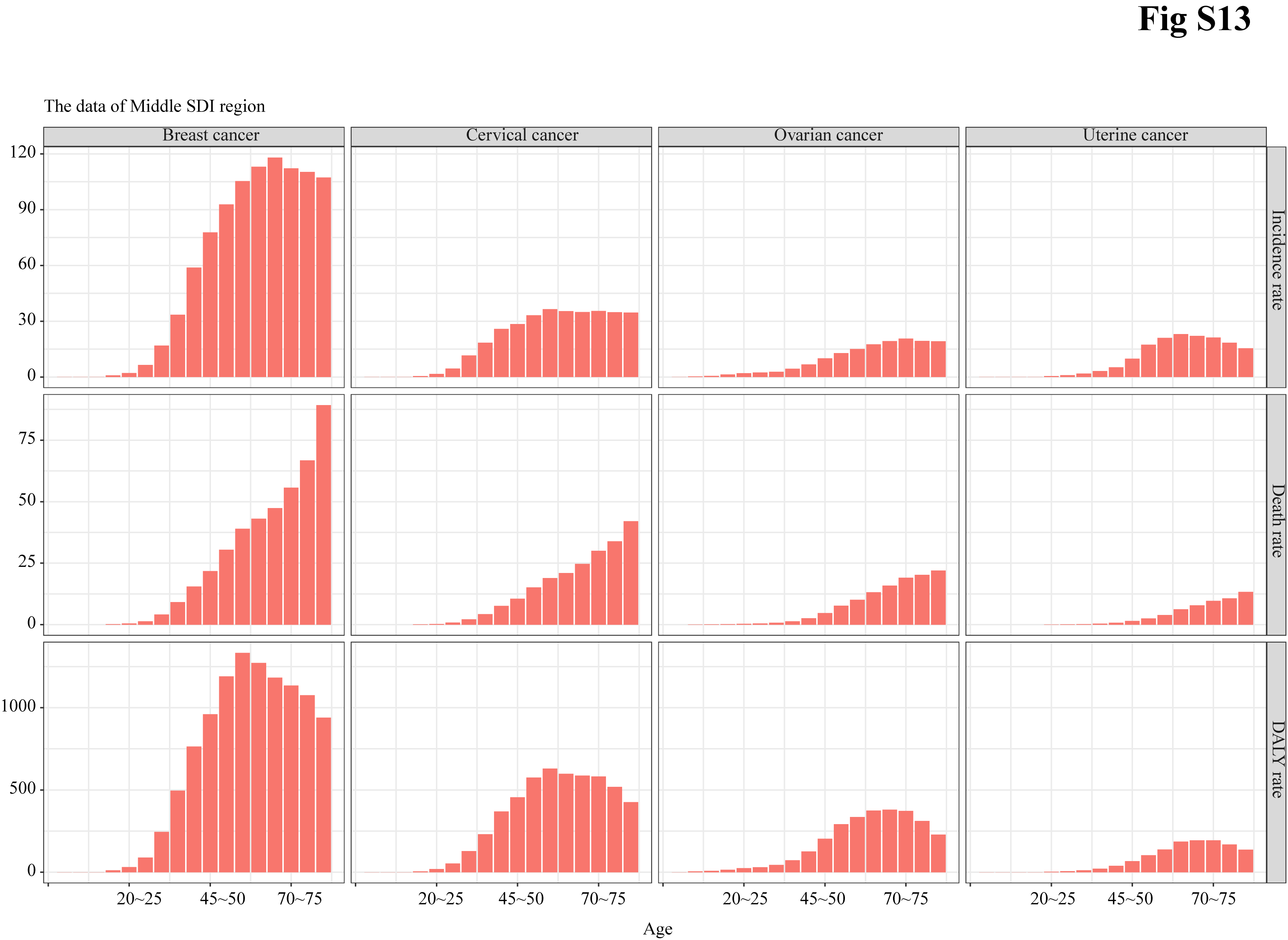

Supplement: Supplementary file 13 — Additional file 13: Figure S13: The ASIR, ASDR, and age-standardized DALY rate of cancers in different age groups in middle SDI region in 2019. Note: ASIR, Age-standardized incidence rate; ASDR, Age-standardized death rate; DALY, disability adjusted life year. [file 40364_2021_310_MOESM13_ESM.tif]

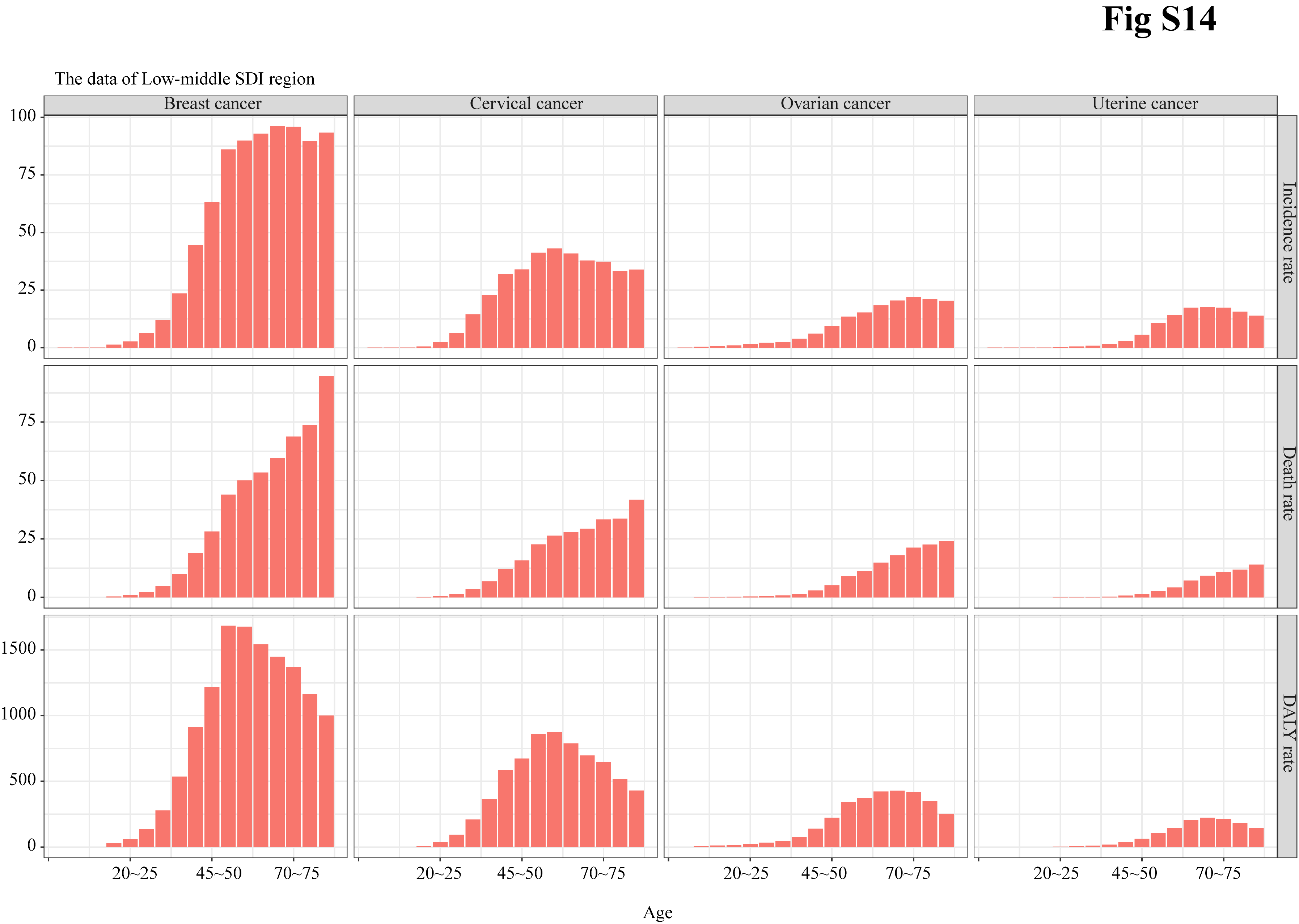

Supplement: Supplementary file 14 — Additional file 14: Figure S14: The ASIR, ASDR, and age-standardized DALY rate of cancers in different age groups in low-middle SDI region in 2019. Note: ASIR, Age-standardized incidence rate; ASDR, Age-standardized death rate; DALY, disability adjusted life year. [file 40364_2021_310_MOESM14_ESM.tif]

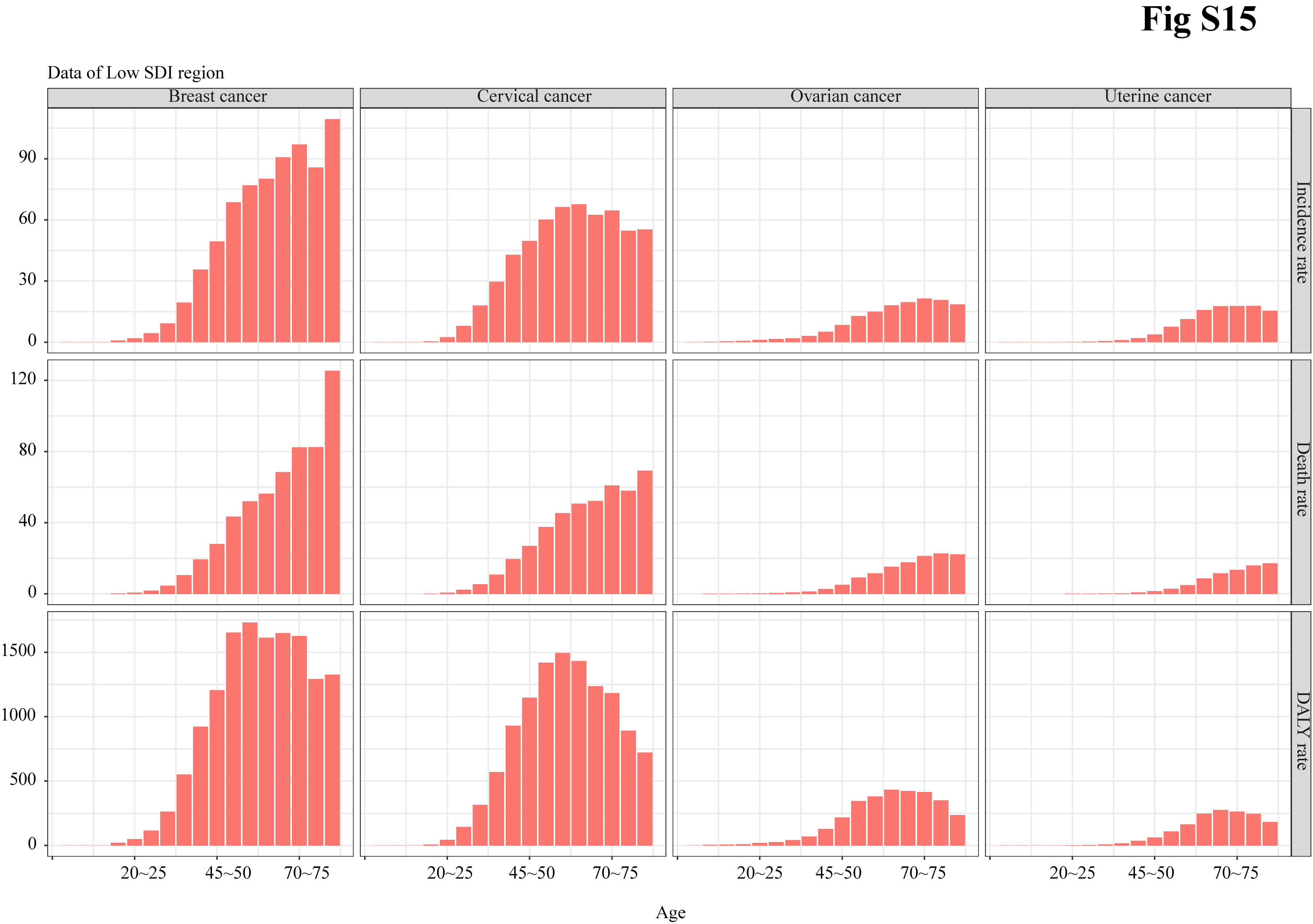

Supplement: Supplementary file 15 — Additional file 15: Figure S15: The ASIR, ASDR, and age-standardized DALY rate of cancers in different age groups in low SDI region in 2019. Note: ASIR, Age-standardized incidence rate; ASDR, Age-standardized death rate; DALY, disability adjusted life year. [file 40364_2021_310_MOESM15_ESM.tif]
