# Supplementary material for: Epidemiological trends of women’s cancers from 1990 to 2019 at the global, regional, and national levels: a population-based study
Source: Biomark Res. 2021 Jul 7;9:55. doi: 10.1186/s40364-021-00310-y (PMC8261911; doi:10.1186/s40364-021-00310-y)
Supplement: Supplementary file 16 — Additional file 16: Table S1: The incidence of female breast cancer and temporal trends. [file 40364_2021_310_MOESM16_ESM.docx]

**Table S1: The incidence of female breast cancer and temporal trends.**

|  | **1990** | | **2019** | | **1990-2019** |
| --- | --- | --- | --- | --- | --- |
|  | **Incident cases**  **No *10^3^ (95% UI)** | **ASIR /100,000**  **No. (95% UI)** | **Incident cases**  **No *10^3^ (95% UI)** | **ASIR /100,000**  **No. (95% UI)** | **EAPC**  **No. (95% CI)** |
| **Overall** | 867.62 (840.4~894.76) | 40.12 (38.78~41.33) | 1977.21 (1807.61~2145.21) | 45.86 (41.91~49.76) | 0.36 (0.31~0.42) |
| **Socio-demographic factor** | | | | | |
| **High SDI** | 428.09 (413.89~437.16) | 79.3 (77~80.87) | 673.15 (601.27~747.67) | 79.22 (70.83~87.7) | -0.12 (-0.22~-0.01) |
| **High-middle SDI** | 226.75 (219.26~234.58) | 38.52 (37.22~39.86) | 510.3 (458.38~567.97) | 48.93 (43.84~54.49) | 0.74 (0.65~0.83) |
| **Middle SDI** | 124.2 (114.52~134.28) | 20.81 (19.25~22.45) | 485.83 (430.22~545.19) | 35.52 (31.47~39.81) | 1.87 (1.84~1.9) |
| **Low-middle SDI** | 64.49 (56.63~72.66) | 18.8 (16.33~21.13) | 227.24 (199.11~256.01) | 29.47 (25.91~33.2) | 1.44 (1.33~1.54) |
| **Low SDI** | 23.57 (19.45~27.52) | 17.43 (14.22~20.52) | 79.45 (69.2~90.89) | 25.67 (22.54~29.1) | 1.3 (1.25~1.36) |
| **Region** | | | | | |
| **Andean Latin America** | 2.26 (2.02~2.53) | 19.22 (17.23~21.54) | 8.97 (7.27~11.03) | 29.63 (24.05~36.45) | 1.35 (1.23~1.46) |
| **Australasia** | 10.11 (9.66~10.5) | 85.02 (81.28~88.27) | 19.15 (15.49~23.74) | 84.69 (68.25~104.99) | -0.26 (-0.44~-0.08) |
| **Caribbean** | 6 (5.71~6.31) | 43.15 (41.14~45.36) | 14.94 (12.62~17.58) | 55.37 (46.63~65.11) | 1.01 (0.93~1.09) |
| **Central Asia** | 9.82 (9.45~10.22) | 35.4 (34.09~36.86) | 17.75 (15.77~19.92) | 38.36 (34.23~42.8) | 0.33 (0.29~0.37) |
| **Central Europe** | 36.2 (35.14~37.18) | 46.42 (45.08~47.66) | 60.77 (52.62~69.89) | 60.22 (52.04~69.57) | 0.93 (0.81~1.05) |
| **Central Latin America** | 11.92 (11.57~12.23) | 24.06 (23.29~24.71) | 50.56 (42.5~60.05) | 38.45 (32.3~45.64) | 1.56 (1.45~1.66) |
| **Central Sub-Saharan Africa** | 2.89 (2.26~3.59) | 20.94 (16.67~25.61) | 9.71 (6.95~12.82) | 28.98 (20.86~38.55) | 1.02 (0.86~1.19) |
| **East Asia** | 85.21 (70.23~100.98) | 17.23 (14.27~20.37) | 382.32 (303.31~477.17) | 35.69 (28.32~44.54) | 2.67 (2.58~2.77) |
| **Eastern Europe** | 62.24 (60.52~64.59) | 39.69 (38.55~41.28) | 93.97 (80.45~110.27) | 51.89 (44.14~61.31) | 0.68 (0.5~0.86) |
| **Eastern Sub-Saharan Africa** | 7.86 (6.46~9.32) | 17.94 (14.68~21.14) | 23.91 (20.17~27.84) | 24.04 (20.78~27.49) | 0.93 (0.81~1.05) |
| **High-income Asia Pacific** | 35.59 (33.6~37.64) | 32.74 (30.94~34.62) | 97.17 (80.97~115.11) | 56.3 (47.14~67.18) | 2.15 (1.91~2.39) |
| **High-income North America** | 205.88 (198.4~211.12) | 114.22 (110.57~116.81) | 280.02 (233.43~334.69) | 93.75 (78.03~112.64) | -0.95 (-1.05~-0.85) |
| **North Africa and Middle East** | 19.61 (17.75~22.42) | 19.64 (17.76~22.61) | 94.75 (82.33~108.87) | 37.48 (32.68~42.94) | 2.31 (2.25~2.37) |
| **Oceania** | 0.84 (0.67~1.05) | 45.27 (36.44~55.41) | 2.99 (2.29~3.84) | 65.58 (50.44~83.58) | 1.27 (1.23~1.31) |
| **South Asia** | 54.03 (44.07~62.38) | 17.05 (13.65~19.79) | 215.79 (178.05~256.86) | 27.72 (22.91~33) | 1.55 (1.42~1.68) |
| **Southeast Asia** | 44.45 (39.53~50.64) | 27.34 (24.58~30.96) | 138.54 (118.94~161.24) | 38.52 (33.11~44.64) | 1.15 (1.09~1.2) |
| **Southern Latin America** | 12.01 (11.56~12.52) | 47.99 (46.09~50.01) | 24.59 (19.22~31.15) | 56.51 (43.78~71.94) | 0.4 (0.26~0.55) |
| **Southern Sub-Saharan Africa** | 4.33 (3.89~4.81) | 25.67 (22.94~28.83) | 11.54 (10.26~12.97) | 33.89 (30.14~38.02) | 1.27 (1.12~1.43) |
| **Tropical Latin America** | 17.03 (16.39~17.61) | 31.73 (30.41~32.83) | 53.2 (49.82~56.53) | 39.75 (37.24~42.24) | 0.71 (0.48~0.94) |
| **Western Europe** | 229.06 (221.41~234.54) | 81.08 (78.79~82.91) | 338.61 (292.3~386.97) | 85.85 (74.12~98.85) | 0.07 (-0.11~0.24) |
| **Western Sub-Saharan Africa** | 10.29 (8.14~12.82) | 21.95 (17.39~27.21) | 37.98 (29.49~46.86) | 32.91 (25.93~40.11) | 1.48 (1.4~1.57) |

**Note: ASIR:** age-standardized incidence rate
