# Supplementary material for: Epidemiological trends of women’s cancers from 1990 to 2019 at the global, regional, and national levels: a population-based study
Source: Biomark Res. 2021 Jul 7;9:55. doi: 10.1186/s40364-021-00310-y (PMC8261911; doi:10.1186/s40364-021-00310-y)
Supplement: Supplementary file 17 — Additional file 17: Table S2: The death of female breast cancer and temporal trends. [file 40364_2021_310_MOESM17_ESM.docx]

**Table S2: The death of female breast cancer and temporal trends.**

|  | **1990** | | **2019** | | **1990-2019** |
| --- | --- | --- | --- | --- | --- |
|  | **Death cases**  **No *10^3^ (95% UI)** | **ASDR /100,000**  **No. (95% UI)** | **Death cases**  **No *10^3^ (95% UI)** | **ASDR /100,000**  **No. (95% UI)** | **EAPC**  **No. (95% CI)** |
| **Overall** | 375.02 (358.98~390.82) | 17.76 (16.93~18.51) | 688.56 (635.32~739.57) | 15.88 (14.66~17.07) | -0.51 (-0.57~-0.46) |
| **Socio-demographic factor** | | | | | |
| **High SDI** | 136.47 (129.81~139.84) | 23.87 (22.83~24.39) | 165.97 (150.34~175.16) | 16.71 (15.56~17.45) | -1.36 (-1.41~-1.31) |
| **High-middle SDI** | 103.59 (99.76~107.38) | 17.66 (16.98~18.33) | 163.52 (150.46~177.19) | 14.93 (13.75~16.19) | -0.8 (-0.93~-0.68) |
| **Middle SDI** | 71.27 (66.32~77.26) | 12.67 (11.79~13.68) | 181.12 (162.72~201.67) | 13.66 (12.3~15.18) | 0.21 (0.17~0.26) |
| **Low-middle SDI** | 45.11 (39.22~50.96) | 13.97 (11.93~15.79) | 124.91 (107.97~142.59) | 16.86 (14.59~19.24) | 0.52 (0.42~0.62) |
| **Low SDI** | 18.32 (15.13~21.68) | 14.42 (11.76~17.37) | 52.55 (45.73~60.01) | 18.34 (15.98~20.84) | 0.78 (0.73~0.82) |
| **Region** | | | | | |
| **Andean Latin America** | 1.43 (1.28~1.59) | 12.73 (11.44~14.21) | 3.76 (3.1~4.61) | 12.67 (10.44~15.51) | -0.21 (-0.32~-0.1) |
| **Australasia** | 3.25 (3.08~3.35) | 26.46 (25.26~27.3) | 4.45 (4~4.8) | 17.47 (16.11~18.69) | -1.6 (-1.71~-1.49) |
| **Caribbean** | 2.71 (2.56~2.87) | 20.02 (18.88~21.15) | 5.71 (4.85~6.67) | 20.84 (17.62~24.4) | 0.28 (0.2~0.37) |
| **Central Asia** | 5.21 (5.02~5.4) | 18.88 (18.18~19.56) | 7.53 (6.75~8.41) | 17.29 (15.53~19.16) | -0.24 (-0.34~-0.14) |
| **Central Europe** | 17.41 (16.9~17.86) | 21.78 (21.1~22.35) | 23.04 (20.12~26.22) | 19.87 (17.25~22.71) | -0.36 (-0.44~-0.28) |
| **Central Latin America** | 5.75 (5.58~5.89) | 12.38 (11.91~12.71) | 16.68 (14.31~19.57) | 12.87 (11.05~15.09) | 0.13 (0.05~0.2) |
| **Central Sub-Saharan Africa** | 2.26 (1.8~2.77) | 17.84 (14.45~21.48) | 6.85 (4.96~9.01) | 22.42 (16.16~29.76) | 0.69 (0.54~0.83) |
| **East Asia** | 43.3 (36.02~51.02) | 9.2 (7.68~10.78) | 98.16 (79.22~120.11) | 9.12 (7.36~11.13) | -0.1 (-0.18~-0.03) |
| **Eastern Europe** | 29.73 (28.91~30.66) | 17.93 (17.43~18.54) | 34.96 (30.28~40.41) | 17.47 (15.05~20.36) | -0.61 (-0.91~-0.32) |
| **Eastern Sub-Saharan Africa** | 6.2 (5.11~7.33) | 15.18 (12.37~17.91) | 16.39 (14.02~18.92) | 18.15 (15.65~20.6) | 0.57 (0.46~0.67) |
| **High-income Asia Pacific** | 9.52 (9.14~9.76) | 8.71 (8.34~8.92) | 20.53 (17.76~22.34) | 9.78 (8.91~10.41) | 0.57 (0.42~0.71) |
| **High-income North America** | 52.49 (49.83~53.91) | 27.54 (26.42~28.19) | 60.92 (56.3~64.17) | 18.36 (17.28~19.19) | -1.59 (-1.68~-1.49) |
| **North Africa and Middle East** | 11.52 (10.4~13.31) | 12.28 (11.03~14.23) | 35.41 (30.68~40.57) | 15.22 (13.31~17.35) | 0.77 (0.7~0.85) |
| **Oceania** | 0.57 (0.45~0.7) | 32.82 (26.12~40.1) | 1.8 (1.38~2.29) | 42.8 (33.19~54.23) | 0.95 (0.91~1) |
| **South Asia** | 39.45 (32.07~45.57) | 13.41 (10.6~15.75) | 125.31 (103.08~149.36) | 16.83 (13.91~20) | 0.61 (0.49~0.73) |
| **Southeast Asia** | 28.84 (26.02~32.88) | 18.83 (17.11~21.29) | 66.46 (57.09~76.42) | 19.23 (16.62~22.01) | -0.01 (-0.08~0.05) |
| **Southern Latin America** | 7.17 (6.9~7.41) | 28.63 (27.55~29.61) | 11.15 (10.31~11.91) | 24.04 (22.41~25.61) | -0.74 (-0.85~-0.63) |
| **Southern Sub-Saharan Africa** | 2.94 (2.62~3.31) | 18.46 (16.33~21.11) | 7.12 (6.33~7.95) | 22.06 (19.72~24.54) | 0.94 (0.73~1.14) |
| **Tropical Latin America** | 8.95 (8.62~9.26) | 17.74 (16.93~18.39) | 20.3 (18.91~21.54) | 15.19 (14.15~16.11) | -0.6 (-0.76~-0.43) |
| **Western Europe** | 88.4 (83.99~90.61) | 28.37 (27.19~28.98) | 97.51 (87.38~103.33) | 19.79 (18.32~20.77) | -1.42 (-1.48~-1.36) |
| **Western Sub-Saharan Africa** | 7.92 (6.43~9.93) | 17.7 (14.33~22.18) | 24.51 (19.23~30.84) | 23.25 (18.65~28.62) | 0.98 (0.91~1.05) |

**Note: ASDR:** age-standardized death rate
