# Supplementary material for: Epidemiological trends of women’s cancers from 1990 to 2019 at the global, regional, and national levels: a population-based study
Source: Biomark Res. 2021 Jul 7;9:55. doi: 10.1186/s40364-021-00310-y (PMC8261911; doi:10.1186/s40364-021-00310-y)
Supplement: Supplementary file 18 — Additional file 18: Table S3: The Disability-Adjusted Life Year (DALY) of female breast cancer and temporal trends. [file 40364_2021_310_MOESM18_ESM.docx]

**Table S3: The Disability-Adjusted Life Year (DALY) of female breast cancer and temporal trends.**

|  | **1990** | | **2019** | | **1990-2019** |
| --- | --- | --- | --- | --- | --- |
|  | **DALY**  **No *10^3^ (95% UI)** | **Age-standardized DALY rate /100,000**  **No. (95% UI)** | **DALY**  **No *10^3^ (95% UI)** | **Age-standardized DALY rate /100,000**  **No. (95% UI)** | **EAPC**  **No. (95% CI)** |
| **Overall** | 11526.68 (11021.13~12107.83) | 524.87 (501.78~551.15) | 20310.19 (18744.8~21866.65) | 473.83 (437.3~510.51) | -0.51 (-0.57~-0.45) |
| **Socio-demographic factor** | | | | | |
| **High SDI** | 3688.98 (3562.26~3819.43) | 703.06 (680.17~726.62) | 4046.49 (3779.7~4315.58) | 487.45 (459.76~518.84) | -1.41 (-1.47~-1.36) |
| **High-middle SDI** | 3143.41 (3024.85~3277.61) | 534.27 (513.62~557.28) | 4507.16 (4147.35~4888.83) | 434.96 (400.69~473.31) | -0.97 (-1.09~-0.85) |
| **Middle SDI** | 2469.06 (2297.1~2681.3) | 399.52 (372.02~433.14) | 5823.38 (5223.62~6455.65) | 422.91 (378.62~468.87) | 0.14 (0.1~0.18) |
| **Low-middle SDI** | 1581.14 (1390.11~1789.99) | 436.47 (381.96~493.02) | 4128.62 (3554.63~4720.57) | 523.52 (452.09~597.21) | 0.48 (0.37~0.59) |
| **Low SDI** | 636.33 (533.37~739.56) | 437.94 (363.58~513.68) | 1789.1 (1554.12~2051.72) | 544.03 (475.61~621.56) | 0.67 (0.62~0.73) |
| **Region** | | | | | |
| **Andean Latin America** | 47.26 (42.45~52.93) | 385.28 (345.28~431.27) | 113.07 (91.32~140.75) | 370.35 (300.19~459.51) | -0.36 (-0.47~-0.24) |
| **Australasia** | 92.34 (88.7~96.02) | 794.4 (763.72~825.24) | 113.16 (104.13~123.47) | 509.03 (471.05~552.78) | -1.72 (-1.82~-1.63) |
| **Caribbean** | 84.25 (78.82~90.55) | 596.8 (559.99~637.9) | 166.49 (138.08~197.24) | 623.62 (515.19~740.86) | 0.27 (0.2~0.35) |
| **Central Asia** | 170.99 (164.77~177.41) | 612.61 (589.89~635.8) | 246.48 (217.86~278.22) | 523.88 (464.12~588.67) | -0.6 (-0.7~-0.49) |
| **Central Europe** | 501.39 (487.09~514.86) | 646.94 (628.68~664.47) | 555.79 (483.44~638.98) | 552.66 (476.95~638.66) | -0.62 (-0.7~-0.54) |
| **Central Latin America** | 193.1 (188.26~198.08) | 372.32 (362.37~382.23) | 516.96 (438.01~610.43) | 390.08 (330.64~460.41) | 0.14 (0.07~0.22) |
| **Central Sub-Saharan Africa** | 78.19 (61.26~96.46) | 516.64 (410.33~631.24) | 228.64 (165.7~300.18) | 627.53 (452.46~827.77) | 0.57 (0.44~0.7) |
| **East Asia** | 1489.52 (1240.23~1764.16) | 295.76 (247.21~349.22) | 3024.99 (2477.98~3659.37) | 282.15 (230.81~341.19) | -0.31 (-0.39~-0.22) |
| **Eastern Europe** | 904.28 (877.65~938.5) | 580.67 (563.76~603.07) | 955.44 (831.14~1113.08) | 529.13 (456.07~618.57) | -0.91 (-1.21~-0.61) |
| **Eastern Sub-Saharan Africa** | 213.51 (174.23~253.36) | 447.71 (369.21~527.69) | 541.26 (454.61~633.65) | 501.54 (427.72~580.64) | 0.29 (0.17~0.41) |
| **High-income Asia Pacific** | 318.65 (307.33~331.52) | 295.1 (284.83~306.71) | 534.73 (484.95~582.46) | 321.94 (300.1~349.17) | 0.4 (0.24~0.56) |
| **High-income North America** | 1457.55 (1401.42~1521.28) | 838.52 (809.43~872.63) | 1546.6 (1447.96~1652.15) | 533.82 (502.49~569.8) | -1.77 (-1.87~-1.66) |
| **North Africa and Middle East** | 408.09 (370.19~472.05) | 395.45 (357.63~458.53) | 1222.83 (1053.07~1411.01) | 472.73 (409~544.75) | 0.61 (0.54~0.69) |
| **Oceania** | 21.32 (16.73~26.54) | 1086.68 (862.15~1349.77) | 68.06 (51.63~87.9) | 1416.87 (1084.79~1808.02) | 0.96 (0.91~1.02) |
| **South Asia** | 1383.36 (1149.02~1582.89) | 406.15 (333.57~466.21) | 4153.49 (3398.96~4965.69) | 520.59 (426.84~620.44) | 0.7 (0.57~0.83) |
| **Southeast Asia** | 1052.88 (938.79~1215.77) | 623.5 (559.91~714.09) | 2264.89 (1940.15~2631.32) | 621.22 (534.07~719.1) | -0.08 (-0.14~-0.01) |
| **Southern Latin America** | 198.02 (191.96~204.49) | 794.89 (769.75~820.7) | 275.6 (257.57~296.68) | 643.67 (602.3~691.61) | -0.87 (-0.95~-0.78) |
| **Southern Sub-Saharan Africa** | 93.79 (84.7~102.88) | 532.56 (480.15~590.83) | 206.57 (181.58~235.19) | 588.03 (517.74~667.08) | 0.77 (0.55~0.99) |
| **Tropical Latin America** | 290.82 (281.27~301.24) | 521.91 (503.7~540.25) | 604.64 (568.71~642.19) | 451.18 (424.36~479.09) | -0.55 (-0.71~-0.39) |
| **Western Europe** | 2273.77 (2192.53~2347.26) | 827.52 (801.86~852.63) | 2165.27 (2015.07~2318.47) | 552.56 (519.78~591.68) | -1.6 (-1.66~-1.53) |
| **Western Sub-Saharan Africa** | 253.61 (203.84~319.02) | 513.69 (412.5~642.6) | 805.23 (619.15~1027.29) | 650.96 (506.41~818.74) | 0.86 (0.77~0.94) |
