# Supplementary material for: Epidemiological trends of women’s cancers from 1990 to 2019 at the global, regional, and national levels: a population-based study
Source: Biomark Res. 2021 Jul 7;9:55. doi: 10.1186/s40364-021-00310-y (PMC8261911; doi:10.1186/s40364-021-00310-y)
Supplement: Supplementary file 19 — Additional file 19: Table S4: The incidence of female cervical cancer and temporal trends. [file 40364_2021_310_MOESM19_ESM.docx]

**Table S4: The incidence of cervical cancer and temporal trends.**

|  | **1990** | | **2019** | | **1990-2019** |
| --- | --- | --- | --- | --- | --- |
|  | **Incident cases**  **No *10^3^ (95% UI)** | **ASIR /100,000**  **No. (95% UI)** | **Incident cases**  **No *10^3^ (95% UI)** | **ASIR /100,000**  **No. (95% UI)** | **EAPC**  **No. (95% CI)** |
| **Overall** | 335.64 (300.35~393.89) | 14.91 (13.37~17.55) | 565.54 (481.52~636.43) | 13.35 (11.37~15.03) | -0.38 (-0.41~-0.34) |
| **Socio-demographic factor** | | | | | |
| **High SDI** | 59.69 (54.3~61.65) | 11.83 (10.67~12.22) | 63.86 (55.71~71.45) | 8.91 (7.74~9.99) | -0.95 (-1.06~-0.85) |
| **High-middle SDI** | 75.8 (71.53~88.88) | 12.77 (12.05~15) | 113.12 (89.78~129.15) | 11.59 (9.18~13.24) | -0.27 (-0.31~-0.22) |
| **Middle SDI** | 92.18 (81.45~116.4) | 14.87 (13.17~18.85) | 183.34 (144.49~208.86) | 13.44 (10.61~15.28) | -0.29 (-0.34~-0.24) |
| **Low-middle SDI** | 66.22 (54.06~81.76) | 18.04 (14.86~22.49) | 125.96 (107.88~150.11) | 15.78 (13.57~18.87) | -0.56 (-0.65~-0.46) |
| **Low SDI** | 41.5 (31.77~50.8) | 27.74 (21.56~34.25) | 78.82 (61.61~97.93) | 23.21 (18.31~28.76) | -0.69 (-0.73~-0.65) |
| **Region** | | | | | |
| **Andean Latin America** | 4.1 (3.45~4.86) | 33.39 (28.2~39.63) | 9.1 (6.93~11.61) | 29.74 (22.67~37.83) | -0.53 (-0.66~-0.4) |
| **Australasia** | 1.37 (1.15~1.47) | 11.83 (9.76~12.65) | 1.65 (1.27~2.11) | 8.22 (6.32~10.59) | -0.98 (-1.37~-0.59) |
| **Caribbean** | 4.12 (3.33~4.72) | 28 (22.69~31.86) | 6.86 (5.36~8.5) | 26.23 (20.41~32.58) | -0.24 (-0.3~-0.19) |
| **Central Asia** | 5.27 (4.9~5.63) | 18.58 (17.37~19.85) | 7.67 (6.65~8.83) | 16 (13.94~18.4) | -0.34 (-0.46~-0.23) |
| **Central Europe** | 15.39 (14.39~16.21) | 20.67 (19.23~21.74) | 13.68 (11.26~15.9) | 15.8 (12.97~18.48) | -1.08 (-1.22~-0.95) |
| **Central Latin America** | 17.08 (15.8~17.85) | 32.3 (29.43~33.73) | 28.48 (23.11~35.03) | 21.45 (17.44~26.37) | -1.77 (-1.93~-1.61) |
| **Central Sub-Saharan Africa** | 5.84 (3.95~7.83) | 37.38 (25.91~49.42) | 12.3 (8.23~16.88) | 32.32 (21.74~44.74) | -0.51 (-0.61~-0.41) |
| **East Asia** | 45.26 (35.38~79.36) | 9 (7.08~15.63) | 115.38 (64.35~147.12) | 11.17 (6.25~14.26) | 1.33 (1.11~1.56) |
| **Eastern Europe** | 22.82 (19.67~24.65) | 14.53 (12.66~15.79) | 23 (18.91~28.03) | 14.76 (11.91~18.14) | 0.03 (-0.13~0.19) |
| **Eastern Sub-Saharan Africa** | 19.08 (14.41~23.81) | 38.27 (28.81~47.55) | 36.33 (25.76~48.45) | 31.79 (22.9~41.68) | -0.8 (-0.87~-0.72) |
| **High-income Asia Pacific** | 12.47 (11.64~14.36) | 11.65 (10.81~13.42) | 15.06 (11.91~17.96) | 10.33 (7.99~12.4) | -0.17 (-0.28~-0.05) |
| **High-income North America** | 17.53 (15.11~18.26) | 10.39 (8.89~10.83) | 21.85 (17.42~26.62) | 8.93 (7.09~10.93) | -0.58 (-0.72~-0.43) |
| **North Africa and Middle East** | 7.03 (5.03~8.03) | 6.9 (4.92~7.88) | 14.63 (11.14~17.63) | 5.78 (4.43~6.89) | -0.63 (-0.72~-0.54) |
| **Oceania** | 0.57 (0.4~0.76) | 29.58 (21.39~39.83) | 1.33 (0.86~1.82) | 28.22 (19~38.09) | -0.05 (-0.12~0.03) |
| **South Asia** | 56.36 (44.21~68.59) | 16.04 (12.64~19.66) | 100.02 (80.11~124.77) | 12.37 (9.94~15.46) | -1.09 (-1.3~-0.89) |
| **Southeast Asia** | 31.13 (23.52~38.68) | 18.75 (14.3~23.63) | 52.06 (41.93~68.67) | 14.48 (11.73~19) | -1.07 (-1.17~-0.96) |
| **Southern Latin America** | 6.48 (6.05~6.87) | 26.3 (24.53~27.91) | 9.84 (7.27~12.85) | 24.85 (18.23~32.74) | -0.39 (-0.52~-0.24) |
| **Southern Sub-Saharan Africa** | 6.17 (4.68~7.53) | 33.33 (25.19~40.62) | 12.02 (9.74~14.44) | 32.9 (26.88~39.48) | 0.28 (0.05~0.52) |
| **Tropical Latin America** | 14.12 (13.36~16.36) | 24.52 (23.12~28.28) | 23.74 (22.13~27.18) | 17.91 (16.69~20.43) | -1.29 (-1.4~-1.19) |
| **Western Europe** | 28.6 (25.91~29.68) | 11.19 (9.88~11.62) | 27.17 (22.69~31.7) | 8.26 (6.85~9.68) | -0.97 (-1.07~-0.87) |
| **Western Sub-Saharan Africa** | 14.85 (11.66~18.64) | 28.64 (22.59~35.8) | 33.37 (26.14~42.54) | 25.47 (20.17~31.94) | -0.35 (-0.4~-0.31) |

**Note: ASIR:** age-standardized incidence rate
