# Supplementary material for: Epidemiological trends of women’s cancers from 1990 to 2019 at the global, regional, and national levels: a population-based study
Source: Biomark Res. 2021 Jul 7;9:55. doi: 10.1186/s40364-021-00310-y (PMC8261911; doi:10.1186/s40364-021-00310-y)
Supplement: Supplementary file 20 — Additional file 20: Table S5: The death of female cervical cancer and temporal trends. [file 40364_2021_310_MOESM20_ESM.docx]

**Table S5: The death of cervical cancer and temporal trends.**

|  | **1990** | | **2019** | | **1990-2019** |
| --- | --- | --- | --- | --- | --- |
|  | **Death cases**  **No *10^3^ (95% UI)** | **ASDR /100,000**  **No. (95% UI)** | **Death cases**  **No *10^3^ (95% UI)** | **ASDR /100,000**  **No. (95% UI)** | **EAPC**  **No. (95% CI)** |
| **Overall** | 184.53 (164.84~218.94) | 8.48 (7.59~10.07) | 280.48 (238.86~313.93) | 6.51 (5.55~7.29) | -0.93 (-0.98~-0.88) |
| **Socio-demographic factor** | | | | | |
| **High SDI** | 25.22 (23.28~26.19) | 4.56 (4.22~4.71) | 26.17 (22.82~28.15) | 2.9 (2.6~3.1) | -1.57 (-1.68~-1.46) |
| **High-middle SDI** | 41.35 (38.69~48.4) | 6.95 (6.5~8.13) | 51.77 (41.66~57.87) | 4.89 (3.92~5.47) | -1.25 (-1.31~-1.19) |
| **Middle SDI** | 52.53 (46.63~65.12) | 9.32 (8.31~11.54) | 90.1 (71.33~103.2) | 6.78 (5.4~7.76) | -1.03 (-1.09~-0.97) |
| **Low-middle SDI** | 39.21 (32.46~50.05) | 11.71 (9.73~15.05) | 66.68 (57.27~81.24) | 8.85 (7.62~10.83) | -1.04 (-1.12~-0.96) |
| **Low SDI** | 26.08 (20.23~32.11) | 19.18 (15~23.66) | 45.54 (35.8~56.26) | 15.05 (11.92~18.46) | -0.9 (-0.94~-0.86) |
| **Region** | | | | | |
| **Andean Latin America** | 2.33 (1.96~2.76) | 20.39 (17.22~24.05) | 4.28 (3.32~5.38) | 14.37 (11.18~18.04) | -1.33 (-1.45~-1.21) |
| **Australasia** | 0.45 (0.39~0.48) | 3.73 (3.16~3.92) | 0.52 (0.45~0.58) | 2.17 (1.88~2.4) | -1.57 (-1.97~-1.17) |
| **Caribbean** | 2.23 (1.75~2.55) | 15.83 (12.55~18.04) | 3.47 (2.72~4.26) | 12.95 (10.11~15.96) | -0.65 (-0.72~-0.58) |
| **Central Asia** | 2.72 (2.48~2.89) | 9.81 (8.95~10.43) | 3.42 (3~3.93) | 7.58 (6.68~8.7) | -0.75 (-0.89~-0.61) |
| **Central Europe** | 8 (7.6~8.53) | 10.14 (9.63~10.79) | 6.88 (5.82~7.99) | 6.65 (5.59~7.75) | -1.58 (-1.69~-1.46) |
| **Central Latin America** | 9.59 (8.7~10.01) | 20.35 (18.19~21.28) | 13.83 (11.53~16.8) | 10.65 (8.91~12.92) | -2.61 (-2.76~-2.45) |
| **Central Sub-Saharan Africa** | 3.72 (2.61~4.84) | 26.27 (18.78~34.25) | 7.3 (4.91~10.06) | 21.67 (14.49~30.24) | -0.67 (-0.79~-0.54) |
| **East Asia** | 28.4 (22.32~46.14) | 6.05 (4.77~9.76) | 55.96 (33.19~71.36) | 5.18 (3.09~6.59) | -0.05 (-0.29~0.19) |
| **Eastern Europe** | 12.94 (10.95~13.86) | 7.62 (6.5~8.19) | 10.04 (8.47~11.91) | 5.54 (4.62~6.61) | -1.38 (-1.55~-1.21) |
| **Eastern Sub-Saharan Africa** | 11.94 (9.08~14.92) | 26.51 (20.07~33.45) | 21.11 (15.48~27.86) | 21.13 (15.15~27.62) | -0.9 (-0.96~-0.84) |
| **High-income Asia Pacific** | 4.64 (4.35~5.32) | 4.2 (3.93~4.81) | 5.6 (4.58~6.22) | 2.7 (2.22~2.96) | -1.52 (-1.59~-1.44) |
| **High-income North America** | 6.74 (5.97~7.04) | 3.71 (3.25~3.86) | 8.8 (7.47~9.34) | 2.99 (2.55~3.15) | -0.69 (-0.83~-0.55) |
| **North Africa and Middle East** | 3.97 (2.82~4.53) | 4.37 (3.1~4.99) | 7 (5.44~8.31) | 3.15 (2.47~3.69) | -1.11 (-1.21~-1.01) |
| **Oceania** | 0.3 (0.22~0.42) | 18.16 (13.38~25.22) | 0.67 (0.45~0.91) | 16.41 (11.5~22.19) | -0.18 (-0.27~-0.08) |
| **South Asia** | 33.34 (26.22~39.95) | 10.49 (8.29~12.62) | 53.3 (42.87~69.95) | 7.01 (5.66~9.21) | -1.6 (-1.78~-1.42) |
| **Southeast Asia** | 16.71 (12.57~21.47) | 11.05 (8.36~14.54) | 25.13 (20.52~34.98) | 7.36 (6.03~10.33) | -1.52 (-1.61~-1.43) |
| **Southern Latin America** | 3.07 (2.89~3.31) | 12.34 (11.62~13.3) | 4.18 (3.55~4.6) | 9.64 (8.17~10.56) | -1.01 (-1.13~-0.89) |
| **Southern Sub-Saharan Africa** | 3.24 (2.48~4.07) | 19.17 (14.63~24.27) | 6.56 (5.39~7.75) | 19.34 (15.82~22.77) | 0.46 (0.18~0.73) |
| **Tropical Latin America** | 7.68 (7.21~8.88) | 14.72 (13.7~17.12) | 11.58 (10.71~13.66) | 8.69 (8.04~10.23) | -2.01 (-2.11~-1.92) |
| **Western Europe** | 13.13 (12.04~13.61) | 4.36 (3.99~4.51) | 11.75 (10.27~12.69) | 2.65 (2.38~2.85) | -1.65 (-1.76~-1.54) |
| **Western Sub-Saharan Africa** | 9.38 (7.56~12.31) | 19.74 (15.94~25.76) | 19.09 (15.04~24.01) | 16.83 (13.38~21) | -0.48 (-0.53~-0.43) |

**Note: ASDR:** age-standardized death rate
