# Supplementary material for: Epidemiological trends of women’s cancers from 1990 to 2019 at the global, regional, and national levels: a population-based study
Source: Biomark Res. 2021 Jul 7;9:55. doi: 10.1186/s40364-021-00310-y (PMC8261911; doi:10.1186/s40364-021-00310-y)
Supplement: Supplementary file 21 — Additional file 21: Table S6: The Disability-Adjusted Life Year (DALY) of female cervical cancer and temporal trends. [file 40364_2021_310_MOESM21_ESM.docx]

**Table S6: The Disability-Adjusted Life Year (DALY) of cervical cancer and temporal trends.**

|  | **1990** | | **2019** | | **1990-2019** |
| --- | --- | --- | --- | --- | --- |
|  | **DALY**  **No *10^3^ (95% UI)** | **Age-standardized DALY rate /100,000**  **No. (95% UI)** | **DALY**  **No *10^3^ (95% UI)** | **Age-standardized DALY rate /100,000**  **No. (95% UI)** | **EAPC**  **No. (95% CI)** |
| **Overall** | 6176.25 (5437.67~7316.93) | 275.05 (242.75~326.15) | 8955.01 (7547.73~9978.46) | 210.64 (177.67~234.85) | -0.95 (-1~-0.89) |
| **Socio-demographic factor** | | | | | |
| **High SDI** | 725.85 (665.2~752.59) | 143.23 (130.3~148.45) | 672.11 (608.75~722) | 89.72 (81.88~95.85) | -1.62 (-1.75~-1.49) |
| **High-middle SDI** | 1274.57 (1193.74~1497.01) | 215.21 (201.46~252.85) | 1543.7 (1236~1729.87) | 154.69 (124.02~173.51) | -1.16 (-1.23~-1.1) |
| **Middle SDI** | 1790.63 (1588.48~2223.17) | 287.82 (255.02~356.33) | 2817.25 (2223.19~3217.72) | 204.6 (161.92~233.49) | -1.11 (-1.18~-1.05) |
| **Low-middle SDI** | 1419.29 (1160.75~1789.13) | 381.9 (315.26~485.47) | 2282.24 (1948.33~2722.93) | 285.64 (244.64~342.16) | -1.08 (-1.17~-0.98) |
| **Low SDI** | 961.2 (732.59~1179.64) | 630.59 (487.61~777.41) | 1632.49 (1271.61~2044.29) | 477.53 (374.33~591.38) | -1.05 (-1.1~-1) |
| **Region** | | | | | |
| **Andean Latin America** | 79.25 (66.33~92.9) | 633.25 (529.46~743.28) | 129.59 (99.42~165.4) | 422.28 (323.97~538.4) | -1.54 (-1.67~-1.42) |
| **Australasia** | 13.93 (11.33~14.68) | 120.58 (96.79~127.24) | 13.58 (11.84~15.06) | 65.47 (57.37~72.7) | -1.83 (-2.28~-1.39) |
| **Caribbean** | 77.36 (58.62~90.62) | 526.18 (401.54~612.81) | 114.71 (86.8~145.02) | 438.19 (328.34~557.85) | -0.63 (-0.7~-0.56) |
| **Central Asia** | 89.5 (83.58~95.46) | 317.62 (299.06~339.68) | 119.72 (103.95~138.54) | 249.41 (217.42~288.15) | -0.72 (-0.87~-0.57) |
| **Central Europe** | 251.14 (236.32~264.12) | 333.96 (312.61~350.79) | 190.26 (159.63~221.33) | 212.08 (177.26~247.37) | -1.73 (-1.87~-1.6) |
| **Central Latin America** | 323.66 (302.5~337.95) | 610.29 (562.25~636.93) | 436.92 (361.75~538.52) | 328.59 (272.54~404.33) | -2.48 (-2.64~-2.32) |
| **Central Sub-Saharan Africa** | 135.73 (92.58~179.24) | 848.19 (592.2~1109.46) | 261.63 (176.04~360.43) | 678.72 (454.78~932.08) | -0.77 (-0.9~-0.64) |
| **East Asia** | 921.11 (714.66~1517.19) | 182.74 (142.14~297.89) | 1696.32 (972.55~2166.63) | 159.12 (91.62~202.95) | 0.02 (-0.21~0.25) |
| **Eastern Europe** | 368.58 (319.59~396.93) | 233.88 (203.92~253.7) | 308.61 (255.79~369.57) | 192.88 (156.89~231.51) | -0.89 (-1.1~-0.68) |
| **Eastern Sub-Saharan Africa** | 444.59 (335.91~562.71) | 876.65 (666.35~1103.42) | 758.61 (557.09~1022.32) | 660.28 (484.65~874.17) | -1.13 (-1.21~-1.06) |
| **High-income Asia Pacific** | 136.98 (127.56~156.32) | 126.77 (118.36~144.52) | 133.64 (109.97~146.61) | 85.67 (67.59~93.64) | -1.29 (-1.35~-1.22) |
| **High-income North America** | 206.87 (177.72~216.04) | 123.61 (105.3~129.07) | 245.96 (211.94~259.26) | 96.55 (83.94~101.88) | -0.84 (-1~-0.68) |
| **North Africa and Middle East** | 133 (94.24~152.1) | 130.16 (92.26~148.26) | 221.93 (169.2~268.19) | 88.28 (68.42~105.74) | -1.37 (-1.44~-1.29) |
| **Oceania** | 11.34 (7.93~15.07) | 579.78 (417.98~793.65) | 24.91 (16.09~34.06) | 521.37 (347.49~709.69) | -0.17 (-0.27~-0.07) |
| **South Asia** | 1230.95 (969.02~1468.76) | 345.39 (271.36~413.3) | 1833.69 (1466.66~2370.91) | 226.59 (181.92~292.64) | -1.64 (-1.84~-1.44) |
| **Southeast Asia** | 592.31 (440.72~733.9) | 354.96 (266.86~447.24) | 808.25 (653.21~1088.29) | 223.36 (181.44~302.65) | -1.73 (-1.83~-1.63) |
| **Southern Latin America** | 101.76 (96.27~107.78) | 413.34 (390.66~437.19) | 127.49 (105.4~140.05) | 317.23 (260.3~348.08) | -1.07 (-1.18~-0.95) |
| **Southern Sub-Saharan Africa** | 116.35 (89.85~142.79) | 633.63 (488.91~782.66) | 213.94 (173.97~254.95) | 586.79 (476.2~698.37) | 0.23 (-0.05~0.52) |
| **Tropical Latin America** | 262.8 (247.91~303.91) | 455.15 (428.99~524.5) | 365.28 (340.28~419.75) | 274.27 (255.5~314.34) | -1.95 (-2.06~-1.84) |
| **Western Europe** | 351.5 (321.56~363.85) | 134.35 (119.97~138.99) | 277.36 (248.48~299.74) | 79.19 (71.88~85.34) | -1.77 (-1.9~-1.63) |
| **Western Sub-Saharan Africa** | 327.53 (261.59~424.73) | 626.47 (502.32~815.1) | 672.6 (524.74~854.86) | 507.97 (398.99~640.76) | -0.69 (-0.74~-0.64) |
