# Supplementary material for: Epidemiological trends of women’s cancers from 1990 to 2019 at the global, regional, and national levels: a population-based study
Source: Biomark Res. 2021 Jul 7;9:55. doi: 10.1186/s40364-021-00310-y (PMC8261911; doi:10.1186/s40364-021-00310-y)
Supplement: Supplementary file 22 — Additional file 22:Table S7: The incidence of female ovarian cancer and temporal trends. [file 40364_2021_310_MOESM22_ESM.docx]

**Table S7: The incidence of ovarian cancer and temporal trends.**

|  | **1990** | | **2019** | | **1990-2019** |
| --- | --- | --- | --- | --- | --- |
|  | **Incident cases**  **No *10^3^ (95% UI)** | **ASIR /100,000**  **No. (95% UI)** | **Incident cases**  **No *10^3^ (95% UI)** | **ASIR /100,000**  **No. (95% UI)** | **EAPC**  **No. (95% CI)** |
| **Overall** | 141.71 (130.54~160.78) | 6.46 (5.97~7.29) | 294.42 (260.65~329.73) | 6.87 (6.08~7.7) | 0.11 (0.07~0.15) |
| **Socio-demographic factor** | | | | | |
| **High SDI** | 62.46 (56.72~64.47) | 11.46 (10.4~11.8) | 80.45 (70.5~91.46) | 9.3 (8.23~10.56) | -0.87 (-0.96~-0.78) |
| **High-middle SDI** | 43.57 (40.3~46.7) | 7.31 (6.75~7.84) | 77.29 (65.89~86.46) | 7.56 (6.43~8.45) | 0.03 (-0.04~0.09) |
| **Middle SDI** | 21.29 (18.3~27.01) | 3.42 (2.98~4.3) | 76.55 (63.25~88.97) | 5.71 (4.7~6.63) | 1.72 (1.66~1.77) |
| **Low-middle SDI** | 10.23 (8.03~16.7) | 2.95 (2.32~4.68) | 43.6 (35.3~54.68) | 5.65 (4.6~7.08) | 2.31 (2.27~2.35) |
| **Low SDI** | 4.1 (2.81~8.46) | 2.96 (2.04~5.89) | 16.39 (13.49~20.3) | 5.15 (4.28~6.34) | 1.99 (1.9~2.09) |
| **Region** | | | | | |
| **Andean Latin America** | 0.47 (0.38~0.69) | 3.75 (3.02~5.47) | 2.15 (1.52~2.77) | 7.05 (4.98~9.04) | 2.01 (1.78~2.24) |
| **Australasia** | 1.31 (1.15~1.39) | 10.9 (9.52~11.5) | 1.93 (1.52~2.47) | 8.24 (6.52~10.64) | -1.09 (-1.18~-1) |
| **Caribbean** | 0.29 (0.25~0.44) | 1.98 (1.74~3.05) | 1.53 (1.1~2.04) | 5.72 (4.15~7.7) | 3.63 (2.74~4.52) |
| **Central Asia** | 1.44 (1.22~1.59) | 5.04 (4.25~5.56) | 3.19 (2.76~3.57) | 6.94 (6~7.76) | 1.3 (1.19~1.4) |
| **Central Europe** | 8.73 (8.28~9.01) | 11.21 (10.6~11.57) | 11.71 (10~13.58) | 11.73 (9.96~13.69) | 0.2 (0.09~0.31) |
| **Central Latin America** | 2.64 (2.56~2.75) | 4.99 (4.8~5.28) | 9.79 (8.13~11.85) | 7.46 (6.2~9.03) | 1.48 (1.36~1.6) |
| **Central Sub-Saharan Africa** | 0.32 (0.19~0.68) | 2.22 (1.32~4.52) | 1.12 (0.73~1.81) | 3.2 (2.1~5.13) | 1.18 (0.97~1.4) |
| **East Asia** | 13.42 (10.54~18.33) | 2.61 (2.05~3.6) | 47.85 (35.08~59.6) | 4.61 (3.41~5.74) | 1.89 (1.8~1.98) |
| **Eastern Europe** | 15.65 (13.5~16.56) | 9.9 (8.38~10.52) | 18.95 (15.9~22.9) | 10.84 (9.01~13.11) | 0.27 (0.17~0.37) |
| **Eastern Sub-Saharan Africa** | 1.77 (1.1~3.94) | 3.87 (2.44~8.28) | 6.35 (5.05~7.7) | 6 (4.86~7.18) | 1.51 (1.4~1.63) |
| **High-income Asia Pacific** | 6.62 (6.31~6.96) | 6.13 (5.84~6.43) | 11.88 (9.65~14.07) | 6.85 (5.55~8.16) | 0.32 (0.18~0.46) |
| **High-income North America** | 23.11 (21.22~23.96) | 12.69 (11.73~13.12) | 29.79 (24.35~36.25) | 9.85 (7.96~12.05) | -1.03 (-1.15~-0.91) |
| **North Africa and Middle East** | 3.29 (2.36~6.1) | 3.33 (2.42~6) | 12.88 (10.39~15.07) | 5.27 (4.29~6.15) | 1.62 (1.52~1.72) |
| **Oceania** | 0.05 (0.03~0.1) | 2.69 (1.92~5.46) | 0.18 (0.12~0.36) | 4.25 (2.89~8.03) | 1.55 (1.38~1.71) |
| **South Asia** | 10 (7.38~15.06) | 3.17 (2.42~4.63) | 45.76 (34.9~56.55) | 5.91 (4.58~7.29) | 2.19 (2.11~2.26) |
| **Southeast Asia** | 8.18 (6.45~12.13) | 4.96 (4.02~7.19) | 28.15 (21.78~39) | 7.98 (6.19~11.04) | 1.53 (1.46~1.61) |
| **Southern Latin America** | 1.97 (1.73~2.33) | 7.79 (6.86~9.21) | 3.63 (2.83~4.64) | 8.53 (6.61~10.9) | 0.18 (0.03~0.32) |
| **Southern Sub-Saharan Africa** | 0.82 (0.7~0.98) | 4.7 (3.97~5.61) | 2.32 (1.9~2.79) | 6.7 (5.5~7.97) | 1.28 (1.19~1.38) |
| **Tropical Latin America** | 3.12 (2.98~3.24) | 5.58 (5.31~5.79) | 8.06 (7.38~8.81) | 6.13 (5.61~6.7) | 0.23 (0.11~0.35) |
| **Western Europe** | 37.39 (33.29~38.63) | 12.92 (11.42~13.34) | 42.24 (36.19~49.05) | 10.38 (8.98~12.1) | -0.94 (-1.03~-0.85) |
| **Western Sub-Saharan Africa** | 1.13 (0.84~1.63) | 2.28 (1.7~3.27) | 4.96 (3.59~6.76) | 4.06 (2.87~5.53) | 2 (1.93~2.07) |

**Note: ASIR:** age-standardized incidence rate
