# Supplementary material for: Epidemiological trends of women’s cancers from 1990 to 2019 at the global, regional, and national levels: a population-based study
Source: Biomark Res. 2021 Jul 7;9:55. doi: 10.1186/s40364-021-00310-y (PMC8261911; doi:10.1186/s40364-021-00310-y)
Supplement: Supplementary file 23 — Additional file 23: Table S8: The death of female ovarian cancer and temporal trends. [file 40364_2021_310_MOESM23_ESM.docx]

**Table S8: The death of ovarian cancer and temporal trends.**

|  | **1990** | | **2019** | | **1990-2019** |
| --- | --- | --- | --- | --- | --- |
|  | **Death cases**  **No *10^3^ (95% UI)** | **ASDR /100,000**  **No. (95% UI)** | **Death cases**  **No *10^3^ (95% UI)** | **ASDR /100,000**  **No. (95% UI)** | **EAPC**  **No. (95% CI)** |
| **Overall** | 97.36 (89.7~109.76) | 4.59 (4.24~5.16) | 198.41 (175.36~217.66) | 4.56 (4.03~5) | -0.11 (-0.15~-0.07) |
| **Socio-demographic factor** | | | | | |
| **High SDI** | 43.46 (39.18~45.02) | 7.46 (6.74~7.71) | 56.64 (50.39~61.32) | 5.67 (5.16~6.09) | -1.1 (-1.17~-1.02) |
| **High-middle SDI** | 29.78 (27.76~31.76) | 4.97 (4.63~5.3) | 51.97 (45~57.25) | 4.75 (4.11~5.24) | -0.26 (-0.35~-0.17) |
| **Middle SDI** | 13.71 (12.09~17.15) | 2.44 (2.16~3.05) | 48.49 (39.89~56.53) | 3.66 (3.01~4.26) | 1.37 (1.34~1.41) |
| **Low-middle SDI** | 7.31 (5.76~11.58) | 2.33 (1.84~3.6) | 29.87 (24.42~37.62) | 4.09 (3.36~5.15) | 2.02 (1.98~2.06) |
| **Low SDI** | 3.07 (2.12~6.17) | 2.45 (1.71~4.72) | 11.35 (9.55~13.93) | 4.01 (3.38~4.88) | 1.78 (1.69~1.87) |
| **Region** | | | | | |
| **Andean Latin America** | 0.32 (0.26~0.47) | 2.84 (2.3~4.12) | 1.37 (0.97~1.74) | 4.63 (3.28~5.86) | 1.47 (1.24~1.69) |
| **Australasia** | 0.91 (0.79~0.96) | 7.2 (6.28~7.58) | 1.38 (1.18~1.59) | 5.29 (4.58~6.1) | -1.17 (-1.25~-1.1) |
| **Caribbean** | 0.19 (0.17~0.3) | 1.38 (1.21~2.13) | 1.04 (0.73~1.4) | 3.81 (2.7~5.14) | 3.46 (2.6~4.33) |
| **Central Asia** | 0.95 (0.8~1.06) | 3.42 (2.88~3.78) | 2.04 (1.77~2.29) | 4.68 (4.04~5.22) | 1.32 (1.19~1.44) |
| **Central Europe** | 6.11 (5.83~6.32) | 7.52 (7.17~7.78) | 8.65 (7.45~10.04) | 7.64 (6.57~8.88) | 0.14 (0.04~0.25) |
| **Central Latin America** | 1.69 (1.63~1.8) | 3.63 (3.47~3.88) | 6.14 (5.1~7.29) | 4.76 (3.95~5.65) | 1.02 (0.92~1.11) |
| **Central Sub-Saharan Africa** | 0.24 (0.14~0.5) | 1.87 (1.12~3.68) | 0.8 (0.52~1.3) | 2.59 (1.67~4.11) | 1.06 (0.86~1.27) |
| **East Asia** | 8.43 (6.5~12.14) | 1.78 (1.38~2.59) | 30.35 (22.12~38.07) | 2.79 (2.05~3.49) | 1.51 (1.42~1.61) |
| **Eastern Europe** | 11.13 (10~11.73) | 6.53 (5.8~6.9) | 13.28 (11.08~15.92) | 6.74 (5.61~8.05) | 0.002 (-0.11~0.11) |
| **Eastern Sub-Saharan Africa** | 1.32 (0.83~2.86) | 3.22 (2.04~6.66) | 4.46 (3.63~5.41) | 4.83 (3.95~5.78) | 1.45 (1.34~1.55) |
| **High-income Asia Pacific** | 4.13 (3.91~4.42) | 3.73 (3.53~3.99) | 7.34 (6.21~8.06) | 3.45 (3.03~3.72) | -0.38 (-0.45~-0.31) |
| **High-income North America** | 15.85 (14.33~16.51) | 8.09 (7.38~8.4) | 21.63 (19.54~23.59) | 6.41 (5.85~6.95) | -0.94 (-1.05~-0.82) |
| **North Africa and Middle East** | 2.29 (1.66~4.13) | 2.56 (1.86~4.47) | 7.83 (6.49~9.14) | 3.54 (2.93~4.17) | 1.14 (1.02~1.27) |
| **Oceania** | 0.03 (0.02~0.06) | 1.98 (1.42~3.85) | 0.11 (0.07~0.21) | 2.94 (2.05~5.34) | 1.38 (1.25~1.51) |
| **South Asia** | 7.33 (5.5~10.8) | 2.6 (2.01~3.76) | 32.11 (24.89~39.9) | 4.4 (3.39~5.48) | 1.83 (1.76~1.9) |
| **Southeast Asia** | 5.09 (4.11~7.25) | 3.45 (2.83~4.83) | 16.19 (12.72~22.68) | 4.76 (3.75~6.64) | 1.02 (0.97~1.07) |
| **Southern Latin America** | 1.47 (1.29~1.73) | 5.78 (5.08~6.8) | 2.55 (2.34~2.88) | 5.64 (5.19~6.35) | -0.2 (-0.34~-0.06) |
| **Southern Sub-Saharan Africa** | 0.58 (0.49~0.7) | 3.64 (3.03~4.36) | 1.69 (1.38~2.01) | 5.14 (4.21~6.11) | 1.36 (1.27~1.45) |
| **Tropical Latin America** | 2.09 (1.99~2.18) | 4.1 (3.88~4.29) | 5.44 (4.96~5.91) | 4.09 (3.73~4.45) | -0.11 (-0.23~0.01) |
| **Western Europe** | 26.36 (23.61~27.32) | 8.21 (7.33~8.49) | 30.61 (27.02~33.28) | 6.28 (5.7~6.76) | -1.1 (-1.17~-1.02) |
| **Western Sub-Saharan Africa** | 0.85 (0.64~1.21) | 1.87 (1.42~2.66) | 3.41 (2.43~4.57) | 3.19 (2.24~4.24) | 1.87 (1.8~1.93) |

**Note: ASDR:** age-standardized death rate
