# Supplementary material for: Epidemiological trends of women’s cancers from 1990 to 2019 at the global, regional, and national levels: a population-based study
Source: Biomark Res. 2021 Jul 7;9:55. doi: 10.1186/s40364-021-00310-y (PMC8261911; doi:10.1186/s40364-021-00310-y)
Supplement: Supplementary file 24 — Additional file 24: Table S9: The Disability-Adjusted Life Year (DALY) of female ovarian cancer and temporal trends. [file 40364_2021_310_MOESM24_ESM.docx]

**Table S9: The Disability-Adjusted Life Year (DALY) of ovarian cancer and temporal trends.**

|  | **1990** | | **2019** | | **1990-2019** |
| --- | --- | --- | --- | --- | --- |
|  | **DALY**  **No *10^3^ (95% UI)** | **Age-standardized DALY rate /100,000**  **No. (95% UI)** | **DALY**  **No *10^3^ (95% UI)** | **Age-standardized DALY rate /100,000**  **No. (95% UI)** | **EAPC**  **No. (95% CI)** |
| **Overall** | 2732.67 (2493.73~3165.17) | 124.09 (113.68~142.97) | 5359.74 (4692.95~5954.99) | 124.68 (109.13~138.67) | -0.08 (-0.12~-0.04) |
| **Socio-demographic factor** | | | | | |
| **High SDI** | 1061.1 (956.79~1094.31) | 198.32 (178.76~204.3) | 1229.12 (1125.7~1323.42) | 143.78 (132.56~154.51) | -1.27 (-1.34~-1.2) |
| **High-middle SDI** | 864.81 (793.57~925.11) | 145.06 (132.93~155.31) | 1378.23 (1191.05~1526.4) | 133.03 (114.83~147.47) | -0.43 (-0.5~-0.35) |
| **Middle SDI** | 462.01 (401.88~592.52) | 73.86 (64.7~93.48) | 1453.63 (1199.32~1696.72) | 106.38 (87.67~123.96) | 1.21 (1.17~1.25) |
| **Low-middle SDI** | 241.44 (188.02~394.77) | 67.61 (53.25~107.69) | 922.65 (740.01~1169.38) | 118.41 (95.38~150.23) | 2.02 (1.98~2.05) |
| **Low SDI** | 102.2 (69.31~215.44) | 71.03 (48.95~144.88) | 373.32 (311.09~462.23) | 115.17 (96.36~141.83) | 1.73 (1.64~1.82) |
| **Region** | | | | | |
| **Andean Latin America** | 10.64 (8.52~15.74) | 83.91 (67.22~122.98) | 40.93 (29.3~52.54) | 134.17 (95.92~171.71) | 1.45 (1.25~1.65) |
| **Australasia** | 22.73 (19.65~23.92) | 191.67 (165.99~201.8) | 29.64 (25.74~34.3) | 128.09 (111.45~149.4) | -1.51 (-1.58~-1.44) |
| **Caribbean** | 5.99 (5.19~9.69) | 41.57 (35.97~66.46) | 29.26 (21.13~40.85) | 109.44 (78.94~153.64) | 3.32 (2.49~4.16) |
| **Central Asia** | 30.23 (25.69~33.52) | 106.38 (90.39~117.89) | 64.75 (56.17~72.87) | 138.41 (119.88~155.6) | 1.06 (0.95~1.17) |
| **Central Europe** | 174.51 (166.03~180.62) | 223.65 (210.58~231.11) | 210.93 (181~245.58) | 212.29 (181.69~247.19) | -0.13 (-0.22~-0.04) |
| **Central Latin America** | 55.78 (54.06~58.41) | 105.3 (101.71~112.14) | 183.81 (152.07~220.4) | 139.44 (115.36~167.07) | 1.09 (1.01~1.16) |
| **Central Sub-Saharan Africa** | 8.06 (4.67~17.3) | 53.09 (31.29~110.5) | 26.27 (17.01~43.12) | 72.61 (47.24~118.81) | 1.01 (0.81~1.22) |
| **East Asia** | 288.35 (222.94~393.42) | 56.07 (43.42~77.85) | 871.65 (645.11~1098.32) | 81.21 (60.66~102.31) | 1.16 (1.06~1.26) |
| **Eastern Europe** | 323.95 (276.93~343) | 202.97 (170.92~215.66) | 360.69 (299.27~431.83) | 202.71 (167.41~242.93) | -0.14 (-0.26~-0.03) |
| **Eastern Sub-Saharan Africa** | 44.06 (27.03~100.76) | 92.84 (57.74~203.8) | 147.57 (117.4~181.45) | 136.91 (110.38~166.46) | 1.36 (1.25~1.48) |
| **High-income Asia Pacific** | 122.45 (117.88~129.48) | 112.95 (108.81~119.08) | 167.71 (147.32~181.07) | 101.18 (89.47~107.95) | -0.48 (-0.54~-0.41) |
| **High-income North America** | 379.32 (348.9~393.04) | 213.28 (197.04~220.61) | 472.99 (439.02~512.58) | 156.34 (145.58~170.22) | -1.21 (-1.32~-1.11) |
| **North Africa and Middle East** | 73.6 (52.49~138.53) | 73.08 (52.72~134.44) | 243.82 (198.31~284.28) | 98.96 (80.8~115.45) | 1.05 (0.94~1.15) |
| **Oceania** | 1.07 (0.75~2.32) | 58.03 (41.17~120.04) | 3.79 (2.48~7.55) | 85.48 (57.56~163.77) | 1.34 (1.22~1.47) |
| **South Asia** | 240.84 (175.11~367.24) | 72.96 (54.54~107.75) | 982.47 (748.58~1238.01) | 125.29 (95.91~157.33) | 1.91 (1.84~1.98) |
| **Southeast Asia** | 174.39 (136.91~257.86) | 105.2 (84.05~152.22) | 517.38 (404.02~726.12) | 144.22 (112.72~201.78) | 0.99 (0.94~1.05) |
| **Southern Latin America** | 40.14 (35.42~47.24) | 159.59 (140.67~187.64) | 65.52 (60.16~73.66) | 154.93 (142.56~173.7) | -0.23 (-0.36~-0.1) |
| **Southern Sub-Saharan Africa** | 17.9 (15.43~21.18) | 102.03 (86.65~122.2) | 48.66 (39.77~58.35) | 138.52 (113.33~166.72) | 1.23 (1.14~1.31) |
| **Tropical Latin America** | 66.75 (63.75~69.43) | 118.23 (112.6~123.15) | 153.73 (140.93~167.33) | 115.92 (106.16~126.13) | -0.16 (-0.25~-0.06) |
| **Western Europe** | 624.9 (553.97~645.27) | 218.59 (192.34~225.71) | 626.36 (569.66~678.62) | 154.84 (142.25~167.85) | -1.38 (-1.45~-1.31) |
| **Western Sub-Saharan Africa** | 26.99 (20.47~38.67) | 53.43 (40.56~76.88) | 111.8 (80.4~150.52) | 89.45 (63.79~120.33) | 1.78 (1.7~1.86) |
