# Supplementary material for: Epidemiological trends of women’s cancers from 1990 to 2019 at the global, regional, and national levels: a population-based study
Source: Biomark Res. 2021 Jul 7;9:55. doi: 10.1186/s40364-021-00310-y (PMC8261911; doi:10.1186/s40364-021-00310-y)
Supplement: Supplementary file 25 — Additional file 25: Table S10: The incidence of uterine cancer and temporal trends. [file 40364_2021_310_MOESM25_ESM.docx]

**Table S10: The incidence of uterine cancer and temporal trends.**

|  | **1990** | | **2019** | | **1990-2019** |
| --- | --- | --- | --- | --- | --- |
|  | **Incident cases**  **No *10^3^ (95% UI)** | **ASIR /100,000**  **No. (95% UI)** | **Incident cases**  **No *10^3^ (95% UI)** | **ASIR /100,000**  **No. (95% UI)** | **EAPC**  **No. (95% CI)** |
| **Overall** | 187.19 (174.63~196.03) | 8.67 (8.1~9.08) | 435.04 (397.02~479.73) | 9.99 (9.12~11.02) | 0.69 (0.57~0.81) |
| **Socio-demographic factor** | | | | | |
| **High SDI** | 77.5 (74.62~79.41) | 13.82 (13.36~14.15) | 168.02 (148.61~188.55) | 19.16 (16.94~21.48) | 1.37 (1.28~1.46) |
| **High-middle SDI** | 70.52 (67.44~73.69) | 11.66 (11.15~12.19) | 149.13 (133.48~165.01) | 13.87 (12.4~15.37) | 0.8 (0.64~0.97) |
| **Middle SDI** | 25.82 (19.86~29.47) | 4.45 (3.48~5.05) | 78.64 (64.69~92.03) | 5.7 (4.72~6.67) | 1.1 (0.77~1.43) |
| **Low-middle SDI** | 9.85 (8.01~11.8) | 3.09 (2.53~3.71) | 29.42 (25.04~35.62) | 3.94 (3.36~4.8) | 0.73 (0.66~0.79) |
| **Low SDI** | 3.39 (2.69~4.23) | 2.78 (2.2~3.5) | 9.53 (7.77~11.74) | 3.43 (2.81~4.21) | 0.71 (0.65~0.77) |
| **Region** | | | | | |
| **Andean Latin America** | 0.84 (0.67~0.97) | 7.57 (6.06~8.74) | 2.9 (2.28~3.77) | 9.75 (7.65~12.67) | 0.91 (0.77~1.05) |
| **Australasia** | 1.16 (1.08~1.23) | 9.46 (8.88~10.03) | 2.69 (2.18~3.32) | 11.26 (9.13~13.94) | 0.58 (0.45~0.7) |
| **Caribbean** | 1.61 (1.5~1.72) | 11.61 (10.82~12.43) | 4.84 (4.11~5.68) | 17.83 (15.11~20.97) | 1.34 (1.11~1.57) |
| **Central Asia** | 3.05 (2.88~3.24) | 10.77 (10.18~11.44) | 5.46 (4.86~6.11) | 11.72 (10.47~13.11) | 0.36 (0.17~0.54) |
| **Central Europe** | 11.34 (10.91~11.94) | 13.83 (13.32~14.55) | 21.86 (18.94~25.3) | 20.52 (17.68~23.86) | 1.56 (1.47~1.65) |
| **Central Latin America** | 1.89 (1.82~1.96) | 4.1 (3.94~4.26) | 8.37 (7.04~9.92) | 6.4 (5.39~7.58) | 1.54 (1.36~1.71) |
| **Central Sub-Saharan Africa** | 0.37 (0.27~0.52) | 2.82 (2.11~3.96) | 0.92 (0.65~1.31) | 3.01 (2.14~4.32) | 0.2 (0.1~0.3) |
| **East Asia** | 25.44 (18.94~31.28) | 5.17 (3.92~6.33) | 70.92 (55.04~96.59) | 6.55 (5.07~8.8) | 1.35 (0.7~1.99) |
| **Eastern Europe** | 34.57 (33.21~36.19) | 20.42 (19.56~21.44) | 52.51 (44.7~61.83) | 27.5 (23.25~32.58) | 0.91 (0.64~1.19) |
| **Eastern Sub-Saharan Africa** | 1.3 (0.91~1.6) | 3.27 (2.32~4.01) | 3.27 (2.36~4.08) | 3.7 (2.64~4.57) | 0.39 (0.28~0.49) |
| **High-income Asia Pacific** | 7.42 (6.59~7.79) | 6.74 (5.95~7.08) | 18 (14.66~21.64) | 11.32 (9.21~13.68) | 2.46 (2.21~2.7) |
| **High-income North America** | 35.82 (34.36~36.83) | 19.15 (18.49~19.68) | 86.65 (72.24~103.73) | 27.82 (23.11~33.44) | 1.44 (1.34~1.53) |
| **North Africa and Middle East** | 2.84 (2.15~3.4) | 3.1 (2.36~3.73) | 12.51 (8.47~14.86) | 5.41 (3.71~6.39) | 2.14 (1.84~2.43) |
| **Oceania** | 0.11 (0.08~0.14) | 6.75 (4.79~8.38) | 0.34 (0.19~0.46) | 8.58 (4.86~11.31) | 0.86 (0.82~0.89) |
| **South Asia** | 6.02 (4.71~7.59) | 2.17 (1.71~2.74) | 21.83 (17.28~26.69) | 2.94 (2.34~3.61) | 0.89 (0.71~1.06) |
| **Southeast Asia** | 7.14 (4.97~8.35) | 4.7 (3.36~5.46) | 22.13 (14.28~26.65) | 6.23 (4.1~7.47) | 0.9 (0.86~0.94) |
| **Southern Latin America** | 1.77 (1.67~1.88) | 6.97 (6.59~7.36) | 3.64 (2.86~4.59) | 8.23 (6.43~10.38) | 0.32 (0.15~0.49) |
| **Southern Sub-Saharan Africa** | 0.55 (0.45~0.65) | 3.48 (2.82~4.05) | 1.68 (1.19~1.95) | 5.08 (3.57~5.87) | 1.75 (1.55~1.94) |
| **Tropical Latin America** | 2.97 (2.83~3.11) | 5.92 (5.62~6.2) | 9.35 (8.72~10.02) | 6.97 (6.5~7.48) | 0.45 (0.36~0.53) |
| **Western Europe** | 40.04 (38.53~41.25) | 13.14 (12.68~13.52) | 82.5 (70.94~94.18) | 19.62 (16.98~22.47) | 1.71 (1.57~1.86) |
| **Western Sub-Saharan Africa** | 0.93 (0.75~1.36) | 2.13 (1.73~3.16) | 2.68 (2.15~3.6) | 2.64 (2.13~3.58) | 0.83 (0.77~0.89) |

**Note: ASIR:** age-standardized incidence rate
