# Supplementary material for: Epidemiological trends of women’s cancers from 1990 to 2019 at the global, regional, and national levels: a population-based study
Source: Biomark Res. 2021 Jul 7;9:55. doi: 10.1186/s40364-021-00310-y (PMC8261911; doi:10.1186/s40364-021-00310-y)
Supplement: Supplementary file 26 — Additional file 26: Table S11: The death of uterine cancer and temporal trends. [file 40364_2021_310_MOESM26_ESM.docx]

**Table S11: The death of uterine cancer and temporal trends.**

|  | **1990** | | **2019** | | **1990-2019** |
| --- | --- | --- | --- | --- | --- |
|  | **Death cases**  **No *10^3^ (95% UI)** | **ASDR /100,000**  **No. (95% UI)** | **Death cases**  **No *10^3^ (95% UI)** | **ASDR /100,000**  **No. (95% UI)** | **EAPC**  **No. (95% CI)** |
| **Overall** | 56.13 (51.1~60.2) | 2.67 (2.44~2.86) | 91.64 (82.39~101.5) | 2.09 (1.88~2.32) | -0.85 (-0.93~-0.76) |
| **Socio-demographic factor** | | | | | |
| **High SDI** | 16.88 (15.87~17.41) | 2.73 (2.57~2.81) | 26.63 (24~28.14) | 2.52 (2.32~2.64) | -0.12 (-0.2~-0.04) |
| **High-middle SDI** | 19.52 (18.33~20.67) | 3.23 (3.03~3.42) | 26.43 (23.96~28.83) | 2.33 (2.12~2.55) | -1.25 (-1.36~-1.13) |
| **Middle SDI** | 11.81 (9.28~13.51) | 2.22 (1.77~2.52) | 20.95 (17.53~24.33) | 1.61 (1.36~1.87) | -1.01 (-1.27~-0.75) |
| **Low-middle SDI** | 5.57 (4.59~6.75) | 1.93 (1.61~2.36) | 12.25 (10.43~15.28) | 1.75 (1.49~2.21) | -0.48 (-0.57~-0.38) |
| **Low SDI** | 2.32 (1.84~2.93) | 2.08 (1.64~2.65) | 5.3 (4.32~6.64) | 2.1 (1.72~2.63) | -0.01 (-0.05~0.03) |
| **Region** | | | | | |
| **Andean Latin America** | 0.47 (0.38~0.54) | 4.53 (3.67~5.21) | 1.02 (0.81~1.34) | 3.53 (2.8~4.63) | -0.85 (-0.95~-0.74) |
| **Australasia** | 0.35 (0.32~0.36) | 2.62 (2.46~2.77) | 0.65 (0.57~0.72) | 2.4 (2.13~2.65) | -0.34 (-0.4~-0.27) |
| **Caribbean** | 0.61 (0.56~0.67) | 4.54 (4.18~4.99) | 1.56 (1.34~1.82) | 5.68 (4.86~6.62) | 0.8 (0.68~0.93) |
| **Central Asia** | 1.11 (1.05~1.18) | 4.02 (3.8~4.26) | 1.35 (1.21~1.51) | 3.2 (2.88~3.57) | -0.76 (-0.9~-0.63) |
| **Central Europe** | 3.56 (3.42~3.77) | 4.23 (4.05~4.47) | 4.72 (4.1~5.43) | 3.79 (3.28~4.36) | -0.3 (-0.43~-0.18) |
| **Central Latin America** | 0.87 (0.83~0.91) | 2.08 (1.97~2.17) | 2.34 (1.99~2.73) | 1.84 (1.57~2.16) | -0.48 (-0.7~-0.27) |
| **Central Sub-Saharan Africa** | 0.25 (0.19~0.35) | 2.1 (1.59~2.93) | 0.55 (0.39~0.78) | 1.97 (1.42~2.8) | -0.23 (-0.28~-0.18) |
| **East Asia** | 10.89 (8.18~13.3) | 2.35 (1.8~2.86) | 12.93 (10.12~17.99) | 1.19 (0.93~1.65) | -2.16 (-2.74~-1.57) |
| **Eastern Europe** | 8.06 (7.72~8.39) | 4.45 (4.27~4.64) | 8.44 (7.31~9.67) | 3.92 (3.39~4.51) | -0.95 (-1.22~-0.69) |
| **Eastern Sub-Saharan Africa** | 0.9 (0.65~1.12) | 2.48 (1.78~3.09) | 1.92 (1.34~2.38) | 2.41 (1.7~3.01) | -0.12 (-0.2~-0.04) |
| **High-income Asia Pacific** | 2.19 (1.94~2.3) | 1.94 (1.71~2.04) | 3.43 (2.89~3.76) | 1.47 (1.3~1.58) | -0.63 (-0.84~-0.42) |
| **High-income North America** | 6.14 (5.75~6.35) | 2.92 (2.76~3.01) | 11.26 (10.46~11.84) | 3.23 (3.03~3.38) | 0.51 (0.38~0.63) |
| **North Africa and Middle East** | 1.34 (1.02~1.62) | 1.62 (1.25~1.99) | 3.23 (2.35~3.81) | 1.58 (1.17~1.85) | 0.05 (-0.23~0.33) |
| **Oceania** | 0.05 (0.04~0.06) | 3.52 (2.45~4.39) | 0.15 (0.08~0.2) | 4.18 (2.44~5.5) | 0.69 (0.63~0.74) |
| **South Asia** | 3.88 (3.05~4.92) | 1.57 (1.23~2.02) | 10.25 (8.13~13.08) | 1.47 (1.16~1.87) | -0.44 (-0.59~-0.29) |
| **Southeast Asia** | 3.2 (2.34~3.79) | 2.31 (1.73~2.73) | 7.32 (5.13~8.65) | 2.21 (1.6~2.6) | -0.22 (-0.3~-0.14) |
| **Southern Latin America** | 0.77 (0.72~0.81) | 2.99 (2.82~3.15) | 1.14 (1.04~1.25) | 2.4 (2.19~2.62) | -1.01 (-1.16~-0.86) |
| **Southern Sub-Saharan Africa** | 0.31 (0.25~0.37) | 2.09 (1.65~2.48) | 0.87 (0.6~1) | 2.77 (1.92~3.18) | 1.47 (1.17~1.77) |
| **Tropical Latin America** | 1.42 (1.34~1.49) | 3.08 (2.89~3.22) | 3.18 (2.93~3.42) | 2.4 (2.2~2.57) | -0.96 (-1.04~-0.87) |
| **Western Europe** | 9.11 (8.56~9.44) | 2.61 (2.46~2.69) | 13.81 (12.22~14.71) | 2.59 (2.35~2.74) | 0.2 (0.1~0.31) |
| **Western Sub-Saharan Africa** | 0.64 (0.52~0.95) | 1.54 (1.24~2.32) | 1.51 (1.22~2.05) | 1.66 (1.36~2.27) | 0.38 (0.33~0.43) |

**Note: ASDR:** age-standardized death rate
