# Supplementary material for: Epidemiological trends of women’s cancers from 1990 to 2019 at the global, regional, and national levels: a population-based study
Source: Biomark Res. 2021 Jul 7;9:55. doi: 10.1186/s40364-021-00310-y (PMC8261911; doi:10.1186/s40364-021-00310-y)
Supplement: Supplementary file 27 — Additional file 27: Table S12: The Disability-Adjusted Life Year (DALY) of uterine cancer and temporal trends. [file 40364_2021_310_MOESM27_ESM.docx]

**Table S12: The Disability-Adjusted Life Year (DALY) of female uterine cancer and temporal trends.**

|  | **1990** | | **2019** | | **1990-2019** |
| --- | --- | --- | --- | --- | --- |
|  | **DALY**  **No *10^3^ (95% UI)** | **Age-standardized DALY rate /100,000**  **No. (95% UI)** | **DALY**  **No *10^3^ (95% UI)** | **Age-standardized DALY rate /100,000**  **No. (95% UI)** | **EAPC**  **No. (95% CI)** |
| **Overall** | 1483.29 (1317.51~1612.75) | 68.33 (60.86~74.23) | 2329.07 (2092.95~2560.89) | 53.54 (48.13~58.84) | -0.84 (-0.93~-0.75) |
| **Socio-demographic factor** | | | | | |
| **High SDI** | 380.07 (361.66~395.57) | 66.52 (63.07~69.26) | 596.79 (555.85~636.05) | 65.31 (60.99~69.65) | 0.15 (0.04~0.26) |
| **High-middle SDI** | 523.24 (486.39~558.71) | 86.43 (80.17~92.33) | 667.94 (606.21~735.23) | 61.5 (55.81~67.85) | -1.3 (-1.43~-1.17) |
| **Middle SDI** | 354.19 (266.9~409.37) | 60.49 (46.35~69.57) | 584.64 (475.79~674.77) | 42.88 (34.87~49.47) | -1.1 (-1.37~-0.83) |
| **Low-middle SDI** | 159.43 (129.51~193.56) | 49.01 (39.98~59.48) | 332.22 (280.87~404.9) | 44.53 (37.69~54.57) | -0.48 (-0.58~-0.37) |
| **Low SDI** | 65.37 (51.43~82.35) | 51.5 (40.84~65.01) | 145.26 (117.9~181.52) | 51.24 (41.73~64.02) | -0.07 (-0.12~-0.03) |
| **Region** | | | | | |
| **Andean Latin America** | 12.73 (10.19~14.65) | 113.36 (90.87~130.54) | 25.32 (19.72~33.21) | 85.55 (66.72~112.49) | -0.96 (-1.08~-0.85) |
| **Australasia** | 7.7 (7.26~8.11) | 61.71 (58.26~65.17) | 13.8 (12.36~15.23) | 56.3 (50.79~61.96) | -0.33 (-0.4~-0.26) |
| **Caribbean** | 16.65 (15.26~18.31) | 119.52 (109.4~131.55) | 39.69 (33.6~46.79) | 146.98 (124.22~173.54) | 0.7 (0.55~0.84) |
| **Central Asia** | 31.74 (29.97~33.59) | 112.56 (106.46~119.13) | 41.09 (36.62~46.35) | 89.73 (80.18~100.92) | -0.82 (-0.94~-0.71) |
| **Central Europe** | 89.62 (86.04~94.79) | 108.58 (104.28~114.78) | 107.52 (93.14~123.6) | 96.48 (83.29~111.56) | -0.32 (-0.45~-0.19) |
| **Central Latin America** | 23.22 (22.39~24.18) | 50.41 (48.5~52.56) | 61.39 (52.09~72.13) | 47.36 (40.25~55.58) | -0.25 (-0.49~0) |
| **Central Sub-Saharan Africa** | 7.31 (5.45~10.11) | 53.4 (40.06~73.87) | 15.28 (10.88~21.89) | 48.58 (34.5~69.88) | -0.34 (-0.39~-0.3) |
| **East Asia** | 341.16 (244.78~421.71) | 68.79 (50.13~84.6) | 386.02 (306.61~523) | 35.7 (28.39~47.85) | -2.08 (-2.66~-1.5) |
| **Eastern Europe** | 214.45 (205.34~225.44) | 124.15 (118.61~130.58) | 219.2 (190.17~252.53) | 110.79 (95.86~128.31) | -0.93 (-1.22~-0.65) |
| **Eastern Sub-Saharan Africa** | 25.5 (17.95~31.94) | 61.55 (43.74~76.34) | 51.34 (36.53~64.21) | 56.64 (40.16~70.28) | -0.35 (-0.44~-0.26) |
| **High-income Asia Pacific** | 55.83 (47.26~59.08) | 49.96 (42~52.95) | 76.75 (68.87~83.46) | 42.38 (38.85~45.96) | -0.11 (-0.35~0.13) |
| **High-income North America** | 139.75 (132.66~146.46) | 72.79 (69.27~76.36) | 272.49 (254.06~291.03) | 85.62 (79.99~91.48) | 0.75 (0.63~0.87) |
| **North Africa and Middle East** | 37.74 (28.69~45.56) | 40.91 (31.33~49.31) | 89.66 (62.7~107.52) | 39.33 (28.15~46.59) | -0.05 (-0.33~0.23) |
| **Oceania** | 1.59 (1.08~2.01) | 94.6 (65.12~118.8) | 4.56 (2.49~6.18) | 113.23 (63.01~151.27) | 0.73 (0.66~0.8) |
| **South Asia** | 106.45 (83.53~134.19) | 36.89 (29.05~46.71) | 270.46 (213.06~341.84) | 36.23 (28.51~45.91) | -0.26 (-0.41~-0.11) |
| **Southeast Asia** | 96.14 (66.32~115.51) | 63.15 (45.15~75) | 210.94 (137.47~251.35) | 59.84 (39.99~71.06) | -0.26 (-0.36~-0.15) |
| **Southern Latin America** | 18.62 (17.55~19.68) | 72.81 (68.69~76.97) | 25.42 (23.12~27.91) | 56.68 (51.56~62.19) | -1.07 (-1.25~-0.9) |
| **Southern Sub-Saharan Africa** | 7.95 (6.42~9.36) | 49.39 (39.85~58.05) | 20.79 (14.76~24.26) | 62.63 (44.04~73.04) | 1.4 (1.11~1.69) |
| **Tropical Latin America** | 36.15 (34.52~37.74) | 71.87 (68.47~75.08) | 76.61 (71.46~82) | 57.25 (53.41~61.27) | -0.87 (-0.96~-0.78) |
| **Western Europe** | 196.32 (186.5~204.84) | 62.16 (59.26~64.85) | 281.18 (256.98~301.83) | 63.23 (58.28~67.78) | 0.32 (0.22~0.42) |
| **Western Sub-Saharan Africa** | 16.69 (13.46~23.98) | 37.38 (30.15~54.05) | 39.57 (31.68~53.15) | 38.46 (31.19~51.7) | 0.16 (0.12~0.21) |
