# Supplementary material for: Epidemiological trends of women’s cancers from 1990 to 2019 at the global, regional, and national levels: a population-based study
Source: Biomark Res. 2021 Jul 7;9:55. doi: 10.1186/s40364-021-00310-y (PMC8261911; doi:10.1186/s40364-021-00310-y)
Supplement: Supplementary file 28 — Additional file 28: Table S13: The age-standardized rate of women's cancers in 204 countries or territories. [file 40364_2021_310_MOESM28_ESM.pdf]

Table S13: The age-standardized rate of women's cancers in 204 countries or territories.

Measure	Region	Rate	Cancer	Metric	Value
Deaths	Switzerland	Age-standardized	Breast cancer	Rate	17.84650727
Deaths	Switzerland	Age-standardized	Cervical cancer	Rate	2.171463768
Deaths	Switzerland	Age-standardized	Uterine cancer	Rate	1.835799351
Deaths	Denmark	Age-standardized	Breast cancer	Rate	22.78609716
Deaths	Denmark	Age-standardized	Cervical cancer	Rate	3.284950832
Deaths	Denmark	Age-standardized	Uterine cancer	Rate	2.757826493
Deaths	Denmark	Age-standardized	Ovarian cancer	Rate	8.112448938
Deaths	Costa Rica	Age-standardized	Cervical cancer	Rate	7.217049638
Deaths	Costa Rica	Age-standardized	Uterine cancer	Rate	2.447059657
Deaths	Costa Rica	Age-standardized	Ovarian cancer	Rate	3.969312078
Deaths	Dominica	Age-standardized	Breast cancer	Rate	30.76798409
Deaths	Dominica	Age-standardized	Cervical cancer	Rate	17.32926839
Deaths	Dominica	Age-standardized	Uterine cancer	Rate	4.632296822
Deaths	Dominica	Age-standardized	Ovarian cancer	Rate	3.319971624
Deaths	Finland	Age-standardized	Breast cancer	Rate	16.22268875
Deaths	Argentina	Age-standardized	Breast cancer	Rate	27.91723053
Deaths	Argentina	Age-standardized	Cervical cancer	Rate	10.8523371
Deaths	Argentina	Age-standardized	Uterine cancer	Rate	2.582531916
Deaths	Sri Lanka	Age-standardized	Uterine cancer	Rate	1.756279933
Deaths	Sri Lanka	Age-standardized	Ovarian cancer	Rate	3.210482345
Deaths	Hungary	Age-standardized	Cervical cancer	Rate	5.18322138
Deaths	Hungary	Age-standardized	Uterine cancer	Rate	3.24872949
Deaths	Myanmar	Age-standardized	Ovarian cancer	Rate	5.126541459
Deaths	Grenada	Age-standardized	Breast cancer	Rate	30.08152892
Deaths	Grenada	Age-standardized	Cervical cancer	Rate	16.99680252
Deaths	Grenada	Age-standardized	Uterine cancer	Rate	11.30031632
Deaths	Guinea	Age-standardized	Breast cancer	Rate	19.16498781
Deaths	Guinea	Age-standardized	Cervical cancer	Rate	36.15950609
Deaths	Guinea	Age-standardized	Uterine cancer	Rate	2.488234772
Deaths	France	Age-standardized	Breast cancer	Rate	19.94316228
Deaths	France	Age-standardized	Cervical cancer	Rate	2.75099341
Deaths	France	Age-standardized	Uterine cancer	Rate	2.591447015
Deaths	Tokelau	Age-standardized	Breast cancer	Rate	33.67665097
Deaths	Tokelau	Age-standardized	Cervical cancer	Rate	15.89147161
Deaths	Tokelau	Age-standardized	Uterine cancer	Rate	5.551663046
Deaths	Georgia	Age-standardized	Ovarian cancer	Rate	6.519170795
Deaths	Honduras	Age-standardized	Ovarian cancer	Rate	4.233661186
Deaths	Germany	Age-standardized	Breast cancer	Rate	21.03801771
Deaths	Germany	Age-standardized	Cervical cancer	Rate	2.868800102
Deaths	Germany	Age-standardized	Uterine cancer	Rate	2.279226804
Deaths	Guyana	Age-standardized	Breast cancer	Rate	25.1572224
Deaths	Guyana	Age-standardized	Cervical cancer	Rate	21.01409571
Deaths	Guyana	Age-standardized	Uterine cancer	Rate	6.899410244
Deaths	Guyana	Age-standardized	Ovarian cancer	Rate	8.065360932
Deaths	North Macedonia	Age-standardized	Ovarian cancer	Rate	6.933422824
Deaths	Germany	Age-standardized	Ovarian cancer	Rate	6.443472584
Deaths	Philippines	Age-standardized	Breast cancer	Rate	22.84784004
Deaths	Philippines	Age-standardized	Cervical cancer	Rate	6.829867614
Deaths	Philippines	Age-standardized	Uterine cancer	Rate	2.58434344
Deaths	Philippines	Age-standardized	Ovarian cancer	Rate	5.820475121
Deaths	Grenada	Age-standardized	Ovarian cancer	Rate	7.63014339
Deaths	Ukraine	Age-standardized	Breast cancer	Rate	19.77370406
Deaths	Ukraine	Age-standardized	Cervical cancer	Rate	5.234729171
Deaths	Ukraine	Age-standardized	Uterine cancer	Rate	3.886034813
Deaths	Dominican Republic	Age-standardized	Ovarian cancer	Rate	1.700287209
Deaths	Greece	Age-standardized	Ovarian cancer	Rate	6.158459762

Deaths	Gabon	Age-standardized	Breast cancer	Rate	27.67939382
Deaths	Gabon	Age-standardized	Cervical cancer	Rate	16.72741228
Deaths	Gabon	Age-standardized	Uterine cancer	Rate	2.444158627
Deaths	Iraq	Age-standardized	Breast cancer	Rate	21.6388995
Deaths	Iraq	Age-standardized	Cervical cancer	Rate	2.400036938
Deaths	Iraq	Age-standardized	Uterine cancer	Rate	1.915499222
Deaths	Ukraine	Age-standardized	Ovarian cancer	Rate	6.317848969
Deaths	Honduras	Age-standardized	Breast cancer	Rate	13.46534171
Deaths	Honduras	Age-standardized	Cervical cancer	Rate	11.1422268
Deaths	Honduras	Age-standardized	Uterine cancer	Rate	4.33115182
Deaths	Bangladesh	Age-standardized	Ovarian cancer	Rate	3.40416455
Deaths	Guinea	Age-standardized	Ovarian cancer	Rate	4.16233876
Deaths	Sri Lanka	Age-standardized	Breast cancer	Rate	12.13952957
Deaths	Sri Lanka	Age-standardized	Cervical cancer	Rate	3.601752772
Deaths	Poland	Age-standardized	Breast cancer	Rate	19.2001756
Deaths	Poland	Age-standardized	Cervical cancer	Rate	5.948783418
Deaths	Poland	Age-standardized	Uterine cancer	Rate	4.187941805
Deaths	Jordan	Age-standardized	Ovarian cancer	Rate	3.571632265
Deaths	Taiwan (Province of China)	Age-standardized	Breast cancer	Rate	11.57155619
Deaths	Taiwan (Province of China)	Age-standardized	Cervical cancer	Rate	5.001157801
Deaths	Taiwan (Province of China)	Age-standardized	Uterine cancer	Rate	1.640188901
Deaths	Taiwan (Province of China)	Age-standardized	Ovarian cancer	Rate	3.841079726
Deaths	Thailand	Age-standardized	Ovarian cancer	Rate	3.774458145
Deaths	Greece	Age-standardized	Breast cancer	Rate	21.64561221
Deaths	Greece	Age-standardized	Cervical cancer	Rate	2.958011216
Deaths	Greece	Age-standardized	Uterine cancer	Rate	2.667131877
Deaths	Tokelau	Age-standardized	Ovarian cancer	Rate	3.90421693
Deaths	Zambia	Age-standardized	Ovarian cancer	Rate	5.762741842
Deaths	Djibouti	Age-standardized	Ovarian cancer	Rate	5.87482284
Deaths	Ghana	Age-standardized	Breast cancer	Rate	30.3191249
Deaths	Ghana	Age-standardized	Cervical cancer	Rate	16.93832128
Deaths	Ghana	Age-standardized	Uterine cancer	Rate	2.663465487
Deaths	Thailand	Age-standardized	Breast cancer	Rate	12.41038108
Deaths	Thailand	Age-standardized	Cervical cancer	Rate	6.732353515
Deaths	Thailand	Age-standardized	Uterine cancer	Rate	1.128425055
Deaths	Finland	Age-standardized	Cervical cancer	Rate	1.782280419
Deaths	Finland	Age-standardized	Uterine cancer	Rate	3.059610582
Deaths	Finland	Age-standardized	Ovarian cancer	Rate	6.748990188
Deaths	Burundi	Age-standardized	Breast cancer	Rate	16.16204949
Deaths	Burundi	Age-standardized	Cervical cancer	Rate	25.33787821
Deaths	Burundi	Age-standardized	Uterine cancer	Rate	2.441690904
Deaths	Burundi	Age-standardized	Ovarian cancer	Rate	3.702335155
Deaths	Armenia	Age-standardized	Breast cancer	Rate	23.94669154
Deaths	Armenia	Age-standardized	Cervical cancer	Rate	7.093659981
Deaths	Armenia	Age-standardized	Uterine cancer	Rate	3.745445088
Deaths	Armenia	Age-standardized	Ovarian cancer	Rate	5.248921368
Deaths	Montenegro	Age-standardized	Breast cancer	Rate	26.3663292
Deaths	Montenegro	Age-standardized	Cervical cancer	Rate	4.837567031
Deaths	Montenegro	Age-standardized	Uterine cancer	Rate	2.981877249
Deaths	France	Age-standardized	Ovarian cancer	Rate	6.065032023
Deaths	Timor-Leste	Age-standardized	Uterine cancer	Rate	2.830448787
Deaths	Timor-Leste	Age-standardized	Ovarian cancer	Rate	3.610404413
Deaths	Switzerland	Age-standardized	Ovarian cancer	Rate	5.414584994
Deaths	Haiti	Age-standardized	Breast cancer	Rate	28.78741927
Deaths	Haiti	Age-standardized	Cervical cancer	Rate	27.76420641
Deaths	Haiti	Age-standardized	Uterine cancer	Rate	5.800654046
Deaths	El Salvador	Age-standardized	Breast cancer	Rate	10.75942053
Deaths	El Salvador	Age-standardized	Cervical cancer	Rate	15.26290371

Deaths	El Salvador	Age-standardized	Uterine cancer	Rate	2.252332615
Deaths	Dominican Republic	Age-standardized	Breast cancer	Rate	15.90309524
Deaths	Dominican Republic	Age-standardized	Cervical cancer	Rate	13.31250759
Deaths	Dominican Republic	Age-standardized	Uterine cancer	Rate	5.507361695
Deaths	Iraq	Age-standardized	Ovarian cancer	Rate	3.749275198
Deaths	Timor-Leste	Age-standardized	Breast cancer	Rate	16.25730326
Deaths	Timor-Leste	Age-standardized	Cervical cancer	Rate	9.03078111
Deaths	North Macedonia	Age-standardized	Breast cancer	Rate	26.46066957
Deaths	North Macedonia	Age-standardized	Cervical cancer	Rate	6.117320361
Deaths	North Macedonia	Age-standardized	Uterine cancer	Rate	5.141941935
Deaths	Sudan	Age-standardized	Cervical cancer	Rate	3.265785895
Deaths	Sudan	Age-standardized	Uterine cancer	Rate	0.982310315
Deaths	Sudan	Age-standardized	Ovarian cancer	Rate	2.176256717
Deaths	Djibouti	Age-standardized	Breast cancer	Rate	20.0132588
Deaths	Gambia	Age-standardized	Breast cancer	Rate	12.14796221
Deaths	Gambia	Age-standardized	Cervical cancer	Rate	16.69350151
Deaths	Gambia	Age-standardized	Uterine cancer	Rate	1.846627605
Deaths	Gambia	Age-standardized	Ovarian cancer	Rate	3.426276694
Deaths	Azerbaijan	Age-standardized	Breast cancer	Rate	17.79297727
Deaths	Azerbaijan	Age-standardized	Cervical cancer	Rate	6.037520093
Deaths	Azerbaijan	Age-standardized	Uterine cancer	Rate	3.017375614
Deaths	Azerbaijan	Age-standardized	Ovarian cancer	Rate	3.547530464
Deaths	Montenegro	Age-standardized	Ovarian cancer	Rate	5.744561799
Deaths	El Salvador	Age-standardized	Ovarian cancer	Rate	3.948693126
Deaths	Djibouti	Age-standardized	Cervical cancer	Rate	21.76946022
Deaths	Djibouti	Age-standardized	Uterine cancer	Rate	2.99280337
Deaths	Czechia	Age-standardized	Ovarian cancer	Rate	7.422919149
Deaths	Iran (Islamic Republic of)	Age-standardized	Breast cancer	Rate	11.85795357
Deaths	Iran (Islamic Republic of)	Age-standardized	Cervical cancer	Rate	2.063045532
Deaths	Iran (Islamic Republic of)	Age-standardized	Uterine cancer	Rate	0.979372421
Deaths	Democratic People's Republic of Korea	Age-standardized	Breast cancer	Rate	12.99589667
Deaths	Democratic People's Republic of Korea	Age-standardized	Cervical cancer	Rate	8.28113865
Deaths	Democratic People's Republic of Korea	Age-standardized	Uterine cancer	Rate	1.998351136
Deaths	Democratic People's Republic of Korea	Age-standardized	Ovarian cancer	Rate	2.666978463
Deaths	Republic of Korea	Age-standardized	Breast cancer	Rate	8.725512741
Deaths	Republic of Korea	Age-standardized	Cervical cancer	Rate	2.724707489
Deaths	Republic of Korea	Age-standardized	Uterine cancer	Rate	0.770229668
Deaths	Saint Kitts and Nevis	Age-standardized	Breast cancer	Rate	29.99714133
Deaths	Saint Kitts and Nevis	Age-standardized	Cervical cancer	Rate	14.69853961
Deaths	Saint Kitts and Nevis	Age-standardized	Uterine cancer	Rate	6.627519346
Deaths	Saint Kitts and Nevis	Age-standardized	Ovarian cancer	Rate	7.119487147
Deaths	Kuwait	Age-standardized	Breast cancer	Rate	13.01031731
Deaths	Kuwait	Age-standardized	Cervical cancer	Rate	1.762253195
Deaths	Kuwait	Age-standardized	Uterine cancer	Rate	2.213464759
Deaths	Kuwait	Age-standardized	Ovarian cancer	Rate	3.690758339
Deaths	United Kingdom	Age-standardized	Breast cancer	Rate	22.57904825
Deaths	United Kingdom	Age-standardized	Cervical cancer	Rate	2.893690179
Deaths	United Kingdom	Age-standardized	Uterine cancer	Rate	3.227961787
Deaths	Russian Federation	Age-standardized	Ovarian cancer	Rate	6.893617066
Deaths	Hungary	Age-standardized	Breast cancer	Rate	20.06161349
Deaths	Ghana	Age-standardized	Ovarian cancer	Rate	3.715251873
Deaths	Costa Rica	Age-standardized	Breast cancer	Rate	15.46431732
Deaths	Myanmar	Age-standardized	Breast cancer	Rate	16.77997474
Deaths	Jamaica	Age-standardized	Ovarian cancer	Rate	5.471229964
Deaths	Afghanistan	Age-standardized	Breast cancer	Rate	16.46526992
Deaths	Afghanistan	Age-standardized	Cervical cancer	Rate	7.587224657
Deaths	Afghanistan	Age-standardized	Uterine cancer	Rate	1.872858457
Deaths	Poland	Age-standardized	Ovarian cancer	Rate	9.051302344

Deaths	Brunei Darussalam	Age-standardized	Breast cancer	Rate	22.90173343
Deaths	Brunei Darussalam	Age-standardized	Cervical cancer	Rate	10.45980634
Deaths	Brunei Darussalam	Age-standardized	Uterine cancer	Rate	2.812662816
Deaths	Brunei Darussalam	Age-standardized	Ovarian cancer	Rate	10.07653606
Deaths	Cambodia	Age-standardized	Breast cancer	Rate	13.86301781
Deaths	Cambodia	Age-standardized	Cervical cancer	Rate	9.673025555
Deaths	Cambodia	Age-standardized	Uterine cancer	Rate	3.031088091
Deaths	United Kingdom	Age-standardized	Ovarian cancer	Rate	8.001275045
Deaths	Hungary	Age-standardized	Ovarian cancer	Rate	7.381891279
Deaths	Guatemala	Age-standardized	Breast cancer	Rate	10.60969113
Deaths	China	Age-standardized	Ovarian cancer	Rate	2.768822525
Deaths	Uganda	Age-standardized	Breast cancer	Rate	22.41880556
Deaths	Uganda	Age-standardized	Cervical cancer	Rate	24.29390301
Deaths	Uganda	Age-standardized	Uterine cancer	Rate	4.084965453
Deaths	Libya	Age-standardized	Ovarian cancer	Rate	5.137396132
Deaths	Czechia	Age-standardized	Breast cancer	Rate	16.30608298
Deaths	Czechia	Age-standardized	Cervical cancer	Rate	4.348690783
Deaths	Czechia	Age-standardized	Uterine cancer	Rate	3.622140006
Deaths	Uganda	Age-standardized	Ovarian cancer	Rate	6.936961245
Deaths	Liberia	Age-standardized	Breast cancer	Rate	18.76020462
Deaths	San Marino	Age-standardized	Breast cancer	Rate	19.51734187
Deaths	San Marino	Age-standardized	Cervical cancer	Rate	2.347408825
Deaths	San Marino	Age-standardized	Uterine cancer	Rate	0.826591911
Deaths	San Marino	Age-standardized	Ovarian cancer	Rate	4.35609681
Deaths	Eritrea	Age-standardized	Breast cancer	Rate	22.5040746
Deaths	Eritrea	Age-standardized	Cervical cancer	Rate	30.25783157
Deaths	Slovakia	Age-standardized	Breast cancer	Rate	18.73728354
Deaths	Slovakia	Age-standardized	Cervical cancer	Rate	5.187661992
Deaths	Slovakia	Age-standardized	Uterine cancer	Rate	4.513042473
Deaths	Myanmar	Age-standardized	Cervical cancer	Rate	7.727019655
Deaths	Myanmar	Age-standardized	Uterine cancer	Rate	2.623513642
Deaths	Indonesia	Age-standardized	Breast cancer	Rate	20.47290211
Deaths	Indonesia	Age-standardized	Cervical cancer	Rate	7.079926521
Deaths	Indonesia	Age-standardized	Uterine cancer	Rate	2.864262605
Deaths	Indonesia	Age-standardized	Ovarian cancer	Rate	5.07702734
Deaths	Iran (Islamic Republic of)	Age-standardized	Ovarian cancer	Rate	3.031104679
Deaths	Liberia	Age-standardized	Cervical cancer	Rate	20.48819094
Deaths	Liberia	Age-standardized	Uterine cancer	Rate	2.388183939
Deaths	Liberia	Age-standardized	Ovarian cancer	Rate	3.485383713
Deaths	Viet Nam	Age-standardized	Breast cancer	Rate	21.53585864
Deaths	Viet Nam	Age-standardized	Cervical cancer	Rate	8.801855612
Deaths	Viet Nam	Age-standardized	Uterine cancer	Rate	1.306128251
Deaths	Slovakia	Age-standardized	Ovarian cancer	Rate	6.514520983
Deaths	Guatemala	Age-standardized	Cervical cancer	Rate	18.44083736
Deaths	Guatemala	Age-standardized	Uterine cancer	Rate	2.809584807
Deaths	Guatemala	Age-standardized	Ovarian cancer	Rate	3.277217838
Deaths	Argentina	Age-standardized	Ovarian cancer	Rate	5.996782556
Deaths	Nepal	Age-standardized	Ovarian cancer	Rate	4.595725871
Deaths	Romania	Age-standardized	Ovarian cancer	Rate	6.421008068
Deaths	Morocco	Age-standardized	Breast cancer	Rate	24.40817574
Deaths	Morocco	Age-standardized	Cervical cancer	Rate	7.825463337
Deaths	Morocco	Age-standardized	Uterine cancer	Rate	1.714034433
Deaths	Iceland	Age-standardized	Breast cancer	Rate	14.21747802
Deaths	Iceland	Age-standardized	Cervical cancer	Rate	1.90491987
Deaths	Iceland	Age-standardized	Uterine cancer	Rate	1.599175705
Deaths	Jordan	Age-standardized	Breast cancer	Rate	19.88524146
Deaths	Jordan	Age-standardized	Cervical cancer	Rate	2.040605575
Deaths	Jordan	Age-standardized	Uterine cancer	Rate	2.074784719

Deaths	Israel	Age-standardized	Ovarian cancer	Rate	6.13287567
Deaths	Cook Islands	Age-standardized	Breast cancer	Rate	38.23868375
Deaths	Cook Islands	Age-standardized	Cervical cancer	Rate	3.906164256
Deaths	Cook Islands	Age-standardized	Uterine cancer	Rate	1.879608474
Deaths	China	Age-standardized	Breast cancer	Rate	9.016081197
Deaths	China	Age-standardized	Cervical cancer	Rate	5.128984191
Deaths	China	Age-standardized	Uterine cancer	Rate	1.170899806
Deaths	Republic of Korea	Age-standardized	Ovarian cancer	Rate	2.906022149
Deaths	Fiji	Age-standardized	Breast cancer	Rate	37.08389576
Deaths	Fiji	Age-standardized	Cervical cancer	Rate	22.0981588
Deaths	Fiji	Age-standardized	Uterine cancer	Rate	4.700691838
Deaths	Iceland	Age-standardized	Ovarian cancer	Rate	5.435901479
Deaths	Kyrgyzstan	Age-standardized	Breast cancer	Rate	10.7994039
Deaths	Kyrgyzstan	Age-standardized	Cervical cancer	Rate	8.942064464
Deaths	Kyrgyzstan	Age-standardized	Uterine cancer	Rate	2.78669123
Deaths	Kyrgyzstan	Age-standardized	Ovarian cancer	Rate	4.744638123
Deaths	Comoros	Age-standardized	Breast cancer	Rate	19.66397022
Deaths	Comoros	Age-standardized	Cervical cancer	Rate	22.16640055
Deaths	Comoros	Age-standardized	Uterine cancer	Rate	2.881116224
Deaths	Comoros	Age-standardized	Ovarian cancer	Rate	6.454545862
Deaths	Afghanistan	Age-standardized	Ovarian cancer	Rate	2.83450339
Deaths	Eritrea	Age-standardized	Uterine cancer	Rate	3.385026884
Deaths	Eritrea	Age-standardized	Ovarian cancer	Rate	5.217354891
Deaths	Viet Nam	Age-standardized	Ovarian cancer	Rate	4.256578025
Deaths	Lebanon	Age-standardized	Breast cancer	Rate	35.48805051
Deaths	American Samoa	Age-standardized	Breast cancer	Rate	31.76938982
Deaths	American Samoa	Age-standardized	Cervical cancer	Rate	9.587496118
Deaths	Cook Islands	Age-standardized	Ovarian cancer	Rate	2.819958321
Deaths	Haiti	Age-standardized	Ovarian cancer	Rate	3.407751944
Deaths	Bhutan	Age-standardized	Breast cancer	Rate	12.02170417
Deaths	Bhutan	Age-standardized	Cervical cancer	Rate	7.241662728
Deaths	Bhutan	Age-standardized	Uterine cancer	Rate	1.459789303
Deaths	Kenya	Age-standardized	Breast cancer	Rate	17.04735021
Deaths	Kenya	Age-standardized	Cervical cancer	Rate	12.92230054
Deaths	Kenya	Age-standardized	Uterine cancer	Rate	1.448521287
Deaths	Italy	Age-standardized	Breast cancer	Rate	18.46881125
Deaths	Italy	Age-standardized	Cervical cancer	Rate	2.232132925
Deaths	Pakistan	Age-standardized	Breast cancer	Rate	51.93970127
Deaths	Pakistan	Age-standardized	Cervical cancer	Rate	4.553542623
Deaths	Pakistan	Age-standardized	Uterine cancer	Rate	4.857371123
Deaths	Namibia	Age-standardized	Breast cancer	Rate	29.67784561
Deaths	Namibia	Age-standardized	Cervical cancer	Rate	16.38284463
Deaths	Kenya	Age-standardized	Ovarian cancer	Rate	4.669726244
Deaths	Georgia	Age-standardized	Breast cancer	Rate	27.97941027
Deaths	Georgia	Age-standardized	Cervical cancer	Rate	7.751486475
Deaths	Georgia	Age-standardized	Uterine cancer	Rate	5.796448698
Deaths	Tajikistan	Age-standardized	Breast cancer	Rate	15.17498871
Deaths	Tajikistan	Age-standardized	Cervical cancer	Rate	4.679838994
Deaths	Tajikistan	Age-standardized	Uterine cancer	Rate	3.913564587
Deaths	Suriname	Age-standardized	Ovarian cancer	Rate	6.091274219
Deaths	Guinea-Bissau	Age-standardized	Breast cancer	Rate	22.27846228
Deaths	Guinea-Bissau	Age-standardized	Cervical cancer	Rate	29.28376945
Deaths	Guinea-Bissau	Age-standardized	Uterine cancer	Rate	2.902951627
Deaths	Guinea-Bissau	Age-standardized	Ovarian cancer	Rate	3.299650275
Deaths	American Samoa	Age-standardized	Uterine cancer	Rate	10.74137878
Deaths	Uruguay	Age-standardized	Breast cancer	Rate	29.96992838
Deaths	Uruguay	Age-standardized	Cervical cancer	Rate	8.44932119
Deaths	Uruguay	Age-standardized	Uterine cancer	Rate	2.433790445

Deaths	Uruguay	Age-standardized	Ovarian cancer	Rate	6.446010603
Deaths	Ireland	Age-standardized	Breast cancer	Rate	20.51090556
Deaths	Ireland	Age-standardized	Cervical cancer	Rate	2.812871478
Deaths	Ireland	Age-standardized	Uterine cancer	Rate	2.669854454
Deaths	Ireland	Age-standardized	Ovarian cancer	Rate	8.018936107
Deaths	Singapore	Age-standardized	Ovarian cancer	Rate	4.056095028
Deaths	American Samoa	Age-standardized	Ovarian cancer	Rate	10.00060854
Deaths	Nicaragua	Age-standardized	Breast cancer	Rate	12.0684128
Deaths	Nicaragua	Age-standardized	Cervical cancer	Rate	16.73705958
Deaths	Nicaragua	Age-standardized	Uterine cancer	Rate	1.645532674
Deaths	Botswana	Age-standardized	Breast cancer	Rate	28.63248512
Deaths	Botswana	Age-standardized	Cervical cancer	Rate	26.69892504
Deaths	Botswana	Age-standardized	Uterine cancer	Rate	3.665962547
Deaths	Zambia	Age-standardized	Breast cancer	Rate	19.47023809
Deaths	Zambia	Age-standardized	Cervical cancer	Rate	26.41613289
Deaths	Zambia	Age-standardized	Uterine cancer	Rate	3.092779169
Deaths	Mali	Age-standardized	Breast cancer	Rate	16.91627421
Deaths	Mali	Age-standardized	Cervical cancer	Rate	18.41987152
Deaths	Mali	Age-standardized	Uterine cancer	Rate	1.721172497
Deaths	Tuvalu	Age-standardized	Breast cancer	Rate	35.11702098
Deaths	Tuvalu	Age-standardized	Cervical cancer	Rate	16.85162496
Deaths	Tuvalu	Age-standardized	Uterine cancer	Rate	5.470785924
Deaths	Tuvalu	Age-standardized	Ovarian cancer	Rate	4.495814845
Deaths	Mali	Age-standardized	Ovarian cancer	Rate	1.932350405
Deaths	Romania	Age-standardized	Breast cancer	Rate	18.47458536
Deaths	Romania	Age-standardized	Cervical cancer	Rate	10.95874651
Deaths	Romania	Age-standardized	Uterine cancer	Rate	2.89134055
Deaths	Serbia	Age-standardized	Breast cancer	Rate	29.10124091
Deaths	Serbia	Age-standardized	Cervical cancer	Rate	8.534154287
Deaths	Serbia	Age-standardized	Uterine cancer	Rate	4.192776114
Deaths	Lebanon	Age-standardized	Cervical cancer	Rate	2.430910702
Deaths	Lebanon	Age-standardized	Uterine cancer	Rate	2.562867168
Deaths	Lebanon	Age-standardized	Ovarian cancer	Rate	7.500199132
Deaths	Chile	Age-standardized	Ovarian cancer	Rate	4.663012713
Deaths	India	Age-standardized	Breast cancer	Rate	13.66958166
Deaths	India	Age-standardized	Cervical cancer	Rate	7.379379102
Deaths	India	Age-standardized	Uterine cancer	Rate	1.236005009
Deaths	India	Age-standardized	Ovarian cancer	Rate	3.781848409
Deaths	Botswana	Age-standardized	Ovarian cancer	Rate	5.881431584
Deaths	Jamaica	Age-standardized	Breast cancer	Rate	29.31377356
Deaths	Serbia	Age-standardized	Ovarian cancer	Rate	7.928190476
Deaths	Israel	Age-standardized	Breast cancer	Rate	21.47022962
Deaths	Israel	Age-standardized	Cervical cancer	Rate	2.529585051
Deaths	Israel	Age-standardized	Uterine cancer	Rate	2.722881302
Deaths	Japan	Age-standardized	Breast cancer	Rate	10.14253761
Deaths	Japan	Age-standardized	Cervical cancer	Rate	2.780009285
Deaths	Japan	Age-standardized	Uterine cancer	Rate	1.702793384
Deaths	Japan	Age-standardized	Ovarian cancer	Rate	3.676990834
Deaths	Bhutan	Age-standardized	Ovarian cancer	Rate	5.130660314
Deaths	Bangladesh	Age-standardized	Breast cancer	Rate	14.54072507
Deaths	Bangladesh	Age-standardized	Cervical cancer	Rate	5.672332014
Deaths	Bangladesh	Age-standardized	Uterine cancer	Rate	0.927151148
Deaths	Namibia	Age-standardized	Uterine cancer	Rate	2.296529042
Deaths	Belarus	Age-standardized	Breast cancer	Rate	14.00790234
Deaths	Belarus	Age-standardized	Cervical cancer	Rate	5.895556383
Deaths	Belarus	Age-standardized	Uterine cancer	Rate	2.237738719
Deaths	Nicaragua	Age-standardized	Ovarian cancer	Rate	3.670133972
Deaths	Trinidad and Tobago	Age-standardized	Breast cancer	Rate	23.69074233

Deaths	Trinidad and Tobago	Age-standardized	Cervical cancer	Rate	11.55376895
Deaths	Trinidad and Tobago	Age-standardized	Uterine cancer	Rate	6.383031003
Deaths	Jamaica	Age-standardized	Cervical cancer	Rate	15.89674166
Deaths	Jamaica	Age-standardized	Uterine cancer	Rate	7.038747985
Deaths	Chile	Age-standardized	Breast cancer	Rate	13.93371495
Deaths	Chile	Age-standardized	Cervical cancer	Rate	7.188942445
Deaths	Chile	Age-standardized	Uterine cancer	Rate	1.986500491
Deaths	Belarus	Age-standardized	Ovarian cancer	Rate	5.71991815
Deaths	Panama	Age-standardized	Uterine cancer	Rate	2.68191112
Deaths	Saint Vincent and the Grenadines	Age-standardized	Breast cancer	Rate	30.73418266
Deaths	Saint Vincent and the Grenadines	Age-standardized	Cervical cancer	Rate	20.61380681
Deaths	Saint Vincent and the Grenadines	Age-standardized	Uterine cancer	Rate	8.079035634
Deaths	Cambodia	Age-standardized	Ovarian cancer	Rate	5.26245142
Deaths	Mexico	Age-standardized	Breast cancer	Rate	12.48117345
Deaths	Mexico	Age-standardized	Cervical cancer	Rate	9.525403734
Deaths	Mexico	Age-standardized	Uterine cancer	Rate	1.471743403
Deaths	Singapore	Age-standardized	Breast cancer	Rate	12.66282603
Deaths	Singapore	Age-standardized	Cervical cancer	Rate	2.620830724
Deaths	Singapore	Age-standardized	Uterine cancer	Rate	1.919667272
Deaths	Saint Vincent and the Grenadines	Age-standardized	Ovarian cancer	Rate	6.163687511
Deaths	Australia	Age-standardized	Breast cancer	Rate	17.09350551
Deaths	Australia	Age-standardized	Cervical cancer	Rate	2.138781684
Deaths	Australia	Age-standardized	Uterine cancer	Rate	2.276118465
Deaths	Niger	Age-standardized	Breast cancer	Rate	11.16287532
Deaths	Niger	Age-standardized	Cervical cancer	Rate	23.61912921
Deaths	Niger	Age-standardized	Uterine cancer	Rate	2.116601774
Deaths	Qatar	Age-standardized	Breast cancer	Rate	36.9065233
Deaths	Qatar	Age-standardized	Cervical cancer	Rate	5.033796014
Deaths	Qatar	Age-standardized	Uterine cancer	Rate	2.507204893
Deaths	Maldives	Age-standardized	Ovarian cancer	Rate	5.598872212
Deaths	Ethiopia	Age-standardized	Breast cancer	Rate	17.35910086
Deaths	Ethiopia	Age-standardized	Cervical cancer	Rate	16.81822449
Deaths	Ethiopia	Age-standardized	Uterine cancer	Rate	1.856864296
Deaths	Marshall Islands	Age-standardized	Breast cancer	Rate	39.6328254
Deaths	Marshall Islands	Age-standardized	Cervical cancer	Rate	21.42314099
Deaths	Marshall Islands	Age-standardized	Uterine cancer	Rate	6.658310683
Deaths	Niger	Age-standardized	Ovarian cancer	Rate	1.720592204
Deaths	Qatar	Age-standardized	Ovarian cancer	Rate	8.191749891
Deaths	Mauritania	Age-standardized	Breast cancer	Rate	18.10091609
Deaths	Mauritania	Age-standardized	Cervical cancer	Rate	18.06783347
Deaths	Mauritania	Age-standardized	Uterine cancer	Rate	2.666023989
Deaths	Ethiopia	Age-standardized	Ovarian cancer	Rate	4.194989928
Deaths	Libya	Age-standardized	Breast cancer	Rate	17.21732508
Deaths	Libya	Age-standardized	Cervical cancer	Rate	4.019961046
Deaths	Marshall Islands	Age-standardized	Ovarian cancer	Rate	4.959985046
Deaths	United States Virgin Islands	Age-standardized	Breast cancer	Rate	28.08871223
Deaths	United States Virgin Islands	Age-standardized	Cervical cancer	Rate	7.766556509
Deaths	United States Virgin Islands	Age-standardized	Uterine cancer	Rate	4.043475619
Deaths	Sudan	Age-standardized	Breast cancer	Rate	13.11441081
Deaths	Libya	Age-standardized	Uterine cancer	Rate	1.877479439
Deaths	Panama	Age-standardized	Breast cancer	Rate	13.50941218
Deaths	Panama	Age-standardized	Cervical cancer	Rate	10.01850775
Deaths	Bermuda	Age-standardized	Breast cancer	Rate	19.33202054
Deaths	Bermuda	Age-standardized	Cervical cancer	Rate	3.250624694
Deaths	Bermuda	Age-standardized	Uterine cancer	Rate	3.172806242
Deaths	Netherlands	Age-standardized	Breast cancer	Rate	24.13204431
Deaths	Netherlands	Age-standardized	Cervical cancer	Rate	2.252661813
Deaths	Netherlands	Age-standardized	Uterine cancer	Rate	3.027386158

Deaths	Fiji	Age-standardized	Ovarian cancer	Rate	1.930208284
Deaths	Kazakhstan	Age-standardized	Breast cancer	Rate	16.30489815
Deaths	Kazakhstan	Age-standardized	Cervical cancer	Rate	7.645432295
Deaths	Kazakhstan	Age-standardized	Uterine cancer	Rate	3.273936869
Deaths	Kazakhstan	Age-standardized	Ovarian cancer	Rate	6.399166192
Deaths	Australia	Age-standardized	Ovarian cancer	Rate	5.177925175
Deaths	Panama	Age-standardized	Ovarian cancer	Rate	3.912871424
Deaths	United States Virgin Islands	Age-standardized	Ovarian cancer	Rate	9.835831105
Deaths	United States of America	Age-standardized	Ovarian cancer	Rate	6.470711588
Deaths	Italy	Age-standardized	Uterine cancer	Rate	2.241509883
Deaths	Malaysia	Age-standardized	Breast cancer	Rate	25.81352243
Deaths	Malaysia	Age-standardized	Cervical cancer	Rate	9.001530819
Deaths	Malaysia	Age-standardized	Uterine cancer	Rate	2.607403347
Deaths	Malaysia	Age-standardized	Ovarian cancer	Rate	4.768938203
Deaths	Mexico	Age-standardized	Ovarian cancer	Rate	5.214789316
Deaths	Saint Lucia	Age-standardized	Breast cancer	Rate	21.73374047
Deaths	Saint Lucia	Age-standardized	Cervical cancer	Rate	13.50340296
Deaths	Saint Lucia	Age-standardized	Uterine cancer	Rate	4.560100258
Deaths	Saint Lucia	Age-standardized	Ovarian cancer	Rate	6.136216586
Deaths	Bermuda	Age-standardized	Ovarian cancer	Rate	6.313162134
Deaths	Turkmenistan	Age-standardized	Cervical cancer	Rate	7.178267892
Deaths	Turkmenistan	Age-standardized	Uterine cancer	Rate	0.907907323
Deaths	Turkmenistan	Age-standardized	Ovarian cancer	Rate	3.149710769
Deaths	Micronesia (Federated States of)	Age-standardized	Breast cancer	Rate	42.13290467
Deaths	Micronesia (Federated States of)	Age-standardized	Cervical cancer	Rate	19.93962777
Deaths	Micronesia (Federated States of)	Age-standardized	Uterine cancer	Rate	7.018097163
Deaths	Italy	Age-standardized	Ovarian cancer	Rate	5.68538635
Deaths	Mauritius	Age-standardized	Breast cancer	Rate	21.97376525
Deaths	Mauritius	Age-standardized	Cervical cancer	Rate	5.761302356
Deaths	Mauritius	Age-standardized	Uterine cancer	Rate	3.469619319
Deaths	Morocco	Age-standardized	Ovarian cancer	Rate	5.406054054
Deaths	Micronesia (Federated States of)	Age-standardized	Ovarian cancer	Rate	5.650728629
Deaths	Mauritius	Age-standardized	Ovarian cancer	Rate	5.221343963
Deaths	Lesotho	Age-standardized	Breast cancer	Rate	28.36568683
Deaths	Lesotho	Age-standardized	Cervical cancer	Rate	35.95851941
Deaths	Lesotho	Age-standardized	Uterine cancer	Rate	3.912144092
Deaths	Mongolia	Age-standardized	Breast cancer	Rate	9.937911187
Deaths	Mongolia	Age-standardized	Cervical cancer	Rate	12.20368232
Deaths	Mongolia	Age-standardized	Uterine cancer	Rate	2.199321931
Deaths	Mongolia	Age-standardized	Ovarian cancer	Rate	4.365553775
Deaths	Lesotho	Age-standardized	Ovarian cancer	Rate	6.282210241
Deaths	Angola	Age-standardized	Breast cancer	Rate	19.96380301
Deaths	Angola	Age-standardized	Cervical cancer	Rate	19.84592409
Deaths	Angola	Age-standardized	Uterine cancer	Rate	1.986230133
Deaths	New Zealand	Age-standardized	Ovarian cancer	Rate	5.862791058
Deaths	Lao People's Democratic Republic	Age-standardized	Breast cancer	Rate	20.78919699
Deaths	Mauritania	Age-standardized	Ovarian cancer	Rate	3.853314366
Deaths	South Africa	Age-standardized	Breast cancer	Rate	20.47879794
Deaths	South Africa	Age-standardized	Cervical cancer	Rate	16.63709854
Deaths	South Africa	Age-standardized	Uterine cancer	Rate	2.300342358
Deaths	South Africa	Age-standardized	Ovarian cancer	Rate	4.7314735
Deaths	Lao People's Democratic Republic	Age-standardized	Cervical cancer	Rate	8.978225275
Deaths	Lao People's Democratic Republic	Age-standardized	Uterine cancer	Rate	3.021866834
Deaths	Lao People's Democratic Republic	Age-standardized	Ovarian cancer	Rate	5.153013222
Deaths	Zimbabwe	Age-standardized	Breast cancer	Rate	26.92724592
Deaths	Zimbabwe	Age-standardized	Cervical cancer	Rate	31.39109591
Deaths	Zimbabwe	Age-standardized	Uterine cancer	Rate	5.491603778
Deaths	South Sudan	Age-standardized	Breast cancer	Rate	13.12414004

Deaths	South Sudan	Age-standardized	Cervical cancer	Rate	18.21995085
Deaths	South Sudan	Age-standardized	Uterine cancer	Rate	2.46272986
Deaths	Madagascar	Age-standardized	Ovarian cancer	Rate	3.906240377
Deaths	Suriname	Age-standardized	Breast cancer	Rate	19.02851382
Deaths	Suriname	Age-standardized	Cervical cancer	Rate	16.8140779
Deaths	Suriname	Age-standardized	Uterine cancer	Rate	2.654563934
Deaths	Namibia	Age-standardized	Ovarian cancer	Rate	3.743169716
Deaths	Nepal	Age-standardized	Breast cancer	Rate	18.08324112
Deaths	Nepal	Age-standardized	Cervical cancer	Rate	8.146062369
Deaths	Nepal	Age-standardized	Uterine cancer	Rate	1.335442159
Deaths	Slovenia	Age-standardized	Ovarian cancer	Rate	6.738471548
Deaths	Kiribati	Age-standardized	Ovarian cancer	Rate	2.530597805
Deaths	Lithuania	Age-standardized	Ovarian cancer	Rate	9.092064322
Deaths	Canada	Age-standardized	Ovarian cancer	Rate	5.863127463
Deaths	Greenland	Age-standardized	Ovarian cancer	Rate	9.505849381
Deaths	Ecuador	Age-standardized	Breast cancer	Rate	13.05967992
Deaths	Ecuador	Age-standardized	Cervical cancer	Rate	13.30330572
Deaths	Ecuador	Age-standardized	Uterine cancer	Rate	3.967660026
Deaths	Tunisia	Age-standardized	Breast cancer	Rate	15.73428823
Deaths	Tunisia	Age-standardized	Cervical cancer	Rate	2.568287926
Deaths	Tunisia	Age-standardized	Uterine cancer	Rate	1.13325652
Deaths	Kiribati	Age-standardized	Breast cancer	Rate	34.617423
Deaths	Kiribati	Age-standardized	Cervical cancer	Rate	69.51576467
Deaths	Kiribati	Age-standardized	Uterine cancer	Rate	5.064686066
Deaths	Greenland	Age-standardized	Breast cancer	Rate	19.31780412
Deaths	Greenland	Age-standardized	Cervical cancer	Rate	9.098340622
Deaths	Greenland	Age-standardized	Uterine cancer	Rate	1.310775091
Deaths	South Sudan	Age-standardized	Ovarian cancer	Rate	3.871687656
Deaths	Malawi	Age-standardized	Ovarian cancer	Rate	4.042382661
Deaths	Senegal	Age-standardized	Ovarian cancer	Rate	3.249021125
Deaths	Tunisia	Age-standardized	Ovarian cancer	Rate	3.491840573
Deaths	Brazil	Age-standardized	Breast cancer	Rate	15.12534521
Deaths	Brazil	Age-standardized	Cervical cancer	Rate	8.507448896
Deaths	Brazil	Age-standardized	Uterine cancer	Rate	2.3788519
Deaths	Pakistan	Age-standardized	Ovarian cancer	Rate	11.83819887
Deaths	United States of America	Age-standardized	Breast cancer	Rate	18.39772832
Deaths	United States of America	Age-standardized	Cervical cancer	Rate	3.04875179
Deaths	United States of America	Age-standardized	Uterine cancer	Rate	3.303627822
Deaths	Albania	Age-standardized	Ovarian cancer	Rate	2.837905492
Deaths	Malawi	Age-standardized	Breast cancer	Rate	17.24695776
Deaths	Solomon Islands	Age-standardized	Breast cancer	Rate	75.04148729
Deaths	Solomon Islands	Age-standardized	Cervical cancer	Rate	29.43591455
Deaths	Solomon Islands	Age-standardized	Uterine cancer	Rate	7.530505134
Deaths	Oman	Age-standardized	Breast cancer	Rate	15.88486043
Deaths	Oman	Age-standardized	Cervical cancer	Rate	3.103042868
Deaths	Oman	Age-standardized	Uterine cancer	Rate	0.952841579
Deaths	Slovenia	Age-standardized	Breast cancer	Rate	17.74084853
Deaths	Slovenia	Age-standardized	Cervical cancer	Rate	2.889720694
Deaths	Slovenia	Age-standardized	Uterine cancer	Rate	3.001988575
Deaths	Nigeria	Age-standardized	Breast cancer	Rate	26.98453538
Deaths	Nigeria	Age-standardized	Cervical cancer	Rate	12.0779963
Deaths	Nigeria	Age-standardized	Uterine cancer	Rate	0.796355559
Deaths	Nigeria	Age-standardized	Ovarian cancer	Rate	3.348987251
Deaths	Zimbabwe	Age-standardized	Ovarian cancer	Rate	7.46585795
Deaths	Republic of Moldova	Age-standardized	Breast cancer	Rate	15.3102002
Deaths	Samoa	Age-standardized	Ovarian cancer	Rate	8.482993507
Deaths	Palestine	Age-standardized	Breast cancer	Rate	25.4831903
Deaths	Palestine	Age-standardized	Cervical cancer	Rate	2.868601238

Deaths	Palestine	Age-standardized	Uterine cancer	Rate	4.073673028
Deaths	Canada	Age-standardized	Breast cancer	Rate	18.00095907
Deaths	Canada	Age-standardized	Cervical cancer	Rate	2.443786986
Deaths	Canada	Age-standardized	Uterine cancer	Rate	2.631564256
Deaths	Malawi	Age-standardized	Cervical cancer	Rate	25.60994821
Deaths	Malawi	Age-standardized	Uterine cancer	Rate	1.42529075
Deaths	Palestine	Age-standardized	Ovarian cancer	Rate	4.574060199
Deaths	Seychelles	Age-standardized	Ovarian cancer	Rate	8.866528224
Deaths	Solomon Islands	Age-standardized	Ovarian cancer	Rate	3.78549695
Deaths	Venezuela (Bolivarian Republic of)	Age-standardized	Breast cancer	Rate	17.6515814
Deaths	Venezuela (Bolivarian Republic of)	Age-standardized	Cervical cancer	Rate	15.18082507
Deaths	Venezuela (Bolivarian Republic of)	Age-standardized	Uterine cancer	Rate	2.754128879
Deaths	Venezuela (Bolivarian Republic of)	Age-standardized	Ovarian cancer	Rate	4.528571869
Deaths	Spain	Age-standardized	Breast cancer	Rate	15.15857387
Deaths	Spain	Age-standardized	Cervical cancer	Rate	2.385433539
Deaths	Spain	Age-standardized	Uterine cancer	Rate	2.81423705
Deaths	Bosnia and Herzegovina	Age-standardized	Breast cancer	Rate	20.85446376
Deaths	Bosnia and Herzegovina	Age-standardized	Cervical cancer	Rate	5.611090305
Deaths	Bosnia and Herzegovina	Age-standardized	Uterine cancer	Rate	3.29984098
Deaths	Luxembourg	Age-standardized	Breast cancer	Rate	19.15546662
Deaths	Luxembourg	Age-standardized	Cervical cancer	Rate	1.940144219
Deaths	Luxembourg	Age-standardized	Uterine cancer	Rate	3.013273254
Deaths	Luxembourg	Age-standardized	Ovarian cancer	Rate	7.508800715
Deaths	Sao Tome and Principe	Age-standardized	Breast cancer	Rate	20.81689276
Deaths	Sao Tome and Principe	Age-standardized	Cervical cancer	Rate	26.48438896
Deaths	Netherlands	Age-standardized	Ovarian cancer	Rate	7.394099752
Deaths	Sao Tome and Principe	Age-standardized	Ovarian cancer	Rate	6.764293758
Deaths	Belgium	Age-standardized	Breast cancer	Rate	23.08806384
Deaths	Belgium	Age-standardized	Cervical cancer	Rate	2.693204737
Deaths	Belgium	Age-standardized	Uterine cancer	Rate	2.700913921
Deaths	Malta	Age-standardized	Breast cancer	Rate	20.5550238
Deaths	Malta	Age-standardized	Cervical cancer	Rate	1.782594208
Deaths	Malta	Age-standardized	Uterine cancer	Rate	2.607193844
Deaths	Malta	Age-standardized	Ovarian cancer	Rate	6.938434679
Deaths	Oman	Age-standardized	Ovarian cancer	Rate	4.592674944
Deaths	Uzbekistan	Age-standardized	Breast cancer	Rate	17.42638653
Deaths	Uzbekistan	Age-standardized	Cervical cancer	Rate	8.446072494
Deaths	Uzbekistan	Age-standardized	Uterine cancer	Rate	2.622493221
Deaths	Uzbekistan	Age-standardized	Ovarian cancer	Rate	3.733226795
Deaths	Guam	Age-standardized	Breast cancer	Rate	17.52332956
Deaths	Guam	Age-standardized	Cervical cancer	Rate	5.939883228
Deaths	Guam	Age-standardized	Uterine cancer	Rate	3.046774349
Deaths	Andorra	Age-standardized	Breast cancer	Rate	17.25334011
Deaths	Andorra	Age-standardized	Cervical cancer	Rate	4.095207527
Deaths	Andorra	Age-standardized	Uterine cancer	Rate	2.840643805
Deaths	Guam	Age-standardized	Ovarian cancer	Rate	3.900758866
Deaths	Bahamas	Age-standardized	Breast cancer	Rate	35.94417498
Deaths	Bahamas	Age-standardized	Cervical cancer	Rate	10.85385292
Deaths	Bahamas	Age-standardized	Uterine cancer	Rate	5.242581437
Deaths	Madagascar	Age-standardized	Breast cancer	Rate	16.80044283
Deaths	Madagascar	Age-standardized	Cervical cancer	Rate	21.86731164
Deaths	Madagascar	Age-standardized	Uterine cancer	Rate	2.412470908
Deaths	Bolivia (Plurinational State of)	Age-standardized	Breast cancer	Rate	18.97689064
Deaths	Bolivia (Plurinational State of)	Age-standardized	Cervical cancer	Rate	24.13785314
Deaths	Bolivia (Plurinational State of)	Age-standardized	Uterine cancer	Rate	5.4035324
Deaths	Trinidad and Tobago	Age-standardized	Ovarian cancer	Rate	6.63160277
Deaths	Brazil	Age-standardized	Ovarian cancer	Rate	4.098355513
Deaths	Andorra	Age-standardized	Ovarian cancer	Rate	4.501967712

Deaths	Spain	Age-standardized	Ovarian cancer	Rate	4.739023597
Deaths	Bahamas	Age-standardized	Ovarian cancer	Rate	7.336618842
Deaths	Democratic Republic of the Congo	Age-standardized	Breast cancer	Rate	22.52189669
Deaths	Democratic Republic of the Congo	Age-standardized	Cervical cancer	Rate	21.83650161
Deaths	Democratic Republic of the Congo	Age-standardized	Uterine cancer	Rate	1.885818987
Deaths	Nauru	Age-standardized	Ovarian cancer	Rate	4.802651336
Deaths	Bolivia (Plurinational State of)	Age-standardized	Ovarian cancer	Rate	4.599008108
Deaths	New Zealand	Age-standardized	Breast cancer	Rate	19.42867534
Deaths	New Zealand	Age-standardized	Cervical cancer	Rate	2.353144898
Deaths	New Zealand	Age-standardized	Uterine cancer	Rate	3.070871865
Deaths	Lithuania	Age-standardized	Breast cancer	Rate	16.6591386
Deaths	Lithuania	Age-standardized	Cervical cancer	Rate	5.407222266
Deaths	Lithuania	Age-standardized	Uterine cancer	Rate	4.125601982
Deaths	Democratic Republic of the Congo	Age-standardized	Ovarian cancer	Rate	2.427240804
Deaths	Maldives	Age-standardized	Breast cancer	Rate	13.64860159
Deaths	Maldives	Age-standardized	Cervical cancer	Rate	4.139869656
Deaths	Maldives	Age-standardized	Uterine cancer	Rate	1.167839963
Deaths	Norway	Age-standardized	Breast cancer	Rate	15.30039648
Deaths	Norway	Age-standardized	Cervical cancer	Rate	2.79605655
Deaths	Norway	Age-standardized	Uterine cancer	Rate	2.726699569
Deaths	Eswatini	Age-standardized	Breast cancer	Rate	23.02591894
Deaths	Eswatini	Age-standardized	Cervical cancer	Rate	26.84881758
Deaths	Eswatini	Age-standardized	Uterine cancer	Rate	3.375256133
Deaths	Eswatini	Age-standardized	Ovarian cancer	Rate	6.325380547
Deaths	Sao Tome and Principe	Age-standardized	Uterine cancer	Rate	4.790221399
Deaths	Senegal	Age-standardized	Breast cancer	Rate	21.28476578
Deaths	Senegal	Age-standardized	Cervical cancer	Rate	20.08928745
Deaths	Senegal	Age-standardized	Uterine cancer	Rate	2.467107432
Deaths	Tajikistan	Age-standardized	Ovarian cancer	Rate	4.224092711
Deaths	Angola	Age-standardized	Ovarian cancer	Rate	2.618215006
Deaths	Papua New Guinea	Age-standardized	Ovarian cancer	Rate	2.615075915
Deaths	Central African Republic	Age-standardized	Ovarian cancer	Rate	2.242134954
Deaths	Latvia	Age-standardized	Breast cancer	Rate	18.51743672
Deaths	Latvia	Age-standardized	Cervical cancer	Rate	4.459000092
Deaths	Latvia	Age-standardized	Uterine cancer	Rate	4.464307919
Deaths	Monaco	Age-standardized	Ovarian cancer	Rate	13.66999442
Deaths	Rwanda	Age-standardized	Ovarian cancer	Rate	6.191338347
Deaths	Paraguay	Age-standardized	Ovarian cancer	Rate	3.770627323
Deaths	Bahrain	Age-standardized	Ovarian cancer	Rate	7.793608229
Deaths	Antigua and Barbuda	Age-standardized	Breast cancer	Rate	30.15373362
Deaths	Antigua and Barbuda	Age-standardized	Cervical cancer	Rate	10.81102085
Deaths	Antigua and Barbuda	Age-standardized	Uterine cancer	Rate	5.42760386
Deaths	Antigua and Barbuda	Age-standardized	Ovarian cancer	Rate	6.727222387
Deaths	Latvia	Age-standardized	Ovarian cancer	Rate	9.222248194
Deaths	Samoa	Age-standardized	Breast cancer	Rate	24.45329356
Deaths	Samoa	Age-standardized	Cervical cancer	Rate	12.36122638
Deaths	Samoa	Age-standardized	Uterine cancer	Rate	4.585856048
Deaths	Paraguay	Age-standardized	Breast cancer	Rate	18.04317749
Deaths	Paraguay	Age-standardized	Cervical cancer	Rate	16.69168735
Deaths	Paraguay	Age-standardized	Uterine cancer	Rate	3.191816015
Deaths	Monaco	Age-standardized	Breast cancer	Rate	32.09720105
Deaths	Monaco	Age-standardized	Cervical cancer	Rate	2.690815126
Deaths	Monaco	Age-standardized	Uterine cancer	Rate	1.282089652
Deaths	Bulgaria	Age-standardized	Breast cancer	Rate	23.04735431
Deaths	Bulgaria	Age-standardized	Cervical cancer	Rate	8.248208478
Deaths	Bulgaria	Age-standardized	Uterine cancer	Rate	5.104698422
Deaths	Bulgaria	Age-standardized	Ovarian cancer	Rate	7.561316299
Deaths	Barbados	Age-standardized	Breast cancer	Rate	36.06441862

Deaths	Benin	Age-standardized	Breast cancer	Rate	15.77249971
Deaths	Turkmenistan	Age-standardized	Breast cancer	Rate	12.54237613
Deaths	Rwanda	Age-standardized	Breast cancer	Rate	19.96484467
Deaths	Rwanda	Age-standardized	Cervical cancer	Rate	20.62315138
Deaths	Rwanda	Age-standardized	Uterine cancer	Rate	2.532980607
Deaths	Egypt	Age-standardized	Breast cancer	Rate	14.23043169
Deaths	Peru	Age-standardized	Ovarian cancer	Rate	4.56450429
Deaths	Central African Republic	Age-standardized	Breast cancer	Rate	20.22772058
Deaths	Central African Republic	Age-standardized	Cervical cancer	Rate	29.30827824
Deaths	Central African Republic	Age-standardized	Uterine cancer	Rate	2.204367835
Deaths	Belgium	Age-standardized	Ovarian cancer	Rate	6.339600118
Deaths	Yemen	Age-standardized	Ovarian cancer	Rate	1.941903667
Deaths	Saudi Arabia	Age-standardized	Breast cancer	Rate	14.34532098
Deaths	Saudi Arabia	Age-standardized	Cervical cancer	Rate	2.349227591
Deaths	Saudi Arabia	Age-standardized	Uterine cancer	Rate	1.58810505
Deaths	Niue	Age-standardized	Breast cancer	Rate	30.05561442
Deaths	Niue	Age-standardized	Cervical cancer	Rate	10.89721653
Deaths	Niue	Age-standardized	Uterine cancer	Rate	4.596768668
Deaths	Niue	Age-standardized	Ovarian cancer	Rate	4.936599453
Deaths	Papua New Guinea	Age-standardized	Breast cancer	Rate	43.46759847
Deaths	Papua New Guinea	Age-standardized	Cervical cancer	Rate	14.19568246
Deaths	Papua New Guinea	Age-standardized	Uterine cancer	Rate	3.660566249
Deaths	Burkina Faso	Age-standardized	Breast cancer	Rate	20.54164025
Deaths	Burkina Faso	Age-standardized	Cervical cancer	Rate	22.75976802
Deaths	Burkina Faso	Age-standardized	Uterine cancer	Rate	2.381647056
Deaths	Norway	Age-standardized	Ovarian cancer	Rate	7.832449347
Deaths	Colombia	Age-standardized	Breast cancer	Rate	11.70700037
Deaths	Colombia	Age-standardized	Cervical cancer	Rate	8.411021053
Deaths	Colombia	Age-standardized	Uterine cancer	Rate	1.53547556
Deaths	Estonia	Age-standardized	Breast cancer	Rate	17.18947406
Deaths	Togo	Age-standardized	Breast cancer	Rate	17.5897167
Deaths	Togo	Age-standardized	Cervical cancer	Rate	20.09666548
Deaths	Togo	Age-standardized	Uterine cancer	Rate	2.259832685
Deaths	Saudi Arabia	Age-standardized	Ovarian cancer	Rate	3.908945111
Deaths	Ecuador	Age-standardized	Ovarian cancer	Rate	4.811664876
Deaths	Palau	Age-standardized	Ovarian cancer	Rate	2.865192259
Deaths	Estonia	Age-standardized	Cervical cancer	Rate	5.076643142
Deaths	Estonia	Age-standardized	Uterine cancer	Rate	3.062132548
Deaths	Estonia	Age-standardized	Ovarian cancer	Rate	7.378946814
Deaths	Burkina Faso	Age-standardized	Ovarian cancer	Rate	2.572406842
Deaths	Togo	Age-standardized	Ovarian cancer	Rate	2.97622121
Deaths	Austria	Age-standardized	Breast cancer	Rate	17.55902847
Deaths	Austria	Age-standardized	Cervical cancer	Rate	2.657163298
Deaths	Austria	Age-standardized	Uterine cancer	Rate	2.171799025
Deaths	Austria	Age-standardized	Ovarian cancer	Rate	5.953324977
Deaths	Cyprus	Age-standardized	Ovarian cancer	Rate	6.394693211
Deaths	Sierra Leone	Age-standardized	Breast cancer	Rate	17.79633068
Deaths	Sierra Leone	Age-standardized	Cervical cancer	Rate	22.45289554
Deaths	Sierra Leone	Age-standardized	Uterine cancer	Rate	2.36700265
Deaths	Sierra Leone	Age-standardized	Ovarian cancer	Rate	2.844058171
Deaths	Algeria	Age-standardized	Ovarian cancer	Rate	2.693864218
Deaths	Algeria	Age-standardized	Breast cancer	Rate	13.85902034
Deaths	Algeria	Age-standardized	Cervical cancer	Rate	4.522771804
Deaths	Algeria	Age-standardized	Uterine cancer	Rate	0.749184803
Deaths	Egypt	Age-standardized	Cervical cancer	Rate	1.774393801
Deaths	Egypt	Age-standardized	Uterine cancer	Rate	1.636865904
Deaths	Egypt	Age-standardized	Ovarian cancer	Rate	2.17969442
Deaths	Congo	Age-standardized	Breast cancer	Rate	29.87549342

Deaths	Congo	Age-standardized	Cervical cancer	Rate	23.80127029
Deaths	Congo	Age-standardized	Uterine cancer	Rate	2.749123589
Deaths	Congo	Age-standardized	Ovarian cancer	Rate	3.85546449
Deaths	Bosnia and Herzegovina	Age-standardized	Ovarian cancer	Rate	7.424690182
Deaths	Puerto Rico	Age-standardized	Breast cancer	Rate	16.93248446
Deaths	Puerto Rico	Age-standardized	Cervical cancer	Rate	3.768985706
Deaths	Puerto Rico	Age-standardized	Uterine cancer	Rate	2.670813175
Deaths	Colombia	Age-standardized	Ovarian cancer	Rate	4.651183673
Deaths	Vanuatu	Age-standardized	Ovarian cancer	Rate	2.992742041
Deaths	Portugal	Age-standardized	Breast cancer	Rate	16.44477857
Deaths	Portugal	Age-standardized	Cervical cancer	Rate	3.173641699
Deaths	Portugal	Age-standardized	Uterine cancer	Rate	2.688229649
Deaths	Mozambique	Age-standardized	Breast cancer	Rate	21.47621112
Deaths	Mozambique	Age-standardized	Cervical cancer	Rate	28.76217629
Deaths	Mozambique	Age-standardized	Uterine cancer	Rate	2.809657476
Deaths	Mozambique	Age-standardized	Ovarian cancer	Rate	4.81362076
Deaths	Peru	Age-standardized	Breast cancer	Rate	10.79376699
Deaths	Peru	Age-standardized	Cervical cancer	Rate	12.23180312
Deaths	Peru	Age-standardized	Uterine cancer	Rate	2.841644542
Deaths	Syrian Arab Republic	Age-standardized	Cervical cancer	Rate	1.779211413
Deaths	Syrian Arab Republic	Age-standardized	Uterine cancer	Rate	1.108692329
Deaths	Barbados	Age-standardized	Cervical cancer	Rate	13.10574601
Deaths	Barbados	Age-standardized	Uterine cancer	Rate	7.550950548
Deaths	Barbados	Age-standardized	Ovarian cancer	Rate	6.554300901
Deaths	Tonga	Age-standardized	Breast cancer	Rate	32.34017374
Deaths	Tonga	Age-standardized	Cervical cancer	Rate	17.3973164
Deaths	Tonga	Age-standardized	Uterine cancer	Rate	3.597039476
Deaths	Portugal	Age-standardized	Ovarian cancer	Rate	3.883858333
Deaths	Cuba	Age-standardized	Breast cancer	Rate	17.51747879
Deaths	Cuba	Age-standardized	Cervical cancer	Rate	7.599480356
Deaths	Cuba	Age-standardized	Uterine cancer	Rate	6.478470194
Deaths	Cuba	Age-standardized	Ovarian cancer	Rate	3.710359586
Deaths	Syrian Arab Republic	Age-standardized	Breast cancer	Rate	11.28555475
Deaths	Cyprus	Age-standardized	Breast cancer	Rate	22.13707272
Deaths	Cyprus	Age-standardized	Cervical cancer	Rate	2.89130336
Deaths	Cyprus	Age-standardized	Uterine cancer	Rate	3.051792019
Deaths	Benin	Age-standardized	Cervical cancer	Rate	20.12079588
Deaths	Benin	Age-standardized	Uterine cancer	Rate	2.330093079
Deaths	Benin	Age-standardized	Ovarian cancer	Rate	2.631760357
Deaths	Syrian Arab Republic	Age-standardized	Ovarian cancer	Rate	2.290528235
Deaths	Republic of Moldova	Age-standardized	Cervical cancer	Rate	6.364486782
Deaths	Republic of Moldova	Age-standardized	Uterine cancer	Rate	2.952369075
Deaths	Tonga	Age-standardized	Ovarian cancer	Rate	4.154833249
Deaths	Chad	Age-standardized	Ovarian cancer	Rate	1.826201229
Deaths	Belize	Age-standardized	Breast cancer	Rate	11.86340593
Deaths	Belize	Age-standardized	Cervical cancer	Rate	17.66139873
Deaths	Belize	Age-standardized	Uterine cancer	Rate	4.629438012
Deaths	Belize	Age-standardized	Ovarian cancer	Rate	2.832196449
Deaths	Republic of Moldova	Age-standardized	Ovarian cancer	Rate	4.488792867
Deaths	Seychelles	Age-standardized	Breast cancer	Rate	24.99884232
Deaths	Seychelles	Age-standardized	Cervical cancer	Rate	15.99190959
Deaths	Seychelles	Age-standardized	Uterine cancer	Rate	3.04512272
Deaths	Turkey	Age-standardized	Breast cancer	Rate	12.64835466
Deaths	Turkey	Age-standardized	Cervical cancer	Rate	2.531200941
Deaths	Turkey	Age-standardized	Uterine cancer	Rate	2.216754535
Deaths	Turkey	Age-standardized	Ovarian cancer	Rate	4.418760621
Deaths	Sweden	Age-standardized	Breast cancer	Rate	17.7663341
Deaths	Sweden	Age-standardized	Cervical cancer	Rate	2.715740147

Deaths	Sweden	Age-standardized	Uterine cancer	Rate	2.525344638
Deaths	Puerto Rico	Age-standardized	Ovarian cancer	Rate	4.234534825
Deaths	Nauru	Age-standardized	Breast cancer	Rate	41.37077827
Deaths	Nauru	Age-standardized	Cervical cancer	Rate	19.09336254
Deaths	Nauru	Age-standardized	Uterine cancer	Rate	6.883333275
Deaths	Equatorial Guinea	Age-standardized	Breast cancer	Rate	26.71993076
Deaths	Equatorial Guinea	Age-standardized	Cervical cancer	Rate	17.0900016
Deaths	Equatorial Guinea	Age-standardized	Uterine cancer	Rate	2.342996188
Deaths	Equatorial Guinea	Age-standardized	Ovarian cancer	Rate	3.924297962
Deaths	Russian Federation	Age-standardized	Breast cancer	Rate	17.0196524
Deaths	Russian Federation	Age-standardized	Cervical cancer	Rate	5.598447627
Deaths	Russian Federation	Age-standardized	Uterine cancer	Rate	4.058994146
Deaths	Bahrain	Age-standardized	Breast cancer	Rate	25.16373583
Deaths	Bahrain	Age-standardized	Cervical cancer	Rate	2.961822298
Deaths	Bahrain	Age-standardized	Uterine cancer	Rate	2.280181944
Deaths	Albania	Age-standardized	Breast cancer	Rate	11.94276478
Deaths	Albania	Age-standardized	Cervical cancer	Rate	3.125198052
Deaths	Albania	Age-standardized	Uterine cancer	Rate	2.023246674
Deaths	Vanuatu	Age-standardized	Breast cancer	Rate	27.23057498
Deaths	Vanuatu	Age-standardized	Cervical cancer	Rate	17.07365571
Deaths	Vanuatu	Age-standardized	Uterine cancer	Rate	5.069534508
Deaths	Northern Mariana Islands	Age-standardized	Breast cancer	Rate	27.54830448
Deaths	Northern Mariana Islands	Age-standardized	Cervical cancer	Rate	15.63842607
Deaths	Northern Mariana Islands	Age-standardized	Uterine cancer	Rate	7.662736744
Deaths	Northern Mariana Islands	Age-standardized	Ovarian cancer	Rate	4.576378804
Deaths	Cabo Verde	Age-standardized	Breast cancer	Rate	15.38260291
Deaths	Cabo Verde	Age-standardized	Cervical cancer	Rate	13.86366684
Deaths	Cabo Verde	Age-standardized	Uterine cancer	Rate	3.385413627
Deaths	United Republic of Tanzania	Age-standardized	Breast cancer	Rate	17.84387371
Deaths	United Republic of Tanzania	Age-standardized	Cervical cancer	Rate	22.51170914
Deaths	United Republic of Tanzania	Age-standardized	Uterine cancer	Rate	2.828387784
Deaths	Sweden	Age-standardized	Ovarian cancer	Rate	6.391611196
Deaths	Somalia	Age-standardized	Breast cancer	Rate	12.90633391
Deaths	Somalia	Age-standardized	Cervical cancer	Rate	30.98851366
Deaths	Somalia	Age-standardized	Uterine cancer	Rate	2.5394022
Deaths	Somalia	Age-standardized	Ovarian cancer	Rate	3.332180566
Deaths	United Republic of Tanzania	Age-standardized	Ovarian cancer	Rate	5.32754525
Deaths	Cameroon	Age-standardized	Breast cancer	Rate	21.93212732
Deaths	Cameroon	Age-standardized	Cervical cancer	Rate	20.87052799
Deaths	Cameroon	Age-standardized	Uterine cancer	Rate	2.762594542
Deaths	Cameroon	Age-standardized	Ovarian cancer	Rate	3.969147667
Deaths	United Arab Emirates	Age-standardized	Breast cancer	Rate	26.19235879
Deaths	United Arab Emirates	Age-standardized	Cervical cancer	Rate	6.801419659
Deaths	United Arab Emirates	Age-standardized	Uterine cancer	Rate	1.497970358
Deaths	United Arab Emirates	Age-standardized	Ovarian cancer	Rate	5.673701467
Deaths	Gabon	Age-standardized	Ovarian cancer	Rate	4.880409405
Deaths	Cabo Verde	Age-standardized	Ovarian cancer	Rate	2.52274775
Deaths	Croatia	Age-standardized	Breast cancer	Rate	19.9593668
Deaths	Croatia	Age-standardized	Cervical cancer	Rate	3.678924472
Deaths	Croatia	Age-standardized	Uterine cancer	Rate	3.356754345
Deaths	Croatia	Age-standardized	Ovarian cancer	Rate	6.786532226
Deaths	Yemen	Age-standardized	Breast cancer	Rate	13.43549298
Deaths	Yemen	Age-standardized	Cervical cancer	Rate	4.003313114
Deaths	Yemen	Age-standardized	Uterine cancer	Rate	0.981247307
Deaths	Palau	Age-standardized	Breast cancer	Rate	33.98387979
Deaths	Palau	Age-standardized	Cervical cancer	Rate	29.79107938
Deaths	Palau	Age-standardized	Uterine cancer	Rate	0.516281121
Deaths	Chad	Age-standardized	Breast cancer	Rate	14.04416826

Deaths	Chad	Age-standardized	Cervical cancer	Rate	25.07840413
Deaths	Chad	Age-standardized	Uterine cancer	Rate	2.375772924
Deaths	C么te d'Ivoire	Age-standardized	Breast cancer	Rate	16.13247209
Deaths	C么te d'Ivoire	Age-standardized	Cervical cancer	Rate	18.93712677
Deaths	C么te d'Ivoire	Age-standardized	Uterine cancer	Rate	2.293217598
Deaths	C么te d'Ivoire	Age-standardized	Ovarian cancer	Rate	3.165899949
Incidence	Tonga	Age-standardized	Breast cancer	Rate	55.61488144
Incidence	Tonga	Age-standardized	Cervical cancer	Rate	31.45144065
Incidence	Tonga	Age-standardized	Uterine cancer	Rate	9.253180402
Incidence	Sri Lanka	Age-standardized	Breast cancer	Rate	29.7848638
Incidence	Sri Lanka	Age-standardized	Cervical cancer	Rate	7.840524133
Incidence	Sri Lanka	Age-standardized	Uterine cancer	Rate	6.529447211
Incidence	Sri Lanka	Age-standardized	Ovarian cancer	Rate	5.823242251
Incidence	Brunei Darussalam	Age-standardized	Ovarian cancer	Rate	16.11709402
Incidence	Norway	Age-standardized	Breast cancer	Rate	67.71429325
Incidence	Norway	Age-standardized	Cervical cancer	Rate	8.039199296
Incidence	Norway	Age-standardized	Ovarian cancer	Rate	12.33280667
Incidence	Thailand	Age-standardized	Breast cancer	Rate	33.3142753
Incidence	Thailand	Age-standardized	Cervical cancer	Rate	16.27258073
Incidence	Thailand	Age-standardized	Uterine cancer	Rate	5.064636099
Incidence	Norway	Age-standardized	Uterine cancer	Rate	18.61666894
Incidence	Burundi	Age-standardized	Breast cancer	Rate	20.7283725
Incidence	Burundi	Age-standardized	Cervical cancer	Rate	38.08087725
Incidence	Burundi	Age-standardized	Uterine cancer	Rate	3.53361959
Incidence	Burundi	Age-standardized	Ovarian cancer	Rate	4.585939791
Incidence	Benin	Age-standardized	Ovarian cancer	Rate	3.367962662
Incidence	Algeria	Age-standardized	Breast cancer	Rate	33.99628397
Incidence	Algeria	Age-standardized	Cervical cancer	Rate	9.479132637
Incidence	Algeria	Age-standardized	Uterine cancer	Rate	2.261680473
Incidence	Albania	Age-standardized	Breast cancer	Rate	38.99915534
Incidence	Albania	Age-standardized	Cervical cancer	Rate	7.983522878
Incidence	Albania	Age-standardized	Uterine cancer	Rate	12.15768782
Incidence	Thailand	Age-standardized	Ovarian cancer	Rate	7.106140876
Incidence	Slovakia	Age-standardized	Ovarian cancer	Rate	11.80444416
Incidence	Japan	Age-standardized	Breast cancer	Rate	60.4720046
Incidence	Japan	Age-standardized	Cervical cancer	Rate	11.16926687
Incidence	Japan	Age-standardized	Uterine cancer	Rate	14.51221455
Incidence	Japan	Age-standardized	Ovarian cancer	Rate	7.443830301
Incidence	France	Age-standardized	Ovarian cancer	Rate	9.452907437
Incidence	Albania	Age-standardized	Ovarian cancer	Rate	4.957261892
Incidence	Somalia	Age-standardized	Breast cancer	Rate	14.94064587
Incidence	Somalia	Age-standardized	Cervical cancer	Rate	42.17943401
Incidence	Somalia	Age-standardized	Uterine cancer	Rate	3.268214104
Incidence	Somalia	Age-standardized	Ovarian cancer	Rate	3.888511761
Incidence	Timor-Leste	Age-standardized	Uterine cancer	Rate	6.002989998
Incidence	Timor-Leste	Age-standardized	Ovarian cancer	Rate	5.572796309
Incidence	Haiti	Age-standardized	Breast cancer	Rate	43.44877188
Incidence	Haiti	Age-standardized	Cervical cancer	Rate	44.11800122
Incidence	Haiti	Age-standardized	Uterine cancer	Rate	9.02939888
Incidence	Vanuatu	Age-standardized	Breast cancer	Rate	38.2418905
Incidence	Vanuatu	Age-standardized	Cervical cancer	Rate	28.70202136
Incidence	Vanuatu	Age-standardized	Uterine cancer	Rate	9.555172352
Incidence	Slovakia	Age-standardized	Breast cancer	Rate	60.61136766
Incidence	Slovakia	Age-standardized	Cervical cancer	Rate	16.71601109
Incidence	Slovakia	Age-standardized	Uterine cancer	Rate	22.00817971
Incidence	Brunei Darussalam	Age-standardized	Breast cancer	Rate	67.73234715
Incidence	Brunei Darussalam	Age-standardized	Cervical cancer	Rate	25.03519178
Incidence	Brunei Darussalam	Age-standardized	Uterine cancer	Rate	8.620702382

Incidence	Vanuatu	Age-standardized	Ovarian cancer	Rate	4.262476715
Incidence	Democratic People's Republic of Korea	Age-standardized	Breast cancer	Rate	26.56211524
Incidence	Democratic People's Republic of Korea	Age-standardized	Cervical cancer	Rate	18.45802322
Incidence	Democratic People's Republic of Korea	Age-standardized	Uterine cancer	Rate	7.374668244
Incidence	Democratic People's Republic of Korea	Age-standardized	Ovarian cancer	Rate	4.732536349
Incidence	Bosnia and Herzegovina	Age-standardized	Breast cancer	Rate	55.46891091
Incidence	Bosnia and Herzegovina	Age-standardized	Cervical cancer	Rate	13.47016296
Incidence	Bosnia and Herzegovina	Age-standardized	Uterine cancer	Rate	15.71216454
Incidence	Slovenia	Age-standardized	Breast cancer	Rate	64.72796221
Incidence	Slovenia	Age-standardized	Cervical cancer	Rate	10.03285342
Incidence	Slovenia	Age-standardized	Uterine cancer	Rate	18.28510963
Incidence	Algeria	Age-standardized	Ovarian cancer	Rate	4.098526767
Incidence	Puerto Rico	Age-standardized	Ovarian cancer	Rate	6.861839587
Incidence	Timor-Leste	Age-standardized	Breast cancer	Rate	24.92715757
Incidence	Timor-Leste	Age-standardized	Cervical cancer	Rate	15.52179518
Incidence	Burkina Faso	Age-standardized	Breast cancer	Rate	27.07225535
Incidence	Burkina Faso	Age-standardized	Cervical cancer	Rate	34.4587648
Incidence	Burkina Faso	Age-standardized	Uterine cancer	Rate	3.586853223
Incidence	China	Age-standardized	Ovarian cancer	Rate	4.535544033
Incidence	Qatar	Age-standardized	Breast cancer	Rate	103.7201007
Incidence	Qatar	Age-standardized	Cervical cancer	Rate	8.342181668
Incidence	Qatar	Age-standardized	Uterine cancer	Rate	9.493107479
Incidence	Qatar	Age-standardized	Ovarian cancer	Rate	11.42065857
Incidence	China	Age-standardized	Breast cancer	Rate	35.61250147
Incidence	China	Age-standardized	Cervical cancer	Rate	11.00952938
Incidence	Slovenia	Age-standardized	Ovarian cancer	Rate	9.57188422
Incidence	Czechia	Age-standardized	Ovarian cancer	Rate	12.35004479
Incidence	Nepal	Age-standardized	Breast cancer	Rate	28.81489064
Incidence	Nepal	Age-standardized	Cervical cancer	Rate	13.91421344
Incidence	Nepal	Age-standardized	Uterine cancer	Rate	2.457838574
Incidence	Bosnia and Herzegovina	Age-standardized	Ovarian cancer	Rate	11.44612227
Incidence	Haiti	Age-standardized	Ovarian cancer	Rate	4.430600996
Incidence	Denmark	Age-standardized	Ovarian cancer	Rate	11.78923517
Incidence	Portugal	Age-standardized	Breast cancer	Rate	73.33374547
Incidence	Portugal	Age-standardized	Cervical cancer	Rate	10.37456101
Incidence	Portugal	Age-standardized	Uterine cancer	Rate	19.47874026
Incidence	Saint Kitts and Nevis	Age-standardized	Breast cancer	Rate	84.08069578
Incidence	Saint Kitts and Nevis	Age-standardized	Cervical cancer	Rate	29.0742878
Incidence	Saint Kitts and Nevis	Age-standardized	Uterine cancer	Rate	20.83228349
Incidence	El Salvador	Age-standardized	Breast cancer	Rate	30.08126063
Incidence	El Salvador	Age-standardized	Cervical cancer	Rate	29.33148927
Incidence	El Salvador	Age-standardized	Uterine cancer	Rate	7.127874112
Incidence	Bahrain	Age-standardized	Cervical cancer	Rate	5.645015124
Incidence	Bahrain	Age-standardized	Uterine cancer	Rate	9.133317601
Incidence	Bahrain	Age-standardized	Ovarian cancer	Rate	10.86647282
Incidence	Greece	Age-standardized	Breast cancer	Rate	85.48997574
Incidence	Greece	Age-standardized	Cervical cancer	Rate	8.279004331
Incidence	Greece	Age-standardized	Uterine cancer	Rate	20.59689441
Incidence	Republic of Korea	Age-standardized	Breast cancer	Rate	46.39715237
Incidence	Republic of Korea	Age-standardized	Cervical cancer	Rate	9.08020599
Incidence	Republic of Korea	Age-standardized	Uterine cancer	Rate	4.637250162
Incidence	Hungary	Age-standardized	Ovarian cancer	Rate	11.42951545
Incidence	Taiwan (Province of China)	Age-standardized	Breast cancer	Rate	49.41875887
Incidence	Taiwan (Province of China)	Age-standardized	Cervical cancer	Rate	13.09212465
Incidence	Taiwan (Province of China)	Age-standardized	Uterine cancer	Rate	14.49240746
Incidence	Germany	Age-standardized	Breast cancer	Rate	84.69533562
Incidence	Germany	Age-standardized	Cervical cancer	Rate	9.329778067
Incidence	Germany	Age-standardized	Uterine cancer	Rate	15.51765993

Incidence	Germany	Age-standardized	Ovarian cancer	Rate	10.67665614
Incidence	Belarus	Age-standardized	Breast cancer	Rate	48.8760489
Incidence	Belarus	Age-standardized	Cervical cancer	Rate	15.40968757
Incidence	Belarus	Age-standardized	Uterine cancer	Rate	14.37383493
Incidence	Jamaica	Age-standardized	Ovarian cancer	Rate	8.250647201
Incidence	Azerbaijan	Age-standardized	Ovarian cancer	Rate	5.60701042
Incidence	Saudi Arabia	Age-standardized	Ovarian cancer	Rate	6.273018336
Incidence	India	Age-standardized	Breast cancer	Rate	23.03544651
Incidence	India	Age-standardized	Cervical cancer	Rate	13.10363217
Incidence	India	Age-standardized	Uterine cancer	Rate	2.530198428
Incidence	Croatia	Age-standardized	Breast cancer	Rate	68.33758223
Incidence	Croatia	Age-standardized	Cervical cancer	Rate	12.24560845
Incidence	Croatia	Age-standardized	Uterine cancer	Rate	27.84745623
Incidence	Armenia	Age-standardized	Ovarian cancer	Rate	7.728284785
Incidence	Guatemala	Age-standardized	Breast cancer	Rate	22.06733928
Incidence	Guatemala	Age-standardized	Cervical cancer	Rate	31.18012116
Incidence	Guatemala	Age-standardized	Uterine cancer	Rate	6.532228448
Incidence	Armenia	Age-standardized	Breast cancer	Rate	61.17275735
Incidence	Armenia	Age-standardized	Cervical cancer	Rate	15.61159906
Incidence	Armenia	Age-standardized	Uterine cancer	Rate	15.81064215
Incidence	Saint Vincent and the Grenadines	Age-standardized	Cervical cancer	Rate	41.01000695
Incidence	Saint Vincent and the Grenadines	Age-standardized	Uterine cancer	Rate	19.66903637
Incidence	Saint Vincent and the Grenadines	Age-standardized	Ovarian cancer	Rate	9.084988059
Incidence	Guatemala	Age-standardized	Ovarian cancer	Rate	4.733872424
Incidence	Syrian Arab Republic	Age-standardized	Ovarian cancer	Rate	3.550516338
Incidence	Pakistan	Age-standardized	Ovarian cancer	Rate	15.84853905
Incidence	United Republic of Tanzania	Age-standardized	Ovarian cancer	Rate	6.800831655
Incidence	Taiwan (Province of China)	Age-standardized	Ovarian cancer	Rate	8.683028997
Incidence	North Macedonia	Age-standardized	Breast cancer	Rate	72.94421465
Incidence	North Macedonia	Age-standardized	Cervical cancer	Rate	14.50950995
Incidence	North Macedonia	Age-standardized	Uterine cancer	Rate	25.11890491
Incidence	Greenland	Age-standardized	Breast cancer	Rate	45.17306212
Incidence	Greenland	Age-standardized	Cervical cancer	Rate	24.61878821
Incidence	Greenland	Age-standardized	Uterine cancer	Rate	3.862846421
Incidence	United States of America	Age-standardized	Breast cancer	Rate	94.20601707
Incidence	United States of America	Age-standardized	Cervical cancer	Rate	8.669382737
Incidence	United States of America	Age-standardized	Uterine cancer	Rate	28.80376548
Incidence	United States of America	Age-standardized	Ovarian cancer	Rate	9.908228979
Incidence	Viet Nam	Age-standardized	Breast cancer	Rate	48.64747007
Incidence	Viet Nam	Age-standardized	Cervical cancer	Rate	17.76852587
Incidence	Viet Nam	Age-standardized	Uterine cancer	Rate	4.309424777
Incidence	Singapore	Age-standardized	Ovarian cancer	Rate	7.422203871
Incidence	Iran (Islamic Republic of)	Age-standardized	Breast cancer	Rate	34.04920395
Incidence	Iran (Islamic Republic of)	Age-standardized	Cervical cancer	Rate	3.986777205
Incidence	Iran (Islamic Republic of)	Age-standardized	Uterine cancer	Rate	4.021489989
Incidence	China	Age-standardized	Uterine cancer	Rate	6.387985279
Incidence	Uganda	Age-standardized	Uterine cancer	Rate	6.420798355
Incidence	Uganda	Age-standardized	Ovarian cancer	Rate	8.802569402
Incidence	Djibouti	Age-standardized	Breast cancer	Rate	27.61746401
Incidence	Nepal	Age-standardized	Ovarian cancer	Rate	5.964884179
Incidence	Burkina Faso	Age-standardized	Ovarian cancer	Rate	3.303375488
Incidence	Croatia	Age-standardized	Ovarian cancer	Rate	12.79548113
Incidence	Costa Rica	Age-standardized	Ovarian cancer	Rate	6.20735022
Incidence	Comoros	Age-standardized	Ovarian cancer	Rate	8.22236458
Incidence	Kiribati	Age-standardized	Ovarian cancer	Rate	3.662555342
Incidence	Djibouti	Age-standardized	Ovarian cancer	Rate	7.407847867
Incidence	Uganda	Age-standardized	Breast cancer	Rate	31.44022512
Incidence	Bermuda	Age-standardized	Breast cancer	Rate	80.23393332

Incidence	Bermuda	Age-standardized	Cervical cancer	Rate	8.337051118
Incidence	Bermuda	Age-standardized	Uterine cancer	Rate	15.50901259
Incidence	Sudan	Age-standardized	Breast cancer	Rate	24.04411728
Incidence	Sudan	Age-standardized	Cervical cancer	Rate	5.493179124
Incidence	Sudan	Age-standardized	Uterine cancer	Rate	2.250024124
Incidence	Bulgaria	Age-standardized	Breast cancer	Rate	78.17598562
Incidence	Bulgaria	Age-standardized	Cervical cancer	Rate	22.94339486
Incidence	Bulgaria	Age-standardized	Uterine cancer	Rate	30.65761892
Incidence	Suriname	Age-standardized	Breast cancer	Rate	39.46140536
Incidence	Suriname	Age-standardized	Cervical cancer	Rate	32.61069356
Incidence	Suriname	Age-standardized	Uterine cancer	Rate	6.067886686
Incidence	Saint Lucia	Age-standardized	Ovarian cancer	Rate	9.329211083
Incidence	India	Age-standardized	Ovarian cancer	Rate	5.011083236
Incidence	San Marino	Age-standardized	Ovarian cancer	Rate	7.186553859
Incidence	Cameroon	Age-standardized	Breast cancer	Rate	30.8398372
Incidence	Cameroon	Age-standardized	Cervical cancer	Rate	32.57411071
Incidence	Cameroon	Age-standardized	Uterine cancer	Rate	4.471207054
Incidence	Kiribati	Age-standardized	Breast cancer	Rate	46.02280515
Incidence	Kiribati	Age-standardized	Cervical cancer	Rate	108.800995
Incidence	Kiribati	Age-standardized	Uterine cancer	Rate	8.521302322
Incidence	Azerbaijan	Age-standardized	Breast cancer	Rate	38.50841279
Incidence	Azerbaijan	Age-standardized	Cervical cancer	Rate	11.88056684
Incidence	Azerbaijan	Age-standardized	Uterine cancer	Rate	9.72152853
Incidence	Nigeria	Age-standardized	Breast cancer	Rate	38.82474452
Incidence	Nigeria	Age-standardized	Cervical cancer	Rate	18.23118372
Incidence	Nigeria	Age-standardized	Uterine cancer	Rate	1.311653697
Incidence	Georgia	Age-standardized	Breast cancer	Rate	65.83491142
Incidence	Georgia	Age-standardized	Cervical cancer	Rate	17.17823136
Incidence	Georgia	Age-standardized	Uterine cancer	Rate	23.68419254
Incidence	Georgia	Age-standardized	Ovarian cancer	Rate	9.678311833
Incidence	Republic of Korea	Age-standardized	Ovarian cancer	Rate	5.512807776
Incidence	Honduras	Age-standardized	Breast cancer	Rate	27.29940127
Incidence	Honduras	Age-standardized	Cervical cancer	Rate	18.98688181
Incidence	Honduras	Age-standardized	Uterine cancer	Rate	10.23211824
Incidence	Honduras	Age-standardized	Ovarian cancer	Rate	5.640725465
Incidence	Saint Lucia	Age-standardized	Breast cancer	Rate	55.29465625
Incidence	Saint Lucia	Age-standardized	Cervical cancer	Rate	28.52222226
Incidence	Saint Lucia	Age-standardized	Uterine cancer	Rate	13.06627348
Incidence	Saint Vincent and the Grenadines	Age-standardized	Breast cancer	Rate	68.05169577
Incidence	Montenegro	Age-standardized	Breast cancer	Rate	86.13694614
Incidence	Montenegro	Age-standardized	Cervical cancer	Rate	13.57934757
Incidence	Montenegro	Age-standardized	Uterine cancer	Rate	17.23735739
Incidence	Jamaica	Age-standardized	Breast cancer	Rate	79.28247575
Incidence	Jamaica	Age-standardized	Cervical cancer	Rate	34.14334502
Incidence	Jamaica	Age-standardized	Uterine cancer	Rate	18.50409239
Incidence	Fiji	Age-standardized	Ovarian cancer	Rate	2.950231686
Incidence	Suriname	Age-standardized	Ovarian cancer	Rate	8.867327616
Incidence	Marshall Islands	Age-standardized	Ovarian cancer	Rate	7.245085544
Incidence	American Samoa	Age-standardized	Breast cancer	Rate	55.95435982
Incidence	American Samoa	Age-standardized	Cervical cancer	Rate	18.64709287
Incidence	Puerto Rico	Age-standardized	Breast cancer	Rate	70.80964134
Incidence	Puerto Rico	Age-standardized	Cervical cancer	Rate	10.85506455
Incidence	Puerto Rico	Age-standardized	Uterine cancer	Rate	13.38225898
Incidence	Uganda	Age-standardized	Cervical cancer	Rate	37.91767149
Incidence	Latvia	Age-standardized	Ovarian cancer	Rate	12.0247886
Incidence	Comoros	Age-standardized	Breast cancer	Rate	26.51928147
Incidence	Comoros	Age-standardized	Cervical cancer	Rate	34.70704114
Incidence	Comoros	Age-standardized	Uterine cancer	Rate	4.511337244

Incidence	Czechia	Age-standardized	Breast cancer	Rate	58.33021787
Incidence	Czechia	Age-standardized	Cervical cancer	Rate	11.20451224
Incidence	Czechia	Age-standardized	Uterine cancer	Rate	21.21320341
Incidence	Syrian Arab Republic	Age-standardized	Breast cancer	Rate	26.87998064
Incidence	Syrian Arab Republic	Age-standardized	Cervical cancer	Rate	3.249687339
Incidence	Syrian Arab Republic	Age-standardized	Uterine cancer	Rate	3.808414633
Incidence	Pakistan	Age-standardized	Breast cancer	Rate	76.49448443
Incidence	Pakistan	Age-standardized	Cervical cancer	Rate	7.703104587
Incidence	Cook Islands	Age-standardized	Ovarian cancer	Rate	4.967222482
Incidence	Pakistan	Age-standardized	Uterine cancer	Rate	8.602807608
Incidence	Denmark	Age-standardized	Breast cancer	Rate	84.4800065
Incidence	Denmark	Age-standardized	Cervical cancer	Rate	8.979370096
Incidence	Portugal	Age-standardized	Ovarian cancer	Rate	5.819249722
Incidence	Estonia	Age-standardized	Breast cancer	Rate	65.58530898
Incidence	Estonia	Age-standardized	Cervical cancer	Rate	16.60615043
Incidence	Estonia	Age-standardized	Uterine cancer	Rate	25.08040176
Incidence	Singapore	Age-standardized	Breast cancer	Rate	62.72190381
Incidence	Singapore	Age-standardized	Cervical cancer	Rate	7.699611049
Incidence	Singapore	Age-standardized	Uterine cancer	Rate	9.932645022
Incidence	Egypt	Age-standardized	Ovarian cancer	Rate	3.265215376
Incidence	Belarus	Age-standardized	Ovarian cancer	Rate	8.590428202
Incidence	Cameroon	Age-standardized	Ovarian cancer	Rate	5.174130739
Incidence	Djibouti	Age-standardized	Cervical cancer	Rate	33.90299232
Incidence	Djibouti	Age-standardized	Uterine cancer	Rate	4.914483996
Incidence	Saudi Arabia	Age-standardized	Breast cancer	Rate	43.06609521
Incidence	Saudi Arabia	Age-standardized	Cervical cancer	Rate	4.952895915
Incidence	Saudi Arabia	Age-standardized	Uterine cancer	Rate	6.731588059
Incidence	Bermuda	Age-standardized	Ovarian cancer	Rate	10.29626195
Incidence	Tunisia	Age-standardized	Breast cancer	Rate	46.02166343
Incidence	Tunisia	Age-standardized	Cervical cancer	Rate	5.719067968
Incidence	Tunisia	Age-standardized	Uterine cancer	Rate	4.428402119
Incidence	Hungary	Age-standardized	Breast cancer	Rate	63.99975243
Incidence	Hungary	Age-standardized	Cervical cancer	Rate	14.26792617
Incidence	Hungary	Age-standardized	Uterine cancer	Rate	17.55626452
Incidence	Antigua and Barbuda	Age-standardized	Breast cancer	Rate	80.44348167
Incidence	Antigua and Barbuda	Age-standardized	Cervical cancer	Rate	21.43654193
Incidence	Antigua and Barbuda	Age-standardized	Uterine cancer	Rate	16.23339631
Incidence	Antigua and Barbuda	Age-standardized	Ovarian cancer	Rate	9.502405522
Incidence	Bahamas	Age-standardized	Breast cancer	Rate	92.11532966
Incidence	Bahamas	Age-standardized	Cervical cancer	Rate	23.8157276
Incidence	Bahamas	Age-standardized	Uterine cancer	Rate	15.20237935
Incidence	Kazakhstan	Age-standardized	Breast cancer	Rate	40.06503607
Incidence	Kazakhstan	Age-standardized	Cervical cancer	Rate	18.48093345
Incidence	Kazakhstan	Age-standardized	Uterine cancer	Rate	13.41144374
Incidence	Cabo Verde	Age-standardized	Breast cancer	Rate	27.74730553
Incidence	Finland	Age-standardized	Breast cancer	Rate	84.60799597
Incidence	Finland	Age-standardized	Cervical cancer	Rate	5.178167674
Incidence	Finland	Age-standardized	Uterine cancer	Rate	18.46787955
Incidence	Finland	Age-standardized	Ovarian cancer	Rate	11.53392961
Incidence	Mexico	Age-standardized	Breast cancer	Rate	36.80727773
Incidence	Mexico	Age-standardized	Cervical cancer	Rate	18.34353605
Incidence	Mexico	Age-standardized	Uterine cancer	Rate	5.398918696
Incidence	Spain	Age-standardized	Breast cancer	Rate	69.89736029
Incidence	Spain	Age-standardized	Cervical cancer	Rate	8.699812722
Incidence	Greenland	Age-standardized	Ovarian cancer	Rate	13.57139714
Incidence	Nigeria	Age-standardized	Ovarian cancer	Rate	4.186003043
Incidence	Sao Tome and Principe	Age-standardized	Ovarian cancer	Rate	9.288226001
Incidence	American Samoa	Age-standardized	Uterine cancer	Rate	29.8246173

Incidence	American Samoa	Age-standardized	Ovarian cancer	Rate	15.59884767
Incidence	Niger	Age-standardized	Ovarian cancer	Rate	2.145844718
Incidence	El Salvador	Age-standardized	Ovarian cancer	Rate	6.191043751
Incidence	Bahamas	Age-standardized	Ovarian cancer	Rate	11.52848998
Incidence	Niger	Age-standardized	Breast cancer	Rate	14.36745595
Incidence	Niger	Age-standardized	Cervical cancer	Rate	33.97807453
Incidence	Niger	Age-standardized	Uterine cancer	Rate	3.016077782
Incidence	Tunisia	Age-standardized	Ovarian cancer	Rate	5.394591883
Incidence	Benin	Age-standardized	Breast cancer	Rate	21.18364747
Incidence	Benin	Age-standardized	Cervical cancer	Rate	30.14661742
Incidence	Benin	Age-standardized	Uterine cancer	Rate	3.532170863
Incidence	San Marino	Age-standardized	Breast cancer	Rate	84.15957321
Incidence	San Marino	Age-standardized	Cervical cancer	Rate	6.751616629
Incidence	San Marino	Age-standardized	Uterine cancer	Rate	5.833528686
Incidence	Indonesia	Age-standardized	Breast cancer	Rate	37.42214156
Incidence	Spain	Age-standardized	Uterine cancer	Rate	22.00698458
Incidence	Viet Nam	Age-standardized	Ovarian cancer	Rate	7.010819134
Incidence	Saint Kitts and Nevis	Age-standardized	Ovarian cancer	Rate	10.116687
Incidence	Indonesia	Age-standardized	Ovarian cancer	Rate	8.159578155
Incidence	Indonesia	Age-standardized	Cervical cancer	Rate	12.80533329
Incidence	Indonesia	Age-standardized	Uterine cancer	Rate	6.852260821
Incidence	Republic of Moldova	Age-standardized	Ovarian cancer	Rate	6.562709049
Incidence	New Zealand	Age-standardized	Ovarian cancer	Rate	9.153063707
Incidence	Trinidad and Tobago	Age-standardized	Ovarian cancer	Rate	9.963184693
Incidence	Egypt	Age-standardized	Breast cancer	Rate	29.31176473
Incidence	Egypt	Age-standardized	Cervical cancer	Rate	2.838374816
Incidence	Egypt	Age-standardized	Uterine cancer	Rate	4.45118497
Incidence	Sudan	Age-standardized	Ovarian cancer	Rate	3.070370148
Incidence	United Republic of Tanzania	Age-standardized	Breast cancer	Rate	23.85641693
Incidence	United Republic of Tanzania	Age-standardized	Cervical cancer	Rate	35.16911284
Incidence	United Republic of Tanzania	Age-standardized	Uterine cancer	Rate	4.555112826
Incidence	Sweden	Age-standardized	Breast cancer	Rate	82.51096398
Incidence	Sweden	Age-standardized	Cervical cancer	Rate	6.928689576
Incidence	Sweden	Age-standardized	Uterine cancer	Rate	17.85108184
Incidence	Lithuania	Age-standardized	Breast cancer	Rate	50.50752951
Incidence	Lithuania	Age-standardized	Cervical cancer	Rate	12.43334266
Incidence	Lithuania	Age-standardized	Uterine cancer	Rate	15.12626414
Incidence	Lithuania	Age-standardized	Ovarian cancer	Rate	12.51356211
Incidence	Guam	Age-standardized	Breast cancer	Rate	38.02461649
Incidence	Guam	Age-standardized	Cervical cancer	Rate	13.64711819
Incidence	Guam	Age-standardized	Uterine cancer	Rate	11.5286606
Incidence	United Arab Emirates	Age-standardized	Ovarian cancer	Rate	8.461333492
Incidence	Mexico	Age-standardized	Ovarian cancer	Rate	8.121211449
Incidence	Bulgaria	Age-standardized	Ovarian cancer	Rate	12.28942254
Incidence	Denmark	Age-standardized	Uterine cancer	Rate	17.70253486
Incidence	Bahrain	Age-standardized	Breast cancer	Rate	67.49501356
Incidence	Spain	Age-standardized	Ovarian cancer	Rate	9.304524823
Incidence	Sweden	Age-standardized	Ovarian cancer	Rate	9.52659722
Incidence	United Kingdom	Age-standardized	Breast cancer	Rate	94.28797237
Incidence	United Kingdom	Age-standardized	Cervical cancer	Rate	8.186649638
Incidence	United Kingdom	Age-standardized	Uterine cancer	Rate	18.40511572
Incidence	Estonia	Age-standardized	Ovarian cancer	Rate	10.90976917
Incidence	France	Age-standardized	Breast cancer	Rate	86.90309754
Incidence	France	Age-standardized	Cervical cancer	Rate	8.095957318
Incidence	France	Age-standardized	Uterine cancer	Rate	18.6745986
Incidence	Iran (Islamic Republic of)	Age-standardized	Ovarian cancer	Rate	4.753055907
Incidence	Cambodia	Age-standardized	Breast cancer	Rate	23.52036151
Incidence	Cambodia	Age-standardized	Cervical cancer	Rate	17.82061227

Incidence	Cambodia	Age-standardized	Uterine cancer	Rate	7.198383152
Incidence	Australia	Age-standardized	Cervical cancer	Rate	8.54913056
Incidence	Australia	Age-standardized	Uterine cancer	Rate	10.35445249
Incidence	Fiji	Age-standardized	Breast cancer	Rate	59.21876124
Incidence	Fiji	Age-standardized	Cervical cancer	Rate	40.43653901
Incidence	Kazakhstan	Age-standardized	Ovarian cancer	Rate	10.02043385
Incidence	Cook Islands	Age-standardized	Breast cancer	Rate	90.88826736
Incidence	Cook Islands	Age-standardized	Cervical cancer	Rate	8.863583135
Incidence	Cook Islands	Age-standardized	Uterine cancer	Rate	8.076049759
Incidence	Marshall Islands	Age-standardized	Breast cancer	Rate	58.34284526
Incidence	Marshall Islands	Age-standardized	Cervical cancer	Rate	37.89618179
Incidence	Marshall Islands	Age-standardized	Uterine cancer	Rate	13.59503635
Incidence	Trinidad and Tobago	Age-standardized	Breast cancer	Rate	57.69025943
Incidence	Trinidad and Tobago	Age-standardized	Cervical cancer	Rate	22.28112365
Incidence	Trinidad and Tobago	Age-standardized	Uterine cancer	Rate	16.01993754
Incidence	Kyrgyzstan	Age-standardized	Breast cancer	Rate	22.43058248
Incidence	Micronesia (Federated States of)	Age-standardized	Cervical cancer	Rate	36.20545046
Incidence	Micronesia (Federated States of)	Age-standardized	Uterine cancer	Rate	16.20485682
Incidence	Eritrea	Age-standardized	Breast cancer	Rate	28.73213898
Incidence	Cabo Verde	Age-standardized	Cervical cancer	Rate	23.01529896
Incidence	Cabo Verde	Age-standardized	Uterine cancer	Rate	7.16098204
Incidence	Cabo Verde	Age-standardized	Ovarian cancer	Rate	3.840997902
Incidence	Cambodia	Age-standardized	Ovarian cancer	Rate	8.393171073
Incidence	Micronesia (Federated States of)	Age-standardized	Ovarian cancer	Rate	8.497325337
Incidence	New Zealand	Age-standardized	Breast cancer	Rate	101.2075181
Incidence	New Zealand	Age-standardized	Cervical cancer	Rate	6.362957723
Incidence	New Zealand	Age-standardized	Uterine cancer	Rate	15.92663202
Incidence	Fiji	Age-standardized	Uterine cancer	Rate	10.96495035
Incidence	Panama	Age-standardized	Ovarian cancer	Rate	6.155359083
Incidence	Sao Tome and Principe	Age-standardized	Breast cancer	Rate	32.13239163
Incidence	Sao Tome and Principe	Age-standardized	Cervical cancer	Rate	44.0073991
Incidence	Sao Tome and Principe	Age-standardized	Uterine cancer	Rate	8.357249643
Incidence	Yemen	Age-standardized	Ovarian cancer	Rate	2.593206241
Incidence	Turkey	Age-standardized	Breast cancer	Rate	36.08433474
Incidence	Tokelau	Age-standardized	Breast cancer	Rate	64.01826147
Incidence	Tokelau	Age-standardized	Cervical cancer	Rate	33.61533075
Incidence	Tokelau	Age-standardized	Uterine cancer	Rate	17.07634713
Incidence	Tokelau	Age-standardized	Ovarian cancer	Rate	6.355523174
Incidence	Switzerland	Age-standardized	Breast cancer	Rate	77.46725554
Incidence	Switzerland	Age-standardized	Cervical cancer	Rate	5.733613321
Incidence	Switzerland	Age-standardized	Uterine cancer	Rate	13.50592775
Incidence	Zambia	Age-standardized	Breast cancer	Rate	27.34686069
Incidence	Zambia	Age-standardized	Cervical cancer	Rate	43.37312116
Incidence	Zambia	Age-standardized	Uterine cancer	Rate	5.230407336
Incidence	Barbados	Age-standardized	Ovarian cancer	Rate	9.950753333
Incidence	Australia	Age-standardized	Ovarian cancer	Rate	8.06251445
Incidence	Barbados	Age-standardized	Breast cancer	Rate	102.3095459
Incidence	Barbados	Age-standardized	Cervical cancer	Rate	27.97281949
Incidence	Barbados	Age-standardized	Uterine cancer	Rate	24.28288372
Incidence	Turkey	Age-standardized	Cervical cancer	Rate	4.671283363
Incidence	Turkey	Age-standardized	Uterine cancer	Rate	8.879514884
Incidence	Turkey	Age-standardized	Ovarian cancer	Rate	6.693850519
Incidence	Switzerland	Age-standardized	Ovarian cancer	Rate	8.671004833
Incidence	Ethiopia	Age-standardized	Uterine cancer	Rate	2.840674123
Incidence	Ethiopia	Age-standardized	Ovarian cancer	Rate	5.216037104
Incidence	Latvia	Age-standardized	Breast cancer	Rate	55.60239336
Incidence	Lao People's Democratic Republic	Age-standardized	Ovarian cancer	Rate	7.925679071
Incidence	Republic of Moldova	Age-standardized	Breast cancer	Rate	39.16233108

Incidence	Republic of Moldova	Age-standardized	Cervical cancer	Rate	14.76757514
Incidence	Republic of Moldova	Age-standardized	Uterine cancer	Rate	14.33287034
Incidence	Latvia	Age-standardized	Cervical cancer	Rate	8.677501959
Incidence	Latvia	Age-standardized	Uterine cancer	Rate	29.10305269
Incidence	Ethiopia	Age-standardized	Breast cancer	Rate	23.00114631
Incidence	Ethiopia	Age-standardized	Cervical cancer	Rate	24.59817863
Incidence	Venezuela (Bolivarian Republic of)	Age-standardized	Breast cancer	Rate	52.99779692
Incidence	Venezuela (Bolivarian Republic of)	Age-standardized	Cervical cancer	Rate	34.35511956
Incidence	Venezuela (Bolivarian Republic of)	Age-standardized	Uterine cancer	Rate	9.232904331
Incidence	C么te d'Ivoire	Age-standardized	Ovarian cancer	Rate	4.03822087
Incidence	United Kingdom	Age-standardized	Ovarian cancer	Rate	13.22206806
Incidence	Greece	Age-standardized	Ovarian cancer	Rate	10.17649069
Incidence	Papua New Guinea	Age-standardized	Breast cancer	Rate	64.51505016
Incidence	Papua New Guinea	Age-standardized	Cervical cancer	Rate	23.52128265
Incidence	Papua New Guinea	Age-standardized	Uterine cancer	Rate	6.658957223
Incidence	Cuba	Age-standardized	Ovarian cancer	Rate	6.162684787
Incidence	Bolivia (Plurinational State of)	Age-standardized	Breast cancer	Rate	34.52096949
Incidence	Bolivia (Plurinational State of)	Age-standardized	Cervical cancer	Rate	41.59027083
Incidence	Bolivia (Plurinational State of)	Age-standardized	Uterine cancer	Rate	10.85559302
Incidence	Bolivia (Plurinational State of)	Age-standardized	Ovarian cancer	Rate	6.282496069
Incidence	Venezuela (Bolivarian Republic of)	Age-standardized	Ovarian cancer	Rate	7.30002257
Incidence	Iraq	Age-standardized	Breast cancer	Rate	51.99932362
Incidence	Iraq	Age-standardized	Cervical cancer	Rate	4.607115862
Incidence	Iraq	Age-standardized	Uterine cancer	Rate	6.740470966
Incidence	Papua New Guinea	Age-standardized	Ovarian cancer	Rate	3.7029215
Incidence	Iraq	Age-standardized	Ovarian cancer	Rate	6.027961896
Incidence	Eritrea	Age-standardized	Cervical cancer	Rate	44.96290565
Incidence	Eritrea	Age-standardized	Uterine cancer	Rate	4.905092045
Incidence	Eritrea	Age-standardized	Ovarian cancer	Rate	6.426615116
Incidence	Tuvalu	Age-standardized	Ovarian cancer	Rate	6.916858782
Incidence	Kyrgyzstan	Age-standardized	Cervical cancer	Rate	18.27181707
Incidence	Kyrgyzstan	Age-standardized	Uterine cancer	Rate	9.434636812
Incidence	United Arab Emirates	Age-standardized	Breast cancer	Rate	57.47842292
Incidence	United Arab Emirates	Age-standardized	Cervical cancer	Rate	10.81790837
Incidence	United Arab Emirates	Age-standardized	Uterine cancer	Rate	4.72367303
Incidence	Guam	Age-standardized	Ovarian cancer	Rate	6.718842291
Incidence	Chad	Age-standardized	Breast cancer	Rate	17.69635578
Incidence	Chad	Age-standardized	Cervical cancer	Rate	35.474368
Incidence	Chad	Age-standardized	Uterine cancer	Rate	3.274843032
Incidence	Kyrgyzstan	Age-standardized	Ovarian cancer	Rate	7.025212772
Incidence	Angola	Age-standardized	Breast cancer	Rate	26.42691462
Incidence	Angola	Age-standardized	Cervical cancer	Rate	30.30809741
Incidence	Angola	Age-standardized	Uterine cancer	Rate	3.113490363
Incidence	Zambia	Age-standardized	Ovarian cancer	Rate	7.531726469
Incidence	Argentina	Age-standardized	Ovarian cancer	Rate	8.956709007
Incidence	Senegal	Age-standardized	Ovarian cancer	Rate	4.130869329
Incidence	Chad	Age-standardized	Ovarian cancer	Rate	2.248558643
Incidence	Iceland	Age-standardized	Breast cancer	Rate	67.50004565
Incidence	Iceland	Age-standardized	Cervical cancer	Rate	5.774425631
Incidence	Iceland	Age-standardized	Uterine cancer	Rate	13.34575543
Incidence	Jordan	Age-standardized	Breast cancer	Rate	52.94732794
Incidence	Jordan	Age-standardized	Cervical cancer	Rate	4.02530361
Incidence	Jordan	Age-standardized	Uterine cancer	Rate	7.354627944
Incidence	Iceland	Age-standardized	Ovarian cancer	Rate	9.412084351
Incidence	Kenya	Age-standardized	Ovarian cancer	Rate	5.44731096
Incidence	Malaysia	Age-standardized	Uterine cancer	Rate	9.141824699
Incidence	Malaysia	Age-standardized	Ovarian cancer	Rate	8.102190478
Incidence	Angola	Age-standardized	Ovarian cancer	Rate	3.271537849

Incidence	North Macedonia	Age-standardized	Ovarian cancer	Rate	10.81429727
Incidence	Belize	Age-standardized	Breast cancer	Rate	26.98872414
Incidence	Belize	Age-standardized	Cervical cancer	Rate	35.7807544
Incidence	Belize	Age-standardized	Uterine cancer	Rate	11.82858546
Incidence	Belize	Age-standardized	Ovarian cancer	Rate	4.20351176
Incidence	Tajikistan	Age-standardized	Ovarian cancer	Rate	5.482414605
Incidence	Australia	Age-standardized	Breast cancer	Rate	81.54826484
Incidence	Senegal	Age-standardized	Breast cancer	Rate	29.11116039
Incidence	Senegal	Age-standardized	Cervical cancer	Rate	29.88253575
Incidence	Senegal	Age-standardized	Uterine cancer	Rate	3.772541815
Incidence	Tuvalu	Age-standardized	Breast cancer	Rate	57.89126583
Incidence	Tuvalu	Age-standardized	Cervical cancer	Rate	32.26137395
Incidence	Tuvalu	Age-standardized	Uterine cancer	Rate	13.5117902
Incidence	Chile	Age-standardized	Uterine cancer	Rate	7.96041825
Incidence	Micronesia (Federated States of)	Age-standardized	Breast cancer	Rate	66.07648018
Incidence	Nicaragua	Age-standardized	Breast cancer	Rate	32.84774281
Incidence	Nicaragua	Age-standardized	Cervical cancer	Rate	29.75808125
Incidence	Nicaragua	Age-standardized	Uterine cancer	Rate	5.122116195
Incidence	Congo	Age-standardized	Ovarian cancer	Rate	4.87549543
Incidence	Chile	Age-standardized	Ovarian cancer	Rate	7.326335003
Incidence	Gambia	Age-standardized	Breast cancer	Rate	17.02413183
Incidence	Gambia	Age-standardized	Cervical cancer	Rate	26.10305776
Incidence	Kenya	Age-standardized	Breast cancer	Rate	22.96392347
Incidence	Kenya	Age-standardized	Cervical cancer	Rate	18.29675971
Incidence	Kenya	Age-standardized	Uterine cancer	Rate	2.236831654
Incidence	Gambia	Age-standardized	Uterine cancer	Rate	2.968758939
Incidence	Gambia	Age-standardized	Ovarian cancer	Rate	4.352051065
Incidence	Lao People's Democratic Republic	Age-standardized	Breast cancer	Rate	31.24391981
Incidence	Lao People's Democratic Republic	Age-standardized	Cervical cancer	Rate	15.68554774
Incidence	Lao People's Democratic Republic	Age-standardized	Uterine cancer	Rate	6.174447308
Incidence	Botswana	Age-standardized	Breast cancer	Rate	49.01923555
Incidence	Botswana	Age-standardized	Cervical cancer	Rate	47.63494291
Incidence	Botswana	Age-standardized	Uterine cancer	Rate	7.773827303
Incidence	Italy	Age-standardized	Ovarian cancer	Rate	9.686902281
Incidence	Dominica	Age-standardized	Breast cancer	Rate	65.38252278
Incidence	C么te d'Ivoire	Age-standardized	Breast cancer	Rate	21.38997937
Incidence	C么te d'Ivoire	Age-standardized	Cervical cancer	Rate	28.23527685
Incidence	C么te d'Ivoire	Age-standardized	Uterine cancer	Rate	3.409072113
Incidence	Yemen	Age-standardized	Breast cancer	Rate	22.67600332
Incidence	Yemen	Age-standardized	Cervical cancer	Rate	6.386100609
Incidence	Yemen	Age-standardized	Uterine cancer	Rate	2.060063997
Incidence	Ecuador	Age-standardized	Ovarian cancer	Rate	7.252952717
Incidence	Ecuador	Age-standardized	Breast cancer	Rate	31.025675
Incidence	Ecuador	Age-standardized	Cervical cancer	Rate	27.2013876
Incidence	Ecuador	Age-standardized	Uterine cancer	Rate	10.80097136
Incidence	Uruguay	Age-standardized	Breast cancer	Rate	72.6450292
Incidence	Uruguay	Age-standardized	Cervical cancer	Rate	21.81104411
Incidence	Uruguay	Age-standardized	Uterine cancer	Rate	8.097119546
Incidence	Turkmenistan	Age-standardized	Breast cancer	Rate	28.78704291
Incidence	Turkmenistan	Age-standardized	Cervical cancer	Rate	15.59449484
Incidence	Turkmenistan	Age-standardized	Uterine cancer	Rate	3.147715464
Incidence	Samoa	Age-standardized	Breast cancer	Rate	43.98371296
Incidence	Samoa	Age-standardized	Cervical cancer	Rate	25.68444052
Incidence	Samoa	Age-standardized	Uterine cancer	Rate	11.46733941
Incidence	Andorra	Age-standardized	Breast cancer	Rate	80.62848194
Incidence	Andorra	Age-standardized	Cervical cancer	Rate	12.48514086
Incidence	Andorra	Age-standardized	Uterine cancer	Rate	22.38504859
Incidence	Andorra	Age-standardized	Ovarian cancer	Rate	7.487019185

Incidence	Madagascar	Age-standardized	Ovarian cancer	Rate	4.934277113
Incidence	Nicaragua	Age-standardized	Ovarian cancer	Rate	5.457611345
Incidence	Turkmenistan	Age-standardized	Ovarian cancer	Rate	5.431788783
Incidence	Dominica	Age-standardized	Cervical cancer	Rate	33.46095075
Incidence	Dominica	Age-standardized	Uterine cancer	Rate	10.54013697
Incidence	Dominica	Age-standardized	Ovarian cancer	Rate	4.869728442
Incidence	Ireland	Age-standardized	Breast cancer	Rate	94.95522227
Incidence	Ireland	Age-standardized	Cervical cancer	Rate	9.63711621
Incidence	Ireland	Age-standardized	Uterine cancer	Rate	21.12214305
Incidence	Montenegro	Age-standardized	Ovarian cancer	Rate	9.4457703
Incidence	United States Virgin Islands	Age-standardized	Breast cancer	Rate	74.54435947
Incidence	United States Virgin Islands	Age-standardized	Cervical cancer	Rate	15.45904389
Incidence	United States Virgin Islands	Age-standardized	Uterine cancer	Rate	12.0152802
Incidence	Poland	Age-standardized	Breast cancer	Rate	55.53771451
Incidence	Poland	Age-standardized	Cervical cancer	Rate	10.52682251
Incidence	Poland	Age-standardized	Uterine cancer	Rate	20.89726955
Incidence	Afghanistan	Age-standardized	Breast cancer	Rate	22.28219032
Incidence	Afghanistan	Age-standardized	Cervical cancer	Rate	11.3859649
Incidence	Afghanistan	Age-standardized	Uterine cancer	Rate	3.055925692
Incidence	Afghanistan	Age-standardized	Ovarian cancer	Rate	3.65496966
Incidence	Uruguay	Age-standardized	Ovarian cancer	Rate	9.605871627
Incidence	Jordan	Age-standardized	Ovarian cancer	Rate	5.420452143
Incidence	Peru	Age-standardized	Breast cancer	Rate	27.63306751
Incidence	Cuba	Age-standardized	Breast cancer	Rate	59.3760467
Incidence	Cuba	Age-standardized	Cervical cancer	Rate	19.25153145
Incidence	Cuba	Age-standardized	Uterine cancer	Rate	26.49183764
Incidence	Mongolia	Age-standardized	Breast cancer	Rate	17.86387379
Incidence	Mongolia	Age-standardized	Cervical cancer	Rate	20.96516585
Incidence	Mongolia	Age-standardized	Uterine cancer	Rate	6.716486027
Incidence	Mongolia	Age-standardized	Ovarian cancer	Rate	6.135633233
Incidence	Maldives	Age-standardized	Breast cancer	Rate	33.75180605
Incidence	Maldives	Age-standardized	Cervical cancer	Rate	8.781285973
Incidence	Maldives	Age-standardized	Uterine cancer	Rate	4.867298513
Incidence	United States Virgin Islands	Age-standardized	Ovarian cancer	Rate	14.13272887
Incidence	Peru	Age-standardized	Cervical cancer	Rate	27.64069326
Incidence	Peru	Age-standardized	Uterine cancer	Rate	8.983432489
Incidence	Peru	Age-standardized	Ovarian cancer	Rate	7.218731295
Incidence	Kuwait	Age-standardized	Breast cancer	Rate	42.76946455
Incidence	Kuwait	Age-standardized	Cervical cancer	Rate	3.615961945
Incidence	Austria	Age-standardized	Breast cancer	Rate	69.85950797
Incidence	Austria	Age-standardized	Cervical cancer	Rate	7.280623463
Incidence	Austria	Age-standardized	Uterine cancer	Rate	16.62405018
Incidence	Botswana	Age-standardized	Ovarian cancer	Rate	8.110543098
Incidence	Paraguay	Age-standardized	Breast cancer	Rate	44.32550097
Incidence	Paraguay	Age-standardized	Cervical cancer	Rate	34.25907915
Incidence	Paraguay	Age-standardized	Uterine cancer	Rate	9.018815639
Incidence	Dominican Republic	Age-standardized	Breast cancer	Rate	36.11805865
Incidence	Dominican Republic	Age-standardized	Cervical cancer	Rate	25.40478199
Incidence	Dominican Republic	Age-standardized	Uterine cancer	Rate	13.78671002
Incidence	Argentina	Age-standardized	Breast cancer	Rate	62.71964927
Incidence	Argentina	Age-standardized	Cervical cancer	Rate	28.02362636
Incidence	South Africa	Age-standardized	Breast cancer	Rate	32.07965711
Incidence	South Africa	Age-standardized	Cervical cancer	Rate	29.19011072
Incidence	South Africa	Age-standardized	Uterine cancer	Rate	4.357886527
Incidence	Ghana	Age-standardized	Breast cancer	Rate	45.06228754
Incidence	Ghana	Age-standardized	Cervical cancer	Rate	27.6513183
Incidence	Ghana	Age-standardized	Uterine cancer	Rate	4.928898522
Incidence	Democratic Republic of the Congo	Age-standardized	Uterine cancer	Rate	2.815251166

Incidence	Bangladesh	Age-standardized	Breast cancer	Rate	25.03097675
Incidence	Bangladesh	Age-standardized	Cervical cancer	Rate	10.06821801
Incidence	Bangladesh	Age-standardized	Uterine cancer	Rate	1.901040674
Incidence	Argentina	Age-standardized	Uterine cancer	Rate	8.355500604
Incidence	South Africa	Age-standardized	Ovarian cancer	Rate	6.13989297
Incidence	Myanmar	Age-standardized	Ovarian cancer	Rate	8.101057356
Incidence	Samoa	Age-standardized	Ovarian cancer	Rate	13.11838431
Incidence	Uzbekistan	Age-standardized	Breast cancer	Rate	37.26799389
Incidence	Uzbekistan	Age-standardized	Cervical cancer	Rate	17.26153484
Incidence	Uzbekistan	Age-standardized	Uterine cancer	Rate	10.21865363
Incidence	Central African Republic	Age-standardized	Breast cancer	Rate	23.73665417
Incidence	Central African Republic	Age-standardized	Cervical cancer	Rate	40.67569462
Incidence	Central African Republic	Age-standardized	Uterine cancer	Rate	2.860982487
Incidence	Central African Republic	Age-standardized	Ovarian cancer	Rate	2.658773906
Incidence	Maldives	Age-standardized	Ovarian cancer	Rate	9.97735615
Incidence	Romania	Age-standardized	Breast cancer	Rate	50.91875901
Incidence	Romania	Age-standardized	Cervical cancer	Rate	27.36052291
Incidence	Romania	Age-standardized	Uterine cancer	Rate	15.63330278
Incidence	Romania	Age-standardized	Ovarian cancer	Rate	10.34951999
Incidence	Malaysia	Age-standardized	Breast cancer	Rate	59.47933321
Incidence	Malaysia	Age-standardized	Cervical cancer	Rate	17.91815362
Incidence	Panama	Age-standardized	Breast cancer	Rate	43.13032768
Incidence	Panama	Age-standardized	Cervical cancer	Rate	22.51973162
Incidence	Panama	Age-standardized	Uterine cancer	Rate	9.744422721
Incidence	Sierra Leone	Age-standardized	Breast cancer	Rate	24.01943421
Incidence	Sierra Leone	Age-standardized	Cervical cancer	Rate	34.00416602
Incidence	Sierra Leone	Age-standardized	Uterine cancer	Rate	3.596877969
Incidence	Sierra Leone	Age-standardized	Ovarian cancer	Rate	3.626956401
Incidence	Austria	Age-standardized	Ovarian cancer	Rate	9.621661113
Incidence	Chile	Age-standardized	Breast cancer	Rate	39.39153356
Incidence	Chile	Age-standardized	Cervical cancer	Rate	18.07657141
Incidence	Ireland	Age-standardized	Ovarian cancer	Rate	12.83899529
Incidence	Guinea-Bissau	Age-standardized	Ovarian cancer	Rate	4.284273173
Incidence	Malawi	Age-standardized	Breast cancer	Rate	22.16693799
Incidence	Malawi	Age-standardized	Cervical cancer	Rate	39.79685305
Incidence	Malawi	Age-standardized	Uterine cancer	Rate	2.141522415
Incidence	Poland	Age-standardized	Ovarian cancer	Rate	12.77549334
Incidence	Kuwait	Age-standardized	Uterine cancer	Rate	10.03009028
Incidence	Democratic Republic of the Congo	Age-standardized	Ovarian cancer	Rate	2.975176861
Incidence	Lesotho	Age-standardized	Breast cancer	Rate	36.99560249
Incidence	Lesotho	Age-standardized	Cervical cancer	Rate	52.76661703
Incidence	Lesotho	Age-standardized	Uterine cancer	Rate	5.757214611
Incidence	Israel	Age-standardized	Breast cancer	Rate	78.82001629
Incidence	Israel	Age-standardized	Cervical cancer	Rate	6.646674763
Incidence	Israel	Age-standardized	Uterine cancer	Rate	15.71411936
Incidence	Serbia	Age-standardized	Breast cancer	Rate	83.47500694
Incidence	Serbia	Age-standardized	Cervical cancer	Rate	22.27284112
Incidence	Serbia	Age-standardized	Uterine cancer	Rate	23.04380937
Incidence	Congo	Age-standardized	Breast cancer	Rate	40.46661818
Incidence	Congo	Age-standardized	Cervical cancer	Rate	37.2352968
Incidence	Congo	Age-standardized	Uterine cancer	Rate	4.526341971
Incidence	Uzbekistan	Age-standardized	Ovarian cancer	Rate	5.35735531
Incidence	Malawi	Age-standardized	Ovarian cancer	Rate	5.083391135
Incidence	Grenada	Age-standardized	Breast cancer	Rate	70.94446875
Incidence	Grenada	Age-standardized	Cervical cancer	Rate	34.27508703
Incidence	Namibia	Age-standardized	Ovarian cancer	Rate	4.892680762
Incidence	Monaco	Age-standardized	Breast cancer	Rate	149.5993757
Incidence	Monaco	Age-standardized	Cervical cancer	Rate	8.474504539

Incidence	Monaco	Age-standardized	Uterine cancer	Rate	9.926544103
Incidence	Togo	Age-standardized	Ovarian cancer	Rate	3.86186408
Incidence	Tajikistan	Age-standardized	Breast cancer	Rate	26.02643366
Incidence	Tajikistan	Age-standardized	Cervical cancer	Rate	7.648206139
Incidence	Tajikistan	Age-standardized	Uterine cancer	Rate	10.4454641
Incidence	Kuwait	Age-standardized	Ovarian cancer	Rate	5.678955309
Incidence	Russian Federation	Age-standardized	Breast cancer	Rate	54.38933506
Incidence	Russian Federation	Age-standardized	Cervical cancer	Rate	15.65956369
Incidence	Russian Federation	Age-standardized	Uterine cancer	Rate	32.55354269
Incidence	Russian Federation	Age-standardized	Ovarian cancer	Rate	10.93985289
Incidence	Equatorial Guinea	Age-standardized	Breast cancer	Rate	38.9936381
Incidence	Lesotho	Age-standardized	Ovarian cancer	Rate	7.649169905
Incidence	Madagascar	Age-standardized	Breast cancer	Rate	21.91256141
Incidence	Madagascar	Age-standardized	Cervical cancer	Rate	33.53925819
Incidence	Madagascar	Age-standardized	Uterine cancer	Rate	3.579634724
Incidence	Colombia	Age-standardized	Breast cancer	Rate	39.41000526
Incidence	Colombia	Age-standardized	Cervical cancer	Rate	18.72736542
Incidence	Colombia	Age-standardized	Uterine cancer	Rate	6.009585828
Incidence	Brazil	Age-standardized	Ovarian cancer	Rate	6.14349648
Incidence	Canada	Age-standardized	Breast cancer	Rate	90.09877345
Incidence	Canada	Age-standardized	Cervical cancer	Rate	11.25501897
Incidence	Canada	Age-standardized	Uterine cancer	Rate	19.561251
Incidence	Ghana	Age-standardized	Ovarian cancer	Rate	5.031045072
Incidence	Palau	Age-standardized	Ovarian cancer	Rate	4.717160829
Incidence	Gabon	Age-standardized	Ovarian cancer	Rate	6.315136615
Incidence	Libya	Age-standardized	Ovarian cancer	Rate	7.326324988
Incidence	Monaco	Age-standardized	Ovarian cancer	Rate	22.74574966
Incidence	Israel	Age-standardized	Ovarian cancer	Rate	9.470102596
Incidence	Solomon Islands	Age-standardized	Breast cancer	Rate	126.4800192
Incidence	Solomon Islands	Age-standardized	Cervical cancer	Rate	56.99714453
Incidence	Solomon Islands	Age-standardized	Uterine cancer	Rate	17.33864692
Incidence	Solomon Islands	Age-standardized	Ovarian cancer	Rate	5.967957652
Incidence	Colombia	Age-standardized	Ovarian cancer	Rate	7.633286427
Incidence	Mauritius	Age-standardized	Breast cancer	Rate	53.47746926
Incidence	Mauritius	Age-standardized	Cervical cancer	Rate	12.09348211
Incidence	Mauritius	Age-standardized	Uterine cancer	Rate	12.24744759
Incidence	Brazil	Age-standardized	Breast cancer	Rate	39.64462081
Incidence	Brazil	Age-standardized	Cervical cancer	Rate	17.50776571
Incidence	Brazil	Age-standardized	Uterine cancer	Rate	6.924003785
Incidence	Belgium	Age-standardized	Breast cancer	Rate	95.21328087
Incidence	Belgium	Age-standardized	Cervical cancer	Rate	7.494818895
Incidence	Belgium	Age-standardized	Uterine cancer	Rate	18.13289555
Incidence	Belgium	Age-standardized	Ovarian cancer	Rate	9.609441637
Incidence	Liberia	Age-standardized	Ovarian cancer	Rate	4.449662016
Incidence	Ukraine	Age-standardized	Breast cancer	Rate	45.04014007
Incidence	Ukraine	Age-standardized	Cervical cancer	Rate	11.90917336
Incidence	Ukraine	Age-standardized	Uterine cancer	Rate	16.19323539
Incidence	Morocco	Age-standardized	Breast cancer	Rate	52.51540785
Incidence	Morocco	Age-standardized	Cervical cancer	Rate	14.0294662
Incidence	Morocco	Age-standardized	Uterine cancer	Rate	4.43904702
Incidence	Morocco	Age-standardized	Ovarian cancer	Rate	7.728204263
Incidence	Seychelles	Age-standardized	Ovarian cancer	Rate	15.65792823
Incidence	Democratic Republic of the Congo	Age-standardized	Breast cancer	Rate	28.78106748
Incidence	Democratic Republic of the Congo	Age-standardized	Cervical cancer	Rate	32.31062698
Incidence	Togo	Age-standardized	Breast cancer	Rate	24.40983636
Incidence	Togo	Age-standardized	Cervical cancer	Rate	30.94165833
Incidence	Togo	Age-standardized	Uterine cancer	Rate	3.566673451
Incidence	Serbia	Age-standardized	Ovarian cancer	Rate	11.84440281

Incidence	Mauritius	Age-standardized	Ovarian cancer	Rate	9.683051493
Incidence	Mali	Age-standardized	Breast cancer	Rate	23.16049569
Incidence	Mali	Age-standardized	Cervical cancer	Rate	28.0732797
Incidence	Mali	Age-standardized	Uterine cancer	Rate	2.677717483
Incidence	Namibia	Age-standardized	Breast cancer	Rate	48.17409656
Incidence	Namibia	Age-standardized	Cervical cancer	Rate	26.92772509
Incidence	Namibia	Age-standardized	Uterine cancer	Rate	4.355024394
Incidence	Equatorial Guinea	Age-standardized	Cervical cancer	Rate	27.7410708
Incidence	Equatorial Guinea	Age-standardized	Uterine cancer	Rate	4.338546584
Incidence	Equatorial Guinea	Age-standardized	Ovarian cancer	Rate	5.095086258
Incidence	Costa Rica	Age-standardized	Breast cancer	Rate	54.17243339
Incidence	Costa Rica	Age-standardized	Cervical cancer	Rate	16.62967018
Incidence	Costa Rica	Age-standardized	Uterine cancer	Rate	10.24930745
Incidence	Nauru	Age-standardized	Breast cancer	Rate	75.52082458
Incidence	Nauru	Age-standardized	Cervical cancer	Rate	39.50121321
Incidence	Nauru	Age-standardized	Uterine cancer	Rate	19.899259
Incidence	Ukraine	Age-standardized	Ovarian cancer	Rate	11.11969888
Incidence	Lebanon	Age-standardized	Breast cancer	Rate	122.5146413
Incidence	Lebanon	Age-standardized	Cervical cancer	Rate	6.078436937
Incidence	Lebanon	Age-standardized	Uterine cancer	Rate	12.6227175
Incidence	Lebanon	Age-standardized	Ovarian cancer	Rate	11.7761412
Incidence	Mali	Age-standardized	Ovarian cancer	Rate	2.497238714
Incidence	Mozambique	Age-standardized	Breast cancer	Rate	27.15416952
Incidence	Mozambique	Age-standardized	Cervical cancer	Rate	43.2076628
Incidence	Palestine	Age-standardized	Breast cancer	Rate	57.13680877
Incidence	Palestine	Age-standardized	Cervical cancer	Rate	4.664733054
Incidence	Palestine	Age-standardized	Uterine cancer	Rate	12.53720659
Incidence	Nauru	Age-standardized	Ovarian cancer	Rate	7.695459511
Incidence	Niue	Age-standardized	Ovarian cancer	Rate	8.555523901
Incidence	Myanmar	Age-standardized	Breast cancer	Rate	26.63705392
Incidence	Myanmar	Age-standardized	Cervical cancer	Rate	14.25328413
Incidence	Myanmar	Age-standardized	Uterine cancer	Rate	6.015320796
Incidence	Italy	Age-standardized	Breast cancer	Rate	89.17135932
Incidence	Italy	Age-standardized	Cervical cancer	Rate	7.567476617
Incidence	Italy	Age-standardized	Uterine cancer	Rate	26.93079571
Incidence	Cyprus	Age-standardized	Ovarian cancer	Rate	9.786931539
Incidence	Philippines	Age-standardized	Breast cancer	Rate	40.62580834
Incidence	Philippines	Age-standardized	Cervical cancer	Rate	13.89529841
Incidence	Philippines	Age-standardized	Uterine cancer	Rate	7.118954589
Incidence	Paraguay	Age-standardized	Ovarian cancer	Rate	5.59741657
Incidence	Luxembourg	Age-standardized	Breast cancer	Rate	79.66442373
Incidence	Luxembourg	Age-standardized	Cervical cancer	Rate	5.31790809
Incidence	Luxembourg	Age-standardized	Uterine cancer	Rate	21.75114025
Incidence	South Sudan	Age-standardized	Breast cancer	Rate	15.91351617
Incidence	South Sudan	Age-standardized	Cervical cancer	Rate	25.59038883
Incidence	South Sudan	Age-standardized	Uterine cancer	Rate	3.280589635
Incidence	Philippines	Age-standardized	Ovarian cancer	Rate	9.90877478
Incidence	Tonga	Age-standardized	Ovarian cancer	Rate	6.262132611
Incidence	Oman	Age-standardized	Breast cancer	Rate	44.65997788
Incidence	Mauritania	Age-standardized	Breast cancer	Rate	26.41648079
Incidence	Mauritania	Age-standardized	Cervical cancer	Rate	28.0066475
Incidence	Mauritania	Age-standardized	Uterine cancer	Rate	4.595966994
Incidence	Dominican Republic	Age-standardized	Ovarian cancer	Rate	2.534486766
Incidence	Bangladesh	Age-standardized	Ovarian cancer	Rate	4.568704841
Incidence	Gabon	Age-standardized	Breast cancer	Rate	39.98456835
Incidence	Gabon	Age-standardized	Cervical cancer	Rate	26.92516737
Incidence	Gabon	Age-standardized	Uterine cancer	Rate	4.468521992
Incidence	South Sudan	Age-standardized	Ovarian cancer	Rate	4.646265254

Incidence	Guinea	Age-standardized	Breast cancer	Rate	24.64793678
Incidence	Guinea	Age-standardized	Cervical cancer	Rate	53.60568043
Incidence	Guinea	Age-standardized	Uterine cancer	Rate	3.652155317
Incidence	Eswatini	Age-standardized	Cervical cancer	Rate	41.01538191
Incidence	Eswatini	Age-standardized	Uterine cancer	Rate	5.311735035
Incidence	Eswatini	Age-standardized	Ovarian cancer	Rate	8.020229226
Incidence	Mozambique	Age-standardized	Uterine cancer	Rate	4.067038332
Incidence	Mozambique	Age-standardized	Ovarian cancer	Rate	5.997402272
Incidence	Canada	Age-standardized	Ovarian cancer	Rate	9.371436104
Incidence	Cyprus	Age-standardized	Breast cancer	Rate	101.3340381
Incidence	Cyprus	Age-standardized	Cervical cancer	Rate	6.488990372
Incidence	Cyprus	Age-standardized	Uterine cancer	Rate	20.5364513
Incidence	Palestine	Age-standardized	Ovarian cancer	Rate	6.61833329
Incidence	Grenada	Age-standardized	Uterine cancer	Rate	29.41181647
Incidence	Luxembourg	Age-standardized	Ovarian cancer	Rate	11.41386217
Incidence	Northern Mariana Islands	Age-standardized	Breast cancer	Rate	65.55463404
Incidence	Northern Mariana Islands	Age-standardized	Cervical cancer	Rate	36.16932889
Incidence	Northern Mariana Islands	Age-standardized	Uterine cancer	Rate	32.76676489
Incidence	Northern Mariana Islands	Age-standardized	Ovarian cancer	Rate	7.75407295
Incidence	Grenada	Age-standardized	Ovarian cancer	Rate	11.23635342
Incidence	Libya	Age-standardized	Breast cancer	Rate	41.43853949
Incidence	Libya	Age-standardized	Cervical cancer	Rate	8.289111761
Incidence	Libya	Age-standardized	Uterine cancer	Rate	5.912816944
Incidence	Bhutan	Age-standardized	Breast cancer	Rate	20.86618985
Incidence	Bhutan	Age-standardized	Cervical cancer	Rate	12.93690289
Incidence	Bhutan	Age-standardized	Uterine cancer	Rate	3.048056507
Incidence	Bhutan	Age-standardized	Ovarian cancer	Rate	6.839969306
Incidence	Guinea	Age-standardized	Ovarian cancer	Rate	5.198977512
Incidence	Oman	Age-standardized	Cervical cancer	Rate	5.937067301
Incidence	Oman	Age-standardized	Uterine cancer	Rate	3.852887459
Incidence	Oman	Age-standardized	Ovarian cancer	Rate	6.849193094
Incidence	Guyana	Age-standardized	Breast cancer	Rate	49.42461515
Incidence	Guyana	Age-standardized	Cervical cancer	Rate	38.84534467
Incidence	Guyana	Age-standardized	Uterine cancer	Rate	14.78991241
Incidence	Guyana	Age-standardized	Ovarian cancer	Rate	11.84323657
Incidence	Zimbabwe	Age-standardized	Breast cancer	Rate	37.62948833
Incidence	Zimbabwe	Age-standardized	Cervical cancer	Rate	48.94584633
Incidence	Zimbabwe	Age-standardized	Uterine cancer	Rate	8.960742821
Incidence	Niue	Age-standardized	Breast cancer	Rate	67.62880895
Incidence	Niue	Age-standardized	Cervical cancer	Rate	24.90884489
Incidence	Niue	Age-standardized	Uterine cancer	Rate	17.92314644
Incidence	Malta	Age-standardized	Breast cancer	Rate	80.51689353
Incidence	Malta	Age-standardized	Cervical cancer	Rate	4.942845613
Incidence	Mauritania	Age-standardized	Ovarian cancer	Rate	4.988226926
Incidence	Malta	Age-standardized	Uterine cancer	Rate	17.31802202
Incidence	Zimbabwe	Age-standardized	Ovarian cancer	Rate	9.745830174
Incidence	Guinea-Bissau	Age-standardized	Breast cancer	Rate	29.45010027
Incidence	Guinea-Bissau	Age-standardized	Cervical cancer	Rate	44.76757739
Incidence	Guinea-Bissau	Age-standardized	Uterine cancer	Rate	4.242374043
Incidence	Malta	Age-standardized	Ovarian cancer	Rate	11.42630601
Incidence	Eswatini	Age-standardized	Breast cancer	Rate	31.60797289
Incidence	Palau	Age-standardized	Breast cancer	Rate	75.30053094
Incidence	Palau	Age-standardized	Cervical cancer	Rate	66.57855531
Incidence	Palau	Age-standardized	Uterine cancer	Rate	1.996828575
Incidence	Rwanda	Age-standardized	Breast cancer	Rate	27.26336588
Incidence	Rwanda	Age-standardized	Cervical cancer	Rate	32.38649397
Incidence	Rwanda	Age-standardized	Uterine cancer	Rate	4.057266619
Incidence	Rwanda	Age-standardized	Ovarian cancer	Rate	7.894166734

Incidence	Liberia	Age-standardized	Breast cancer	Rate	25.5063389
Incidence	Liberia	Age-standardized	Cervical cancer	Rate	30.98942686
Incidence	Liberia	Age-standardized	Uterine cancer	Rate	3.707297149
Incidence	Netherlands	Age-standardized	Breast cancer	Rate	111.4929873
Incidence	Netherlands	Age-standardized	Cervical cancer	Rate	6.973286526
Incidence	Netherlands	Age-standardized	Uterine cancer	Rate	23.02469632
Incidence	Netherlands	Age-standardized	Ovarian cancer	Rate	11.56373552
Incidence	Seychelles	Age-standardized	Breast cancer	Rate	55.22482148
Incidence	Seychelles	Age-standardized	Cervical cancer	Rate	33.51003085
Incidence	Seychelles	Age-standardized	Uterine cancer	Rate	10.31886135
DALYs (Disabi	Belize	Age-standardized	Breast cancer	Rate	356.3365116
DALYs (Disabi	Belize	Age-standardized	Cervical cancer	Rate	573.6980942
DALYs (Disabi	Belize	Age-standardized	Uterine cancer	Rate	122.7413965
DALYs (Disabi	Belize	Age-standardized	Ovarian cancer	Rate	83.9781553
DALYs (Disabi	Lao People's Democratic Republic	Age-standardized	Breast cancer	Rate	650.1109608
DALYs (Disabi	Lao People's Democratic Republic	Age-standardized	Cervical cancer	Rate	289.7587327
DALYs (Disabi	Lao People's Democratic Republic	Age-standardized	Uterine cancer	Rate	83.49221445
DALYs (Disabi	Mexico	Age-standardized	Breast cancer	Rate	381.1271245
DALYs (Disabi	Mexico	Age-standardized	Cervical cancer	Rate	282.987736
DALYs (Disabi	Mexico	Age-standardized	Uterine cancer	Rate	39.40431338
DALYs (Disabi	Bosnia and Herzegovina	Age-standardized	Breast cancer	Rate	577.7508609
DALYs (Disabi	Bosnia and Herzegovina	Age-standardized	Cervical cancer	Rate	179.3958491
DALYs (Disabi	Bosnia and Herzegovina	Age-standardized	Uterine cancer	Rate	86.83010712
DALYs (Disabi	Puerto Rico	Age-standardized	Uterine cancer	Rate	72.51064002
DALYs (Disabi	Puerto Rico	Age-standardized	Ovarian cancer	Rate	115.6279318
DALYs (Disabi	Kiribati	Age-standardized	Cervical cancer	Rate	2143.060649
DALYs (Disabi	Kiribati	Age-standardized	Uterine cancer	Rate	134.5182724
DALYs (Disabi	Mexico	Age-standardized	Ovarian cancer	Rate	153.1582381
DALYs (Disabi	Kiribati	Age-standardized	Breast cancer	Rate	1041.567613
DALYs (Disabi	Lao People's Democratic Republic	Age-standardized	Ovarian cancer	Rate	159.7175071
DALYs (Disabi	Trinidad and Tobago	Age-standardized	Breast cancer	Rate	687.2837808
DALYs (Disabi	Trinidad and Tobago	Age-standardized	Cervical cancer	Rate	341.2743647
DALYs (Disabi	Trinidad and Tobago	Age-standardized	Uterine cancer	Rate	151.2645276
DALYs (Disabi	Trinidad and Tobago	Age-standardized	Ovarian cancer	Rate	192.2058995
DALYs (Disabi	Georgia	Age-standardized	Breast cancer	Rate	866.5904577
DALYs (Disabi	Georgia	Age-standardized	Cervical cancer	Rate	261.6406722
DALYs (Disabi	Georgia	Age-standardized	Uterine cancer	Rate	169.3087137
DALYs (Disabi	Saudi Arabia	Age-standardized	Breast cancer	Rate	446.1473168
DALYs (Disabi	Saudi Arabia	Age-standardized	Cervical cancer	Rate	64.91805636
DALYs (Disabi	Saudi Arabia	Age-standardized	Uterine cancer	Rate	39.81265231
DALYs (Disabi	Cuba	Age-standardized	Ovarian cancer	Rate	109.1694561
DALYs (Disabi	Bahrain	Age-standardized	Ovarian cancer	Rate	187.1272878
DALYs (Disabi	Egypt	Age-standardized	Breast cancer	Rate	436.2262003
DALYs (Disabi	Egypt	Age-standardized	Cervical cancer	Rate	45.12678509
DALYs (Disabi	Egypt	Age-standardized	Uterine cancer	Rate	40.63419528
DALYs (Disabi	Qatar	Age-standardized	Breast cancer	Rate	856.375117
DALYs (Disabi	Qatar	Age-standardized	Cervical cancer	Rate	104.1769731
DALYs (Disabi	Qatar	Age-standardized	Uterine cancer	Rate	50.63986701
DALYs (Disabi	Nicaragua	Age-standardized	Cervical cancer	Rate	465.4197568
DALYs (Disabi	Nicaragua	Age-standardized	Uterine cancer	Rate	39.57803226
DALYs (Disabi	Nicaragua	Age-standardized	Ovarian cancer	Rate	101.7927527
DALYs (Disabi	Saint Kitts and Nevis	Age-standardized	Ovarian cancer	Rate	188.1661962
DALYs (Disabi	Azerbaijan	Age-standardized	Breast cancer	Rate	550.2306066
DALYs (Disabi	Kiribati	Age-standardized	Ovarian cancer	Rate	78.7263123
DALYs (Disabi	Bulgaria	Age-standardized	Breast cancer	Rate	698.4902825
DALYs (Disabi	Bulgaria	Age-standardized	Cervical cancer	Rate	293.8873179
DALYs (Disabi	Bulgaria	Age-standardized	Uterine cancer	Rate	150.3728838
DALYs (Disabi	Bulgaria	Age-standardized	Ovarian cancer	Rate	230.7887508

DALYs (Disabi	Cook Islands	Age-standardized	Ovarian cancer	Rate	80.43775889
DALYs (Disabi	Qatar	Age-standardized	Ovarian cancer	Rate	195.6205678
DALYs (Disabi	Azerbaijan	Age-standardized	Cervical cancer	Rate	188.3475445
DALYs (Disabi	Azerbaijan	Age-standardized	Uterine cancer	Rate	78.96424052
DALYs (Disabi	Azerbaijan	Age-standardized	Ovarian cancer	Rate	112.726338
DALYs (Disabi	Malaysia	Age-standardized	Breast cancer	Rate	757.2384691
DALYs (Disabi	Malaysia	Age-standardized	Cervical cancer	Rate	243.9089574
DALYs (Disabi	Malaysia	Age-standardized	Uterine cancer	Rate	64.17244495
DALYs (Disabi	Luxembourg	Age-standardized	Cervical cancer	Rate	55.33842527
DALYs (Disabi	Luxembourg	Age-standardized	Uterine cancer	Rate	73.21580458
DALYs (Disabi	Slovenia	Age-standardized	Breast cancer	Rate	472.5853139
DALYs (Disabi	Slovenia	Age-standardized	Cervical cancer	Rate	87.44922422
DALYs (Disabi	Slovenia	Age-standardized	Uterine cancer	Rate	70.21139883
DALYs (Disabi	Bhutan	Age-standardized	Breast cancer	Rate	347.8971104
DALYs (Disabi	Bhutan	Age-standardized	Cervical cancer	Rate	222.6095379
DALYs (Disabi	Bhutan	Age-standardized	Uterine cancer	Rate	33.32697954
DALYs (Disabi	Bhutan	Age-standardized	Ovarian cancer	Rate	138.972793
DALYs (Disabi	Slovenia	Age-standardized	Ovarian cancer	Rate	178.9027159
DALYs (Disabi	Luxembourg	Age-standardized	Ovarian cancer	Rate	182.5631602
DALYs (Disabi	Belgium	Age-standardized	Breast cancer	Rate	629.7690375
DALYs (Disabi	Belgium	Age-standardized	Cervical cancer	Rate	78.16205323
DALYs (Disabi	Belgium	Age-standardized	Uterine cancer	Rate	62.75038111
DALYs (Disabi	Mauritius	Age-standardized	Breast cancer	Rate	678.214583
DALYs (Disabi	Mauritius	Age-standardized	Cervical cancer	Rate	162.8563261
DALYs (Disabi	Mauritius	Age-standardized	Uterine cancer	Rate	85.37910151
DALYs (Disabi	Maldives	Age-standardized	Breast cancer	Rate	373.4176143
DALYs (Disabi	Maldives	Age-standardized	Cervical cancer	Rate	110.4845259
DALYs (Disabi	Maldives	Age-standardized	Uterine cancer	Rate	29.02217338
DALYs (Disabi	Maldives	Age-standardized	Ovarian cancer	Rate	156.8319292
DALYs (Disabi	Malaysia	Age-standardized	Ovarian cancer	Rate	136.7876978
DALYs (Disabi	C么te d'Ivoire	Age-standardized	Breast cancer	Rate	448.7621575
DALYs (Disabi	Mauritius	Age-standardized	Ovarian cancer	Rate	160.4691185
DALYs (Disabi	Kazakhstan	Age-standardized	Breast cancer	Rate	486.4028347
DALYs (Disabi	Kazakhstan	Age-standardized	Cervical cancer	Rate	262.0073562
DALYs (Disabi	Kazakhstan	Age-standardized	Uterine cancer	Rate	88.59056975
DALYs (Disabi	Egypt	Age-standardized	Ovarian cancer	Rate	64.7873235
DALYs (Disabi	Marshall Islands	Age-standardized	Breast cancer	Rate	1202.900492
DALYs (Disabi	Marshall Islands	Age-standardized	Cervical cancer	Rate	696.4388036
DALYs (Disabi	Marshall Islands	Age-standardized	Uterine cancer	Rate	184.5331898
DALYs (Disabi	Equatorial Guinea	Age-standardized	Ovarian cancer	Rate	108.0064774
DALYs (Disabi	Panama	Age-standardized	Ovarian cancer	Rate	110.3827335
DALYs (Disabi	Bolivia (Plurinational State of)	Age-standardized	Ovarian cancer	Rate	130.1060083
DALYs (Disabi	Namibia	Age-standardized	Ovarian cancer	Rate	103.11389
DALYs (Disabi	Puerto Rico	Age-standardized	Breast cancer	Rate	515.6481532
DALYs (Disabi	Syrian Arab Republic	Age-standardized	Breast cancer	Rate	334.0492568
DALYs (Disabi	Syrian Arab Republic	Age-standardized	Cervical cancer	Rate	46.56422498
DALYs (Disabi	Syrian Arab Republic	Age-standardized	Uterine cancer	Rate	27.99467394
DALYs (Disabi	Croatia	Age-standardized	Breast cancer	Rate	521.2985583
DALYs (Disabi	Croatia	Age-standardized	Cervical cancer	Rate	108.5460889
DALYs (Disabi	Brunei Darussalam	Age-standardized	Breast cancer	Rate	737.82324
DALYs (Disabi	Brunei Darussalam	Age-standardized	Cervical cancer	Rate	305.1289391
DALYs (Disabi	Brunei Darussalam	Age-standardized	Uterine cancer	Rate	76.35512527
DALYs (Disabi	Gabon	Age-standardized	Breast cancer	Rate	729.2275913
DALYs (Disabi	Gabon	Age-standardized	Cervical cancer	Rate	500.1592866
DALYs (Disabi	Gabon	Age-standardized	Uterine cancer	Rate	57.80226157
DALYs (Disabi	Gambia	Age-standardized	Breast cancer	Rate	354.5370893
DALYs (Disabi	Gambia	Age-standardized	Cervical cancer	Rate	523.791287
DALYs (Disabi	Mozambique	Age-standardized	Cervical cancer	Rate	915.0423578

DALYs (Disabi	Mozambique	Age-standardized	Uterine cancer	Rate	65.85999529
DALYs (Disabi	Croatia	Age-standardized	Ovarian cancer	Rate	183.0171421
DALYs (Disabi	Luxembourg	Age-standardized	Breast cancer	Rate	523.6286572
DALYs (Disabi	Saudi Arabia	Age-standardized	Ovarian cancer	Rate	113.2607251
DALYs (Disabi	Marshall Islands	Age-standardized	Ovarian cancer	Rate	146.3881567
DALYs (Disabi	Iran (Islamic Republic of)	Age-standardized	Breast cancer	Rate	368.7090065
DALYs (Disabi	Iran (Islamic Republic of)	Age-standardized	Cervical cancer	Rate	54.11468571
DALYs (Disabi	Iran (Islamic Republic of)	Age-standardized	Uterine cancer	Rate	25.04656341
DALYs (Disabi	Belgium	Age-standardized	Ovarian cancer	Rate	150.3945749
DALYs (Disabi	Gambia	Age-standardized	Uterine cancer	Rate	43.9744063
DALYs (Disabi	Namibia	Age-standardized	Breast cancer	Rate	866.4333931
DALYs (Disabi	Namibia	Age-standardized	Cervical cancer	Rate	486.5302851
DALYs (Disabi	Namibia	Age-standardized	Uterine cancer	Rate	52.6391603
DALYs (Disabi	Georgia	Age-standardized	Ovarian cancer	Rate	194.4793235
DALYs (Disabi	Malta	Age-standardized	Ovarian cancer	Rate	176.0597725
DALYs (Disabi	Cuba	Age-standardized	Breast cancer	Rate	481.6065185
DALYs (Disabi	Cuba	Age-standardized	Cervical cancer	Rate	242.7844998
DALYs (Disabi	Cuba	Age-standardized	Uterine cancer	Rate	166.3663592
DALYs (Disabi	Senegal	Age-standardized	Breast cancer	Rate	602.6338413
DALYs (Disabi	Senegal	Age-standardized	Cervical cancer	Rate	594.1383969
DALYs (Disabi	Senegal	Age-standardized	Uterine cancer	Rate	56.10756719
DALYs (Disabi	Gabon	Age-standardized	Ovarian cancer	Rate	134.6247125
DALYs (Disabi	India	Age-standardized	Breast cancer	Rate	416.3760711
DALYs (Disabi	India	Age-standardized	Cervical cancer	Rate	239.4866232
DALYs (Disabi	India	Age-standardized	Uterine cancer	Rate	29.87006564
DALYs (Disabi	Mozambique	Age-standardized	Breast cancer	Rate	587.3865996
DALYs (Disabi	Cyprus	Age-standardized	Breast cancer	Rate	592.2251943
DALYs (Disabi	Brunei Darussalam	Age-standardized	Ovarian cancer	Rate	281.9858508
DALYs (Disabi	Mozambique	Age-standardized	Ovarian cancer	Rate	137.3877067
DALYs (Disabi	Iran (Islamic Republic of)	Age-standardized	Ovarian cancer	Rate	85.35071236
DALYs (Disabi	Croatia	Age-standardized	Uterine cancer	Rate	83.02398833
DALYs (Disabi	Cyprus	Age-standardized	Cervical cancer	Rate	72.27887527
DALYs (Disabi	Cyprus	Age-standardized	Uterine cancer	Rate	69.35472221
DALYs (Disabi	Japan	Age-standardized	Breast cancer	Rate	338.0881443
DALYs (Disabi	Japan	Age-standardized	Cervical cancer	Rate	90.9878711
DALYs (Disabi	Japan	Age-standardized	Uterine cancer	Rate	50.94707963
DALYs (Disabi	Senegal	Age-standardized	Ovarian cancer	Rate	92.58268401
DALYs (Disabi	Chile	Age-standardized	Breast cancer	Rate	377.2036755
DALYs (Disabi	Estonia	Age-standardized	Breast cancer	Rate	500.6137786
DALYs (Disabi	Estonia	Age-standardized	Cervical cancer	Rate	160.8160545
DALYs (Disabi	Estonia	Age-standardized	Uterine cancer	Rate	79.47212763
DALYs (Disabi	Nepal	Age-standardized	Breast cancer	Rate	549.9366262
DALYs (Disabi	Nepal	Age-standardized	Cervical cancer	Rate	259.0567389
DALYs (Disabi	Nepal	Age-standardized	Uterine cancer	Rate	31.43463236
DALYs (Disabi	C么te d'Ivoire	Age-standardized	Ovarian cancer	Rate	91.0950654
DALYs (Disabi	Nicaragua	Age-standardized	Breast cancer	Rate	335.654079
DALYs (Disabi	Kyrgyzstan	Age-standardized	Breast cancer	Rate	317.4315242
DALYs (Disabi	Kyrgyzstan	Age-standardized	Cervical cancer	Rate	291.7775604
DALYs (Disabi	Kyrgyzstan	Age-standardized	Uterine cancer	Rate	76.37569284
DALYs (Disabi	Kyrgyzstan	Age-standardized	Ovarian cancer	Rate	143.3431947
DALYs (Disabi	Chile	Age-standardized	Cervical cancer	Rate	212.5792848
DALYs (Disabi	Chile	Age-standardized	Uterine cancer	Rate	45.50324831
DALYs (Disabi	Chile	Age-standardized	Ovarian cancer	Rate	127.454412
DALYs (Disabi	Cyprus	Age-standardized	Ovarian cancer	Rate	155.7260569
DALYs (Disabi	Papua New Guinea	Age-standardized	Breast cancer	Rate	1467.979286
DALYs (Disabi	Papua New Guinea	Age-standardized	Cervical cancer	Rate	451.6271592
DALYs (Disabi	Papua New Guinea	Age-standardized	Uterine cancer	Rate	99.18453925
DALYs (Disabi	Sierra Leone	Age-standardized	Breast cancer	Rate	512.5828675

DALYs (Disabi	Dominica	Age-standardized	Breast cancer	Rate	862.5082116
DALYs (Disabi	Malta	Age-standardized	Breast cancer	Rate	589.2954841
DALYs (Disabi	Malta	Age-standardized	Cervical cancer	Rate	50.94210092
DALYs (Disabi	Malta	Age-standardized	Uterine cancer	Rate	63.34244228
DALYs (Disabi	Myanmar	Age-standardized	Ovarian cancer	Rate	155.7055008
DALYs (Disabi	Saint Kitts and Nevis	Age-standardized	Breast cancer	Rate	802.7833845
DALYs (Disabi	Saint Kitts and Nevis	Age-standardized	Cervical cancer	Rate	415.7296659
DALYs (Disabi	Saint Kitts and Nevis	Age-standardized	Uterine cancer	Rate	159.3755207
DALYs (Disabi	Syrian Arab Republic	Age-standardized	Ovarian cancer	Rate	66.45536197
DALYs (Disabi	Czechia	Age-standardized	Ovarian cancer	Rate	195.2104742
DALYs (Disabi	India	Age-standardized	Ovarian cancer	Rate	104.7925819
DALYs (Disabi	Myanmar	Age-standardized	Breast cancer	Rate	505.6948018
DALYs (Disabi	Myanmar	Age-standardized	Cervical cancer	Rate	247.2787548
DALYs (Disabi	Myanmar	Age-standardized	Uterine cancer	Rate	71.83633913
DALYs (Disabi	Eswatini	Age-standardized	Breast cancer	Rate	614.4410054
DALYs (Disabi	Eswatini	Age-standardized	Cervical cancer	Rate	794.7402591
DALYs (Disabi	Eswatini	Age-standardized	Uterine cancer	Rate	74.2931416
DALYs (Disabi	Kazakhstan	Age-standardized	Ovarian cancer	Rate	194.8750916
DALYs (Disabi	Belarus	Age-standardized	Ovarian cancer	Rate	171.4523742
DALYs (Disabi	Sierra Leone	Age-standardized	Cervical cancer	Rate	691.2758388
DALYs (Disabi	Sierra Leone	Age-standardized	Uterine cancer	Rate	55.38264322
DALYs (Disabi	Japan	Age-standardized	Ovarian cancer	Rate	109.8825929
DALYs (Disabi	Ghana	Age-standardized	Ovarian cancer	Rate	109.4151743
DALYs (Disabi	Denmark	Age-standardized	Breast cancer	Rate	591.186807
DALYs (Disabi	Denmark	Age-standardized	Cervical cancer	Rate	94.17169468
DALYs (Disabi	Denmark	Age-standardized	Uterine cancer	Rate	62.49900589
DALYs (Disabi	Rwanda	Age-standardized	Breast cancer	Rate	547.9154177
DALYs (Disabi	Rwanda	Age-standardized	Cervical cancer	Rate	643.9123081
DALYs (Disabi	Rwanda	Age-standardized	Uterine cancer	Rate	58.76386788
DALYs (Disabi	Netherlands	Age-standardized	Breast cancer	Rate	669.6901376
DALYs (Disabi	Netherlands	Age-standardized	Cervical cancer	Rate	65.3424395
DALYs (Disabi	Netherlands	Age-standardized	Uterine cancer	Rate	71.55486642
DALYs (Disabi	Tunisia	Age-standardized	Breast cancer	Rate	483.5488237
DALYs (Disabi	Tunisia	Age-standardized	Cervical cancer	Rate	75.11852824
DALYs (Disabi	Tunisia	Age-standardized	Uterine cancer	Rate	27.26583362
DALYs (Disabi	Tunisia	Age-standardized	Ovarian cancer	Rate	96.93969076
DALYs (Disabi	Belarus	Age-standardized	Breast cancer	Rate	433.5164617
DALYs (Disabi	Belarus	Age-standardized	Cervical cancer	Rate	194.8290028
DALYs (Disabi	Belarus	Age-standardized	Uterine cancer	Rate	60.75968329
DALYs (Disabi	Burundi	Age-standardized	Ovarian cancer	Rate	105.8199858
DALYs (Disabi	Nepal	Age-standardized	Ovarian cancer	Rate	126.0004094
DALYs (Disabi	Guinea	Age-standardized	Ovarian cancer	Rate	120.7305592
DALYs (Disabi	Finland	Age-standardized	Ovarian cancer	Rate	162.9549044
DALYs (Disabi	France	Age-standardized	Breast cancer	Rate	566.323883
DALYs (Disabi	France	Age-standardized	Cervical cancer	Rate	82.26507035
DALYs (Disabi	France	Age-standardized	Uterine cancer	Rate	62.75129286
DALYs (Disabi	Guam	Age-standardized	Breast cancer	Rate	514.2255263
DALYs (Disabi	Guam	Age-standardized	Cervical cancer	Rate	187.8013235
DALYs (Disabi	Guam	Age-standardized	Uterine cancer	Rate	85.3832458
DALYs (Disabi	Finland	Age-standardized	Breast cancer	Rate	464.9899814
DALYs (Disabi	Finland	Age-standardized	Cervical cancer	Rate	47.49138549
DALYs (Disabi	Finland	Age-standardized	Uterine cancer	Rate	68.58368403
DALYs (Disabi	United States of America	Age-standardized	Breast cancer	Rate	536.4357377
DALYs (Disabi	Seychelles	Age-standardized	Uterine cancer	Rate	78.69937313
DALYs (Disabi	Seychelles	Age-standardized	Ovarian cancer	Rate	263.4475596
DALYs (Disabi	Pakistan	Age-standardized	Breast cancer	Rate	1570.061314
DALYs (Disabi	Pakistan	Age-standardized	Cervical cancer	Rate	149.9342262
DALYs (Disabi	Pakistan	Age-standardized	Uterine cancer	Rate	120.2300266

DALYs (Disabi	South Africa	Age-standardized	Ovarian cancer	Rate	124.6442261
DALYs (Disabi	Latvia	Age-standardized	Ovarian cancer	Rate	256.6087689
DALYs (Disabi	Hungary	Age-standardized	Breast cancer	Rate	555.7899381
DALYs (Disabi	Hungary	Age-standardized	Cervical cancer	Rate	170.0566444
DALYs (Disabi	Hungary	Age-standardized	Uterine cancer	Rate	80.39001194
DALYs (Disabi	Hungary	Age-standardized	Ovarian cancer	Rate	200.6901505
DALYs (Disabi	Jordan	Age-standardized	Ovarian cancer	Rate	98.27271564
DALYs (Disabi	Guinea	Age-standardized	Breast cancer	Rate	551.9964421
DALYs (Disabi	Guinea	Age-standardized	Cervical cancer	Rate	1143.795742
DALYs (Disabi	Guinea	Age-standardized	Uterine cancer	Rate	61.02380304
DALYs (Disabi	Zimbabwe	Age-standardized	Breast cancer	Rate	782.3117509
DALYs (Disabi	Zimbabwe	Age-standardized	Cervical cancer	Rate	957.2227383
DALYs (Disabi	Zimbabwe	Age-standardized	Uterine cancer	Rate	133.8173758
DALYs (Disabi	Burundi	Age-standardized	Breast cancer	Rate	465.6136695
DALYs (Disabi	Burundi	Age-standardized	Cervical cancer	Rate	818.3689554
DALYs (Disabi	Burundi	Age-standardized	Uterine cancer	Rate	58.76047314
DALYs (Disabi	Micronesia (Federated States of)	Age-standardized	Breast cancer	Rate	1238.217131
DALYs (Disabi	Micronesia (Federated States of)	Age-standardized	Cervical cancer	Rate	622.057796
DALYs (Disabi	Micronesia (Federated States of)	Age-standardized	Uterine cancer	Rate	191.7551879
DALYs (Disabi	Greece	Age-standardized	Breast cancer	Rate	582.8997621
DALYs (Disabi	Greece	Age-standardized	Cervical cancer	Rate	86.06407751
DALYs (Disabi	Greece	Age-standardized	Uterine cancer	Rate	70.45889055
DALYs (Disabi	Uruguay	Age-standardized	Breast cancer	Rate	810.4926712
DALYs (Disabi	Monaco	Age-standardized	Breast cancer	Rate	925.2069647
DALYs (Disabi	Monaco	Age-standardized	Cervical cancer	Rate	80.38804706
DALYs (Disabi	Monaco	Age-standardized	Uterine cancer	Rate	31.60109075
DALYs (Disabi	Dominican Republic	Age-standardized	Breast cancer	Rate	481.114551
DALYs (Disabi	Venezuela (Bolivarian Republic of)	Age-standardized	Ovarian cancer	Rate	133.4951147
DALYs (Disabi	Germany	Age-standardized	Breast cancer	Rate	574.1360175
DALYs (Disabi	Germany	Age-standardized	Cervical cancer	Rate	86.55695989
DALYs (Disabi	Germany	Age-standardized	Uterine cancer	Rate	53.73774322
DALYs (Disabi	Norway	Age-standardized	Breast cancer	Rate	423.1928387
DALYs (Disabi	Norway	Age-standardized	Cervical cancer	Rate	80.42818281
DALYs (Disabi	Spain	Age-standardized	Cervical cancer	Rate	71.20383263
DALYs (Disabi	Spain	Age-standardized	Uterine cancer	Rate	69.1028746
DALYs (Disabi	Spain	Age-standardized	Ovarian cancer	Rate	125.1416821
DALYs (Disabi	Bolivia (Plurinational State of)	Age-standardized	Breast cancer	Rate	536.085377
DALYs (Disabi	Bolivia (Plurinational State of)	Age-standardized	Cervical cancer	Rate	682.1334193
DALYs (Disabi	Bolivia (Plurinational State of)	Age-standardized	Uterine cancer	Rate	126.6426057
DALYs (Disabi	Panama	Age-standardized	Breast cancer	Rate	400.8867626
DALYs (Disabi	Panama	Age-standardized	Cervical cancer	Rate	321.0390061
DALYs (Disabi	Panama	Age-standardized	Uterine cancer	Rate	65.68209731
DALYs (Disabi	Iceland	Age-standardized	Breast cancer	Rate	414.4150214
DALYs (Disabi	Iceland	Age-standardized	Cervical cancer	Rate	54.9269956
DALYs (Disabi	Iceland	Age-standardized	Uterine cancer	Rate	39.98362102
DALYs (Disabi	Guinea-Bissau	Age-standardized	Breast cancer	Rate	665.6509697
DALYs (Disabi	Guinea-Bissau	Age-standardized	Cervical cancer	Rate	937.6330135
DALYs (Disabi	Guinea-Bissau	Age-standardized	Uterine cancer	Rate	69.78111533
DALYs (Disabi	Cook Islands	Age-standardized	Breast cancer	Rate	1034.306039
DALYs (Disabi	Cook Islands	Age-standardized	Cervical cancer	Rate	111.0896105
DALYs (Disabi	Cook Islands	Age-standardized	Uterine cancer	Rate	49.52862942
DALYs (Disabi	Dominica	Age-standardized	Cervical cancer	Rate	535.7157683
DALYs (Disabi	Dominica	Age-standardized	Uterine cancer	Rate	113.3481636
DALYs (Disabi	Dominica	Age-standardized	Ovarian cancer	Rate	94.63742494
DALYs (Disabi	Papua New Guinea	Age-standardized	Ovarian cancer	Rate	77.01516115
DALYs (Disabi	South Africa	Age-standardized	Breast cancer	Rate	529.9076776
DALYs (Disabi	South Africa	Age-standardized	Cervical cancer	Rate	500.4380126
DALYs (Disabi	South Africa	Age-standardized	Uterine cancer	Rate	49.59939672

DALYs (Disabi	Czechia	Age-standardized	Breast cancer	Rate	428.2691402
DALYs (Disabi	Czechia	Age-standardized	Cervical cancer	Rate	132.8093833
DALYs (Disabi	Czechia	Age-standardized	Uterine cancer	Rate	88.39183839
DALYs (Disabi	Ghana	Age-standardized	Breast cancer	Rate	863.4649555
DALYs (Disabi	Ghana	Age-standardized	Cervical cancer	Rate	516.8483283
DALYs (Disabi	Ghana	Age-standardized	Uterine cancer	Rate	66.35460828
DALYs (Disabi	Rwanda	Age-standardized	Ovarian cancer	Rate	175.0693408
DALYs (Disabi	North Macedonia	Age-standardized	Ovarian cancer	Rate	198.0535995
DALYs (Disabi	Uruguay	Age-standardized	Ovarian cancer	Rate	175.074666
DALYs (Disabi	Samoa	Age-standardized	Breast cancer	Rate	744.3422117
DALYs (Disabi	Samoa	Age-standardized	Cervical cancer	Rate	409.1436161
DALYs (Disabi	Samoa	Age-standardized	Uterine cancer	Rate	116.0584687
DALYs (Disabi	Republic of Korea	Age-standardized	Ovarian cancer	Rate	81.44059955
DALYs (Disabi	Iceland	Age-standardized	Ovarian cancer	Rate	138.3005098
DALYs (Disabi	Spain	Age-standardized	Breast cancer	Rate	443.7930454
DALYs (Disabi	Latvia	Age-standardized	Breast cancer	Rate	540.2903537
DALYs (Disabi	Latvia	Age-standardized	Cervical cancer	Rate	134.7033283
DALYs (Disabi	Latvia	Age-standardized	Uterine cancer	Rate	115.3889647
DALYs (Disabi	China	Age-standardized	Breast cancer	Rate	277.9804449
DALYs (Disabi	China	Age-standardized	Cervical cancer	Rate	157.5044883
DALYs (Disabi	China	Age-standardized	Uterine cancer	Rate	34.92583289
DALYs (Disabi	Iraq	Age-standardized	Breast cancer	Rate	714.8507201
DALYs (Disabi	Iraq	Age-standardized	Cervical cancer	Rate	70.19509911
DALYs (Disabi	Iraq	Age-standardized	Uterine cancer	Rate	53.33056734
DALYs (Disabi	Iraq	Age-standardized	Ovarian cancer	Rate	115.7588719
DALYs (Disabi	Comoros	Age-standardized	Breast cancer	Rate	554.5232651
DALYs (Disabi	Comoros	Age-standardized	Cervical cancer	Rate	707.0151604
DALYs (Disabi	Comoros	Age-standardized	Uterine cancer	Rate	68.37447684
DALYs (Disabi	Pakistan	Age-standardized	Ovarian cancer	Rate	348.3691668
DALYs (Disabi	Greenland	Age-standardized	Breast cancer	Rate	554.3174226
DALYs (Disabi	Greenland	Age-standardized	Cervical cancer	Rate	298.2062672
DALYs (Disabi	Greenland	Age-standardized	Uterine cancer	Rate	30.79857751
DALYs (Disabi	Ecuador	Age-standardized	Breast cancer	Rate	380.0577079
DALYs (Disabi	Ecuador	Age-standardized	Cervical cancer	Rate	383.7860955
DALYs (Disabi	Ecuador	Age-standardized	Uterine cancer	Rate	93.37282061
DALYs (Disabi	Gambia	Age-standardized	Ovarian cancer	Rate	97.70116493
DALYs (Disabi	Somalia	Age-standardized	Breast cancer	Rate	375.0737604
DALYs (Disabi	Somalia	Age-standardized	Cervical cancer	Rate	1013.759927
DALYs (Disabi	Somalia	Age-standardized	Uterine cancer	Rate	62.61224499
DALYs (Disabi	Republic of Korea	Age-standardized	Breast cancer	Rate	281.1901529
DALYs (Disabi	Republic of Korea	Age-standardized	Cervical cancer	Rate	77.89686554
DALYs (Disabi	Republic of Korea	Age-standardized	Uterine cancer	Rate	21.63467718
DALYs (Disabi	Haiti	Age-standardized	Breast cancer	Rate	884.6278079
DALYs (Disabi	Haiti	Age-standardized	Cervical cancer	Rate	913.0645778
DALYs (Disabi	Haiti	Age-standardized	Uterine cancer	Rate	153.4824403
DALYs (Disabi	Singapore	Age-standardized	Breast cancer	Rate	386.853509
DALYs (Disabi	Singapore	Age-standardized	Cervical cancer	Rate	71.94865183
DALYs (Disabi	Micronesia (Federated States of)	Age-standardized	Ovarian cancer	Rate	164.7324485
DALYs (Disabi	Mongolia	Age-standardized	Ovarian cancer	Rate	130.0998752
DALYs (Disabi	Denmark	Age-standardized	Ovarian cancer	Rate	195.2441417
DALYs (Disabi	Australia	Age-standardized	Breast cancer	Rate	492.1068482
DALYs (Disabi	Australia	Age-standardized	Cervical cancer	Rate	64.4122478
DALYs (Disabi	Australia	Age-standardized	Uterine cancer	Rate	52.57159757
DALYs (Disabi	Estonia	Age-standardized	Ovarian cancer	Rate	191.3774309
DALYs (Disabi	Djibouti	Age-standardized	Ovarian cancer	Rate	166.0124987
DALYs (Disabi	Norway	Age-standardized	Uterine cancer	Rate	64.06525987
DALYs (Disabi	Lithuania	Age-standardized	Breast cancer	Rate	497.666308
DALYs (Disabi	Lithuania	Age-standardized	Cervical cancer	Rate	166.855191

DALYs (Disabi	Lithuania	Age-standardized	Uterine cancer	Rate	102.4291662
DALYs (Disabi	Lithuania	Age-standardized	Ovarian cancer	Rate	255.9062864
DALYs (Disabi	Turkey	Age-standardized	Ovarian cancer	Rate	119.5854243
DALYs (Disabi	Portugal	Age-standardized	Breast cancer	Rate	482.3434435
DALYs (Disabi	Portugal	Age-standardized	Cervical cancer	Rate	96.3238331
DALYs (Disabi	Portugal	Age-standardized	Uterine cancer	Rate	67.28968017
DALYs (Disabi	Comoros	Age-standardized	Ovarian cancer	Rate	185.0736378
DALYs (Disabi	Canada	Age-standardized	Breast cancer	Rate	511.5405689
DALYs (Disabi	Canada	Age-standardized	Cervical cancer	Rate	77.30536814
DALYs (Disabi	Canada	Age-standardized	Uterine cancer	Rate	66.68150623
DALYs (Disabi	Eswatini	Age-standardized	Ovarian cancer	Rate	172.0833179
DALYs (Disabi	Benin	Age-standardized	Breast cancer	Rate	444.8186613
DALYs (Disabi	Benin	Age-standardized	Cervical cancer	Rate	608.3578415
DALYs (Disabi	Benin	Age-standardized	Uterine cancer	Rate	53.6443283
DALYs (Disabi	Philippines	Age-standardized	Breast cancer	Rate	711.6866204
DALYs (Disabi	Philippines	Age-standardized	Cervical cancer	Rate	226.7533562
DALYs (Disabi	Philippines	Age-standardized	Uterine cancer	Rate	73.95017978
DALYs (Disabi	Philippines	Age-standardized	Ovarian cancer	Rate	183.6741435
DALYs (Disabi	Djibouti	Age-standardized	Breast cancer	Rate	545.4486907
DALYs (Disabi	Djibouti	Age-standardized	Cervical cancer	Rate	675.4636289
DALYs (Disabi	Djibouti	Age-standardized	Uterine cancer	Rate	69.68486925
DALYs (Disabi	Nauru	Age-standardized	Breast cancer	Rate	1235.040626
DALYs (Disabi	Nauru	Age-standardized	Cervical cancer	Rate	612.1837822
DALYs (Disabi	Nauru	Age-standardized	Uterine cancer	Rate	191.0643643
DALYs (Disabi	Netherlands	Age-standardized	Ovarian cancer	Rate	173.484706
DALYs (Disabi	Canada	Age-standardized	Ovarian cancer	Rate	144.4926302
DALYs (Disabi	United Republic of Tanzania	Age-standardized	Breast cancer	Rate	471.8343235
DALYs (Disabi	United Republic of Tanzania	Age-standardized	Cervical cancer	Rate	702.4210324
DALYs (Disabi	United Republic of Tanzania	Age-standardized	Uterine cancer	Rate	66.37565168
DALYs (Disabi	China	Age-standardized	Ovarian cancer	Rate	80.52279061
DALYs (Disabi	Dominican Republic	Age-standardized	Cervical cancer	Rate	405.5114063
DALYs (Disabi	Dominican Republic	Age-standardized	Uterine cancer	Rate	143.3240647
DALYs (Disabi	Venezuela (Bolivarian Republic of)	Age-standardized	Breast cancer	Rate	537.7115436
DALYs (Disabi	Venezuela (Bolivarian Republic of)	Age-standardized	Cervical cancer	Rate	505.7049182
DALYs (Disabi	Venezuela (Bolivarian Republic of)	Age-standardized	Uterine cancer	Rate	67.84077914
DALYs (Disabi	France	Age-standardized	Ovarian cancer	Rate	146.6785442
DALYs (Disabi	Uruguay	Age-standardized	Cervical cancer	Rate	275.046357
DALYs (Disabi	Uruguay	Age-standardized	Uterine cancer	Rate	55.2300441
DALYs (Disabi	Norway	Age-standardized	Ovarian cancer	Rate	194.3344868
DALYs (Disabi	Singapore	Age-standardized	Uterine cancer	Rate	50.64417041
DALYs (Disabi	Singapore	Age-standardized	Ovarian cancer	Rate	113.0879843
DALYs (Disabi	Mali	Age-standardized	Breast cancer	Rate	503.3037913
DALYs (Disabi	Mali	Age-standardized	Cervical cancer	Rate	580.5221665
DALYs (Disabi	Mali	Age-standardized	Uterine cancer	Rate	41.86834797
DALYs (Disabi	Eritrea	Age-standardized	Cervical cancer	Rate	973.5832728
DALYs (Disabi	Eritrea	Age-standardized	Uterine cancer	Rate	81.72071059
DALYs (Disabi	Eritrea	Age-standardized	Ovarian cancer	Rate	148.9855747
DALYs (Disabi	Republic of Moldova	Age-standardized	Ovarian cancer	Rate	139.4160123
DALYs (Disabi	San Marino	Age-standardized	Ovarian cancer	Rate	112.8578487
DALYs (Disabi	Sierra Leone	Age-standardized	Ovarian cancer	Rate	82.13772749
DALYs (Disabi	Seychelles	Age-standardized	Breast cancer	Rate	761.5601796
DALYs (Disabi	Seychelles	Age-standardized	Cervical cancer	Rate	468.4890368
DALYs (Disabi	Togo	Age-standardized	Breast cancer	Rate	498.6511106
DALYs (Disabi	Togo	Age-standardized	Cervical cancer	Rate	609.52696
DALYs (Disabi	Togo	Age-standardized	Uterine cancer	Rate	52.05176214
DALYs (Disabi	Liberia	Age-standardized	Breast cancer	Rate	528.2260412
DALYs (Disabi	Liberia	Age-standardized	Cervical cancer	Rate	620.589718
DALYs (Disabi	Liberia	Age-standardized	Uterine cancer	Rate	55.55387848

DALYs (Disabi	Somalia	Age-standardized	Ovarian cancer	Rate	95.85402876
DALYs (Disabi	Greenland	Age-standardized	Ovarian cancer	Rate	258.8547673
DALYs (Disabi	Germany	Age-standardized	Ovarian cancer	Rate	156.6907417
DALYs (Disabi	Grenada	Age-standardized	Breast cancer	Rate	857.9992658
DALYs (Disabi	Grenada	Age-standardized	Cervical cancer	Rate	525.9146811
DALYs (Disabi	Grenada	Age-standardized	Uterine cancer	Rate	283.0854762
DALYs (Disabi	San Marino	Age-standardized	Breast cancer	Rate	556.5949723
DALYs (Disabi	San Marino	Age-standardized	Cervical cancer	Rate	68.39912091
DALYs (Disabi	San Marino	Age-standardized	Uterine cancer	Rate	19.99959201
DALYs (Disabi	Portugal	Age-standardized	Ovarian cancer	Rate	102.2414431
DALYs (Disabi	Dominican Republic	Age-standardized	Ovarian cancer	Rate	50.30137088
DALYs (Disabi	Peru	Age-standardized	Breast cancer	Rate	318.2720845
DALYs (Disabi	Peru	Age-standardized	Cervical cancer	Rate	366.0306927
DALYs (Disabi	Peru	Age-standardized	Uterine cancer	Rate	70.28012911
DALYs (Disabi	Solomon Islands	Age-standardized	Breast cancer	Rate	2635.742894
DALYs (Disabi	Solomon Islands	Age-standardized	Cervical cancer	Rate	1018.693697
DALYs (Disabi	Solomon Islands	Age-standardized	Uterine cancer	Rate	221.030714
DALYs (Disabi	Togo	Age-standardized	Ovarian cancer	Rate	86.28510378
DALYs (Disabi	Uganda	Age-standardized	Breast cancer	Rate	656.1138659
DALYs (Disabi	Uganda	Age-standardized	Cervical cancer	Rate	756.6595616
DALYs (Disabi	Uganda	Age-standardized	Uterine cancer	Rate	94.41111961
DALYs (Disabi	Uganda	Age-standardized	Ovarian cancer	Rate	195.1631076
DALYs (Disabi	Antigua and Barbuda	Age-standardized	Cervical cancer	Rate	308.7189016
DALYs (Disabi	Antigua and Barbuda	Age-standardized	Uterine cancer	Rate	130.6183923
DALYs (Disabi	Antigua and Barbuda	Age-standardized	Ovarian cancer	Rate	176.2306482
DALYs (Disabi	Antigua and Barbuda	Age-standardized	Breast cancer	Rate	809.1877906
DALYs (Disabi	New Zealand	Age-standardized	Ovarian cancer	Rate	147.9536167
DALYs (Disabi	Switzerland	Age-standardized	Breast cancer	Rate	481.326635
DALYs (Disabi	Switzerland	Age-standardized	Cervical cancer	Rate	58.09268703
DALYs (Disabi	Switzerland	Age-standardized	Uterine cancer	Rate	42.92681383
DALYs (Disabi	Guinea-Bissau	Age-standardized	Ovarian cancer	Rate	97.81244756
DALYs (Disabi	New Zealand	Age-standardized	Breast cancer	Rate	598.9842122
DALYs (Disabi	New Zealand	Age-standardized	Cervical cancer	Rate	71.07539181
DALYs (Disabi	New Zealand	Age-standardized	Uterine cancer	Rate	75.6585138
DALYs (Disabi	Benin	Age-standardized	Ovarian cancer	Rate	76.20774365
DALYs (Disabi	Samoa	Age-standardized	Ovarian cancer	Rate	245.9736421
DALYs (Disabi	Sweden	Age-standardized	Breast cancer	Rate	489.4760082
DALYs (Disabi	Sweden	Age-standardized	Cervical cancer	Rate	75.63562333
DALYs (Disabi	Monaco	Age-standardized	Ovarian cancer	Rate	342.074609
DALYs (Disabi	Sudan	Age-standardized	Cervical cancer	Rate	93.65950688
DALYs (Disabi	Sudan	Age-standardized	Uterine cancer	Rate	25.31519459
DALYs (Disabi	Mauritania	Age-standardized	Breast cancer	Rate	488.2337713
DALYs (Disabi	Mauritania	Age-standardized	Cervical cancer	Rate	523.8332153
DALYs (Disabi	Mauritania	Age-standardized	Uterine cancer	Rate	59.95154639
DALYs (Disabi	Solomon Islands	Age-standardized	Ovarian cancer	Rate	118.6981096
DALYs (Disabi	Sweden	Age-standardized	Uterine cancer	Rate	56.55482894
DALYs (Disabi	Zimbabwe	Age-standardized	Ovarian cancer	Rate	210.8445587
DALYs (Disabi	Ecuador	Age-standardized	Ovarian cancer	Rate	136.9769584
DALYs (Disabi	Cambodia	Age-standardized	Breast cancer	Rate	442.6861638
DALYs (Disabi	Cambodia	Age-standardized	Cervical cancer	Rate	305.9785677
DALYs (Disabi	Cambodia	Age-standardized	Uterine cancer	Rate	82.80500145
DALYs (Disabi	United States of America	Age-standardized	Cervical cancer	Rate	98.77152306
DALYs (Disabi	United States of America	Age-standardized	Uterine cancer	Rate	87.87574002
DALYs (Disabi	Ireland	Age-standardized	Cervical cancer	Rate	90.29859467
DALYs (Disabi	Ireland	Age-standardized	Uterine cancer	Rate	65.55381445
DALYs (Disabi	Ireland	Age-standardized	Ovarian cancer	Rate	197.8995412
DALYs (Disabi	Barbados	Age-standardized	Breast cancer	Rate	999.71467
DALYs (Disabi	Barbados	Age-standardized	Cervical cancer	Rate	388.5629376

DALYs (Disabi	Barbados	Age-standardized	Uterine cancer	Rate	186.6589516
DALYs (Disabi	Sri Lanka	Age-standardized	Ovarian cancer	Rate	92.54902803
DALYs (Disabi	Liberia	Age-standardized	Ovarian cancer	Rate	100.2616508
DALYs (Disabi	United States of America	Age-standardized	Ovarian cancer	Rate	157.747758
DALYs (Disabi	Northern Mariana Islands	Age-standardized	Breast cancer	Rate	772.9555089
DALYs (Disabi	Northern Mariana Islands	Age-standardized	Cervical cancer	Rate	461.6268293
DALYs (Disabi	Northern Mariana Islands	Age-standardized	Uterine cancer	Rate	207.3435233
DALYs (Disabi	Montenegro	Age-standardized	Ovarian cancer	Rate	166.1486618
DALYs (Disabi	Democratic People's Republic of Korea	Age-standardized	Ovarian cancer	Rate	84.1311424
DALYs (Disabi	Tokelau	Age-standardized	Breast cancer	Rate	1006.955532
DALYs (Disabi	Tokelau	Age-standardized	Cervical cancer	Rate	508.5850951
DALYs (Disabi	Tokelau	Age-standardized	Uterine cancer	Rate	154.8643172
DALYs (Disabi	Bahamas	Age-standardized	Breast cancer	Rate	1087.226266
DALYs (Disabi	Bahamas	Age-standardized	Cervical cancer	Rate	354.2824551
DALYs (Disabi	Bahamas	Age-standardized	Uterine cancer	Rate	139.6714645
DALYs (Disabi	Sri Lanka	Age-standardized	Breast cancer	Rate	345.0086456
DALYs (Disabi	Sri Lanka	Age-standardized	Cervical cancer	Rate	100.9961037
DALYs (Disabi	Sri Lanka	Age-standardized	Uterine cancer	Rate	41.62441042
DALYs (Disabi	Guam	Age-standardized	Ovarian cancer	Rate	114.0134545
DALYs (Disabi	Tuvalu	Age-standardized	Breast cancer	Rate	1042.579393
DALYs (Disabi	Tuvalu	Age-standardized	Cervical cancer	Rate	536.1445533
DALYs (Disabi	Tuvalu	Age-standardized	Uterine cancer	Rate	150.2862483
DALYs (Disabi	Sudan	Age-standardized	Breast cancer	Rate	416.4201479
DALYs (Disabi	Jamaica	Age-standardized	Breast cancer	Rate	911.2490931
DALYs (Disabi	Jamaica	Age-standardized	Cervical cancer	Rate	511.6469098
DALYs (Disabi	Jamaica	Age-standardized	Uterine cancer	Rate	165.0978358
DALYs (Disabi	Turkey	Age-standardized	Breast cancer	Rate	369.8446992
DALYs (Disabi	Turkey	Age-standardized	Cervical cancer	Rate	61.83610362
DALYs (Disabi	Turkey	Age-standardized	Uterine cancer	Rate	53.88554941
DALYs (Disabi	American Samoa	Age-standardized	Ovarian cancer	Rate	278.7337085
DALYs (Disabi	Ethiopia	Age-standardized	Ovarian cancer	Rate	117.3980159
DALYs (Disabi	Burkina Faso	Age-standardized	Breast cancer	Rate	566.51387
DALYs (Disabi	Burkina Faso	Age-standardized	Cervical cancer	Rate	698.9534143
DALYs (Disabi	Burkina Faso	Age-standardized	Uterine cancer	Rate	55.11720106
DALYs (Disabi	Grenada	Age-standardized	Ovarian cancer	Rate	216.5234743
DALYs (Disabi	Democratic People's Republic of Korea	Age-standardized	Breast cancer	Rate	426.5674612
DALYs (Disabi	Greece	Age-standardized	Ovarian cancer	Rate	158.1347515
DALYs (Disabi	Niger	Age-standardized	Cervical cancer	Rate	711.5250307
DALYs (Disabi	Niger	Age-standardized	Uterine cancer	Rate	48.87095745
DALYs (Disabi	Niger	Age-standardized	Ovarian cancer	Rate	49.53880961
DALYs (Disabi	Zambia	Age-standardized	Ovarian cancer	Rate	167.9078236
DALYs (Disabi	Angola	Age-standardized	Ovarian cancer	Rate	73.37045007
DALYs (Disabi	United Republic of Tanzania	Age-standardized	Ovarian cancer	Rate	151.669373
DALYs (Disabi	American Samoa	Age-standardized	Breast cancer	Rate	890.8135962
DALYs (Disabi	American Samoa	Age-standardized	Cervical cancer	Rate	287.21072
DALYs (Disabi	American Samoa	Age-standardized	Uterine cancer	Rate	285.4055742
DALYs (Disabi	Guyana	Age-standardized	Breast cancer	Rate	766.2182698
DALYs (Disabi	Guyana	Age-standardized	Cervical cancer	Rate	683.1402242
DALYs (Disabi	Guyana	Age-standardized	Uterine cancer	Rate	184.3469288
DALYs (Disabi	Israel	Age-standardized	Ovarian cancer	Rate	153.7408083
DALYs (Disabi	Democratic People's Republic of Korea	Age-standardized	Cervical cancer	Rate	279.5573335
DALYs (Disabi	Democratic People's Republic of Korea	Age-standardized	Uterine cancer	Rate	63.84344581
DALYs (Disabi	Lebanon	Age-standardized	Breast cancer	Rate	1066.954105
DALYs (Disabi	Guyana	Age-standardized	Ovarian cancer	Rate	247.6338799
DALYs (Disabi	Niger	Age-standardized	Breast cancer	Rate	315.1571376
DALYs (Disabi	Colombia	Age-standardized	Breast cancer	Rate	360.924658
DALYs (Disabi	Colombia	Age-standardized	Cervical cancer	Rate	265.4999339
DALYs (Disabi	Colombia	Age-standardized	Uterine cancer	Rate	38.94841749

DALYs (Disabi	Niue	Age-standardized	Breast cancer	Rate	872.2395475
DALYs (Disabi	Niue	Age-standardized	Cervical cancer	Rate	333.9566021
DALYs (Disabi	Niue	Age-standardized	Uterine cancer	Rate	126.1295912
DALYs (Disabi	Jordan	Age-standardized	Breast cancer	Rate	583.5729255
DALYs (Disabi	Jordan	Age-standardized	Cervical cancer	Rate	55.24808984
DALYs (Disabi	Jordan	Age-standardized	Uterine cancer	Rate	48.39428394
DALYs (Disabi	Sweden	Age-standardized	Ovarian cancer	Rate	157.1692328
DALYs (Disabi	Indonesia	Age-standardized	Breast cancer	Rate	704.6979142
DALYs (Disabi	Indonesia	Age-standardized	Cervical cancer	Rate	216.8703525
DALYs (Disabi	Guatemala	Age-standardized	Breast cancer	Rate	306.5892625
DALYs (Disabi	Palau	Age-standardized	Breast cancer	Rate	972.6922906
DALYs (Disabi	Palau	Age-standardized	Cervical cancer	Rate	896.4367432
DALYs (Disabi	Palau	Age-standardized	Uterine cancer	Rate	14.0745499
DALYs (Disabi	Palau	Age-standardized	Ovarian cancer	Rate	80.99365076
DALYs (Disabi	Bahamas	Age-standardized	Ovarian cancer	Rate	218.315555
DALYs (Disabi	Tokelau	Age-standardized	Ovarian cancer	Rate	114.1350906
DALYs (Disabi	United Kingdom	Age-standardized	Breast cancer	Rate	613.7783565
DALYs (Disabi	United Kingdom	Age-standardized	Cervical cancer	Rate	90.25644893
DALYs (Disabi	United Kingdom	Age-standardized	Uterine cancer	Rate	74.20116979
DALYs (Disabi	Australia	Age-standardized	Ovarian cancer	Rate	124.2873694
DALYs (Disabi	Colombia	Age-standardized	Ovarian cancer	Rate	136.9088642
DALYs (Disabi	Cameroon	Age-standardized	Breast cancer	Rate	618.7724895
DALYs (Disabi	Cameroon	Age-standardized	Cervical cancer	Rate	635.1578323
DALYs (Disabi	Cameroon	Age-standardized	Uterine cancer	Rate	63.7116204
DALYs (Disabi	Cameroon	Age-standardized	Ovarian cancer	Rate	114.7497875
DALYs (Disabi	Mali	Age-standardized	Ovarian cancer	Rate	57.64040609
DALYs (Disabi	United Kingdom	Age-standardized	Ovarian cancer	Rate	191.439697
DALYs (Disabi	Tonga	Age-standardized	Ovarian cancer	Rate	116.3689718
DALYs (Disabi	Andorra	Age-standardized	Ovarian cancer	Rate	116.1497478
DALYs (Disabi	Costa Rica	Age-standardized	Breast cancer	Rate	432.5238603
DALYs (Disabi	Costa Rica	Age-standardized	Cervical cancer	Rate	222.0447782
DALYs (Disabi	Costa Rica	Age-standardized	Uterine cancer	Rate	59.70256546
DALYs (Disabi	Nauru	Age-standardized	Ovarian cancer	Rate	140.7954767
DALYs (Disabi	Thailand	Age-standardized	Breast cancer	Rate	394.5783079
DALYs (Disabi	Thailand	Age-standardized	Cervical cancer	Rate	205.2891381
DALYs (Disabi	Thailand	Age-standardized	Uterine cancer	Rate	31.39819553
DALYs (Disabi	Bermuda	Age-standardized	Cervical cancer	Rate	93.43985087
DALYs (Disabi	Bermuda	Age-standardized	Uterine cancer	Rate	76.22558238
DALYs (Disabi	Bermuda	Age-standardized	Ovarian cancer	Rate	164.1988684
DALYs (Disabi	United Arab Emirates	Age-standardized	Breast cancer	Rate	790.9777657
DALYs (Disabi	United Arab Emirates	Age-standardized	Cervical cancer	Rate	168.6165172
DALYs (Disabi	United Arab Emirates	Age-standardized	Uterine cancer	Rate	38.63187532
DALYs (Disabi	Turkmenistan	Age-standardized	Breast cancer	Rate	427.3999156
DALYs (Disabi	Brazil	Age-standardized	Ovarian cancer	Rate	116.135008
DALYs (Disabi	Mongolia	Age-standardized	Breast cancer	Rate	292.7239542
DALYs (Disabi	Mongolia	Age-standardized	Cervical cancer	Rate	363.4905092
DALYs (Disabi	Mongolia	Age-standardized	Uterine cancer	Rate	66.24694353
DALYs (Disabi	Eritrea	Age-standardized	Breast cancer	Rate	644.0830206
DALYs (Disabi	Cabo Verde	Age-standardized	Ovarian cancer	Rate	76.08347037
DALYs (Disabi	Indonesia	Age-standardized	Uterine cancer	Rate	76.18473298
DALYs (Disabi	Indonesia	Age-standardized	Ovarian cancer	Rate	153.6746656
DALYs (Disabi	Saint Lucia	Age-standardized	Breast cancer	Rate	637.4117913
DALYs (Disabi	Saint Lucia	Age-standardized	Cervical cancer	Rate	426.9700265
DALYs (Disabi	North Macedonia	Age-standardized	Breast cancer	Rate	744.5413477
DALYs (Disabi	North Macedonia	Age-standardized	Cervical cancer	Rate	190.8400241
DALYs (Disabi	North Macedonia	Age-standardized	Uterine cancer	Rate	135.6834799
DALYs (Disabi	Nigeria	Age-standardized	Ovarian cancer	Rate	90.72389574
DALYs (Disabi	Saint Vincent and the Grenadines	Age-standardized	Breast cancer	Rate	891.931253

DALYs (Disabi	Saint Vincent and the Grenadines	Age-standardized	Cervical cancer	Rate	653.6073299
DALYs (Disabi	Saint Vincent and the Grenadines	Age-standardized	Uterine cancer	Rate	205.6819829
DALYs (Disabi	Saint Lucia	Age-standardized	Uterine cancer	Rate	117.5638343
DALYs (Disabi	Saint Lucia	Age-standardized	Ovarian cancer	Rate	177.5755028
DALYs (Disabi	United Arab Emirates	Age-standardized	Ovarian cancer	Rate	163.1314028
DALYs (Disabi	Peru	Age-standardized	Ovarian cancer	Rate	134.3387539
DALYs (Disabi	Libya	Age-standardized	Breast cancer	Rate	550.4624682
DALYs (Disabi	Libya	Age-standardized	Cervical cancer	Rate	124.016138
DALYs (Disabi	Libya	Age-standardized	Uterine cancer	Rate	47.00037949
DALYs (Disabi	Costa Rica	Age-standardized	Ovarian cancer	Rate	107.8583165
DALYs (Disabi	Argentina	Age-standardized	Ovarian cancer	Rate	165.4096676
DALYs (Disabi	Vanuatu	Age-standardized	Ovarian cancer	Rate	87.76037056
DALYs (Disabi	Burkina Faso	Age-standardized	Ovarian cancer	Rate	74.97077098
DALYs (Disabi	Puerto Rico	Age-standardized	Cervical cancer	Rate	124.5872132
DALYs (Disabi	Niue	Age-standardized	Ovarian cancer	Rate	142.1833493
DALYs (Disabi	Honduras	Age-standardized	Breast cancer	Rate	380.016534
DALYs (Disabi	Honduras	Age-standardized	Cervical cancer	Rate	343.7329994
DALYs (Disabi	Honduras	Age-standardized	Uterine cancer	Rate	108.1365641
DALYs (Disabi	Kuwait	Age-standardized	Breast cancer	Rate	358.8442246
DALYs (Disabi	Kuwait	Age-standardized	Cervical cancer	Rate	44.34094027
DALYs (Disabi	Switzerland	Age-standardized	Ovarian cancer	Rate	128.32449
DALYs (Disabi	Tuvalu	Age-standardized	Ovarian cancer	Rate	131.4670299
DALYs (Disabi	South Sudan	Age-standardized	Breast cancer	Rate	358.4631097
DALYs (Disabi	South Sudan	Age-standardized	Cervical cancer	Rate	566.6392845
DALYs (Disabi	South Sudan	Age-standardized	Uterine cancer	Rate	56.85598535
DALYs (Disabi	Mauritania	Age-standardized	Ovarian cancer	Rate	108.2583383
DALYs (Disabi	Haiti	Age-standardized	Ovarian cancer	Rate	100.4460343
DALYs (Disabi	Taiwan (Province of China)	Age-standardized	Breast cancer	Rate	385.9604504
DALYs (Disabi	Taiwan (Province of China)	Age-standardized	Cervical cancer	Rate	133.3381224
DALYs (Disabi	Taiwan (Province of China)	Age-standardized	Uterine cancer	Rate	51.8770367
DALYs (Disabi	United States Virgin Islands	Age-standardized	Breast cancer	Rate	754.9005886
DALYs (Disabi	United States Virgin Islands	Age-standardized	Cervical cancer	Rate	223.8798521
DALYs (Disabi	United States Virgin Islands	Age-standardized	Uterine cancer	Rate	96.75716761
DALYs (Disabi	Kuwait	Age-standardized	Uterine cancer	Rate	49.04832386
DALYs (Disabi	Kuwait	Age-standardized	Ovarian cancer	Rate	95.12939616
DALYs (Disabi	South Sudan	Age-standardized	Ovarian cancer	Rate	108.8531462
DALYs (Disabi	Taiwan (Province of China)	Age-standardized	Ovarian cancer	Rate	116.5289944
DALYs (Disabi	Algeria	Age-standardized	Breast cancer	Rate	419.2684564
DALYs (Disabi	Algeria	Age-standardized	Cervical cancer	Rate	133.1621134
DALYs (Disabi	Algeria	Age-standardized	Uterine cancer	Rate	16.76140154
DALYs (Disabi	El Salvador	Age-standardized	Breast cancer	Rate	327.7204454
DALYs (Disabi	El Salvador	Age-standardized	Cervical cancer	Rate	463.1046826
DALYs (Disabi	El Salvador	Age-standardized	Uterine cancer	Rate	55.96877444
DALYs (Disabi	El Salvador	Age-standardized	Ovarian cancer	Rate	117.386623
DALYs (Disabi	Sudan	Age-standardized	Ovarian cancer	Rate	63.39513814
DALYs (Disabi	Tajikistan	Age-standardized	Breast cancer	Rate	450.238502
DALYs (Disabi	Barbados	Age-standardized	Ovarian cancer	Rate	180.5333859
DALYs (Disabi	Yemen	Age-standardized	Cervical cancer	Rate	117.5663468
DALYs (Disabi	Yemen	Age-standardized	Uterine cancer	Rate	25.74402032
DALYs (Disabi	Thailand	Age-standardized	Ovarian cancer	Rate	113.0566895
DALYs (Disabi	Brazil	Age-standardized	Breast cancer	Rate	449.4409759
DALYs (Disabi	Brazil	Age-standardized	Cervical cancer	Rate	268.0006953
DALYs (Disabi	Brazil	Age-standardized	Uterine cancer	Rate	56.77983152
DALYs (Disabi	United States Virgin Islands	Age-standardized	Ovarian cancer	Rate	262.2690775
DALYs (Disabi	Montenegro	Age-standardized	Breast cancer	Rate	773.2941924
DALYs (Disabi	Montenegro	Age-standardized	Cervical cancer	Rate	161.3679681
DALYs (Disabi	Montenegro	Age-standardized	Uterine cancer	Rate	79.44597197
DALYs (Disabi	Northern Mariana Islands	Age-standardized	Ovarian cancer	Rate	126.2592005

DALYs (Disabi	Sao Tome and Principe	Age-standardized	Ovarian cancer	Rate	201.5577556
DALYs (Disabi	Republic of Moldova	Age-standardized	Breast cancer	Rate	459.8931886
DALYs (Disabi	Republic of Moldova	Age-standardized	Cervical cancer	Rate	218.9583108
DALYs (Disabi	Republic of Moldova	Age-standardized	Uterine cancer	Rate	83.96175481
DALYs (Disabi	Guatemala	Age-standardized	Cervical cancer	Rate	559.0640621
DALYs (Disabi	Guatemala	Age-standardized	Uterine cancer	Rate	68.73980637
DALYs (Disabi	Tajikistan	Age-standardized	Cervical cancer	Rate	136.3858172
DALYs (Disabi	Tajikistan	Age-standardized	Uterine cancer	Rate	112.1392093
DALYs (Disabi	Tajikistan	Age-standardized	Ovarian cancer	Rate	115.7668355
DALYs (Disabi	Suriname	Age-standardized	Breast cancer	Rate	556.8280737
DALYs (Disabi	Suriname	Age-standardized	Cervical cancer	Rate	543.4551096
DALYs (Disabi	Suriname	Age-standardized	Uterine cancer	Rate	69.225122
DALYs (Disabi	Viet Nam	Age-standardized	Breast cancer	Rate	653.3610476
DALYs (Disabi	Saint Vincent and the Grenadines	Age-standardized	Ovarian cancer	Rate	178.3344339
DALYs (Disabi	Guatemala	Age-standardized	Ovarian cancer	Rate	96.41710377
DALYs (Disabi	Ethiopia	Age-standardized	Breast cancer	Rate	466.5717543
DALYs (Disabi	Ethiopia	Age-standardized	Cervical cancer	Rate	497.9436926
DALYs (Disabi	Ethiopia	Age-standardized	Uterine cancer	Rate	42.67045436
DALYs (Disabi	Timor-Leste	Age-standardized	Breast cancer	Rate	491.2076975
DALYs (Disabi	Timor-Leste	Age-standardized	Cervical cancer	Rate	278.9410368
DALYs (Disabi	Timor-Leste	Age-standardized	Uterine cancer	Rate	75.83301425
DALYs (Disabi	Bahrain	Age-standardized	Breast cancer	Rate	668.3430986
DALYs (Disabi	Bahrain	Age-standardized	Cervical cancer	Rate	74.42357461
DALYs (Disabi	Bahrain	Age-standardized	Uterine cancer	Rate	54.07688371
DALYs (Disabi	Uzbekistan	Age-standardized	Breast cancer	Rate	535.9523766
DALYs (Disabi	Uzbekistan	Age-standardized	Cervical cancer	Rate	276.8507425
DALYs (Disabi	Uzbekistan	Age-standardized	Uterine cancer	Rate	81.08431459
DALYs (Disabi	Morocco	Age-standardized	Breast cancer	Rate	842.511459
DALYs (Disabi	Morocco	Age-standardized	Cervical cancer	Rate	236.5127694
DALYs (Disabi	Morocco	Age-standardized	Uterine cancer	Rate	42.85785525
DALYs (Disabi	Tonga	Age-standardized	Breast cancer	Rate	953.961714
DALYs (Disabi	Tonga	Age-standardized	Cervical cancer	Rate	504.2699355
DALYs (Disabi	Tonga	Age-standardized	Uterine cancer	Rate	96.38350965
DALYs (Disabi	Albania	Age-standardized	Breast cancer	Rate	375.4941171
DALYs (Disabi	Palestine	Age-standardized	Breast cancer	Rate	738.916044
DALYs (Disabi	Palestine	Age-standardized	Cervical cancer	Rate	69.82851018
DALYs (Disabi	Palestine	Age-standardized	Uterine cancer	Rate	99.51259939
DALYs (Disabi	Jamaica	Age-standardized	Ovarian cancer	Rate	157.0439797
DALYs (Disabi	Timor-Leste	Age-standardized	Ovarian cancer	Rate	110.3408492
DALYs (Disabi	Yemen	Age-standardized	Ovarian cancer	Rate	56.52996371
DALYs (Disabi	Morocco	Age-standardized	Ovarian cancer	Rate	156.6560684
DALYs (Disabi	Suriname	Age-standardized	Ovarian cancer	Rate	178.4139626
DALYs (Disabi	Lebanon	Age-standardized	Cervical cancer	Rate	71.83175742
DALYs (Disabi	Lebanon	Age-standardized	Uterine cancer	Rate	62.09807105
DALYs (Disabi	Russian Federation	Age-standardized	Breast cancer	Rate	509.2921706
DALYs (Disabi	Russian Federation	Age-standardized	Cervical cancer	Rate	199.8726838
DALYs (Disabi	Russian Federation	Age-standardized	Uterine cancer	Rate	115.4662637
DALYs (Disabi	Lebanon	Age-standardized	Ovarian cancer	Rate	201.4876233
DALYs (Disabi	Paraguay	Age-standardized	Breast cancer	Rate	528.3665278
DALYs (Disabi	Paraguay	Age-standardized	Cervical cancer	Rate	535.0893542
DALYs (Disabi	Paraguay	Age-standardized	Uterine cancer	Rate	77.9658753
DALYs (Disabi	Honduras	Age-standardized	Ovarian cancer	Rate	115.5135574
DALYs (Disabi	Bangladesh	Age-standardized	Breast cancer	Rate	450.2364344
DALYs (Disabi	Bangladesh	Age-standardized	Cervical cancer	Rate	180.4208954
DALYs (Disabi	Bangladesh	Age-standardized	Uterine cancer	Rate	22.14754853
DALYs (Disabi	Armenia	Age-standardized	Breast cancer	Rate	704.484316
DALYs (Disabi	Armenia	Age-standardized	Cervical cancer	Rate	222.3303663
DALYs (Disabi	Armenia	Age-standardized	Uterine cancer	Rate	98.82665736

DALYs (Disabi	Yemen	Age-standardized	Breast cancer	Rate	434.074198
DALYs (Disabi	Turkmenistan	Age-standardized	Cervical cancer	Rate	250.3988805
DALYs (Disabi	Turkmenistan	Age-standardized	Uterine cancer	Rate	26.0712609
DALYs (Disabi	Congo	Age-standardized	Breast cancer	Rate	837.9378059
DALYs (Disabi	Congo	Age-standardized	Cervical cancer	Rate	751.5667398
DALYs (Disabi	Congo	Age-standardized	Uterine cancer	Rate	68.1509966
DALYs (Disabi	Russian Federation	Age-standardized	Ovarian cancer	Rate	204.2015403
DALYs (Disabi	Andorra	Age-standardized	Breast cancer	Rate	505.3690592
DALYs (Disabi	Andorra	Age-standardized	Cervical cancer	Rate	121.9964905
DALYs (Disabi	Andorra	Age-standardized	Uterine cancer	Rate	71.11449325
DALYs (Disabi	Turkmenistan	Age-standardized	Ovarian cancer	Rate	108.6425778
DALYs (Disabi	Oman	Age-standardized	Breast cancer	Rate	434.8267916
DALYs (Disabi	Oman	Age-standardized	Cervical cancer	Rate	78.00773941
DALYs (Disabi	Oman	Age-standardized	Uterine cancer	Rate	22.27606863
DALYs (Disabi	Kenya	Age-standardized	Breast cancer	Rate	480.0453675
DALYs (Disabi	Kenya	Age-standardized	Cervical cancer	Rate	395.3048801
DALYs (Disabi	Poland	Age-standardized	Breast cancer	Rate	525.269393
DALYs (Disabi	Poland	Age-standardized	Cervical cancer	Rate	178.2421035
DALYs (Disabi	Poland	Age-standardized	Uterine cancer	Rate	100.6183534
DALYs (Disabi	Romania	Age-standardized	Breast cancer	Rate	538.8675826
DALYs (Disabi	Romania	Age-standardized	Cervical cancer	Rate	364.4793529
DALYs (Disabi	Romania	Age-standardized	Uterine cancer	Rate	81.01984007
DALYs (Disabi	Angola	Age-standardized	Breast cancer	Rate	558.1185149
DALYs (Disabi	Angola	Age-standardized	Cervical cancer	Rate	618.6944462
DALYs (Disabi	Angola	Age-standardized	Uterine cancer	Rate	48.35094744
DALYs (Disabi	Poland	Age-standardized	Ovarian cancer	Rate	246.4905376
DALYs (Disabi	Kenya	Age-standardized	Uterine cancer	Rate	34.08932789
DALYs (Disabi	Kenya	Age-standardized	Ovarian cancer	Rate	133.9920327
DALYs (Disabi	Fiji	Age-standardized	Breast cancer	Rate	1074.442768
DALYs (Disabi	Fiji	Age-standardized	Cervical cancer	Rate	680.1336745
DALYs (Disabi	Fiji	Age-standardized	Uterine cancer	Rate	125.5852666
DALYs (Disabi	Fiji	Age-standardized	Ovarian cancer	Rate	56.9861345
DALYs (Disabi	Paraguay	Age-standardized	Ovarian cancer	Rate	106.7089259
DALYs (Disabi	Vanuatu	Age-standardized	Breast cancer	Rate	817.5754459
DALYs (Disabi	Vanuatu	Age-standardized	Cervical cancer	Rate	544.482377
DALYs (Disabi	Cambodia	Age-standardized	Ovarian cancer	Rate	161.4359372
DALYs (Disabi	Libya	Age-standardized	Ovarian cancer	Rate	141.3973731
DALYs (Disabi	Viet Nam	Age-standardized	Cervical cancer	Rate	248.9444029
DALYs (Disabi	Viet Nam	Age-standardized	Uterine cancer	Rate	32.31566549
DALYs (Disabi	Austria	Age-standardized	Breast cancer	Rate	472.2780356
DALYs (Disabi	Austria	Age-standardized	Cervical cancer	Rate	76.50937863
DALYs (Disabi	Vanuatu	Age-standardized	Uterine cancer	Rate	138.7713115
DALYs (Disabi	Viet Nam	Age-standardized	Ovarian cancer	Rate	120.9812447
DALYs (Disabi	Ukraine	Age-standardized	Breast cancer	Rate	620.1008818
DALYs (Disabi	Ukraine	Age-standardized	Cervical cancer	Rate	171.48658
DALYs (Disabi	Ukraine	Age-standardized	Uterine cancer	Rate	109.7781666
DALYs (Disabi	Palestine	Age-standardized	Ovarian cancer	Rate	125.7692608
DALYs (Disabi	Ukraine	Age-standardized	Ovarian cancer	Rate	203.8051897
DALYs (Disabi	Austria	Age-standardized	Uterine cancer	Rate	52.82048395
DALYs (Disabi	Austria	Age-standardized	Ovarian cancer	Rate	145.9921374
DALYs (Disabi	Central African Republic	Age-standardized	Breast cancer	Rate	593.1680308
DALYs (Disabi	Central African Republic	Age-standardized	Cervical cancer	Rate	955.2542494
DALYs (Disabi	Central African Republic	Age-standardized	Uterine cancer	Rate	56.27481638
DALYs (Disabi	Uzbekistan	Age-standardized	Ovarian cancer	Rate	106.8571315
DALYs (Disabi	Central African Republic	Age-standardized	Ovarian cancer	Rate	64.29829219
DALYs (Disabi	Madagascar	Age-standardized	Breast cancer	Rate	495.4112963
DALYs (Disabi	Madagascar	Age-standardized	Cervical cancer	Rate	718.8996213
DALYs (Disabi	Madagascar	Age-standardized	Uterine cancer	Rate	58.99618637

DALYs (Disabi	Cabo Verde	Age-standardized	Breast cancer	Rate	402.0795382
DALYs (Disabi	Cabo Verde	Age-standardized	Cervical cancer	Rate	365.1319893
DALYs (Disabi	Cabo Verde	Age-standardized	Uterine cancer	Rate	68.59186563
DALYs (Disabi	Romania	Age-standardized	Ovarian cancer	Rate	188.2872645
DALYs (Disabi	Argentina	Age-standardized	Breast cancer	Rate	749.0292867
DALYs (Disabi	Argentina	Age-standardized	Cervical cancer	Rate	366.909381
DALYs (Disabi	Argentina	Age-standardized	Uterine cancer	Rate	61.79862907
DALYs (Disabi	Madagascar	Age-standardized	Ovarian cancer	Rate	114.5947285
DALYs (Disabi	Serbia	Age-standardized	Breast cancer	Rate	787.7956099
DALYs (Disabi	Serbia	Age-standardized	Cervical cancer	Rate	272.0538464
DALYs (Disabi	Serbia	Age-standardized	Uterine cancer	Rate	107.5278982
DALYs (Disabi	Albania	Age-standardized	Cervical cancer	Rate	98.41873162
DALYs (Disabi	Albania	Age-standardized	Uterine cancer	Rate	59.38169563
DALYs (Disabi	Albania	Age-standardized	Ovarian cancer	Rate	85.52526656
DALYs (Disabi	Democratic Republic of the Congo	Age-standardized	Breast cancer	Rate	632.7862582
DALYs (Disabi	Democratic Republic of the Congo	Age-standardized	Cervical cancer	Rate	683.2130502
DALYs (Disabi	Democratic Republic of the Congo	Age-standardized	Uterine cancer	Rate	46.45921146
DALYs (Disabi	Zambia	Age-standardized	Breast cancer	Rate	553.414585
DALYs (Disabi	Zambia	Age-standardized	Cervical cancer	Rate	861.7035331
DALYs (Disabi	Zambia	Age-standardized	Uterine cancer	Rate	75.00655136
DALYs (Disabi	Congo	Age-standardized	Ovarian cancer	Rate	108.8715989
DALYs (Disabi	Slovakia	Age-standardized	Breast cancer	Rate	509.9471557
DALYs (Disabi	Slovakia	Age-standardized	Cervical cancer	Rate	170.2986113
DALYs (Disabi	Slovakia	Age-standardized	Uterine cancer	Rate	111.8846963
DALYs (Disabi	Oman	Age-standardized	Ovarian cancer	Rate	122.100296
DALYs (Disabi	Slovakia	Age-standardized	Ovarian cancer	Rate	182.121064
DALYs (Disabi	Afghanistan	Age-standardized	Breast cancer	Rate	506.1909532
DALYs (Disabi	Afghanistan	Age-standardized	Cervical cancer	Rate	238.6444916
DALYs (Disabi	Afghanistan	Age-standardized	Uterine cancer	Rate	51.1972066
DALYs (Disabi	Malawi	Age-standardized	Breast cancer	Rate	460.0050377
DALYs (Disabi	Malawi	Age-standardized	Cervical cancer	Rate	820.3325088
DALYs (Disabi	Malawi	Age-standardized	Uterine cancer	Rate	33.22994349
DALYs (Disabi	Serbia	Age-standardized	Ovarian cancer	Rate	223.7490286
DALYs (Disabi	Chad	Age-standardized	Breast cancer	Rate	402.6077839
DALYs (Disabi	Chad	Age-standardized	Cervical cancer	Rate	761.7058986
DALYs (Disabi	Chad	Age-standardized	Uterine cancer	Rate	55.63746677
DALYs (Disabi	Bermuda	Age-standardized	Breast cancer	Rate	514.8238878
DALYs (Disabi	Chad	Age-standardized	Ovarian cancer	Rate	52.54757967
DALYs (Disabi	Ireland	Age-standardized	Breast cancer	Rate	574.374913
DALYs (Disabi	Malawi	Age-standardized	Ovarian cancer	Rate	114.8103941
DALYs (Disabi	Democratic Republic of the Congo	Age-standardized	Ovarian cancer	Rate	68.0016605
DALYs (Disabi	Afghanistan	Age-standardized	Ovarian cancer	Rate	85.30511635
DALYs (Disabi	Nigeria	Age-standardized	Breast cancer	Rate	746.0966187
DALYs (Disabi	Nigeria	Age-standardized	Cervical cancer	Rate	359.8363072
DALYs (Disabi	Nigeria	Age-standardized	Uterine cancer	Rate	18.04216782
DALYs (Disabi	Bosnia and Herzegovina	Age-standardized	Ovarian cancer	Rate	210.1356722
DALYs (Disabi	Botswana	Age-standardized	Breast cancer	Rate	792.4549549
DALYs (Disabi	Botswana	Age-standardized	Cervical cancer	Rate	810.030517
DALYs (Disabi	Botswana	Age-standardized	Uterine cancer	Rate	83.37300258
DALYs (Disabi	Lesotho	Age-standardized	Breast cancer	Rate	783.952898
DALYs (Disabi	Lesotho	Age-standardized	Cervical cancer	Rate	1087.774299
DALYs (Disabi	Lesotho	Age-standardized	Uterine cancer	Rate	89.59775829
DALYs (Disabi	Botswana	Age-standardized	Ovarian cancer	Rate	162.9932515
DALYs (Disabi	Equatorial Guinea	Age-standardized	Breast cancer	Rate	695.4635583
DALYs (Disabi	Equatorial Guinea	Age-standardized	Cervical cancer	Rate	508.2955113
DALYs (Disabi	Equatorial Guinea	Age-standardized	Uterine cancer	Rate	54.70685256
DALYs (Disabi	C幺te d'Ivoire	Age-standardized	Cervical cancer	Rate	569.2435281
DALYs (Disabi	C幺te d'Ivoire	Age-standardized	Uterine cancer	Rate	52.14994015

DALYs (Disabi	Sao Tome and Principe	Age-standardized	Breast cancer	Rate	591.9741408
DALYs (Disabi	Sao Tome and Principe	Age-standardized	Cervical cancer	Rate	806.1985
DALYs (Disabi	Sao Tome and Principe	Age-standardized	Uterine cancer	Rate	104.626301
DALYs (Disabi	Armenia	Age-standardized	Ovarian cancer	Rate	147.3892435
DALYs (Disabi	Algeria	Age-standardized	Ovarian cancer	Rate	76.84077895
DALYs (Disabi	Israel	Age-standardized	Breast cancer	Rate	584.5824985
DALYs (Disabi	Israel	Age-standardized	Cervical cancer	Rate	72.51378959
DALYs (Disabi	Israel	Age-standardized	Uterine cancer	Rate	62.25342258
DALYs (Disabi	Bangladesh	Age-standardized	Ovarian cancer	Rate	94.54133555
DALYs (Disabi	Italy	Age-standardized	Breast cancer	Rate	530.7787229
DALYs (Disabi	Italy	Age-standardized	Cervical cancer	Rate	64.95906432
DALYs (Disabi	Italy	Age-standardized	Uterine cancer	Rate	63.16833331
DALYs (Disabi	Lesotho	Age-standardized	Ovarian cancer	Rate	169.1027674
DALYs (Disabi	Italy	Age-standardized	Ovarian cancer	Rate	145.8583161
